# Consolidation of working hours and work-life balance in anaesthesiologists – A cross-sectional national survey

**DOI:** 10.1371/journal.pone.0206050

**Published:** 2018-10-31

**Authors:** Wolfgang Lederer, Peter Paal, Daniel von Langen, Alice Sanwald, Christian Traweger, Johann F. Kinzl

**Affiliations:** 1 Medical University of Innsbruck, Department of Anaesthesiology and Critical Care, Innsbruck, Austria; 2 Department of Anaesthesiology and Intensive Care, Hospitallers Brothers Hospital, Paracelsus Medical University, Salzburg, Austria; 3 National Association of Statutory Health Insurance Funds, Berlin, Germany; 4 University of Innsbruck, Department of Statistics, Innsbruck, Austria; 5 Medical University of Innsbruck, Department of Psychiatry, Division of Psychosocial Psychiatry, Innsbruck, Austria; Universitat de Valencia, SPAIN

## Abstract

Currently, healthcare management fosters a maximization of performance despite a relative shortage of specialists. We evaluated anaesthesiologists’ workload, physical health, emotional well-being, job satisfaction and working conditions under increased pressure from consolidated working hours. A nationwide cross-sectional survey was performed in Austrian anaesthesiologists (overall response rate 41.0%). Three hundred and ninety four anaesthesiologists (280 specialists, 114 anaesthesiology trainees) participated. Anaesthesiologists reported frequently working under time pressure (95%CI: 65.6–74.6), at high working speed (95%CI: 57.6–67.1), with delayed or cancelled breaks (95%CI: 54.5–64.1), and with frequent overtime (95%CI: 42.6–52.4). Perceived work climate correlated with task conduct (manner of work accomplishment, the way in which tasks were completed), participation (decision-making power in joint consultation and teamwork), psychosocial resources, uncertainty, task variability and time tolerance (authority in time management and control over operating speed) (all P <0.001). Having not enough time for oneself (95%CI: 47.6–57.4), for sleep (95%CI: 45.6–55.4) or for one’s partner and children (95%CI: 21.8–30.4) was common. One-third of the participants reported frequent feelings of being unsettled (95%CI: 33.4–43.0) and difficulty talking about their emotions (95%CI: 27.3–36.5). Frequent dissatisfaction with life was reported by 11.4% (95%CI: 8.7–14.9) of the respondents. Strong time pressure and little decision-making authority during work along with long working hours and frequent work interruptions constitute the basis for occupational stress in anaesthesiologists. We conclude that increased pressure to perform during work hours contributes to emotional exhaustion and poor work-life balance. Changes in the work schedule of anaesthesiologists are required to avoid negative effects on health and emotional well-being.

## Introduction

Medical professionals are socially respected and held in high esteem. However, the public is generally not aware of what working in the healthcare system means. The extent of burden and stress from long working hours, night calls, the increasing pressure to perform in situations where staffing levels are inadequate, hard-nosed competition in scientific work, exposure to hazardous substances, radiation and infections, verbal assault and even physical violence and the imminent threat of malpractice litigation are not anticipated by most young colleagues at the outset of their medical career [1–3]. Increasing red tape goes along with limited decision-making authority and pressure to perform to exacting standards. There is a subtle transition from burden to overwork. Anxiety, major depressive disorders, substance abuse, burnout, divorce and suicide all occur more frequently amongst physicians than in the general working population [4–9]. Fatigue in anaesthesiology trainees, was reported to have effects on physical health, psychological well-being and personal relationships [10]. Health status matters; it can seriously impair a person’s performance and limit the achievable quality of life. Furthermore, an unhealthy job environment and inadequate working conditions may contribute to work stress and impair patients’ safety [11]. It goes without saying that a health system should neither make the patient more ill nor should it diminish the health of the healthcare provider. Focusing on a vulnerable group of hospital employees [4,9,12,13], we drew up and distributed to Austrian anaesthesiologists a questionnaire on working conditions, occupational stress and perceived health status.

## Materials and methods

### Study design and setting

A nationwide investigation using a self-reporting questionnaire was conducted from July to August 2013 and focused on physical and mental health, workload, and relationship with senior employees. Responders were identified as members of the Austrian Society of Anaesthesiology, Resuscitation and Intensive Care Medicine (ÖGARI). This anonymized inquiry was approved at the Society of Anaesthesiology, Resuscitation and Intensive Care Medicine (ÖGARI) board meeting on 24th April 2013. Ethics approval for a survey in healthy adult volunteers was not required according to the committee's officers of the institutional ethics committee. Scientific approval for the study (PM 01/100-004.-035.) was provided by the Innsbruck Medical Univeristy Hospital Trust on 31st May 2013 and by the Deputy Rector of the Medical University of Innsbruck on 7th June 2013. Inclusion criteria were anaesthesiologists working in Austrian hospitals, ÖGARI membership, who gave informed consent on the understanding that results will be published in a peer-reviewed journal. Exclusion criteria were incomplete data and lack of informed consent.

### Participants

All ÖGARI members (n = 1,149) were informed by E-mail about the investigation and invited to participate during a four-week period. Prior to study commencement, all members were contacted again by E-mail and instructed how to access the online questionnaire. Prior to the questionnaire participants were informed about the objectives of the survey and that results will be published in a scientific journal. We emphasized that data are collected anonymously and that it is not our intention to compare the situation among the various hospitals. Online agreement to participate was the precondition for accessing the questionnaire details. Participants who completed the questionnaire were offered the chance to win one of three Amazon vouchers worth 100 Euros. Each participant was registered with a number consisting of a consecutive count. Three winning numbers were randomly selected from among the participants who had completed the whole questionnaire. A reminder was sent out two weeks after the questionnaire went online. We were not able to contact non-respondents. Participants were asked to choose the answer that applied best to the situation in the last twelve months. They were informed that there are no ‘correct’ or ‘wrong’ answers. Participants were requested to not select answers they thought would comply with the society’s expectations. Study design, data collection and manuscript presentation are in accordance with the Helsinki Ethical Principles for Medical Research Involving Human Subjects [14] and follow the STROBE checklist for cross-sectional studies [15]. Data from this study have not been previously peer reviewed and published in any peer-reviewed journal. No part of the study is held under copyright due to previous publication. Some of data were previously printed in German in the master’s thesis written by Peter Paal, MD [16]. In addition, Peter Paal, MD and Prof. Heinz K. Stahl wrote an essay based on this questionnaire in German [17]. These works do not constitute dual publication as 1) the master’s thesis written by Peter Paal, MD is accessible only in four hard copies, kept at the institutional library and 2) the number of results displayed in the essay does not exceed the information displayed in an abstract.

### Study objectives and key elements

The primary study objective was to describe work-life balance in anaesthesiologists employed in hospitals. Secondary study objectives included workload, physical health, emotional well-being, job satisfaction and working conditions under increased pressure from consolidated working hours. The questionnaire comprised three sections:

#### Assessment of socio-demographic factors

Socio-demographic factors included gender, age, family situation, qualification, position, and terms of employment.

#### Assessment of occupational stress and workers’ dependence on the working area

Working conditions were investigated with the Instrument for Stress-Oriented Task Analysis (ISTA, Version 6.1) [18]. The ISTA items are arranged in various scores and focus on the assessment of regulation requirements, regulation possibilities and regulation problems when evaluating stress at work. This differentiation is particularly helpful in the analysis of jobs with high complexity of decisions, but diminished control and limited possibility to decide. There are five items each on task complexity, task conduct (manner of work accomplishment, the way in which tasks were completed), task variability, time tolerance (authority in time management and control over operating speed), uncertainty, task-related regulation problems, work interruptions, concentration demands, and time pressure. There was one item on task-related stressors, three items on communication possibilities (potential to communicate with others), seven items on participation (decision-making power in joint consultation and teamwork) and 13 items on cooperation latitude (extent of decision-making power in joint operation). Work latitude was defined as extent of decision-making power in operational procedure.

#### Assessment of physical health and emotional well-being

Physical well-being (17 items) and emotional well-being (21 items) were measured using relevant variables of the German version of the ‘Health and Stress Profile’ [19]. Regarding job satisfaction we evaluated: ‘Pay for work’, ‘Prospect for promotion’ and ‘Time organization’. The questionnaire was arranged as a single battery of questions to be completed on-line (www.2ask.de. Amundis Communications GmbH, Felix-Wankel-Str. 4, Konstanz, Germany). Psychometric data were collected using a Likert-type scale with five response levels (1 = very rarely, 2 = rarely, 3 = sometimes, 4 = often, 5 = very often) [20]. In this study we evaluated the combined response levels ‘often’ and ‘very often’.

### Statistical analysis

Demographic data are presented as frequencies and percentages. Dependent variables included emotional well-being and physical health, time organization, working conditions, occupational stress and dependencies; independent variables were demographic characteristics comprising age, gender, family situation, qualifications, position and working conditions. Nominal variables were analysed with the Pearson chi square test. Binomial proportion confidence intervals were calculated for occupational stress, job dependence, physical health and emotional well-being using the Clopper-Pearson exact method based on beta distribution. Ordinal variables were analysed with the Mann-Whitney U test (n = 2) and the Kruskal-Wallis test (n>2). Metric data were tested for normal distribution and groups were compared in an Analysis of Variance. In order to eliminate known confounding by gender, qualification, leading position and age group we evaluated the subgroups male/female, specialist/anaesthesiology trainee, leading/non-leading and age groups <30, 31–40. 41–50, 51–60 and >60 years. Statistics for each analysis were based on cases with no missing data for any variable in the analysis. Results were defined as significant when the P value was ≤0.05.

## Results

Of the 1,149 ÖGARI members, 1,145 anaesthesiologists working in hospitals were potentially eligible to participate, of whom 470 responded (response rate 41.0%); 394 anaesthesiologists (280 specialists, 114 anaesthesiology trainees) answered the online questionnaire (inclusion rate 34.4%), 76 participants did not complete the entire test battery and were not included in this analysis. Median age and gender distribution of participants were comparable to spread of ages and gender distribution of anaesthesiologists in Austria.

### Socio-demographic factors

Socio-demographic data were displayed in Table 1. Gender correlated with task complexity (P = 0.025). Female anaesthesiologists reported more frequently receiving instructions from superiors and co-workers (P < 0.001); this finding was even more pronounced in young female anaesthesiology trainees (< 30 years of age). Age correlated with task complexity (P <0.001) and task variability (P <0.002) as well as decision-making power (P = 0.005) and cooperation latitude (P <0.001). Family situation correlated with task complexity (P = 0.002) and task variability (P = 0.003), routine workload (P = 0.039) and working place equipment (P <0.001). Educational level correlated with task complexity (P <0.001), task variability (P = 0.003), and cooperation latitude (P <0.001). Anaesthesiologists in leading positions reported greater job satisfaction, task complexity, task variability, and time tolerance (all P <0.001).

**Table 1:** Socio-demographic factors. Gender, age, family situation, qualifications and mode of employment in 394 anaesthesiologists.

	N	%
Male/female	206/188	52.3/47.7
Age: <30 years	10	2.5
30–50 years	258	65.5
>50 years	126	32.0
Single, no children	57	14.5
Single with children	28	7.1
With partner, no children	99	25.1
With partner and children	209	53.1
Specialists/trainees	280/114	71.1/28.9
Leading position	129	32.7
Unlimited contract	258	65.5
Work experience >10 years	216	54.8
Annual general anaesthetics/hospital <5,000	83	21.1
>10,000	156	39.6
Alternating day/night shift-work	285	72.3

### Occupational stress and workers’ dependence on the working area

Results showed that anaesthesiologists suffer from three different types of occupational stress. The first type was occupational stress due to time pressure, uncertainty about work requirements and progress as well as complexity of work tasks. The other two sources of occupational stress derived from organisation of the workplace and cooperation needed for teamwork (Table 2). With regard to occupational stress, participants reported frequently working under huge time pressure (70.3%) as well as working intensively after a long period of stand-by (68.3%) and other periods with a heavy workload and the need to concentrate (85.0%). More than half of the participants suffered from working at high speed, work interruptions by colleagues, no or delayed work breaks, sometimes not even long enough for food intake, and stress caused by complicated decisions and different tasks, all to be executed at the same time. Occupational stress also affected anaesthesiologists’ private life: approximately half of the participants suffered from overtime work and reported not having enough time for themselves and for family members (Table 2).

**Table 2:** Time management. Perceived occupational stress in 394 anaesthesiologists during the previous 12 months.

	N	%	95%CI
Periods requiring intense concentration	335	85.0	81.2–88.2
Time pressure	277	70.3	65.6–74.6
Work intensively after a long period of stand-by	269	68.3	63.5–72.7
Time for communication with colleagues	260	66.0	61.2–70.5
Complicated decisions during work	250	63.5	58.6–68.1
High work speed	246	62.4	57.6–67.1
No or delayed breaks	234	59.4	54.5–64.1
Work interruptions by colleagues	230	58.4	53.5–63.1
Careful planning prior to performance	224	56.9	52.0–61.7
Perform different tasks simultaneously	209	53.1	48.1–58.0
Not enough time for oneself	207	52.5	47.6–57.4
Not enough time for one’s partner and children	102	25.9	21.8–30.4
Not enough time for sleep	199	50.5	45.6–55.4
Not enough time for food intake	127	32.2	27.8–37.0
Keep detailed information in mind	197	50.0	45.1–55.0
Overtime work	187	47.5	42.6–52.4
Routine work	118	30.0	25.6–34.7
Work interruptions by superior	78	19.8	16.2–24.0
Work with unclear instructions	64	16.2	12.9–20.2

One-quarter of the participants suffered from limited control over work latitude and task selection and also from difficulties locating information, material or equipment in order to continue working (Table 3). More than half of the anaesthesiologists reported having a maximum of 10 minutes per day for a break away from their workplace. Anaesthesiologists were strongly affected by the quality (56.7%) and speed (48.7%) of their colleagues’ work. Cooperation latitude was greater in anaesthesiologists holding a leading position than in those in a subordinate position (P = 0.003). Of the anaesthesiologists 5.3% were very fearful they could lose their job in the next six months, while at the same time 57.9% stated that they would easily find new employment, if necessary. Of the whole sample 51.0% stated that they are not paid well for their work.

**Table 3:** Job dependence. Quality of work environment and interaction with colleagues in 394 anaesthesiologists during the previous 12 months.

	N	%	95%CI
Work with high-quality equipment	269	68.3	36.5–72.7
Working place with space to move freely	228	57.9	52.9–26.6
Different working tools	224	56.9	52.0–61.7
Allowed to make own decisions	208	52.8	47.9–57.7
Control over all or most work outcomes	225	57.1	52.2–61.9
Control over work speed	119	30.2	25.9–34.9
Decision-making power in operational procedure	92	23.4	19.4–27.8
Delay in organizing information, equipment, tools	109	27.7	23.5–32.3
Influence on sequence of task performance	172	43.7	38.8–48.6
Influence over planning of working hours	63	16.0	12.7–19.9
Influence over planning of breaks	54	13.7	10.7–17.5
Influence over organization of the work area	45	11.4	8.7–14.9
Decision-making power in task selection	101	25.6	21.6–30.2
Decision-making power in co-worker selection	37	9.4	6.9–12.7
Physical performance variability during work	22	5.6	3.7–8.3
Cooperation despite interpersonal conflicts	266	67.5	62.7–72.0
Dependence on colleagues’ work quality	222	56.4	51.4–61.2
Dependence on exact knowledge of work status of others	194	49.2	44.3–54.2
Dependence on colleagues’ work speed	192	48.7	43.8–53.7
Dependence on joint planning of working steps	175	44.4	39.6–49.4
Management of complications caused by a colleague	174	44.2	39.3–49.1
Decision-making power when working alone or with others	89	22.6	18.7–27.0

### Physical health and emotional well-being

Of the participants 10.2% reported having taken sick leave of more than 15 days during the previous 12 months, while 64.0% reported having worked despite being ill. Most common physical complaints were muscle tension (46.2%), backache (33.0%), sleeping disorder (30.5%) and weakness (25.6%) (Table 4).

**Table 4:** Physical health. Frequency of physical complaints in 394 anaesthesiologists during the previous 12 months.

	N	%	95%CI
Muscle tension	182	46.2	41.3–51.1
Backache	130	33.0	28.4–38.0
Sleeping disorder	120	30.5	26.1–35.2
Weakness	101	25.6	21.6–30.2
Diminished sexual desire	79	20.1	16.4–24.3
Stomach ache	62	15.7	12.5–19.7
Fatigue	58	14.7	11.6–18.6
Headache	55	14.0	11.0–17.7
Memory lapses	50	12.7	9.8–16.3
Constipation	25	6.3	4.3–9.2
Palpitations	17	4.3	2.7–6.8
Loss of appetite	10	2.5	1.4–4.6

Of the study participants only 35.1% and 18.5% felt that anaesthesiologists have high reputations among colleagues in other specialities and the general population, whereas 29.2% and 46.9% felt that anaesthesiologists have poor reputations among colleagues in other specialities and the general population, respectively (Table 5). Low control over operating speed (Time tolerance) correlated with anaesthesiologists’ perceived poor reputation in the eyes of other specialities (P <0.001). Frequent feelings of being unsettled were reported by 38.1% of the respondents, while 31.7% stated that they often had difficulty talking about their emotions and 23.1% of participants confessed that they found it difficult to settle down after being irritated. Frequent dissatisfaction with life was reported by 11.4% of the respondents (Table 5).

**Table 5:** Emotional well-being. Perceived professional reputation and frequency of psychosomatic complaints and emotional stress in 394 anaesthesiologists during the previous 12 months.

	N	%	95%CI
High reputation among colleagues in other specialities	139	35.1	30.6–40.2
High reputation in the general population	73	18.5	14.8–22.7
Poor reputation among colleagues in other specialities	115	29.2	24.7–33.9
Poor reputation in the general population	185	46.9	41.9–52.0
Strive even harder	254	64.5	59.6–69.0
Hide emotions	185	47.0	42.1–51.9
Feel unsettled	150	38.1	33.4–43.0
Difficulty talking about emotions	125	31.7	27.3–36.5
Bad mood	117	29.7	25.4–34.4
Difficulty settling down after irritation	91	23.1	19.2–27.5
Feel nervous	82	20.8	17.1–25.1
Feel pessimistic	72	18.3	15.0–22.4
Feel depressed	58	14.7	11.6–18.6
Displeasure with life	45	11.4	8.7–14.9
Feel sad	40	10.2	7.5–13.5

## Discussion

The results of our investigation reveal that the current working conditions of anaesthesiologists in Austria include several problematic aspects of occupational stress. Apparently, pressure to increase performance is combined with increased time pressure and reduced recovery time during work. Furthermore, there is insufficient time to unwind and recover from work or to catch up on sleep. Long working hours with consequently less time for oneself, one’s partner and children were reported by half of the participants. Breaks during work are often irregular and short, if any, sometimes not even long enough to eat something. One-third of the participants reported suffering from sleep disorders. Sleep disorders impair alertness and may affect quality of patient care [21]. Half of the study anaesthesiologists reported that they frequently have to recall detailed information during their work. However, frequent lapses of memory were reported by 12.7% of the participants. There is the potential for poorer situational awareness when cognitive processes such as planning, concentration abilities and memory become impaired. It has been shown that even 12-hour shifts are exhausting [22]. Furthermore, after having worked two 12-hour shifts, at least three days off are needed to recover from the long working hours [22]. The current practice of 23 hours of rest after 25 duty hours in acute patient care cannot be enough to sufficiently recover. Although the binding regulations of the European Working Time Directive limit daily working time to 11 hours and weekly working time to 48 hours [23], physicians still work up to 72 hours a week on a “voluntary basis” in Austria.

Emotional problems such as bad mood, feeling restless and having difficulty talking about emotions were reported by one-third of the participants. Emotional dissonance from an inability to express emotions may indicate an imbalance between job demands and job resources such as support from superiors [24,25]. About two-thirds of anaesthesiologists reported having worked despite being ill during the last twelve months. Only half of the participants reported ‘feeling at home’ in their hospital, and only one-third felt their hospital strongly encourages them to perform to an excellent standard. About half of the responding anaesthesiologists mentioned insufficient pay, poor prospects for promotion, and poor time organization. Alarmingly, 11.4% of the responding anaesthesiologists reported frequent dissatisfaction with life. The balancing act between high-quality healthcare and business is crucial for good governance in healthcare management. However, when acquisition of money is prioritised over valuing of healthcare staff, achieving a work-life balance becomes difficult.

Variability of physical performance during work was given low scores by three-quarters of the anaesthesiologists participating in our study. Work demands are complex and there is a large range of equipment to work with, mostly of high quality. Two-thirds of the study participants reported frequently being under time pressure and working at high speed. Performing different tasks simultaneously and experiencing frequent interruptions are common. Having to work intensively after a long period of stand-by is commonplace. Decision-making power in task selection was rated low by three-quarters of the study anaesthesiologists. Dependence on colleagues from other specialities is high. More than half of the study anaesthesiologists perceived their reputation as poor in the eyes of colleagues in other specialities and the general public. From our results we cannot tell the extent to which work alienation and personally perceived inappropriate tasks contributed to perceived stress and influenced participants’ image of their profession [26]. There is little opportunity to influence the planning of working hours and breaks. The combination of severe time pressure and little decision-making authority can trigger stress reactions and even burnout [27]. A paradoxical situation arises when healthcare organisations designed to improve patients’ health provide working conditions that can endanger the health of their employees. It is important to develop a novel score for stress perception in anaesthesiologists based on requirements (task complexity and task conduct), teamwork (participation and cooperation latitude), and time management (time tolerance and time pressure) related to individual personality profiles (interpersonal skills, emotionality, neuroticism, open mindedness, detachment) [28]. Scores would then have to be tested for validity and reliability before being applied in a follow-up study.

In our study age correlated with the opportunity to perform more complex and more varied tasks, as well as the power to make decisions in operational procedure. The lifespan perspective of work design and ageing at work includes modification of job demands in relation to age [29]. In particular, 24-hour duties exhaust older colleagues more than younger ones. Furthermore, the interval required for recovery becomes longer with increasing age. Guaranteed breaks of a reasonable duration and reduced time and performance pressure for older colleagues are legal requirements. This is in definite conflict with increased pressure from consolidated working hours. The employer is responsible for safeguarding his employees’ health as one aspect of his fiduciary duty. Ideally, work should be part of a satisfying life and not diametrically opposed to it. We would speculate that the provision of family-friendly working hours, improved work-life balance, and reduction of latent risk factors [30] could bring long-term benefits, especially when searching for new employees in a tight European market. Changes in the work schedule of anaesthesiologists are required to avoid negative effects on health and emotional well-being. In particular, scheduled overtime work must be abolished and the unconditional basic income has to be raised. The ongoing and persistent concentration of working time has to stop. The existing strict employee protection laws that define minimum safety and occupational standards for employers should be enforced without fail [23]. Breaks and off-time must be guaranteed. Management should create a work climate based on esteem for personnel rather than on replaceability of employees.

### Limitations

Although median age and gender distribution of participants are regarded as representative for Austria and the 34.4% inclusion rate of our investigation is within the range of similar studies [31], we cannot exclude bias. The response rate in this convenience sample is low and we are not able to comment on the characteristics of the non-responders. People who are burnt out tend to not participate and may thus be less likely to have responded. Presumably, the numerous items and a completion time of approximately one hour are further reasons for non-participation. Furthermore, subjective assessment in self-reporting measures might reveal anticipated responses and not the real situation. External validity is limited as work expectations of the younger generation of anaesthesiologists might differ from previously published expectations [32]. To date, there is even no comprehensive definition of health. Health means more than the mere absence of disease and more than functional and metabolic efficiency. Neither is health a rigid constant, equally valid for everyone, nor is disease simply a deviation from normal distribution. Health perceptions differ according to age and cultural background and cannot be considered independently of a personal sense of vitality. A variety of psychosocial dimensions were not included in our investigation. We did not explore the frequency of work-related rumination. We asked if there is enough time for partners and children, but we did not enquire about the prevalence of work-family conflicts resulting from effects of work-to-family spill-over and from job demands that interfere with the family domain. We did not ask about the frequency of divorce in anaesthesiologists, while knowing that the divorce rate in physicians is high in Austria. No subjective assessment of inappropriate tasks and their potential contribution to perceived stress was made. We asked about emotional exhaustion, but did not assess the two other burnout dimensions, namely depersonalization and personal accomplishment. We did not focus on coping strategies and we did not intend to evaluate substance use. No questions broached the subject of suicidal ideation, but it is known that the suicide rate in anaesthesiologists was reported to be up to 6.8-fold higher than in internal medicine specialists [33].

## Conclusions

Strong time pressure and little decision-making authority during work along with long working hours and frequent work interruptions constitute the basis for occupational stress in anaesthesiologists. We conclude that increased pressure to perform during work hours contributes to emotional exhaustion and poor work-life balance. Changes in the work schedule of anaesthesiologists are required to avoid negative effects on health and emotional well-being.

## Supporting information

S1 Table Master’s thesis by Peter Paal, MD written and printed in German. (PDF)

S1 Fig Brief essay by Peter Paal, MD and Prof. Heinz K. Stahl in German based on the questionnaire used in the Master’s thesis. (PDF)
