# Supplementary material for: Consolidation of working hours and work-life balance in anaesthesiologists – A cross-sectional national survey
Source: PLoS One. 2018 Oct 31;13(10):e0206050. doi: 10.1371/journal.pone.0206050 (PMC6209218; doi:10.1371/journal.pone.0206050)
Supplement: S1 Table — (PDF) [file pone.0206050.s001.pdf]

MCI MANAGEMENT CENTER INNSBRUCK

GENERAL MANAGEMENT

EXECUTIVE MBA

MASTER´S THESIS

„Arbeitsbedingungen von AnästhesistInnen in Österreich“

EINGEREICHT BEI: PROF. DR. HEINZ K. STAHL

EINGEREICHT VON: PD DR. PETER PAAL, DESA, EDIC

Danksagung

Für die Inspiration zu und das Gelingen dieser Masterarbeit möchte ich Prof. Heinz K. Stahl außerordentlich danken. Mit seinem Scharfsinn und seinem Weitblick hat er mein Reflektieren stimuliert und zu einem fruchtbaren wissenschaftlichen Diskurs eingeladen. Arbeitszufriedenheit, Leistungsmotivation und Commitment in Organisationen sowie balancierte und postklassische Führung sind für mich keine Worthülsen mehr sondern sind nun Teil meines Grundverständnisses von Mitarbeiterführung. Herzlichen Dank!

Danken möchte ich auch dem Team des Management Centers Innsbruck, allen voran Prof. Kurt Matzler, Mag. Susanne Herzog und den Referenten, aber auch Frau Claudia Haidacher und Frau Victoria Abenthung die mit Ihrem Engagement, Ihrer Kompetenz und Freundlichkeit diesen General Management Executive MBA Lehrgang zu einem wahren „Aha“- Erlebnis gemacht haben und mir eine reichhaltige Schatzkiste an Führungswissen in meine geistigen Hände gereicht haben.

Einen großen Dank für die tolle Kooperation möchte ich Prof. Wolfgang Lederer aussprechen. Er hat mich neben Prof. Stahl auf das Thema dieser Arbeit gebracht und zur Diskussion beigetragen. Dankend erwähnen möchte ich noch für die angenehme und professionelle Zusammenarbeit Prof. Johann Kinzl, Ass. Prof. Christian Traweger, Mag. Alice Sanwald, Dr. Daniel Pehböck, Dr. Thomas Mitterlechner und Dr. Daniel von Langen.

Weiters möchte ich meiner lieben Frau Dr. Evelyn Baumgartner für die immer liebevolle Unterstützung danken. In vielen glücklich miteinander verbrachten Jahren haben wir sehr viele Höhen genossen aber auch einige dunkle Täler durchschritten. Vielen Dank für alles, liebe Evelyn! Danke möchte ich unseren lieben Söhnen Jakob, Tobias und Leo, dass sie da sind und unser Leben auf eine einmalige charmante Weise immens bereichern. Zusammen mit Evelyn geben Sie meinem Leben einen enormen Auftrieb und die essentielle Nestwärme.

An nächster, aber nicht minder wichtiger Stelle, möchte ich meinen Eltern danken, welche mich seit nunmehr fast 40 Jahren nach wie vor liebevoll umsorgen, wenn nun auch aus größerer Distanz aber unverminderter Aufmerksamkeit und Zuneigung. Danke, dass ihr mich von der Wiege weg auf meiner Wanderung mit viel Zuneigung und Vertrauen begleitet habt. Ein großer Dank gebührt auch meinen Geschwistern sowie ihren Partnern und meiner Schwägerin für viele schöne gemeinsam verbrachte Momente: Sabine und Hannes, Barbara und Michl, Michael und Bärbl und Verena. Ein abschließendes VIELEN DANK gilt allen Freunden und KollegInnen die mich bei dieser Arbeit (zum Beispiel Martin, Johanna, Hubsi, Julia) unterstützt haben und all jenen Freunden mit denen wir viele unvergessliche Momente verbracht haben und verbringen werden. ☺

Inhaltsverzeichnis

Danksagung 2

Inhaltsverzeichnis 3

Abbildungsverzeichnis 5

Tabellenverzeichnis 7

1. Beweggrund für diese Arbeit 8

2. Problemstellung und Abgrenzung 8

3. Zielsetzung der Arbeit 9

4. Begleitung und Aufbau der Arbeit 10

5. Methodik 10

6. Theoretischer Hintergrund 21

6.1. Arbeitszufriedenheit 21

6.2. Leistungsmotivation 25

6.3. Commitment 29

6.4. Exkurs 32

6.5. Begründung der Schleife Arbeitszufriedenheit-Leistungsmotivation-Commitment-Arbeitszufriedenheit 33

7. Die Studie 35

7.1. Ergebnisse 35

7.2. Demographie 35

7.3. Geistiges Wohlbefinden 36

7.4. Körperliches Wohlbefinden 38

7.5. Instrument der Stress Tätigkeits Analyse 48

7.6. Mitarbeiterbindung 80

7.7. Korrelationen 86

8. Diskussion 88

8.1. Arbeitsbedingungenen von AnästhesistInnen ausserhalb Österreich 88

8.2. Die Entwicklung der Arbeitsbedingungen der AnästhesistInnen 89

8.3. Kritische Bereiche bei den Arbeitsbedingungen der AnästhesistInnen in Österreich 92

9. Schlussfolgerungen aus der Beantwortung der forschungsleitenden Fragen 99

9.1. Anhand welcher Merkmale können die Arbeitsbedingungen der AnästhesistInnen in Österreich am zweckmäßigsten beschrieben werden? 99

9.1.1. Der Durchschnittsanästhesist 100

9.2. Welche vorläufigen Schlussfolgerungen für die Organisationsgestaltung im Krankenhausbereich können aus dieser Bestandsaufnahme abgeleitet werden wenn man dafür Kriterien wie Arbeitszufriedenheit, Leistungsmotivation und Commitment heranzieht? 101
9.2.1. Der Einfluss von Arbeitsmotivation und Führungsstil auf Leistungsmotivation und Commitment 101
9.2.2. Einfluss der Organisationsgestaltung auf Arbeitszufriedenheit, Leistungsmotivation und Commitment 104
9.2.3. Nachhaltige Organisationsgestaltung und Führung im Krankenhaus 108
9.2.4. Konkrete Empfehlungen für die Organisationsgestaltung im Krankenhausbereich unter spezieller Berücksichtigung der Schleife Arbeitszufriedenheit- Leistungsmotivation- Commitment- Arbeitszufriedenheit 113
10. Ausblick 114
11. Literaturverzeichnis..117
12. Eidesstattliche Erklärung 123

Abbildungsverzeichnis

Abbildung 1. Genehmigung zur Studiendurchführung von Seiten der Österreichischen Gesellschaft für Anästhesiologie, Reanimation und Intensivmedizin.

Abbildung 2. Genehmigung der Studiendurchführung durch die Medizinische Universität Innsbruck (MUI).

Abbildung 3. Genehmigung der Studiendurchführung von Seiten der Tiroler Landeskrankenanstalten (TILAK).

Abbildung 4. Persönliche Einladung zur Preisübergabe des Deutschen Freundes- und Förderkreises der Universitäten in Innsbruck e.V.

Abbildung 5. Vorinformation der Mitglieder der Österreichischen Gesellschaft für Anästhesiologie, Reanimation und Intensivmedizin über die bevor stehende Studie (ÖGARI) wenige Tage vor der Aussendung der Umfrage.

Abbildung 6. Die Arbeitszufriedenheit ergibt sich laut dem Equity Modell von JOHN STACEY ADAMS aus dem Vergleich von Input und Output (ADAMS, 1965, 267-299).

Abbildung 7. Eine valide Methode um Arbeitszufriedenheit zu klassifizieren und zu definieren ist die Einteilung nach Agnes Bruggemann (BRUGGEMANN et al., 1975).

Abbildung 8. Leistungsmotivation gründet sich immer auf impliziter Motivation. Explizite Motive übersetzt die Person entsprechend ihrem Wertesystem in innere Motivatoren und entscheidet dann aufgrund ihres inneren Programms (bestehend unter anderem aus Werte- und Motivationssystems) (STAHL, 2013a).

Abbildung 9. Flow bei der Arbeit durch eine richtige Mischung aus Leistungsfähigkeit und Herausforderung, adaptiert nach (CZIKSZENTMIHALYI, 2004).

Abbildung 10. Schematisches Modell für organisationales Commitment (STAHL, 2013a).

Abbildung 11. Die jungen Generationen (zum Beispiel Generation Y und Z) die nun auf den Arbeitsmarkt kommen sind mental anders „gestrickt“ als die älteren Generationen (STAHL, 2013c).

Abbildung 12. Besonders seit der Sozialrevolution im Jahr 1968 hat sich der Schwerpunkt weg von Pflicht- und Akzeptanzwerten und hin zu Selbstentfaltungswerten verschoben (STAHL, 2013c).

Abbildung 13. Darstellung der kausalen Feedbackschleife von Arbeitszufriedenheit-Leistungsmotivation- Commitment- Arbeitszufriedenheit. Eine Zunahme von Commitment verstärkt wiederum die Arbeitszufriedenheit, da der Kopf frei ist weil der Anästhesist weiß dass er am richtigen Arbeitsplatz angekommen ist. Er muss sich zum Beispiel nicht mehr mit Umzug, Arbeitsplatz- und Freundeswechsel auseinandersetzen. Die Menschen am aktuellen

Arbeitsplatz sind seine guten Freunde, fast schon Teil seiner Familie. Somit steigt durch das hohes Commitment auch die Arbeitszufriedenheit, die Schleife schliesst sich.

Abbildung 14. Die Qualität des Gesundheitssystem kann auf der einen Seite mit den Dimensionen Zugänglichkeit, hohe Qualität und Angebotsbreite und auf der anderen Seite mit Finanzierbarkeit beschrieben werden (SALFELD et al., 2009).

Abbildung 15. Das Chirurgie Team und der Anästhesist bei einem Eingriff im Operationssaal. Der Chirurg ist augenscheinlich im Zentrum des medizinischen Geschehens, der Anästhesist steht abseits des Operationsfeldes hinter dem Operationsvorhang (ABC, 2013).

Abbildung 16. Belastungskreislauf und Entlastungsmodell bei Burnout (HILLERT et al., 2007).

Abbildung 17. zeigt Gehälter von KrankenhausärtzInnen und Pflegekräften in verschiedenen Ländern. Österreichische ÄrztInnen werden ähnlich gezahlt wie ihre deutschen KollegInnen; die TILAK zahlt im österreichischen Schnitt vor allem bei Jungen deutlich weniger (SALFELD et al., 2009).

Abbildung 18. Produktivitätsunterschiede in verschiedenen Krankenhäusern unter Berücksichtigung der Fallschwere und der Dienstarten. Für Krankenhäusern mit mehr als 300 Beten) (SALFELD et al., 2009).

Abbildung 19. Produktivität des Personals in verschiedenen nationalen Krankenhaussystemen (SALFELD et al., 2009).

Abbildung 20. Ein Kontinuum des Empowerment (BUCHANAN et al., 2010, 385-416).

Abbildung 21. Das Magische Dreieck des Krankenhausmanagements (PADOSCH et al., 2011, 364-369)

Abbildung 22. Die „Vier Welten“ des Krankenhauses aus (MAYERHOFER, 2013, 326), adaptiert nach (GLOUBERMAN et al., 2001, 56-69).

Abbildung 23. Koalitionen zwischen den „Vier Welten“ des Krankenhauses aus (MAYERHOFER, 2013, 327), adaptiert nach (GLOUBERMAN et al., 2001, 56-69).

Abbildung 24. Balancierte Führung akzeptiert beide kritischen Dualitäten: Die Führungskraft entscheidet sich nicht für eine Seite der Führung (zum Beispiel *nur* Planen), sondern wählt das sowohl als auch entsprechend der Situation (zum Beispiel *sowohl* Planen *als auch* Improvisieren) (STAHL, 2013c).

Abbildung 25. Die Soziale Architektur in einem Unternehmen kann Sollen und Dürfen maximal unterstützen (STAHL, 2013c).

Abbildung 26. Die vier Freiheitsgrade Können, Wollen, Dürfen und Sollen aus (STAHL, 2013c).

Abbildung 27. Abmilderung der Hierarchie um die vier Freiheisgrade „Können, Wollen, Sollen, Dürfen“ eines Anästhesisten zu erhöhen (STAHL, 2013c).

Tabellenverzeichnis

Tabelle 1. Gebiete für möglichen Arbeitsstress, eingeteilt in Bereiche welche mittels Frageskalen abgefragt vom „Instrument der Stressbezogenen Arbeitsanalyse“, ISTA Version 6.1 (SEMMER et al., 2007).

Tabelle 2. Um jemanden zu motivieren muss man sein inneres Programm kennen. Menschenkenntnis und Individualisierung sind in der modernen Führung sehr wichtig. In der Summe sollte also klassische Führung einer postklassischen Führung weichen (STAHL, 2013a).

Tabelle 3. Hygiene and Motivationsfaktoren, adaptiert von (HERZBERG et al., 1959).

Tabelle 4. Präventionsmaßnahmen gegen Burnout, adaptiert aus (MICHALSEN et al., 2011, 31-38).

Tabelle 5. Idealtypische Strukturen der Führung, adaptiert von (STAHL, 2013c).

1. Beweggrund für diese Arbeit

Der Autor dieser Arbeit ist Oberarzt für Anästhesie und Intensivmedizin an der Universitätsklinik für Anästhesie und Intensivmedizin Innsbruck, Österreich. Im Rahmen seiner Tätigkeit stellte der Autor wiederholt fest dass inbesondere in großen Krankenhäusern eine herausragende medizinische Experten Kompetenz der ÄrztInnen vorhanden ist diese aber in der Regel nicht mit einer ähnlich großen Unternehmens- und Führungskultur gepaart ist. Damit entsteht häufig die paradoxe Situation dass zwar die Patienten in Krankenhäusern geheilt werden, die ÄrztInnen jedoch, die Patienten heilen, zunehmend weniger gesund leben und schlussendlich häufig auch aufgrund der Arbeitsbedingungen krank werden. Der Beweggrund für diese Arbeit war also, aus der Perspektive des Anästhesisten die Arbeitsbedingungen von AnästhesistInnen in Österreich zu analysieren, den Jetztzustand festzustellen und mögliche Verbesserungsvorschläge zu formulieren.

2. Problemstellung und Abgrenzung

Arbeitsbedingungen im Allgemeinen und Arbeitsintensität im Besonderen üben eine beachtliche Rolle auf die Arbeitszufriedenheit von AnästhesistInnen aus. Aufgrund eines zunehmenden Personalmangels, Zwängen der Produktivitätssteigerung und einer damit resultierenden Zunahme der Arbeitsintensität können Arbeitsüberlastung, abnehmende Lebensqualität und zunehmende Krankheitssymptome mit dem Endbild Burnout resultieren.

Der Begriff Burnout wurde 1974 in Zusammenhang mit psychischen Erschöpfungszuständen geprägt (FREUDENBERGER, 1974, 159-165). Burnout mit den typischen Merkmalen emotionale Erschöpfung, abgebrühter oder gar zynischer Umgang mit anderen und subjektiver Leistungsminderung ist seit mehreren Jahren sowohl in der Wissenschaft als auch in der Populärliteratur zu einem gängigen Begriff geworden (BAUER et al., 2003, 213-222). Interessanterweise entspricht der subjektive Symptomkomplex Burnout nicht den wissenschaftlichen Kriterien von Objektivität (Unabhängigkeit des Messergebnisses vom Beobachter), Reliabilität (zuverlässige Reproduzierbarkeit des Messergebnisses) und Validität (zuverlässige Gültigkeit des Messergebnisses). Deshalb hat die Diagnose Burnout bisher keine Aufnahme als eigenständige Krankheitsentität in medizinischen Diagnosewerken wie zum Beispiel dem Diagnostic and Statistical Manual of Mental Disorders (DSM IV) oder dem ICD-10 gefunden (MICHALSEN et al., 2011, 23-30). Inzwischen wurden verschiedene Modelle entwickelt, um Burnout besser zu erfassen. Aus diesen Modellen hervorzuheben ist das „Effort-reward-

modell“, mit dem chronische berufsbedingte Erschöpfungszustände als ein Ungleichgewicht zwischen Leistung (Effort) und Belohnung (Reward) beschrieben werden (Effort-reward-imbalance) (BAKKER et al., 2000, 884-891).

Arbeitsbedingungen haben einen wesentlichen Einfluss auf die psychische und körperliche Gesundheit. Zum Beispiel zeigen Untersuchungen dass jeder dritte Deutsche unter Dauerdruck steht und sich in Deutschland die Anzahl der Fehltage aufgrund von psychischer Krankheit an allen Krankheitstagen innerhalb von zwei Dekaden von 3,7% auf 10,8% erhöht und damit fast verdreifacht hat. Zudem dauern die Krankenstände welche durch psychische Krankheit verursacht werden mit 22,5 Abwesenheitstagen deutlich länger als bei den meisten anderen Erkrankungen (BADURA et al., 2010).

Eine Vielzahl von Studien hat sich in den letzten Jahren mit der Untersuchung von Burnout bei Ärzten und Pflegekräften befasst. Eine Grundannahme vieler Studien war, dass gerade diese Berufsgruppen aufgrund ihrer psychisch belastenden Arbeit und häufigen Arbeitszeiten außerhalb der üblichen Kernarbeitszeit (Montag bis Freitag 8-16 Uhr) auffällig häufiger chronisch überlastet sind. Als besonders gefährdet wurden in der Medizin die Bereiche Anästhesie, Intensivmedizin, Notfallmedizin, Onkologie und Psychiatrie eingestuft (KINZL et al., 2006, 2461-2464; EMBRIACO et al., 2007, 686-692; BRAUN et al., 2008, 800-804; BUDDEBERG-FISCHER et al., 2008, 31-38; NYSSEN et al., 2008, 406-411; VERDON et al., 2008, 152-156; CHIRON et al., 2010, 948-958). AnästhesistInnen erscheinen besonders Burnout gefährdet, da sie in drei der oben genannten Risikobereiche (Anästhesie, Notfallmedizin und Intensivmedizin) arbeiten. Einige Studien haben bereits die Arbeitsbedingungen von Anästhesisten in einzelnen Krankenhäusern oder auch auf nationaler Ebene analysiert (LINZER et al., 2002, 191-193; NYSSEN et al., 2003, 333-337; LEDERER et al., 2006, 58-63; MORAIS et al., 2006, 433-439; EMBRIACO et al., 2007, 686-692; KINZL et al., 2007, 334-338; CHIRON et al., 2010, 948-958). Für Österreich liegen bisher noch keine Untersuchungen vor.

3. Zielsetzung der Arbeit

Das Ziel der vorliegenden Arbeit besteht darin, erstmals die Arbeitsbedingungen von AnästhesistInnen in Österreich zu untersuchen. Im Sinne der Philosophie des Pragmatismus und der handlungswissenschaftlichen Perspektive der Arbeit sollten fünf konkrete Handlungsempfehlungen zur Verbesserung der Arbeitssituation der AnästhesistInnen in Österreich ausgearbeitet werden. Dieser Arbeit liegen zwei forschungsleitende Fragen zu Grunde:

1. Anhand welcher Merkmale können die Arbeitsbedingungen der AnästhesistInnen in

Österreich am zweckmäßigsten beschrieben werden?

2. Welche vorläufigen Schlussfolgerungen für die Organisationsgestaltung im Krankenhausbereich können aus dieser Bestandsaufnahme abgeleitet werden wenn man dafür Kriterien wie Leistungsmotivation, Arbeitszufriedenheit und Commitment heranzieht?

4. Begleitung und Aufbau der Arbeit

Der Supervisor dieser Arbeit war HEINZ K. STAHL (Interdisciplinary Department for Management and Organisational Behaviour, Wirtschaftsuniversität Wien; Koleiter der Lehrgänge „General Management“ und „Personalmanagement“, Management Center Innsbruck MCI). Der Fragebogen wurde entwickelt in Kooperation mit WOLFGANG LEDERER (Univ. Klinik für Anästhesie und Intensivmedizin Innsbruck, Medizinische Universität Innsbruck) und JOHANN KINZL (Univ. Klink für Psychosomatik und Psychiatrie, Medizinische Universität Innsbruck). Der aktuelle Fragebogen war die logische Konsequenz einiger Vorarbeiten einiger Mitarbeiter dieser Arbeit (KINZL et al., 2005, 211-215; KINZL et al., 2006, 2461-2464; LEDERER et al., 2006, 58-63; KINZL et al., 2007, 334-338; LEDERER et al., 2008, 208-213). Der aktuelle Fragebogen wurde erstellt und adaptiert auf Basis der erwähnten vorangegangenen Studien und Empfehlungen aus der einschlägigen Literatur (KELLEY et al., 2003, 261-266; BOYNTON, 2004, 1372-1375; BOYNTON et al., 2004, 1312-1315; BURNS et al., 2008, 245-252). Im Fragebogen wurden im Speziellen die Bereiche psychische und körperliche Gesundheit, Arbeitsbelastung und Bindung an den Arbeitgeber analysiert.

5. Methodik

Diese Arbeit beruht in ihrem empirischen Teil auf einem anonymisierten Fragebogen, der Österreich weit an alle AnästhesistInnen, welche bei der Österreichischen Gesellschaft für Anästhesiologie, Reanimation und Intensivmedizin (ÖGARI) als Mitglieder gemeldet waren, ausgeschickt wurde. Es wurde ein *quantitatives* Umfrageverfahren gewählt da es für die vorliegende Fragestellung mehrere Vorteile gegenüber einem qualitativen Verfahren bot:

- Die Ergebnisse sind exakt quantifizierbar.
- Statistische Zusammenhänge können berechnet werden.
- Es besteht die Möglichkeit, eine große Stichprobe zu untersuchen und damit ein repräsentatives Ergebnis zu erzielen.

- Kosten- und Zeitaufwand stehen in einem günstigeren Verhältnis zueinander als bei qualitativen Verfahren.
- Objektivität, Reliabilität und Validität sind höher als bei einem qualitativen Verfahren.
- Es können Korrelationen berechnet werden, um einen klareren Zusammenhang zwischen den Daten (im vorliegenden Fall zum Beispiel Demographie, Geschlecht, Ausbildungsstand, Anstellungsverhältnis und Arbeitsbelastung) zu erhalten.

ALICE SAMWALD (Institut für Finanzwissenschaft der Universität Innsbruck) steuerte den Fragebogen über die Mitarbeiterbindung bei. THOMAS MITTERLECHNER und DANIEL VON LANGEN redigierten den Fragebogen in einer Weise dass DANIEL PEHBÖCK (alle drei Univ. Klinik für Anästhesie und Intensivmedizin Innsbruck, Medizinische Universität Innsbruck) den Fragebogen in eine Online Version übertragen konnte. Schlussendlich wurden die Daten mit Hilfe von CHRISTIAN TRAWEGER (Universität Innsbruck) bei simultaner Diskussion mit JOHANN KINZL und dem Autor statistisch ausgewertet. Die Ergebnisse der vorliegenden Arbeit wurden mit HEINZ K. STAHL diskutiert. Schlussendlich wurde die vorliegende Arbeit im Licht der forschungsleitenden Fragen in enger Abstimmung mit HEINZ K. STAHL erstellt.

Die Österreichische Gesellschaft für Anästhesie, Wiederbelebung und Intensivmedizin (ÖGARI, 2013) genehmigte die Durchführung der Studie und erlaubte Zugriff auf die Emailadressen der bei ihr eingeschriebenen AnästhesistInnen (Abbildung 1).

Oegari Office 24. April 2013 10:00
An: Peter Paal
WG: Masterarbeit "Working Conditions of Anaesthesiologists in Austria", Anfrage um Unterstützung durch die ÖGARI

Sehr geehrter Herr Dozent Dr. Paal,

in den Vorstandssitzung wurde beschlossen, dass wir den Fragebogen geren allen ÖGARI Mitgliedern zukommen lassen.

Es ist jedoch die Bitte ausgesprochen worden, diesen auf Deutsch zu versenden. Wir bitten Sie uns auch den kompletten Fragebogen zur Verfügung zu stellen. Wenn wir den deutschen Fragebogen von Ihnen erhalten, werden wir diesen gerne aussenden. Wir bitten Sie auch einen kurzen Begleittext zu verfassen und ein paar Tage bis zur Freigabe durch den Vorstand einzuplanen.

Mit freundlichen Grüßen/with best regards
Eva Gottfried
ÖGARI
Österreichische Gesellschaft für Anästhesiologie,
Reanimation und Intensivmedizin
Höfergasse 1A/Stg.1/DG/Top 15
1090 Wien,
Tel.: +43.1.406 48 10
Fax: +43.1.409 55 95
e-mail: office@oegari.at
www.oegari.at
ZVR: 050001094

Von: Peter Paal [mailto:peter.paal@chello.at]
Gesendet: Freitag, 12. April 2013 14:51
An: Illievich, Udo
Cc: Sibylle Kozek
Betreff: Masterarbeit "Working Conditions of Anaesthesiologists in Austria", Anfrage um Unterstützung durch die ÖGARI

Sehr geehrter Herr Prof. Illievich,

vielen Dank für das heutige Gespräch.

Anbei schicke ich Ihnen wie besprochen die Kurzbeschreibung der MBA Master Thesis und zusätzlich die standardisierten Fragen (ISTA, Kinzl BJA 2005 Supplementary data und den Fragebogen Zusatz zur Beurteilung der Bindung an den Arbeitgeber) die wir in Form einer Online Befragung stellen möchten. Bitte beachten Sie dass ich Ihnen den ISTA 5.0 Fragebogen (Kinzl BJA Supplementary data) beigelegt habe, da ich den neueren ISTA 6.0 Fragebogen noch nicht erhalten habe.

ISTA- Instrument zur stressbezogenen Tätigkeitsanalyse- ist ein Mittel, das in den 1980er Jahren zur Analyse gewerblicher Arbeitsplätze entwickelt wurde und es erfüllt die Gütekriterien der Reliabilität und Validität:
http://de.wikipedia.org/wiki/Instrument_zur_stressbezogenen_T%C3%A4tigkeitsanalyse

Ich lege Ihnen auch die Arbeit von Kinzl et al aus BJA 2005 bei, damit sie einen Eindruck haben wie die Arbeit aussehen könnte. Zudem finden Sie mein CV.

Ich danke Ihnen für Ihre Unterstützung und würde mich sehr freuen wenn das Präsidium zustimmt dass wir diese Umfrage unter den Mitgliedern der ÖGARI durchführen.

Sehr gerne kann die ÖGARI nach Publikation der Arbeit die Daten für eigene standespolitische Zwecke verwenden.

Für Fragen stehe ich Ihnen gerne zur Verfügung.

Beste Grüße aus Innsbruck

Peter Paal
MD, EDAIC, EDIC
Associate Professor
Department of Anesthesiology and Critical Care Medicine
University Hospital Innsbruck
Anichstr. 35, 6020 Innsbruck, Austria
Tel.:+43-512-504-80448
Fax:+43-512-504-22450
Telefax:+43-512-504 67-80448
http://www2.i-med.ac.at/anaesthesie/

Abbildung 1. Genehmigung zur Studiendurchführung von Seiten der Österreichischen Gesellschaft für Anästhesiologie, Reanimation und Intensivmedizin.

Durch die Kooperation mit der ÖGARI konnten über das Internet die Umfrage verschickt und die Ergebnisse gesammelt werden. Die Umfrage wurde in deutscher Sprache (Appendix 1) durchgeführt da sich Umfragen in der Muttersprache der Befragten am besten eignen wahrheitsgetreue Ergebnisse zu ermitteln. Die Umfrage wurde über eine Internet Plattform

durchgeführt die von der Firma 2ask (2ASK, 2013) entgeltlich zur Verfügung gestellt wurde. Die Umfrage wurde Anfange Juli an alle AnästhesistInnen per Email versandt, welche zum damaligen Zeitpunkt bei der ÖGARI als Mitglied registriert waren.

Die Umfrage bestand aus vier Teilen mit Fragen zu: 1. Demographie, 2. geistige und körperliche Gesundheit welche dem Gesundheit und Stress Fragebogen (GUS) von SCHNEEWIND und Mitarbeitern entnommen wurde (SCHNEEWIND et al., 1999), 3. Arbeitsbedingungen und Belastung am Arbeitsplatz entnommen aus dem **I**nstrument-zur-**S**tressbezogenen-**T**ätigkeits**A**nalyse (ISTA 6.1) von SEMMER und Mitarbeitern (SEMMER et al., 2007) und 4. Fragen zum Arbeitsvertrag und der Bindung an den Arbeitgeber. Für die Verwendung der Teile aus dem GUS und ISTA 6.1 wurde die schriftliche Einwilligung der Urheber eingeholt.

Da wir politische Zwietracht in Österreich durch Vergleiche von Krankenhäusern und Krankenhausträgern vermeiden und möglichst ehrliche Antworten erhalten wollten wurde die Umfrage anonymisiert durchgeführt. Aus diesem Grund wurden der Name und der Standort des Krankenhauses sowie des Krankenhausträgers bewusst nicht erfasst, obwohl die Auswertung sicherlich aufschlussreich gewesen wäre und wertvollen Diskussionsstoff geliefert hätte. Zur politischen Absicherung holte der Autor im Vorfeld schriftliche Einwilligungen von den beiden Krankenhausträgern der Universitätsklinik Innsbruck ein. Die schriftliche Einwilligung der Medizinischen Universität Innsbruck (MUI) vertreten durch Vize Rektor GÜNTHER SPERK (Abbildung 2) und

MEDIZINISCHE UNIVERSITÄT
INNSBRUCK

Vizerektorat für Forschung
Univ.-Prof. Dr. Günther Sperk
Peter-Mayr-Str. 1a
A-6020 Innsbruck
Austria

e-Mail	Telefon	Fax	Datum
guenther.sperk@i-med.ac.at	+43-512-9003-71210<br>+43-512-9003-71201	+43-512-9003-73200	7. Juni 2013

Hiermit bestätige ich dass seitens der Medizinischen Universität Innsbruck keine Bedenken gegen die Umfrage *"Working Conditions of Anaesthesiologists in Austria"* besteht welche PD Dr. Peter Paal, Univ. Klinik für Anästhesie und Intensivmedizin Innsbruck, im Rahmen seiner Masterarbeit für das Master of Business Administration (MBA) Studium am Management Center Innsbruck (MCI) durchführt.

Die Umfrage erfolgt wie im Projektantrag ausgeführt anonym, d.h. weder Teilnehmer noch Arbeitgeber oder Arbeitsstandorte können aus den Umfrageergebnissen nachvollzogen werden. Zudem nehme ich zur Kenntnis, dass sowohl der Krankenhausträger TILAK, durch Personaldirektor Dr. Schwab, als auch die Österreichische Gesellschaft für Anästhesiologie, Reanimation und Intensivmedizin die geplante Masterarbeit unterstützen.

Mit freundlichen Grüßen,

Univ. Prof. Dr. Günther Sperk
Vizerektor für Forschung und Lehre
Medizinische Universität Innsbruck

MEDIZINISCHE UNIVERSITÄT INNSBRUCK

Abbildung 2. Genehmigung der Studiendurchführung durch die Medizinische Universität Innsbruck (MUI).

der Tiroler Landeskrankenanstalten GmbH (TILAK) vertreten durch Personaldirektor MARKUS SCHWAB (Abbildung 3) liegen dieser Arbeit bei.

tilak
Unternehmen Gesundheit

Herrn
Priv.-Doz. Dr. Peter Paal
Univ.-Klinik für Anästhesie und
Allgemeine Intensivmedizin
im Hause

Personalmanagement

Mag. Dr. Markus Schwab
Personaldirektor
Prokurist

Datum 31. Mai 2013
Kontakt Mag. Dr. Markus Schwab
Telefon, Fax +43(0)50 504-28630 | -28669
E-Mail markus.schwab@tilak.at
GZ PM 01/100-004.-035.
Betreff **Bestätigung**

Sehr geehrte Damen und Herren,

das Projekt *"Working Conditions of Anaesthesiologists in Austria"* wurde von Herrn PD Dr. Peter Paal, Oberarzt an der Univ.-Klinik für Anästhesie und Allgemeine Intensivmedizin, a.ö. Landeskrankenhaus (Univ.-Kliniken) Innsbruck, ausführlich dargestellt und erläutert.

Hiermit bestätige ich, dass seitens der TILAK – Tiroler Landeskrankenanstalten GmbH gegen die geplante Umsetzung im Rahmen seines Masters of Business Administration (MBA) Lehrganges am Management Center Innsbruck (MCI), kein Einwand besteht.

Mag. Dr. Markus Schwab

Abbildung 3. Genehmigung der Studiendurchführung von Seiten der Tiroler Landeskrankenanstalten (TILAK).

Um die aktuelle Umfrage nicht zu zeitintensiv zu gestalten wurden nur Teile des GUS und des ISTA 6.1 Fragebogens verwendet. Der ISTA 6.1 Fragebogen umfasst Fragen zu Arbeitsplatz relevanten Bereichen, welche Stress und Überlastung beim Arbeitnehmer auslösen können (SEMMER et al., 2007) (Tabelle 1).

Tabelle 1. Gebiete für möglichen Arbeitsstress, eingeteilt in Bereiche welche mittels Frageskalen abgefragt vom „Instrument der Stressbezogenen Arbeitsanalyse“, ISTA Version 6.1 (SEMMER et al., 2007).

Soziodemographische Angaben:	SD1 - SD8
Qualifikationserfordernisse:	QU1 - QU3
Komplexität:	AK2, AK3, AK4, AK7, AK8
Handlungsspielraum:	HS1 – HS4, HS7
Partizipation:	PA1 - PA7
Variabilität:	VA1 - VA4, VA6
Zeitspielraum:	ZS1 ZS3 – ZS6
Unsicherheit:	UN4 – UN8
Unfallgefährdung:	UG1 - UG5
Arbeitsorganisatorische Probleme:	AOP1 – AOP4, AOP8
Einseitige Belastung	EBA1 - EBA6
Umgebungsbelastungen:	UGB1 - UGB17
Arbeitsunterbrechungen:	AUB1 – AUB4, AUB7
Konzentrationsanforderungen:	KON2 - KON6
Zeitdruck:	ZD1 – ZD4, ZD6
Kommunikationsmöglichkeiten:	KOM1 – KOM3
Kooperationsspielraum:	KSP3 - KSP5
Kooperationsenge:	KOP1 – KOP3, KOP7, KOP8
Kooperationserfordernisse:	KER1 -KER4

Beim ISTA 6.1 Fragebogen sind Fragen in Skalen (das heisst Fragegruppen, siehe Tabelle 1) zusammengefasst um das Rechnen von Korrelationen (zum Beispiel Geschlecht oder Ausbildungsstand mit Aufgaben Komplexität) zu rechnen. Wir limitierten die Skalen, die wir aus dem ISTA 6.1 Fragebogen entnahmen auf folgende Bereiche: Arbeitskomplexität (AK, Fragen 57-61 des aktuellen Fragebogens), Handlungsspielraum (HS, Fragen 62-66), Partizipation (PA, Fragen 69.1-69.7), Variabilität (VA, Fragen 67-68 und 70-72), Zeitspielraum (ZS, Fragen 73-77), Unsicherheit (UN, Fragen 78-82), arbeitsorganisatorische Probleme (AOP, Fragen 83-87), einseitige Belastung (EBA, Frage 88), Arbeitsunterbrechung (AUB, Fragen 89-93), Konzentrationsanforderungen (KON, 94-98), Zeitdruck (ZD, Fragen 99-103), Kommunikationsmöglichkeiten (KOM, Fragen 104-106), Kooperationsspielraum (KSP, Fragen 107-109), Kooperationsenge (KOP, 110-114) und Kooperationserfordernisse (KER, 115-119). Um die Studie finanzieren zu können bewarb sich der Autor bei zwei Forschungswettbewerben. Beim Wettbewerb des „Deutschen Freundeskreises der Universitäten in Innsbruck e.V.“ war der Autor erfolgreich und er erhielt im Juni 2013 für das beste Projekt des Wettbewerbes die Summe von 1000€ zugesprochen (Abbildung 4).

Deutscher Freundes- und Förderkreis
der Universitäten in Innsbruck e.V.

Das Generalsekretariat des Freundeskreises

Innsbruck, am 05. Juni 2013

Persönliche Einladung – Preisübergabe
des Deutschen Freundeskreises

Sehr geehrter Herr Dr. Paal,

der Vorstandsvorsitzende des Deutschen Freundeskreises der Universitäten in Innsbruck beglückwünscht Sie hiermit nochmals zur Erlangung des DFK Förderpreises 2013. Die Jury befand Ihre Einreichung für die Beste.

Die Preisübergabe findet statt am:
Freitag, 21. Juni 2013, 14:30 Uhr, Aula des Hauptgebäudes der Universität Innsbruck
(Christoph-Probst-Platz, Innrain 52, 1. Stock)

Gerne können Sie in Begleitung kommen.

Mit freundlichen Grüßen

Dr. Yorck Schmidt
Vorstandsvorsitzender

Mag. Huberta Scheiber
Generalsekretärin

U.A.w.g. auf beigefügtem Antwortformular.

Deutscher Freundeskreis der Universitäten in Innsbruck e.V.
Generalsekretärin Dir. Mag. Huberta Scheiber
Rechengasse 7, A-6020 Innsbruck
Telefon: +43 (0) 512 501-910, Telefax: +43 (0) 512 501-905
E-Mail: scheiber@studentenhaus.at

Abbildung 4. Persönliche Einladung zur Preisübergabe des Deutschen Freundes- und Förderkreises der Universitäten in Innsbruck e.V.

Um die Rückantwortrate zu optimieren, wurden die ÖGARI Mitglieder wenige Wochen vor Durchführung der Umfrage in der Vereinszeitung auf die anstehende Umfrage hingewiesen, zudem wurde noch wenige Tage vor Aussendung der Umfrage ein Erinnerungsemail ausgesandt (Abbildung 5).

Arbeitsbedingungen von AnästhesistInnen in Österreich

Sehr geehrte KollegInnen,

in den kommenden Wochen möchten wir Sie herzlich per Email einladen an einer Umfrage unter den AnästhesistInnen in Österreich teilzunehmen zu Arbeitsbelastung, Gesundheit und Zufriedenheit am Arbeitsplatz . Mit der Umfrage möchten wir einen Beitrag leisten die Arbeitsbedingungen von AnästhesistInnen in Österreich zu verbessern.
Ihre Antworten werden mittels einer elektronischen Datenbank anonymisiert ausgewertet. Die Österreichische Gesellschaft für Anästhesie, Reanimation und Intensivmedizin (ÖGARI) unterstützt diese Umfrage dankenswerterweise durch Aussendung der Umfrage an alle Mitglieder. Die ÖGARI kann die Ergebnisse für standespolitische Zwecke nutzen. Zudem werden die wichtigsten Ergebnisse in einer der nächsten Ausgaben der A+IC NEWS vorgestellt. Es ist geplant, die Studie in einem international anerkannten peer-reviewed Journal zu veröffentlichen. Eine hohe Rücklaufquote ist für die Aussagekraft der Umfrage sehr wichtig.
Im Namen des gesamten Teams und des Vorstandes der ÖGARI bedanke ich mich für Ihre geschätzte Mitarbeit und hoffe auf eine rege Teilnahme.

Beste Grüße,

PD Dr. Peter Paal, EDAIC, EDIC
Univ. Klinik für Anästhesie und Intensivmedizin Innsbruck
peter.paal@uki.at

Abbildung 5. Vorinformation der Mitglieder der Österreichischen Gesellschaft für Anästhesiologie, Reanimation und Intensivmedizin über die bevor stehende Studie (ÖGARI) wenige Tage vor der Aussendung der Umfrage.

Weiters wurden alle Teilnehmer der Umfrage eingeladen an einer Verlosung von drei Gutscheinen im Wert von je 100€ teil zu nehmen. Schlussendlich wurde der Fragebogen am 24. Juli 2013 ausgesandt. Am 5. August wurde ein Erinnerungsemail an alle ÖGARI Mitglieder versandt welche bis dahin noch nicht an der Umfrage teilgenommen hatten. Am 11. August wurde um Mitternacht die Umfrage abgeschlossen. Die Master Arbeit konnte in der vorliegenden Form am 22. Oktober 2013 zur finalen Beurteilung eingereicht werden.

In zwei Sitzungen wurden Korrelationen mit Christian Traweger und Johann Kinzl diskutiert und mit dem Chi-Quadrat-Test berechnet. Eine Differenz zwischen einzelnen Skalen und demographischen Gruppen wurde mittels ANOVA berechnet. Ein P-Wert <0,05 wurde als statistisch signifikant erachtet.

Diese Arbeit hat Limitationen. Erstens, ist davon auszugehen, dass die motiviertesten AnästhesistInnen in Österreich an dieser Studie teilgenommen haben. AnästhesistInnen, die resigniert, unzufrieden, überarbeitet oder gar im Burnout waren haben wahrscheinlich an dieser Umfrage weniger häufig teilgenommen da ihnen das notwendige Interesse oder die notwendigen Ressourcen fehlten. Zweitens könnten die Ergebnisse nicht die Wahrheit widerspiegeln, da einige Fragen einen sehr persönlichen Charakter hatten und deswegen die Antworten möglicherweise nicht der Wahrheit entsprechend gegeben wurden.

6. Theoretischer Hintergrund

Für die Diskussion des Arbeitsmodells, das in dieser Arbeit vorgestellt wird, wurden die Werte *Arbeitszufriedenheit*, *Leistungsmotivation* und *Commitment* gewählt, da sie zum Verständnis wie AnästhesistInnen in einem Krankenhaus arbeiten essentiell sind und in einer kausalen Verbindung zu einander stehen.

6.1. Arbeitszufriedenheit

Wenn man von Arbeitszufriedenheit spricht sollte im Vorfeld zuerst einmal der Begriff definiert werden. STAHL unterscheidet bei Zufriedenheit in einer Organisation eine solche, die sich nach innen richtet (die Arbeits- oder Mitarbeiterzufriedenheit) und eine die sich nach außen zu den externen Stakeholdern einer Organisation hin richtet (zum Beispiel Kundenzufriedenheit) (STAHL, 2013a). Die Arbeitszufriedenheit einer Organisation ist nicht nur für die Mitarbeiter einer Organisation wichtig, sie wirkt auch auf die externen Stakeholder. Arbeitszufriedenheit bestimmt deshalb über Regelmechanismen im Inneren (zum Beispiel Fluktuation, Krankenstände) und auch nach außen (zum Beispiel Employer Branding, Attraktivität des Unternehmens) wesentlich den Erfolg des Unternehmens mit. Die Arbeitszufriedenheit ergibt sich laut dem Equity Modell von JOHN STACEY ADAMS aus dem Vergleich von Input und Output (ADAMS, 1965, 267-299) (Abbildung 6).

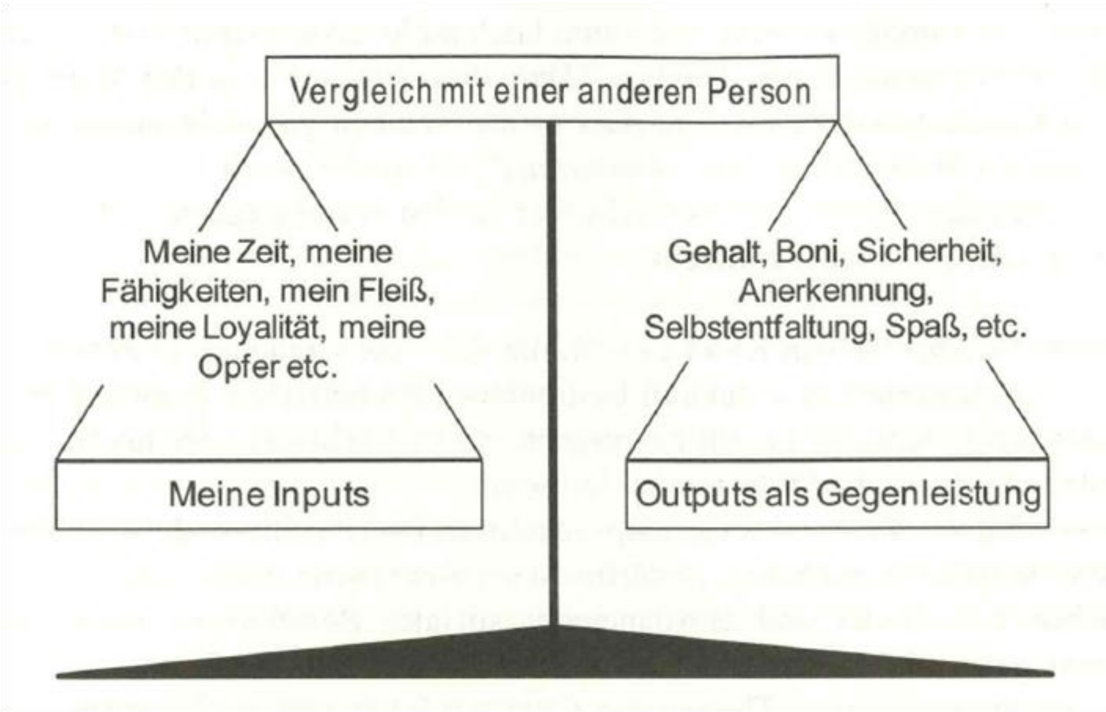

Abbildung 6. Das Equity-Modell der Arbeitszufriedenheit in Anlehnung an JOHN STACEY ADAMS aus dem Vergleich von Input und Output (ADAMS, 1965, 267-299).

WEINERT benennt im Rahmen der Arbeiszufriedenheit für den Vergleich von Ist und Soll drei wichtige Parameter: Persönlichkeitsmerkmale, Arbeitssituation und Beanspruchung (WEINERT, 1998). In der Folge sollen diese drei Parameter im Lichte der aktuellen Ergebnisse diskutiert werden.

Persönlichkeitsmerkmale scheinen einen besonders wichtigen Einfluss auf die Arbeitszufriedenheit zu haben. Zu den Persönlichkeitsmerkmalen, die besonders positiv wirken zählen laut STAHL zum Beispiel Extraversion, ein starkes Selbstkonzept, eine hohe Selbstwirksamkeitserwartung und eine ausgeprägte Selbstkontrolle (STAHL, 2013a). Natürlich sind AnästhesistInnen wie andere Menschen auch in ihren Persönlichkeitsmerkmalen genetisch und auch von den Ereignissen in ihrer frühen Kindheit im Wesentlichen determiniert. Aber das Arbeitsklima kann, vor allem wenn es über eine längere Periode einwirkt, die Persönlichkeit verändern. Dieser Einfluss kann nun auf die Persönlichkeitsmerkmale positiv oder negativ verstärkend wirken. Ein Maximalbeispiel eines negativen Einflusses auf die Persönlichkeitsmerkmale war ein Streit um Poolgelder an der Universitätsklinik für Anästhesie und Intensivmedizin der 2009 im Suizid eines Anästhesisten gipfelte (MARKARITZER, 2009). Die aktuellen Ergebnisse zeigen dass >40% der Anästhesisten in Österreich belastet sind. Zum Beispiel haben über 50% für sich, ihre Familien und Freunde zu wenig Zeit, 11% genießen ihre Freizeit nie oder selten, und 18% denken beim Aufstehen nie oder selten dass der anstehende Tag gut werden wird. Hier besteht von Seiten der Krankenhäuser sicherlich ein Handlungsbedarf diese Risikopersonen am Arbeitsplatz zu entlasten und psychologisch zu betreuen, und in der Folge zum Wohle ihrer Gesundheit zu fördern und zu coachen.

Die **Arbeitssituation** kann die Arbeitszufriedenheit steigern, wenn der Anästhesist am Arbeitsplatz Anerkennung, Gerechtigkeit und Sinn erfährt. Zudem soll die geforderte Arbeit interessant sein und in einem gesunden Maß fordern. Anreiz- und Entgeltsystems sollten an die individuelle Leistung gekoppelt sein. Weiters sollten Eigeninitiative und Selbstverantwortung gefördert werden und Platz sein für sozial wertvolle Kontakte. Die Ergebnisse dieser Studie zeigen, dass 43% ihren Arbeitsplatz während der Arbeitszeit maximal fünf Minuten verlassen können, 59% kommen wegen zu viel Arbeit nicht oder verspätet in die Pause, und 36% kommen wegen zu viel Arbeit verspätet in den Feierabend.

Als letzter Faktor kann **Beanspruchung** die Arbeitszufriedenheit steigern. Die Arbeit wird sich dann positiv auf die Arbeitszufriedenheit auswirken wenn die Arbeit ohne Störung und Unterbrechung ausgeführt werden kann. Zudem sollten möglichst große Autonomie bei der Planung, Durchführung und Kontrolle der Tätigkeiten bestehen. Insgesamt wird die Arbeit umso befriedigender wahrgenommen je mehr positive Gefühle durch Kompetenzerleben und

Selbstwirksamkeit entstehen und möglichst wenig negative Gefühle (zum Beispiel durch Nervosität und Stress) entgegenwirken (WIELAND et al., 2005). Die Ergebnisse aus der vorliegenden Studie zeigen, dass zum Beispiel 53% oft oder sehr oft mehrere Arbeiten gleichzeitig verrichten müssen, und nur 45% oft oder sehr oft bei der Arbeit unterbrochen werden weil etwas Wichtiges dazwischen kommt.

Arbeitszufriedenheit im Krankenhaus ist wegen der Einflussnahme auf drei arbeitsrelevante Faktoren von großer Bedeutung. *Fehlzeiten*, *Fluktuation* und *Produktivität* wurden mit Arbeitszufriedenheit in Zusammenhang gebracht. Diese drei Faktoren sollen im Anschluss diskutiert werden:

Mehrere Studien konnten einen negativ Korrelation zwischen *Fehlzeiten* und Arbeitszufriedenheit nachweisen (STAHL, 2013a), wobei der Zusammenhang in einigen Studien nicht sehr eindeutig war. Auch bei der *Fluktuation* konnte eine negative Korrelation nachgewiesen werden (GEBERT et al., 2000). Keine Korrelation konnte interessanterweise bisher zwischen Arbeitszufriedenheit und *Produktivität* hergestellt werden, auch wenn der Schluss dass es eine Beziehung gibt nahe liegt. Viele Unternehmen, besonders im kreativen Bereich gewähren deshalb zum Beispiel ihren Mitarbeitern Freiräume im wahrsten aber auch im übertragenen Sinn, um die Arbeitszufriedenheit und in der Folge die Produktivität zu steigern. Die vorliegende Studie hat nicht den Einfluss der Arbeitszufriedenheit auf die drei angesprochenen Faktoren analysiert. Aus anderen Berichten kann jedoch abgeleitet werden, dass zahlreiche Leitungsteams, die Arbeitszufriedenheit als zentralen Punkt für das Wohlergehen in einem Unternehmen erkannt haben und fördern wollen (PADOSCH et al., 2011, 364-369; SCHMIDT et al., 2011, 517-524, 2012, 630-634, 636-639); es bleibt zu hoffen dass diese Erkenntnisse auch breitflächig in Krankenhäusern umgesetzt wird.

Mit verschiedenen Methoden (zum Beispiel Diskussionen, Interviews oder Umfragen) können Krankenhäuser die Arbeitszufriedenheit ihrer Anästhesisten bewerten (STAHL, 2013a). Diese Methoden sollen aber bündig, umsetzungsrelevant und zielgerichtet sein um den Aufwand zu rechtfertigen. Sinnlos, wenn nicht gefährlich ist wenn Methoden zur Erfassung der Arbeitszufriedenheit Indifferenz oder Unzufriedenheit als Zufriedenheit einstufen (zum Beispiel wenn viele Indifferente oder Unzufriedene nicht an Umfragen teilnehmen und damit eine Verzerrung des Gesamtbildes durch die Antworten der Zufriedenen entsteht). Eine Messung der Arbeitszufriedenheit sollte alle Gruppen von Zufriedenen und diffus Unzufriedenen erfassen. Eine valide Methode um Arbeitszufriedenheit zu klassifizieren und zu definieren ist die Einteilung nach Agnes Bruggemann (Abbildung 7).

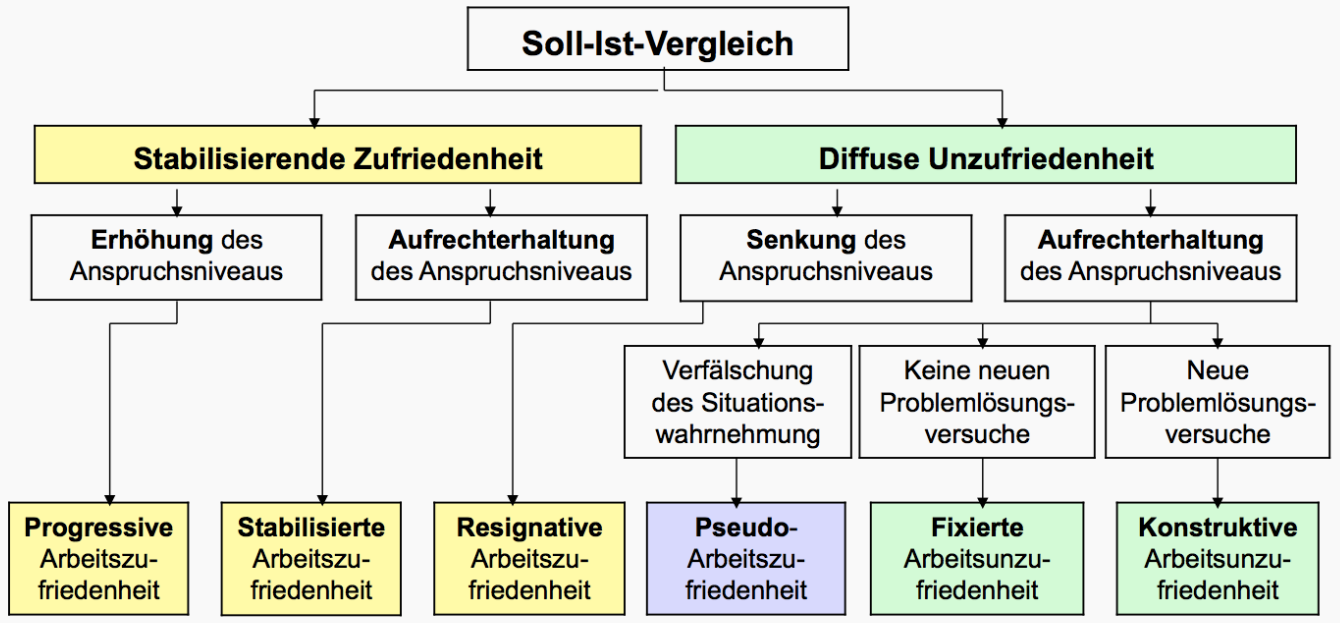

Abbildung 7. Eine valide Methode um Arbeitszufriedenheit zu klassifizieren und zu definieren ist die Einteilung nach Agnes Bruggemann (BRUGGEMANN et al., 1975).

Anhand von reellen Situationen aus dem Arbeitsumfeld der „Universitätsklinik für Anästhesie und Intensivmedizin“, an der der Autor seit gut acht Jahren tätig ist, soll dieses Modell nun dem Leser näher gebracht werden. Die Grundannahme bei diesem Modell ist ein Soll-Ist Vergleich der aktuellen Arbeitssituation. Dieser Soll-Ist Vergleich kann entsprechend der vorliegenden Situation nun zu einer dichotomen Entscheidung führen: 1. stabilisierende Zufriedenheit (der Soll-Ist Vergleich fällt positiv aus) oder 2. diffuse Unzufriedenheit (der Soll-Ist Vergleich fällt negativ aus).

Im Rahmen der **Stabilisierenden Zufriedenheit** kann **bei positivem Soll-Ist Vergleich** als *erste Möglichkeit* das Anspruchsniveau erhöht werden, progressive Arbeitszufriedenheit resultiert. Ein Beispiel dazu ist der Anästhesist, welcher seinen Arbeitseinsatz gerecht belohnt erachtet, dessen Zufriedenheit ist gegeben. Mit dem nun erreichten Niveau der Fach- und Methodenkompetenz (zum Beispiel er kann nun Allgemeinanästhesien bei Kindern selbstständig durchführen) setzt er sich zugleich neue und höhere Ansprüche, die er vielleicht bisher gar nicht erreichbar erachtete. Eine *weitere Möglichkeit* bei der Stabilisierenden Zufriedenheit ist dass der Soll-Ist Vergleich positiv ist, aber das Anspruchsniveau unverändert bleibt, daraus resultiert eine stabilisierende Arbeitszufriedenheit. Ein Beispiel dazu ist die Anästhesistin die mit dem Erreichten sehr zufrieden ist und aufgrund verschiedener Umstände (zum Beispiel Fokus auf Privates) nichts Neues mehr erreichen muss. Die *letzte Möglichkeit* im Rahmen einer Stabilisierenden Arbeitszufriedenheit ist wenn der Soll-Ist Vergleich negativ ist und das Anspruchsniveau reaktiv gesenkt wird. Ein Beispiel dazu, ist die Anästhesistin die in den ersten

Jahren ihrer Karriere sehr viele klinische Fortschritte gemacht hat und auch auf dem besten Wege zur Habilitation war. Nachdem sie aber zweifache Mutter wurde hat sie ihren Lebensmittelpunkt nun außerhalb der Arbeit. Sie arbeitet seit der Rückkehr aus der Karenz in Teilzeit. Die Arbeit gefällt ihr nach wie vor gut, sie verspürt aber nicht mehr das Feuer der frühen Arbeitsjahre. Sie fühlt und weiß auch, dass sie neben der Familie- ihr Mann ist von Berufs wegen sehr wenig zuhause- nicht mehr die Energie und Zeit hat die Bilderbuchkarriere von früher fortzusetzen. Resignative Arbeitszufriedenheit resultiert.

Endet der **Soll-Ist Vergleich negativ** kommt es zu einer **Diffusen Unzufriedenheit**. Als *erste Möglichkeit* kann nun das Anspruchsniveau unverändert bleiben, die Arbeitssituation wird aber geschönt wahrgenommen. Ein Beispiel dafür ist der Anästhesist der eine große Karriere angestrebt hat, aber aufgrund diverser Umstände nie eine Position in der Hierarchie erreicht hat die er angestrebt hat. Da der Soll-Ist Vergleich deshalb negativ ist, und er sein Anspruchsniveau aufrecht erhält muss er, um sich die Situation akzeptabel zu machen, seine derzeitige Position gut reden; Pseudo-Arbeitszufriedenheit resultiert. Die *zweite Möglichkeit* ist im Rahmen eines negativen Soll-Ist Vergleichs und bei unverändertem Anspruchsniveau dass auf Schönrederei aber auch auf Verbesserungsmöglichkeiten verzichtet wird eine fixierte Arbeitsunzufriedenheit folgt daraus. Das Beispiel für diesen Fall ist ein Anästhesist der mit seiner Situation nicht zufrieden ist, da er „weiß“ dass er das Gleiche leisten könnte wie seine Kollegen die meistens die Patienten für die spannenden Operationen anästhesisieren. Aufgrund seiner wiederholten Reibereien mit der Geschäftsführung wird er meist nur mehr in den „Besenkammern“ Operationssälen eingesetzt, Frust resultiert. Als *letzte Möglichkeit* eines negativen Soll-Ist Vergleichs kann bei unverändertem Anspruchsniveau an Lösungsversuchen gearbeitet werden um die Situation zu verbessern. Ein treffendes Beispiel für diese konstruktive Arbeitsunzufriedenheit ist der Kollege der aufgrund seiner sehr ruhigen Art bei der Zuteilung interessanter Arbeitsbereiche meist gegenüber den lauteren, aber nicht besser qualifizierten, KollegInnen den Kürzeren gezogen hat. Der Kollege hat vor ca. zwei Jahren an eine andere Universitätsklinik gewechselt. Dort wurde sein Potential erkannt, wertgeschätzt und mittlerweile ist er zu einem der Leitenden Bereichsoberärzte jener Universitätsklinik aufgestiegen.

6.2. Leistungsmotivation

Die Motivation zur Leistung (Leistungsmotivation) scheint dem Menschen durch die Evolution angeboren und in die Wiege mitgegeben worden zu sein (STAHL, 2013a). Tiere, auch Primaten, verfügen nicht über Leistungsmotivation. Leistungsmotivation muss nicht auf Leistung im Beruf beschränkt sein, mittlerweile wurden in unserer Gesellschaft diverse Formen der

Leistungsmotivationerkannt, wie zum Beispiel Leistungsmotivation im sportlichen, politischen oder sozialen Bereich. Entgegen diversen Unkenrufen geben empirische Studien auch keinen Hinweis dass die Leistungsmotivation jüngerer Generation absinkt, es haben sich aber sehr wohl die Leistungsmotive als auch die Werte verschoben.

Manche Menschen haben durch ihre frühkindliche Erziehung (die Persönlichkeit eines Menschen wird wesentlich in den ersten drei Lebensjahren geformt) eine besonders ausgeprägte Leistungsmotivation (need for achievement) entwickelt. Zudem kann Leistungsmotivation durch gelenkte Lernprozesse verstärkt werden (McClelland, 1975). Zum Verständnis warum eine Person motiviert ist Leistung zu erbringen sollte man sich die drei typischen Leistungshaltungen präsent machen (Geisler, 1977):

- Selbstbewusste Leistungshaltung; diese ist mit der eigenen Persönlichkeit konform. Hohe Leistungsanforderungen werden angenommen, ohne die Person zu destabilisieren.
- Defensive Leistungshaltung; die Person geht Leistung aus dem Weg. Hohe Leistungsanforderungen destabilisieren die Person.
- Kompensatorische Leistungsanforderung; die Person sieht im Leistungserfolg die Möglichkeit das Selbstwertgefühl zu steigern und das Umfeld zu gestalten. Minderwertigkeitskomplexe können eine starke Triebfeder sein (Stahl, 2013a).

Leistungsmotivation ist *tätigkeitszentriert*, wenn der Anreiz aus der Durchführung einer Tätigkeit stammt. Dieser Anreiz hat implizite (unbewusste) Motive.

Leistungsmotivation ist *zweckorientiert*, wenn der Anreiz aus dem zu erwartenden Ergebnis kommt. Dieser Anreiz speist sich sowohl aus impliziten (zum Beispiel innere Motivation zur Leistungserbringung) als auch expliziten Motiven (zum Beispiel Lob).

Leistungsmotivation gründet sich somit auf jedem Fall auf impliziter Motivation. Explizite Motive übersetzt die Person entsprechend ihrem Wertesystem in innere Motivatoren und entscheidet dann aufgrund ihres inneren Programms (bestehend unter anderem aus Werte- und Motivationssystems) (Stahl, 2013a) (Abbildung 8).

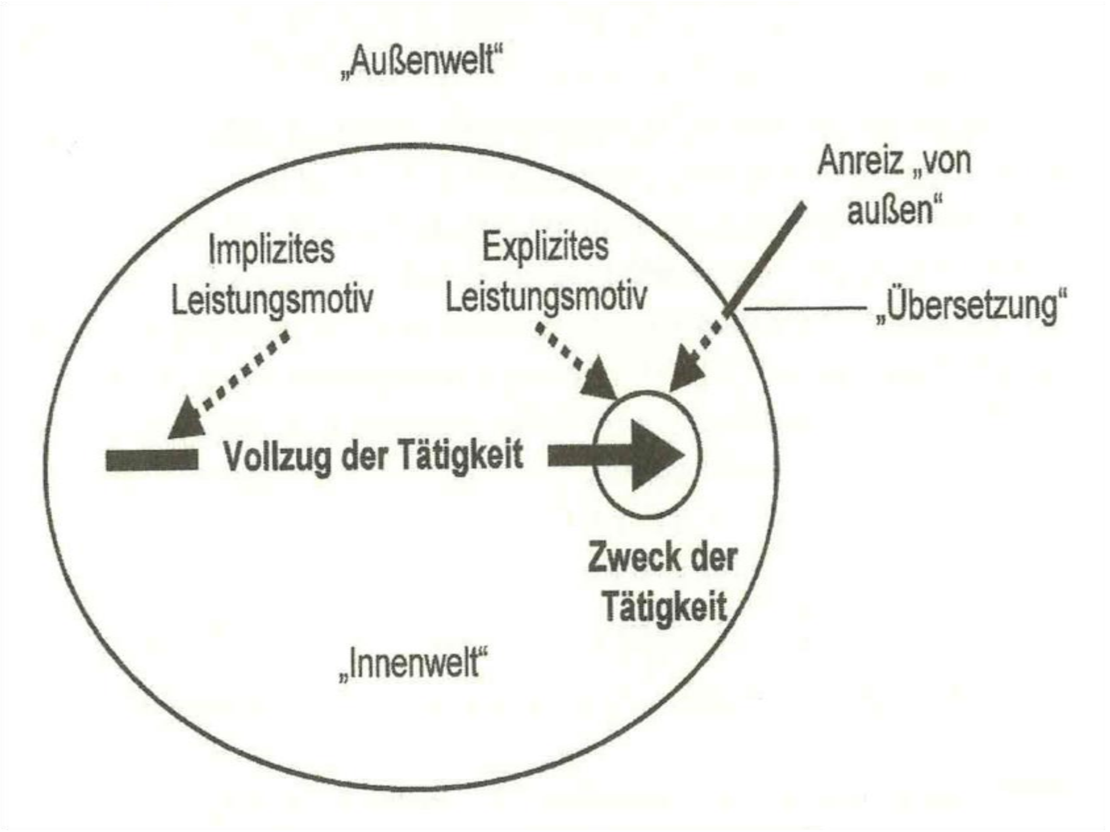

Abbildung 8. Leistungsmotivation gründet sich immer auf impliziter Motivation. Explizite Motive übersetzt die Person entsprechend ihrem Wertesystem in innere Motivatoren und entscheidet dann aufgrund ihres inneren Programms (bestehend unter anderem aus Werte- und Motivationssystems) (STAHL, 2013a).

Für Führungskräfte sind zwei Aspekte in der Leistungsmotivation wichtig.

- Personen sind keine trivialen Systeme, bei denen eine Wirkung zu einer vorhersehbaren Reaktion führt. Der Anspruch Personen steuern zu können muss einer Bescheidenheit weichen.
- Um jemanden zu motivieren muss man sein inneres Programm kennen. Menschenkenntnis und Individualisierung sind in der modernen Führung sehr wichtig. In der Summe muss also klassische Führung einer postklassischen Führung weichen (Tabelle 2).

Tabelle 2. Gegenüberstellung von Beispielen von klassischer und Merkmalen postklassischer Führung, adaptiert nach (STAHL, 2013a).

**Klassische Führung (Beispiele)**	**Postklassische Führung**
Autoritär (Lewin)	Zirkulär (Feedbackschleife zw. Führenden und Geführten
Kooperativ (Lewin	Empathie
Laissez-faire (Lewin	Gerechtigkeit
Konsultativ	Neues Verständnis der Macht
Partizipativ	Entsprechend dem Menschenbild in der Organisation (komplexer Mensch)
Dienend	Beantwortung der Sinnfrage
Despotisch	Balance zwischen Kontrolle und Vertrauen
Situativ	Berücksichtigung der Wertedynamik

Um das Thema Leistungsmotivation in der Anästhesiologie möglichst bildhaft darzustellen möchte der Autor in der Folge einen Fall aus der Praxis vorstellen: Ein junger Kollege hat sich am Ende des Medizinstudiums bereits sehr auf den Start in die Arbeit gefreut. Wie er nun seine Arbeitsstelle antritt kann er sein Glück kaum fassen. Der Primarius der Abteilung für Anästhesiologie ist zuvorkommend, die KollegInnen in der Abteilung sehr hilfsbereit und kompetent, die Pflege ist sehr nett und die Patienten die der junge Kollege betreuen muss bieten ihm reichlich abwechslungsvolle Arbeit. Da der junge Anästhesist von seinen älteren KollegInnen Schritt für Schritt an immer größere Aufgaben herangeführt wird fühlt er sich selten überfordert, und wenn weiß er dass Hilfe in der Nähe ist. Der junge Anästhesist geht in der Arbeit auf. Er merkt fast nicht wie aus den ersten Tagen die ersten Wochen, dann Monate werden. Der junge Anästhesist fühlt sich an seinem Arbeitsplatz als Teil einer Familie, er ist angekommen und erlebt bei der Arbeit es öfteren einen Zustande des „Flow“ (Abbildung 9).

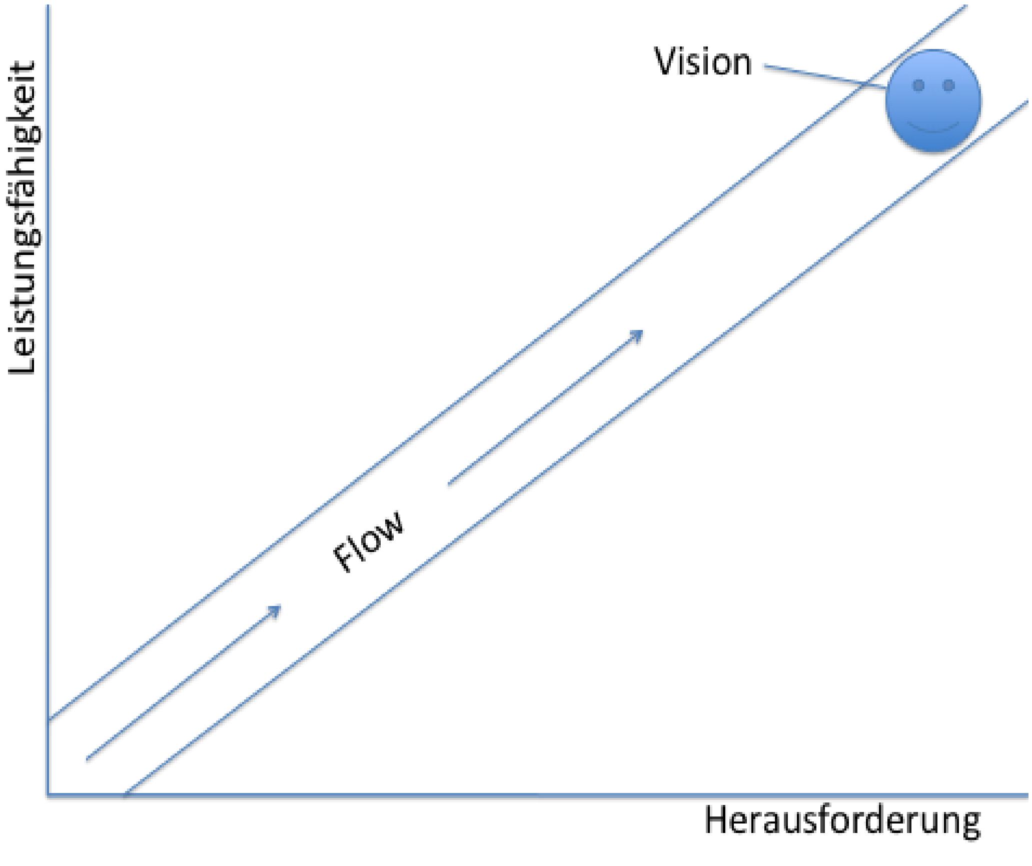

Abbildung 9. Flow bei der Arbeit durch eine richtige Mischung aus Leistungsfähigkeit und Herausforderung, adaptiert nach (CZIKZSENTMIHALYI, 2004).

6.3. Commitment

Bevor man über Änderungen der Organisationsgestaltung spricht, um das Commitment zu verbessern, soll der Begriff Commitment definiert werden (STAHL, 2013a). Commitment steht vor

allem für Bindung, im Sinne von Festlegung und Verpflichtung. Bei Commitment kann man unterscheiden zwischen persönlichen Commitment (Bindung an andere Menschen), interorganisationalen Commitment (Bindung zwischen Organisationen) und das für diese Arbeit relevante organisationale Commitment (Bindung von Menschen an eine Organisation). Commitment bedeutet außerdem eine langfristige Bindung mit Zukunftsbezug, während zum Beispiel Involvement die Bindung in die Tiefe (zum Beispiel durch Vertiefung in Details) zum aktuellen Zeitpunkt bedeutet. Zudem kann Commitment verstanden werden als Abwägung von Antworten auf vier Fragen:

- Wie attraktiv ist die Beziehung zwischen Arbeitgeber und der Organisation für die er tätig ist?
- Welche Alternativen bestehen zur aktuellen Beziehung?
- Wie hoch wäre der Verlust an Investitionen die der Arbeitgeber in die aktuelle Beziehung gesteckt hat, wenn er die Organisation wechseln würde?
- Wie hoch wären bei einem Wechsel der Organisation die Gewissenskosten?

Die Attraktivität einer Beziehung ergibt sich durch das Aufwiegen von Nutzen (zum Beispiel Arbeitsklima, Bezahlung, Karrierechancen) gegenüber Aufwendungen (zum Beispiel investierte Ressourcen wie Lebenszeit), beides sowohl materieller als auch immaterieller Dimension. Ein Maßstab ergibt sich aus dem Vergleich mit ähnlichen Situationen in der Vergangenheit, Annahmen für die eigene Zukunft oder durch den Vergleich mit anderen Menschen (Abbildung 10, STAHL, 2013a).

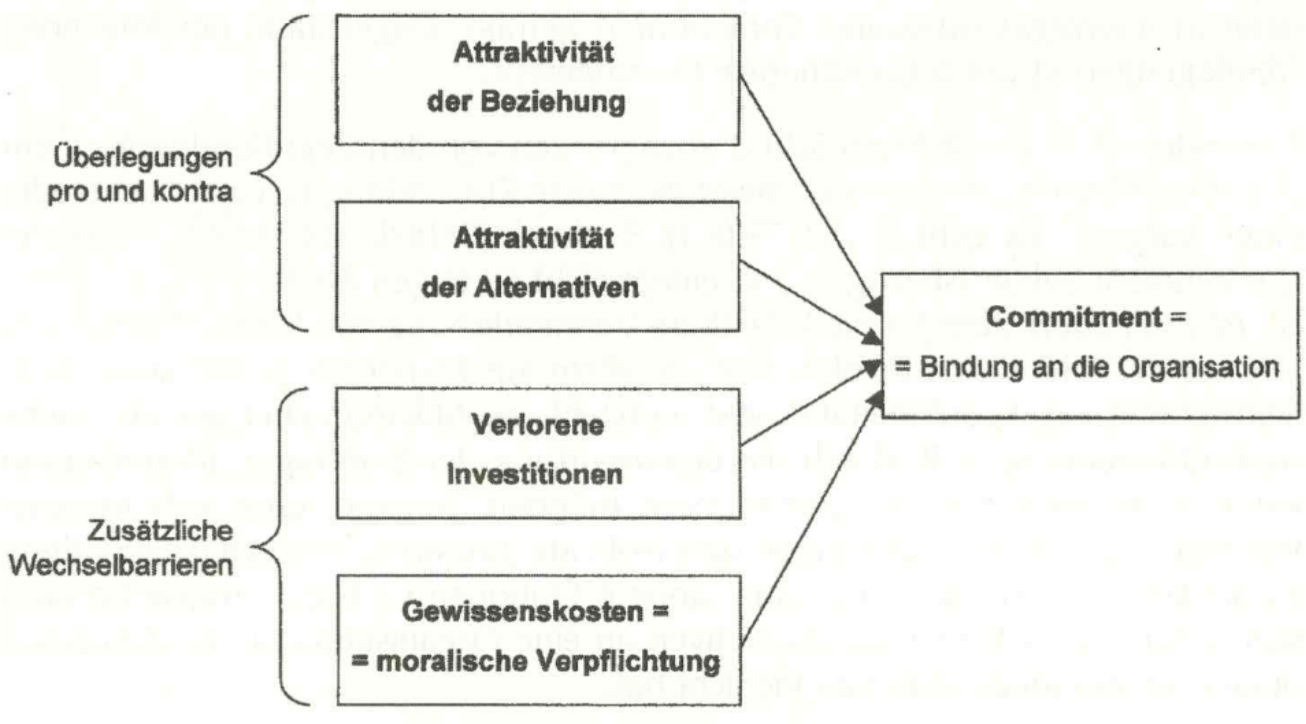

Abbildung 10. Schematisches Modell für organisationales Commitment (STAHL, 2013a).

Wiegt man die aktuelle Beziehung mit Alternativen ab so ergeben sich drei Möglichkeiten:

1. Die aktuelle Beziehung ist attraktiv und es gibt keine gleichwertigen Alternativen. In diesem Fall ist das Commitment des Arbeitnehmers gegenüber der Organisation sehr hoch.
2. Die aktuelle Beziehung ist attraktiv, wird aber von einer anderen Alternative deutlich überboten. In diesem Fall erscheint die erste Beziehung im Vergleich zur Alternative weniger attraktiv. Der Arbeitnehmer muss entscheiden ob ein Wechsel sinnvoll ist, wenn man Investitionsverlust und Gewissenskosten mit ein berechnet. Diese Situation besteht immer häufiger bei Hochqualifizierten, inkl. AnästhesistInnen. Krankenhäuser sollten Strategien entwickeln um das bisherige Commitment von AnästhesistInnen hoch zu halten.
3. Die aktuelle Beziehung ist unattraktiv, aber auch alle Alternativen sind unattraktiv evtl. sogar deutlich unattraktiver. In diesem Fall ist das Commitment zwar gering, aber ein Wechsel unwahrscheinlich. Der Arbeitnehmer ist gefangen in einer unattraktiven Beziehung. Diese Situation besteht häufig bei Niedrigqualifizierten.

Für das Commitment relevant sind auch die Investitionsverluste, welche entstehen wenn man die bisherige Organisation verlässt (zum Beispiel Verlust von Freunden, Ansehen, Position im Unternehmen; Nähe zum Arbeitsplatz). Auch relevant sind die Gewissenskosten, welche bei einem Organisationswechsel entstehen. Ältere (zum Beispiel Nachkriegsgeneration und Baby Boomer) verspüren deutliche höhere Gewissenskosten als Jüngere die wesentlich dynamischer beim Wechsel einer Organisation und einer Beziehung sind (zum Beispiel Generation X und Y).
Organisationen sollten versuchen die Investitionsverluste ihrer Mitarbeiter möglichst hoch zu halten, sie müssen aber darauf achten, dass sie ihre Mitarbeiter nicht in die Enge treiben, da diese mit Reaktanz reagieren könnten. Reaktanz ist ein Phänomen, das vom Psychologen JACK BREHM ausführlich untersucht wurde und eine komplexe Abwehrreaktion beschreibt, bei der Widerstand gegen äußere oder innere Einschränkungen entsteht. Die betroffene Person möchte sich durch Auflehnung gegen das Verbot gleichsam Freiheiten zurückholen, auch wenn das unter Umständen gar nicht realisiert werden kann. Typisch für die Reaktanz ist eine Valorisierung der verbotenen Freiheitsgrade, evtl. war die aktuell verbotene Handlungsmöglichkeit der Person völlig unwichtig und gewinnt erst durch das Verbot einen eindringlichen Wert (BREHM, 1966).

6.4. Exkurs

Aus dem zuvor unter 6.1- 6.3 Besprochenem ergibt sich, dass die jungen Generationen (zum Beispiel Generation Y und Z) die nun auf den Arbeitsmarkt kommen mental anders gestrickt sind als die älteren Generationen. So sind sie im Unterschied zu den früheren Generationen seit dem 2. Weltkrieg wesentlich weniger Hierarchie hörig, ihre Handlungsautonomie ist wesentlich höher, das Gewicht im Leben liegt weiter im Privaten und Jobhopping ist kein Unding sondern natürlicher Bestandteil des Arbeitslebens (Abbildung 11.)

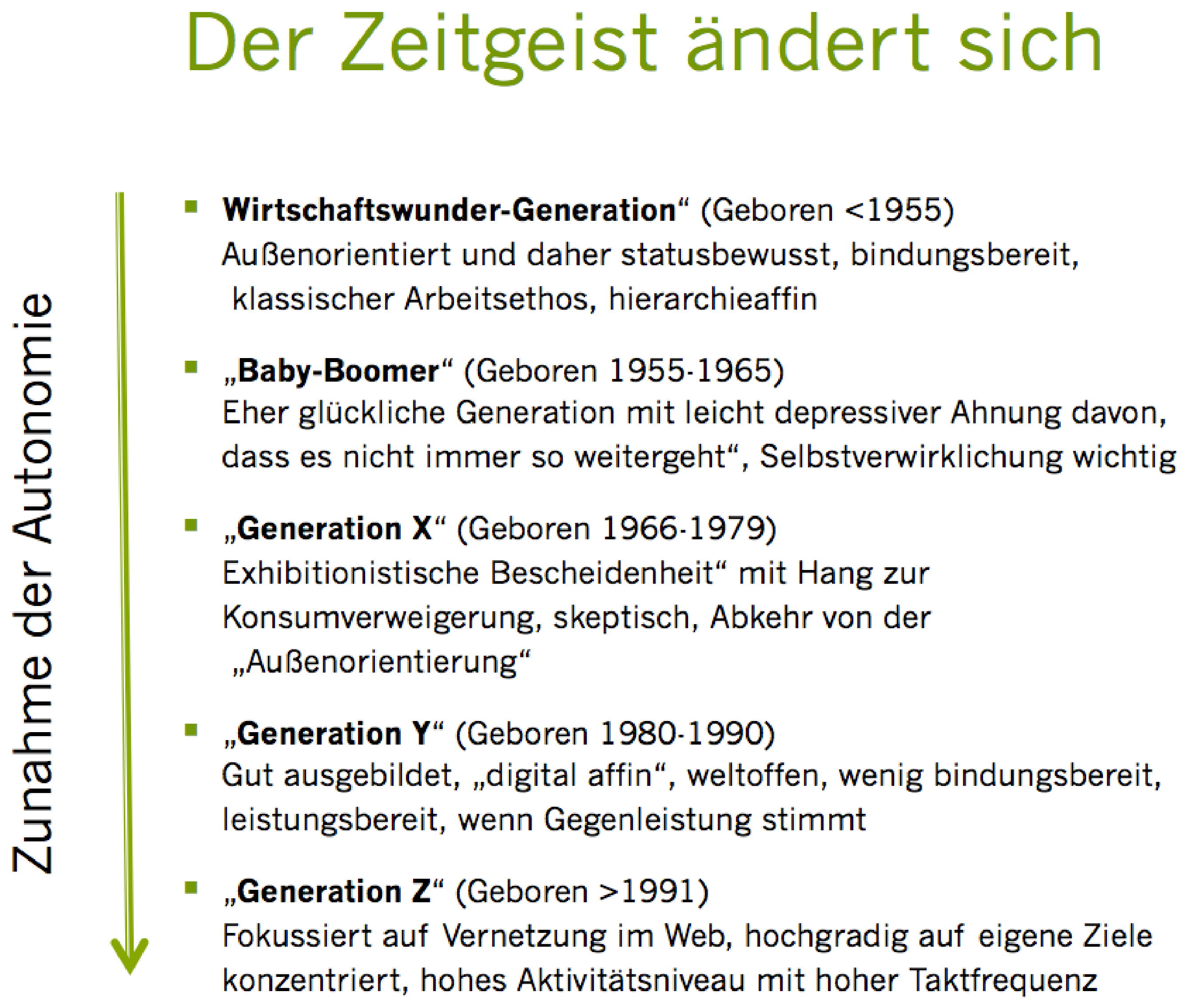

Abbildung 11. Die jungen Generationen (zum Beispiel Generation Y und Z) die nun auf den Arbeitsmarkt kommen sind mental anders „gestrickt“ als die älteren Generationen (STAHL, 2013c).

Mehrere Studien konnten zeigen, dass die Leistungsmotivation jüngerer Generation nicht abgesunken ist, es haben sich einzig die Leistungsmotive als auch die Werte verschoben (Abbildung 12).

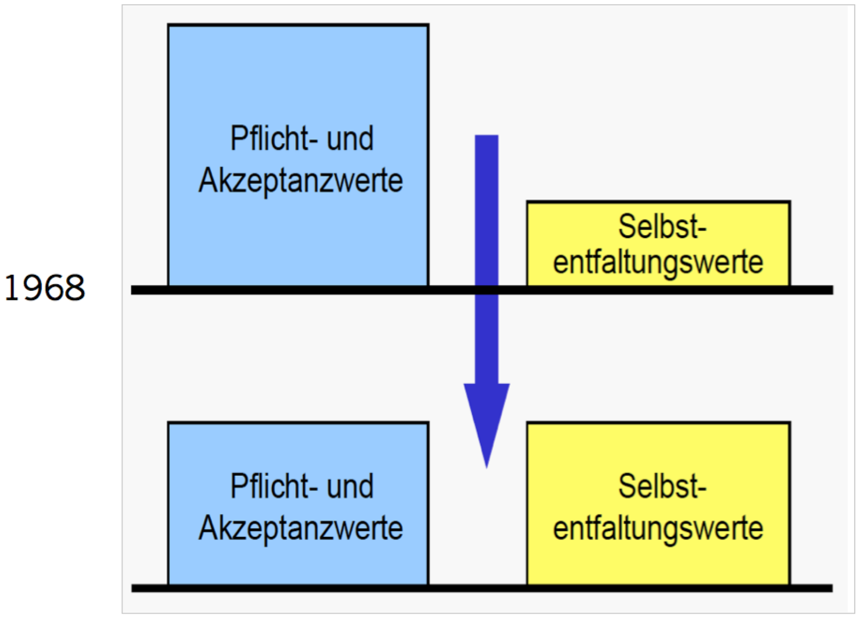

Abbildung 12. Besonders seit der Sozialrevolution im Jahr 1968 hat sich der Schwerpunkt von Pflicht- und Akzeptanzwerten weg hin zu Selbstentfaltungswerten verschoben (STAHL, 2013c).

6.5. Begründung der Schleife Arbeitszufriedenheit-Leistungsmotivation-Commitment-Arbeitszufriedenheit

Aus dem bisher unter 6.1-6.4 Besprochenem kann plausibel abgeleitet werden, dass Arbeitszufriedenheit zu einer gesteigerten Leistungsmotivation führt und diese wiederum kann in einem erhöhten Commitment enden. Wenn man den Gedankengang weiterführt ist unschwer ersichtlich, dass hohes Commitment wiederum die Arbeitszufriedenheit positiv beeinflussen kann, da Investitionsverluste und Gewissenskosten abnehmen. Im Idealfall „ist der Anästhesist in seinem Krankenhaus − wo er schon immer arbeiten wollte- angekommen“. Er fühlt sich an seinem Arbeitsplatz zuhause und unter Freunden.

Abbildung 13 zeigt die kausale Feedbackschleife von Arbeitszufriedenheit, Leistungsmotivation und Commitment. Eine Zunahme von Commitment verstärkt wiederum die Arbeitszufriedenheit, da der Kopf frei ist weil der Anästhesist weiß dass er am richtigen Arbeitsplatz angekommen ist. Er muss sich nicht mehr mit Umzug, Arbeitsplatz- und Freundeswechsel auseinandersetzen. Die Menschen an seinem aktuellen Arbeitsplatz sind für den Anästhesist gute Freunde, fast schon

Teil seiner Familie. Somit steigt durch hohes Commitment die Arbeitszufriedenheit. Die Feedbackschleife schliesst sich.

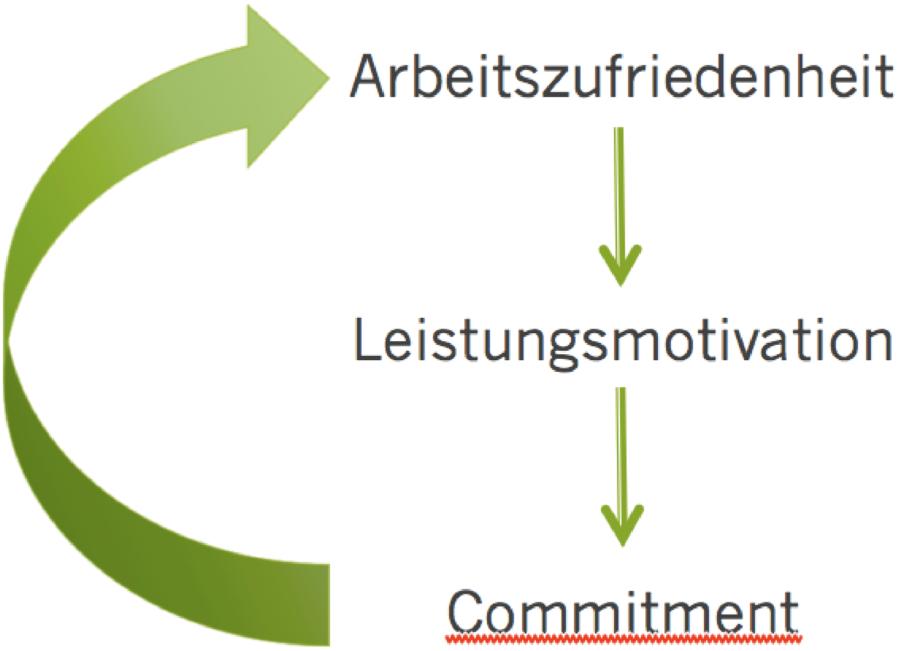

Abbildung 13. Darstellung der kausalen Feedbackschleife von Arbeitszufriedenheit- Leistungsmotivation- Commitment- Arbeitszufriedenheit.

7. Die Studie

In der Folge werden die Ergebnisse der Umfrage vorgestellt. Es wird eine Aufteilung entsprechend den einzelnen Fragekapiteln durchgeführt, wie sie im Fragebogen erfolgte: Demographie, psychische und körperliche Gesundheit, Arbeitsbelastung. Abschließend werden noch einige Korrelationen vorgestellt, die aus dem Vergleich von demographischen Parametern mit verschiedenen Fragen aus dem Kapitel Arbeitsbelastung berechnet wurden.

7.1. Ergebnisse

Eintausend hundert neunundvierzig (n=1.149) AnästhesistInnen waren zum Zeitpunkt der Umfragedurchführung als Mitglieder bei der ÖGARI eingeschrieben, insgesamt waren im August 2013 in Österreich bei der Ärztekammer Österreich 2.492 FachärztInnen für Anästhesie gemeldet (ÄRZTEKAMMER_ÖSTERREICH, 2013, 47). Fünf der angeschriebenen AnästhesistInnen erfüllten nicht die Einschlusskriterien, weil sie im Ausland arbeiteten (n=1), in Mutterschaft waren (n=2), in der Palliativmedizin (n=1) oder außerhalb des Krankenhauses arbeiteten (n=1). Deshalb erfüllten 1.145 ÖGARI Mitglieder die Einschlusskriterien; davon füllten 394 den kompletten Fragebogen aus. Daraus errechnet sich eine Antwortrate von 34,4%; die Originaldaten dieser Arbeit beruhen auf diesen Daten. Die Ergebnisse in Prozent wurden kaufmännisch auf eine Stelle hinter dem Komma gerundet.

7.2. Demographie

Das Alter der AnästhesistInnen war <30 Jahre in 2,5% (n=10), 31-40 Jahre in 38,3% (n=151), 41-50 Jahre in 27,2% (n=107), 51-60 Jahre in 28,2% (n=111) und >60 Jahre in 3,8% (n=15). Der Anteil männlicher Anästhesiten überwog leicht den Anteil weiblicher (52,3 vs. 47,8%; n=206 vs. n=187). Der Familienstand war allein lebend ohne Kinder in 14,45% (n=57), allein lebend mit Kindern in 7,1% (n=28), verheiratet ohne Kinder in 25,1% (n=99) und verheiratet mit Kindern in 53.1% (n=209). Die meisten AnästhesistInnen waren FachärztInnen (71,1%, n=280), während 28,7% (n=113) noch in Ausbildung zum Facharzt waren. Ein Drittel der AnästhesistInnen war in leitender Position tätig (32,7%, n=129), 66,5% (n=262) hatten keine leitende Funktion inne.

Am aktuellen Arbeitsplatz waren <3 Jahre 18,8% (n=74) tätig, während 22,3% (n=88) zwischen drei und fünf Jahren, 14,7% (n=58) sechs bis zehn Jahre und >10 Jahre 41,4% (n=163) tätig waren. Arbeitserfahrung in der Anästhesiologie (Fachbereich der die Anästhesie, Intensiv- und Notfallmedizin sowie die Schmerztherapie umfasst) hatten <3 Jahre 9,9% (n=39), drei bis fünf Jahre 15,7% (n=62), sechs bis zehn Jahre 18,5% (n=73) und >10 Jahre 54,8% (n=216). Die Zahl der jährlichen Narkosen (das heisst Allgemeinanästhesien bei denen der Patient während

der Operation in einen künstlichen Tiefschlaf versetzt wird) an der Klinik der AnästhesistInnen betrug <5.000 in 21.1% (n=83), 5.000-10.000 in 37,3% (n=147) und >10.000 in 39,6% (n=156). AnästhesistInnen waren der Ansicht, dass die Meinung der KollegInnen anderer Fachdisziplinen zu Ihrem Beruf sehr hoch ist in 3,4% (n=14), hoch ist in 31,7% (n=125), neutral ist in 35,3% (n=139), gering ist in 25,9% (n=102) und sehr gering ist in 3,3% (n=13). Das Ansehen welches AnästhesistInnen in der Allgemeinbevölkerung geniessen wurde angenommen als sehr hoch in 2.0% (n=8), hoch in 16,5% (n=65), neutral in 34,5% (n=136), gering in 37,8% (n=149) und sehr gering in 9,1% (n=36). Das Arbeitsklima der KollegInnen in der eigenen Abteilung wurde erachtet als sehr zufriedenstellend in 19,3% (n=76), zufriedenstellend in 50,0% (n=197), neutral in 11,2% (n=44), unbefriedigend in 16,8% (n=66) und ganz unbefriedigend in 2,5%.

7.3. Geistiges Wohlbefinden

Die folgenden Antworten waren zu geben auf die Aussage: Das war für mich in den letzten 12 Monaten eine Stressquelle.

Zu wenig Zeit für mich selbst

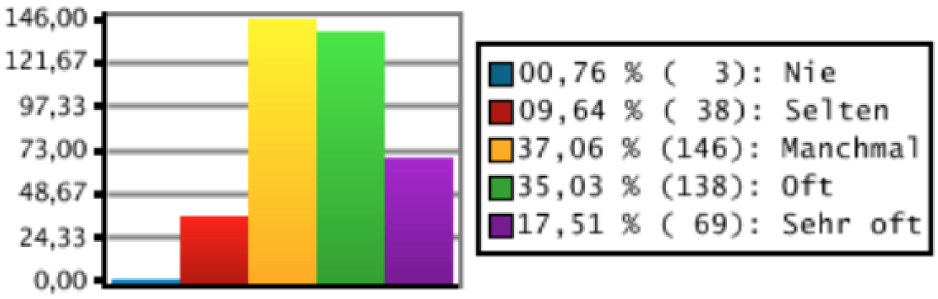

Zu wenig Zeit für Partner und/oder Kinder

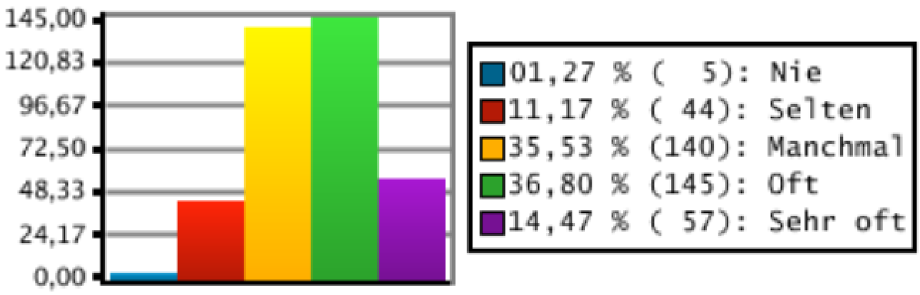

Zu wenig Zeit für Freunde

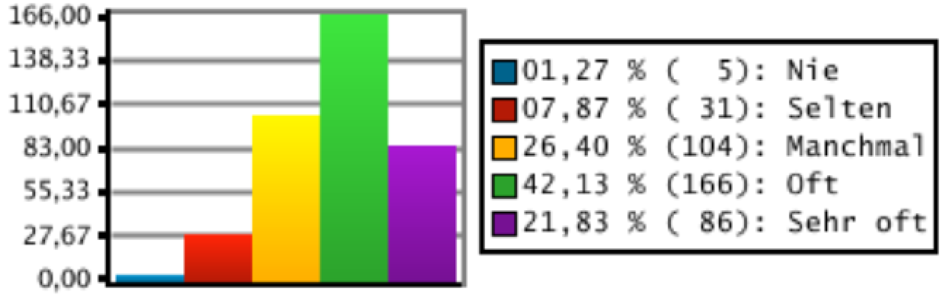

Zu wenig Zeit zum Essen

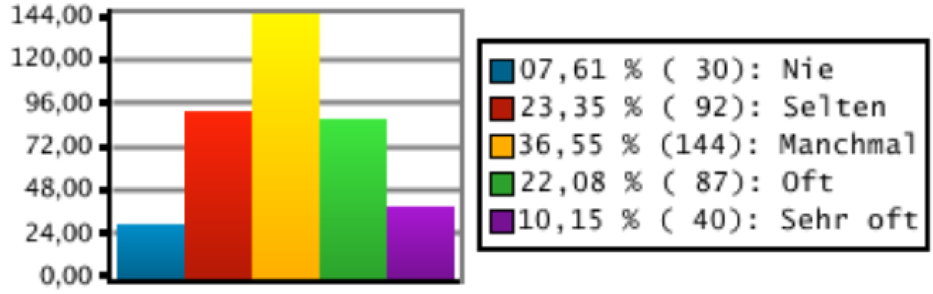

Zu lange Arbeitszeit

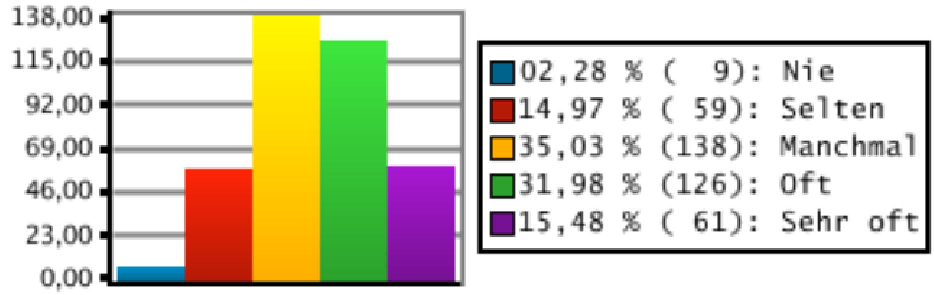

Die Zeit, die ich benötige um zur Arbeitsstätte zu gelangen

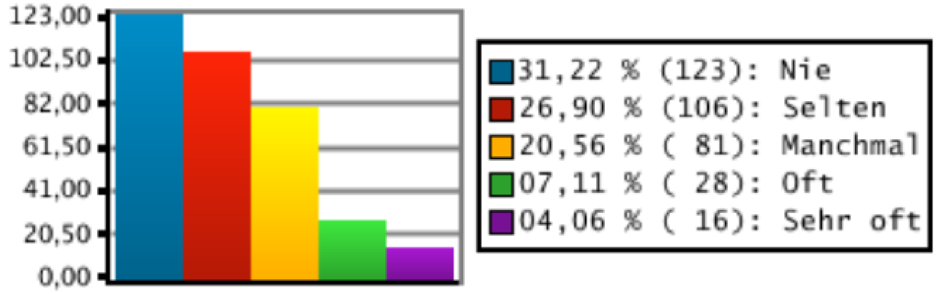

Zu wenig Zeit zum Ausruhen/Schlafen

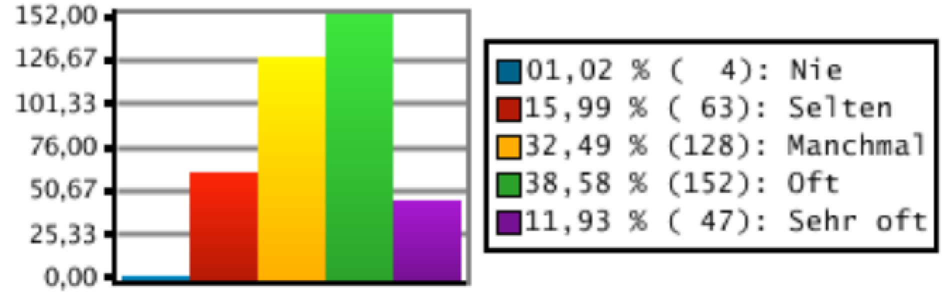

7.4. Körperliches Wohlbefinden

Kopfschmerzen

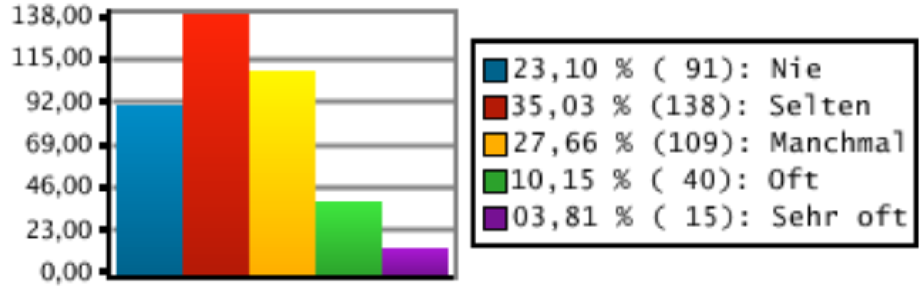

Schwierigkeiten ein- oder durchzuschlafen

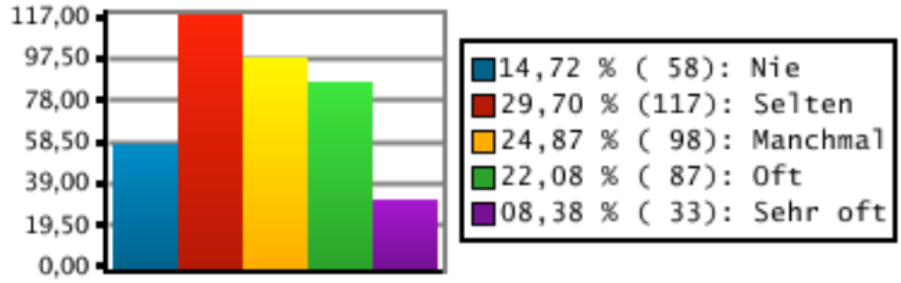

Muskelverspannung im Rücken oder Hals

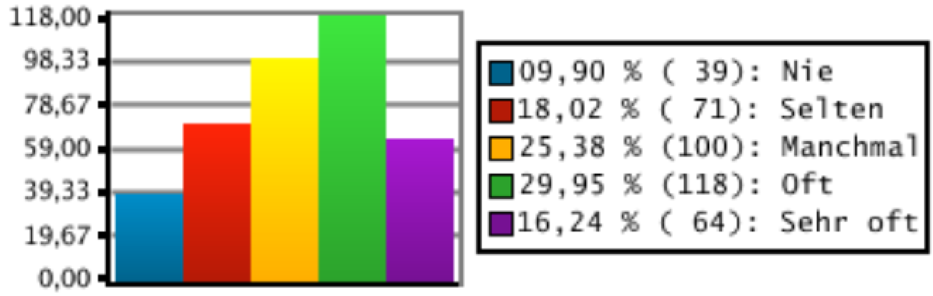

Magenbeschwerden

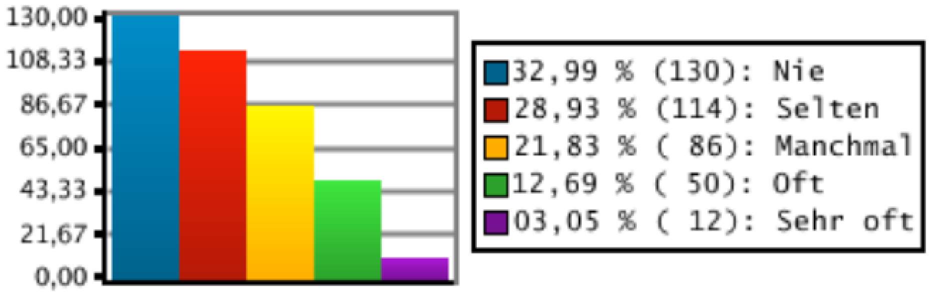

Kurzer Atem, auch wenn Sie sich nicht körperlich anstrengen

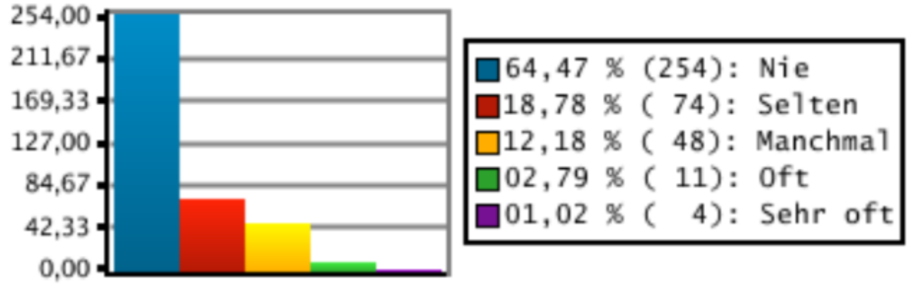

Hautausschlag oder Juckreiz

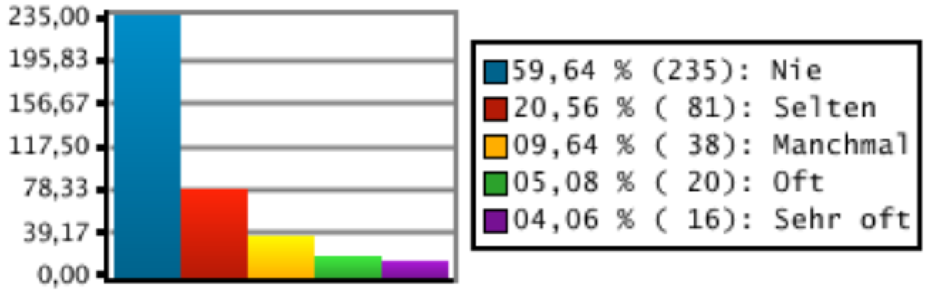

Diarrhöe

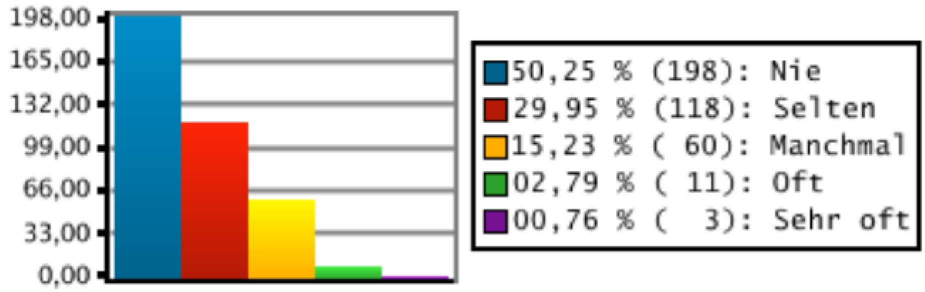

Mattigkeit und Schwindelgefühle

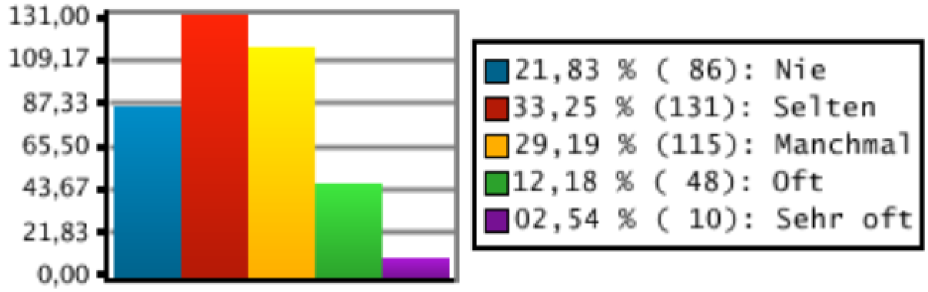

Schwierigkeiten, sich an Dinge zu erinnern

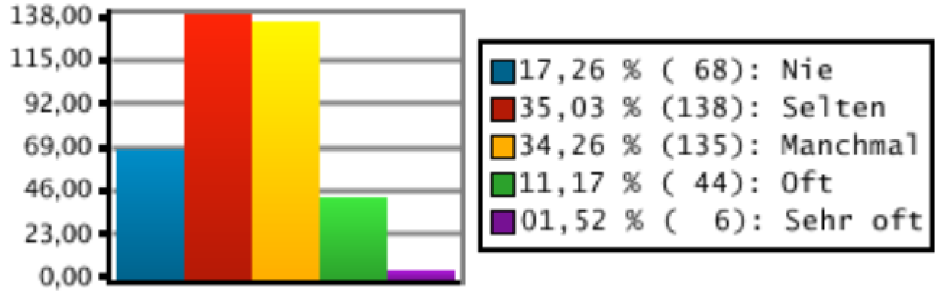

Herzrasen oder starkes Herzschlagen

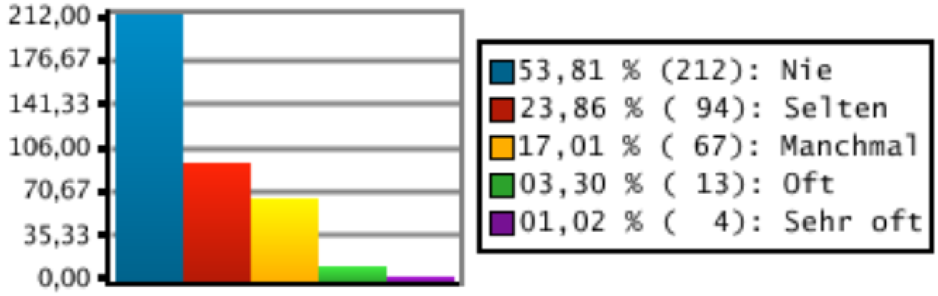

Schmerzen in der Brust

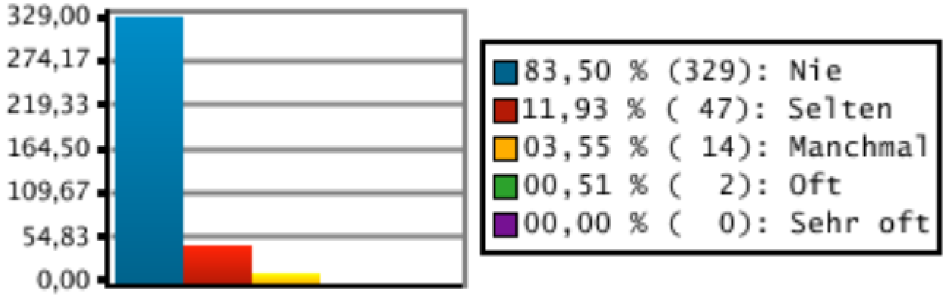

Leichtes Ermüden

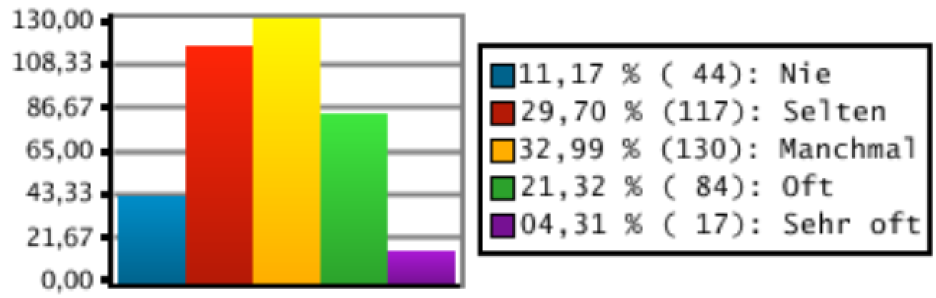

Verstopfung

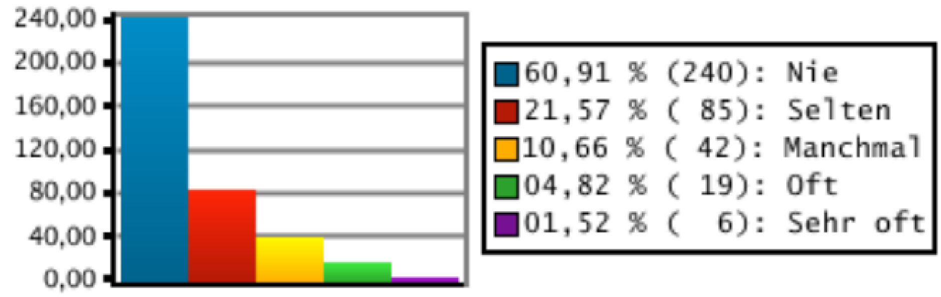

Trockener Mund oder Probleme beim Schlucken

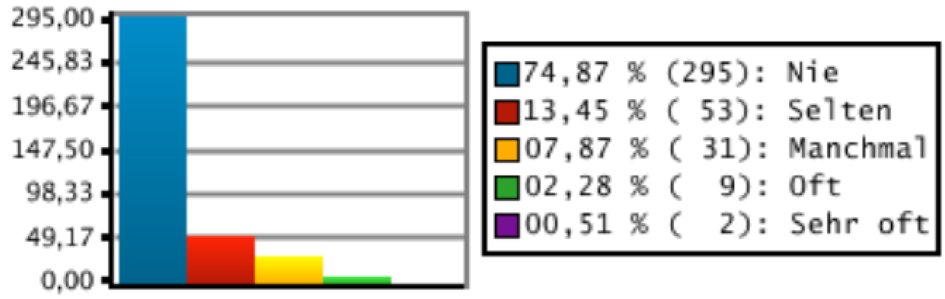

Nachlassen von sexuellem Interesse oder sexuellem Vergnügen

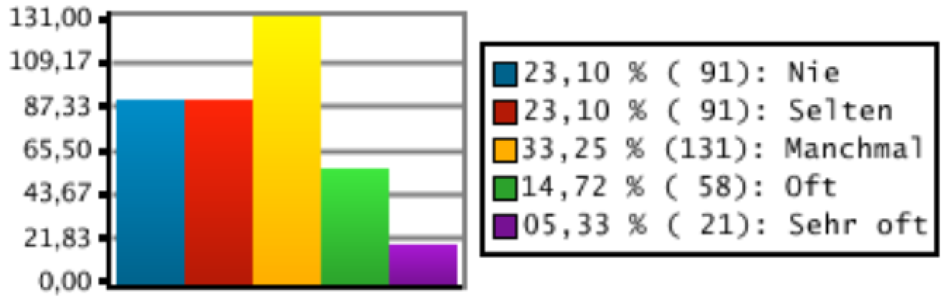

Appetitmangel

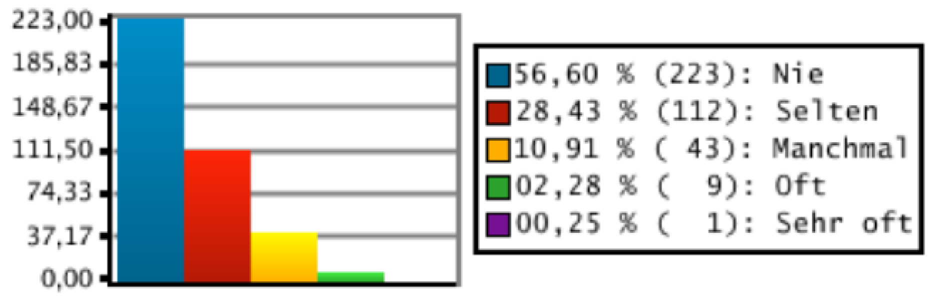

Rückenschmerzen

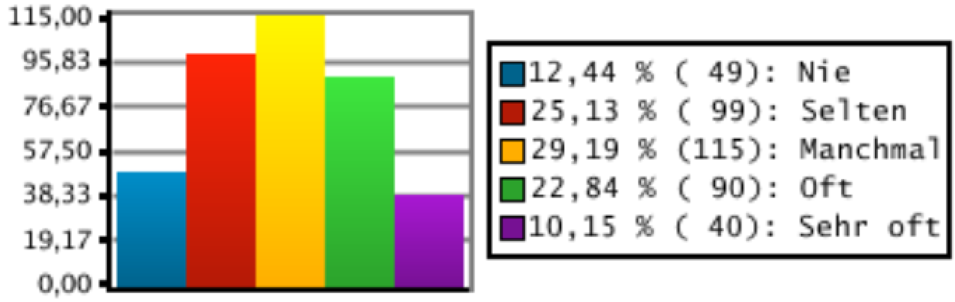

Die folgenden Fragen bezogen sich auf den Umgang von AnästhesistInnen mit Gefühlen und Problemen. Die Antworten „nie, selten, manchmal, oft, sehr oft“ beziehen sich auf die Häufigkeit Ihrer Tätigkeiten bzw. Ihres Fühlens. Die Antworten sollten spontan gegeben werden.

Wie oft genießen Sie das Leben und das, was Sie tun

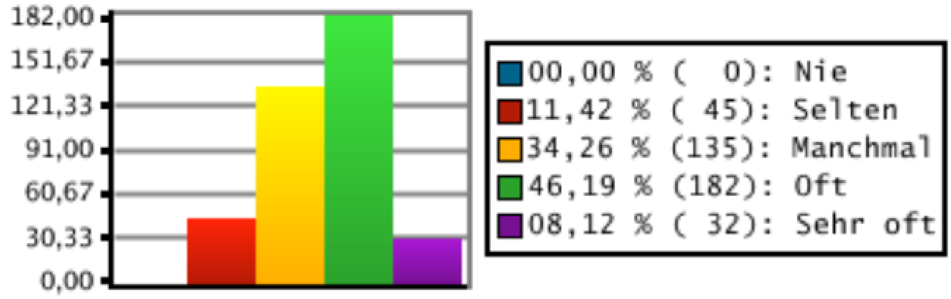

Wie oft gehen Sie mit neuen oder anderen Lösungen an Probleme heran

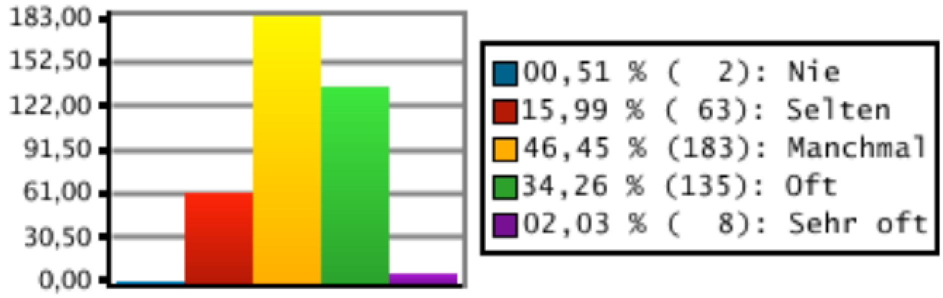

Wie oft fühlen Sie sich schlecht gelaunt oder angespannt wegen irgendwelcher Angelegenheiten?

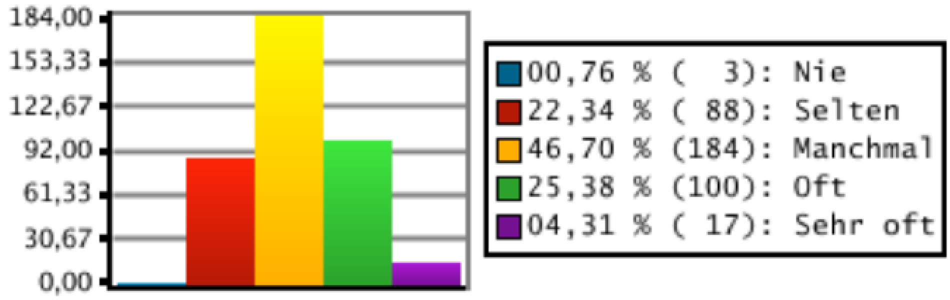

Wie oft unternehmen Sie etwas, um Dinge zum Positiven zu verwandeln?

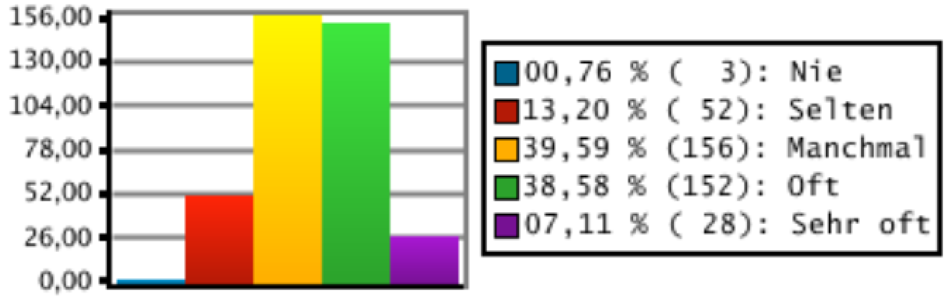

Wie oft finden Sie es schwierig, mit anderen über Ihre Gefühle zu sprechen? Aufpassen, dass in der Endfassung die Graphik nicht auf de4r nächsten Seite erscheint.

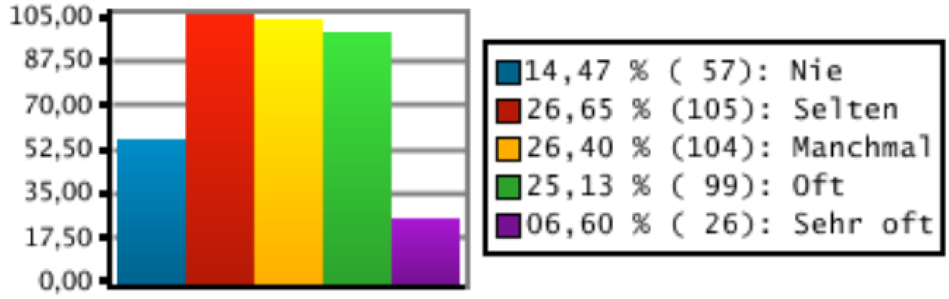

Wie oft geben Sie den Menschen, die Ihnen nahe stehen, zu erkennen, wenn Sie sich traurig oder niedergeschlagen fühlen?

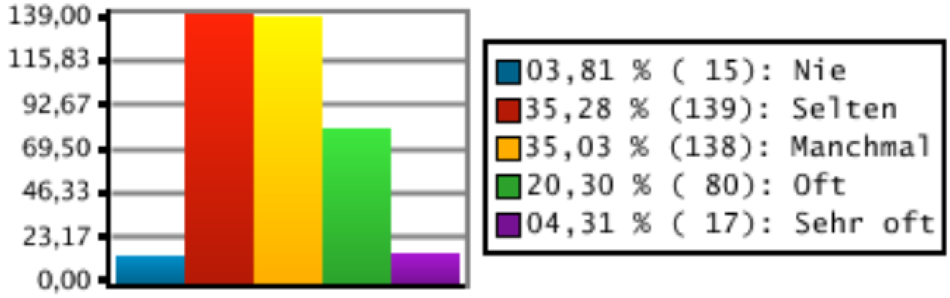

Wie oft haben Sie Schwierigkeiten, sich wieder zu beruhigen, wenn Sie sich über etwas aufgeregt haben?

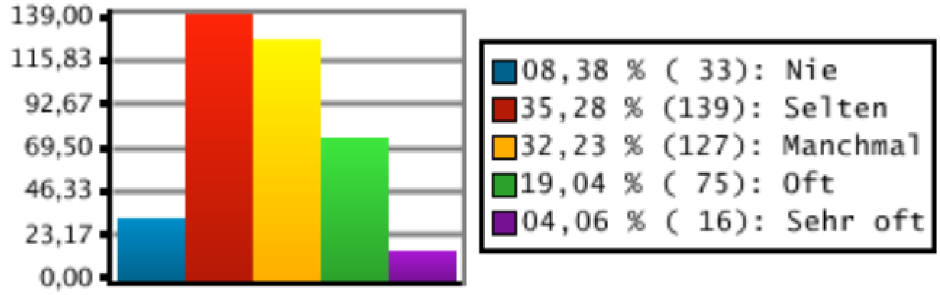

Wie oft versuchen Sie, einem Problem auf den Grund zu gehen und Veränderungen herbeizuführen?

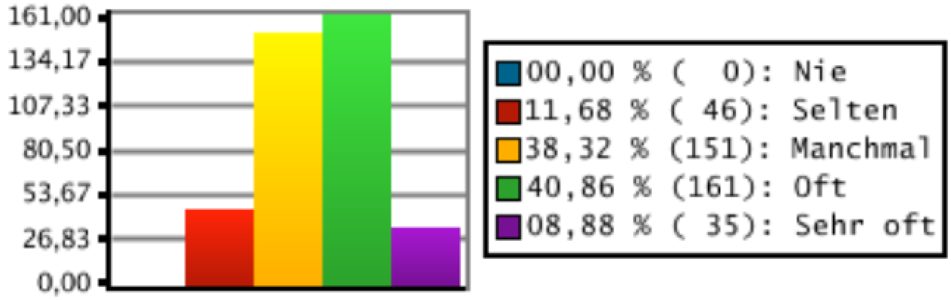

Wie oft behalten Sie Ihre Gefühle für sich?

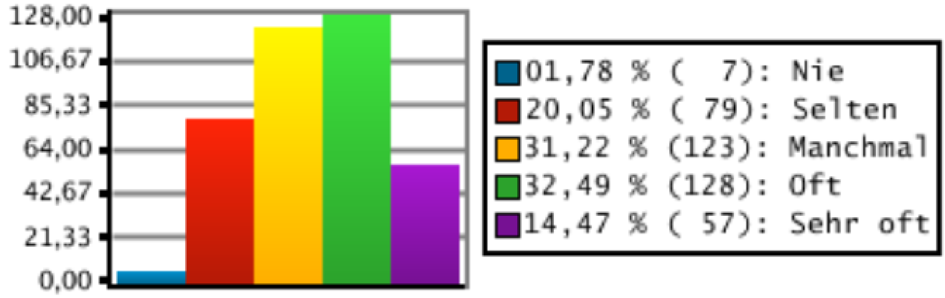

Wie oft fühlen Sie sich nervös oder beunruhigt?

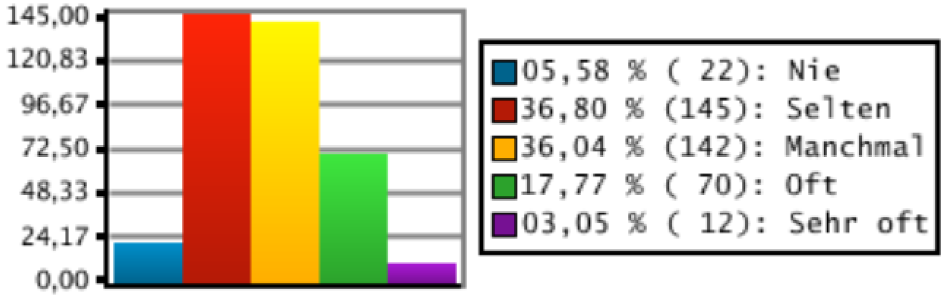

Wie oft haben Sie eine gedrückte Stimmung?

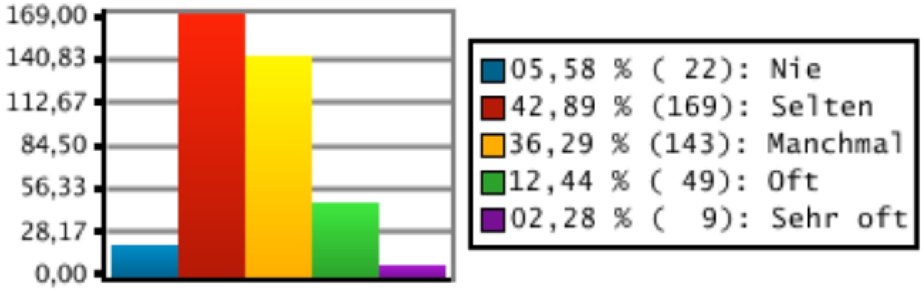

Wie oft erzählen Sie anderen, wenn Sie sich frustriert oder enttäuscht fühlen?

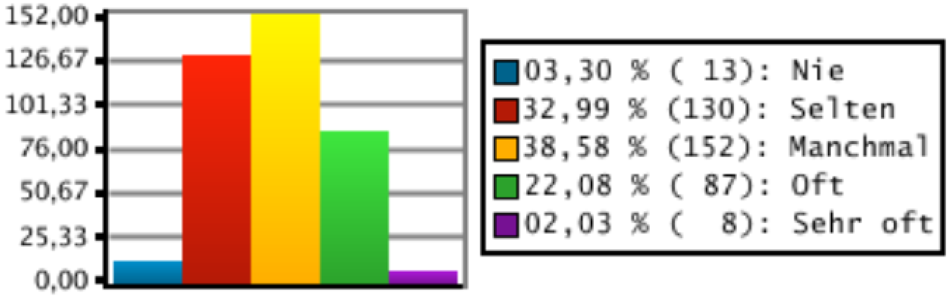

Wie oft äußern Sie, wenn Sie von anderen etwas möchten?

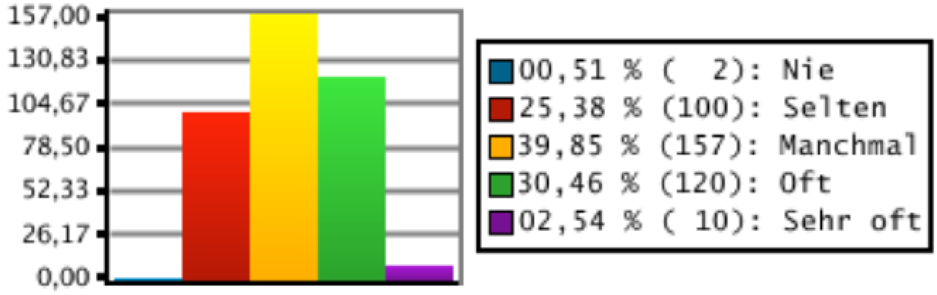

Wie oft fühlen sie sich ganz entspannt?

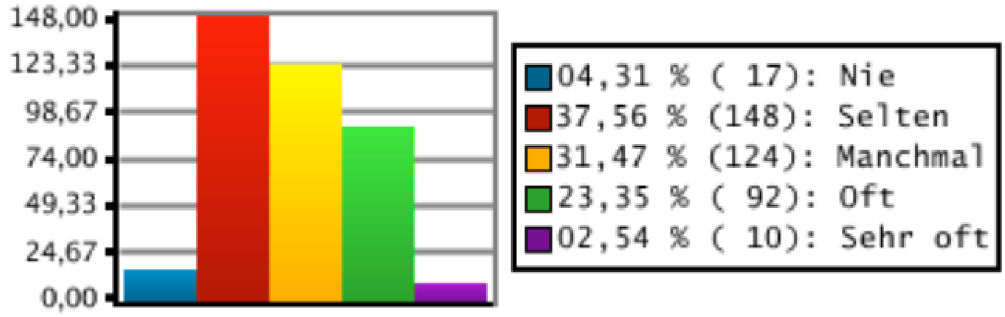

Wie oft fühlen Sie sich ruhelos und ungeduldig?

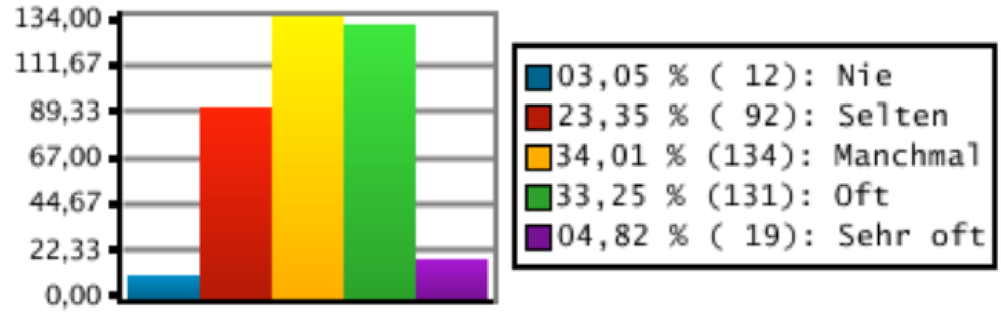

Wie oft lassen Sie anderen wissen, wenn Sie Angst haben oder nervös sind?

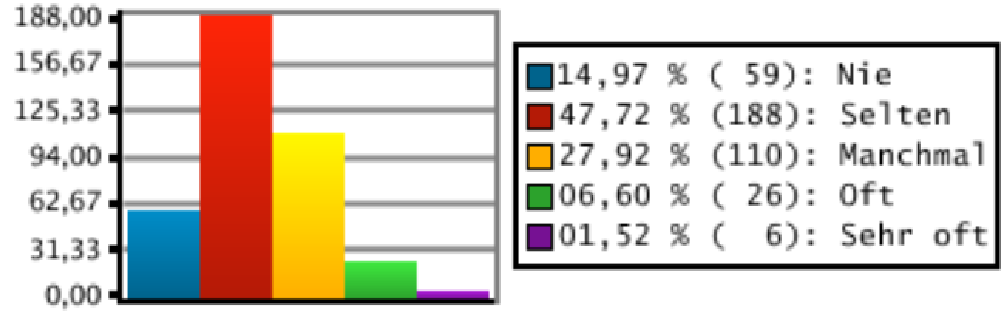

Wie oft haben Sie beim Aufstehen das Gefühl, dass es ein guter Tag wird?

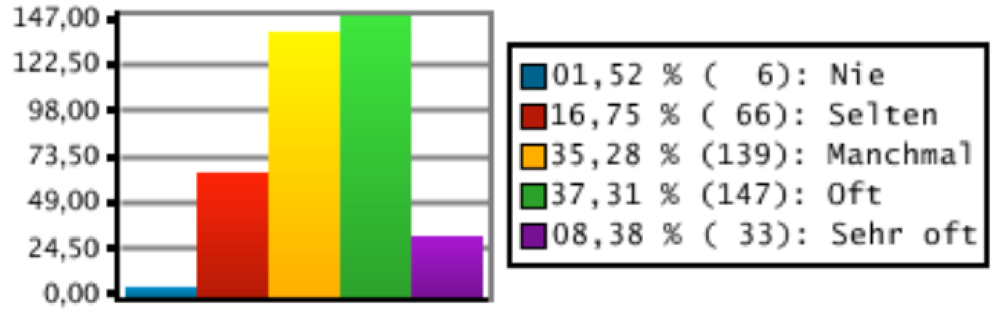

Wie oft umgehen Sie es Entscheidungen zu treffen, in der Hoffnung, dass sich die Situation von alleine ändern wird?

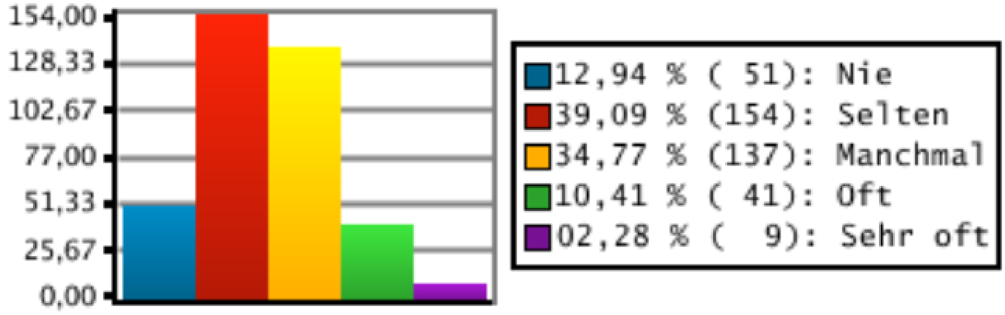

Wie oft fällt es Ihnen leicht, Ihre Gedanken anderen mitzuteilen?

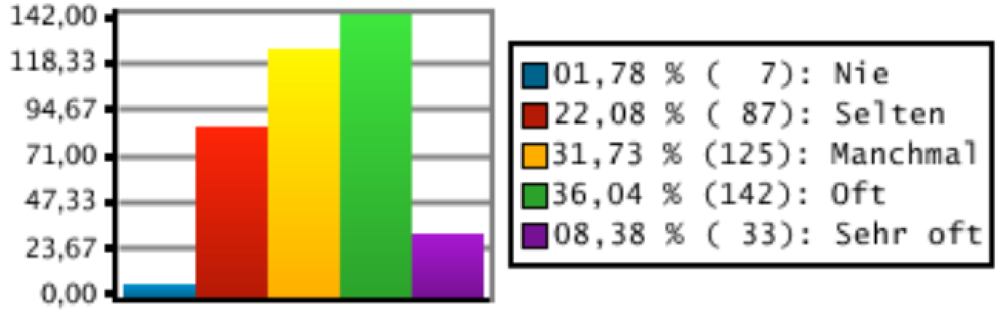

Wie oft fühlen Sie sich traurig und niedergeschlagen?

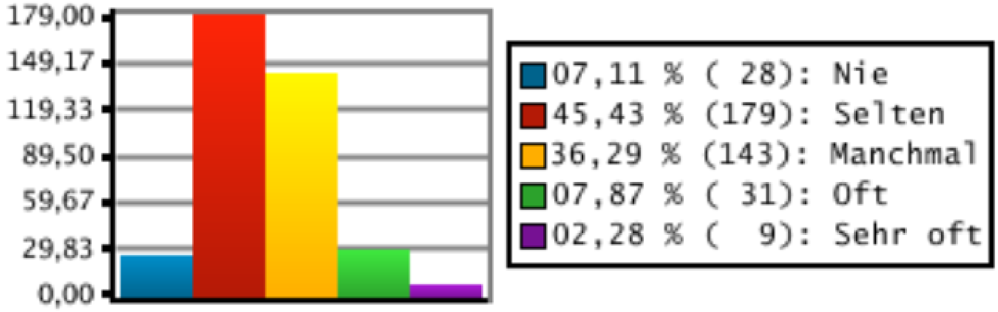

Wie oft konzentrieren Sie sich auf das, was Sie tun müssen, und arbeiten umso härter daran?

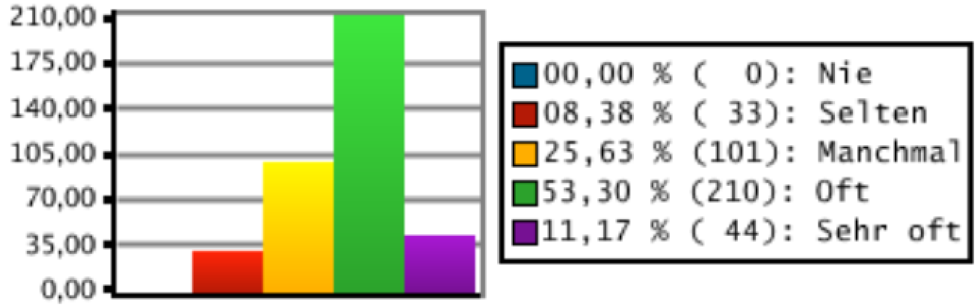

7.5. Instrument der Stress Tätigkeits Analyse

Im Folgenden wurde eine Reihe von Fragen zur Arbeit gestellt. Dabei ging es um die Arbeitsbedingungen und nicht darum, wie gut oder wie schlecht AnästhesistInnen persönlich die Arbeit verrichteten. Die Befragten waren gefordert sich vorzustellen, dass ein/e Kollege/in mit dem gleichen Wissen und Können die Fragen genauso beantworten musste.

Kollege/in A muss bei seiner/ihrer Arbeit sehr komplizierte Entscheidungen treffen. Kollege/in B muss bei seiner/ihrer Arbeit nur sehr einfache Entscheidungen treffen.

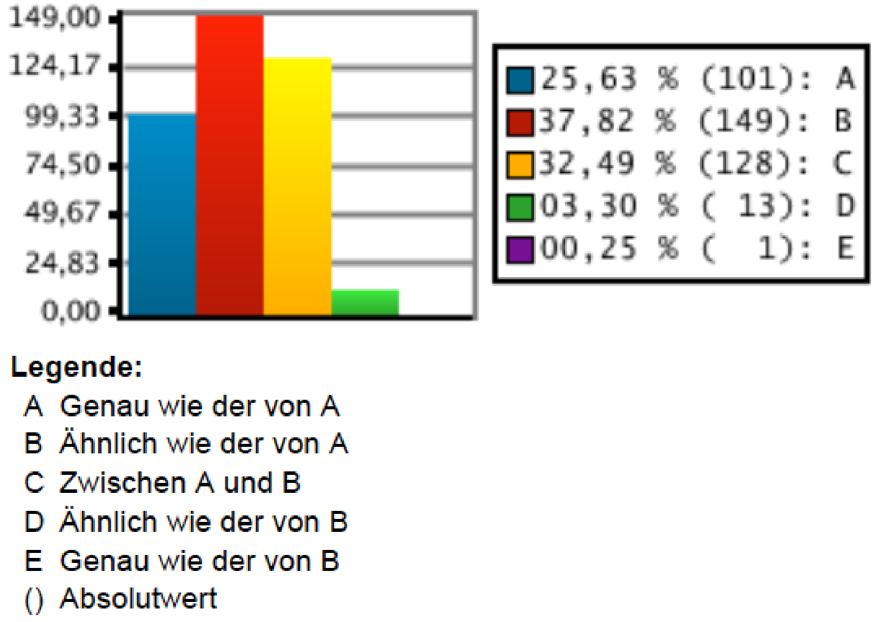

Wie oft erhalten Sie Aufträge, die besonders schwierig sind?

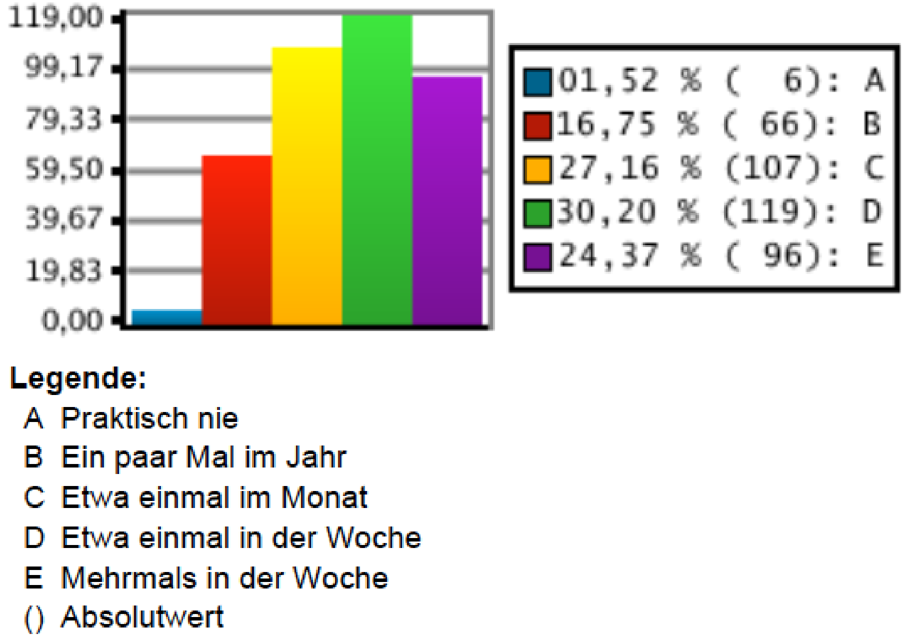

Können Sie bei Ihrer Arbeit Neues dazulernen?

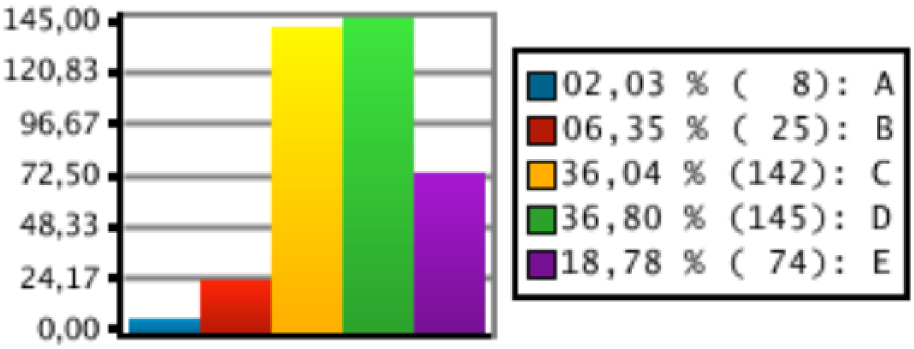

Legende:

A Sehr wenig
B Ziemlich wenig
C Etwas
D Ziemlich viel
E Sehr viel
() Absolutwert

Kollege/in A bearbeitet Aufgaben, bei der er oder sie genau überlegen muss, was im Einzelnen zu tun ist. Kollege/in B bearbeitet Aufgaben, bei denen sofort klar ist, was zu tun ist. Welcher der zwei Arbeitsplätze ist Ihrem Arbeitsplatz am ähnlichsten?

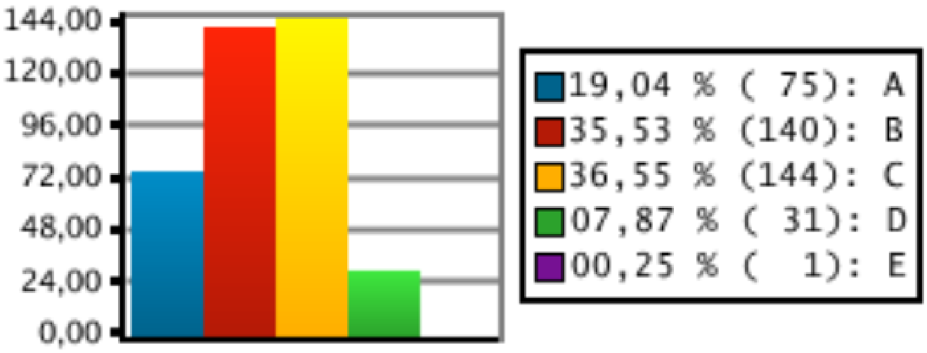

Legende:

A Genau wie der von A
B Ähnlich wie der von A
C Zwischen A und B
D Ähnlich wie der von B
E Genau wie der von B
() Absolutwert

Kollege/in A bearbeitet Aufgaben, bei der er oder sie zuerst genau planen muss, um die Aufgaben ausführen zu können. Kollege/in B bearbeitet Aufgaben, bei denen keine Planung erforderlich ist. Welcher der zwei Arbeitsplätze ist Ihrem Arbeitsplatz am ähnlichsten?

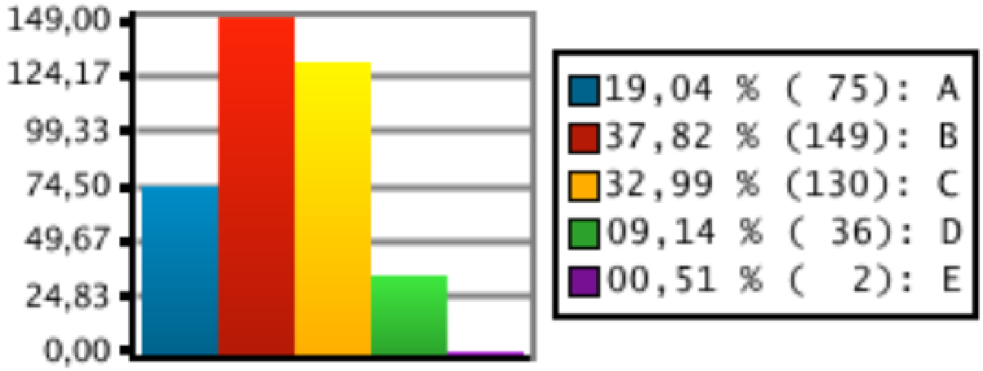

Legende:
A Genau wie der von A
B Ähnlich wie der von A
C Zwischen A und B
D Ähnlich wie der von B
E Genau wie der von B
() Absolutwert

Wenn Sie Ihre Tätigkeit insgesamt betrachten, inwieweit können Sie die Reihenfolge der Arbeitsschritte selbst festlegen?

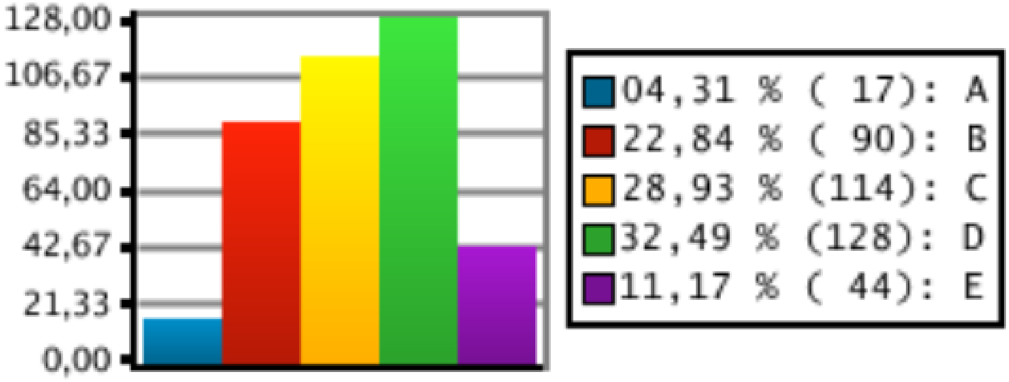

Legende:
A Sehr wenig
B Ziemlich wenig
C Etwas
D Ziemlich viel
E Sehr viel
() Absolutwert

Wie viel Einfluss haben Sie darauf, welche Arbeit Ihnen zugeteilt wird?

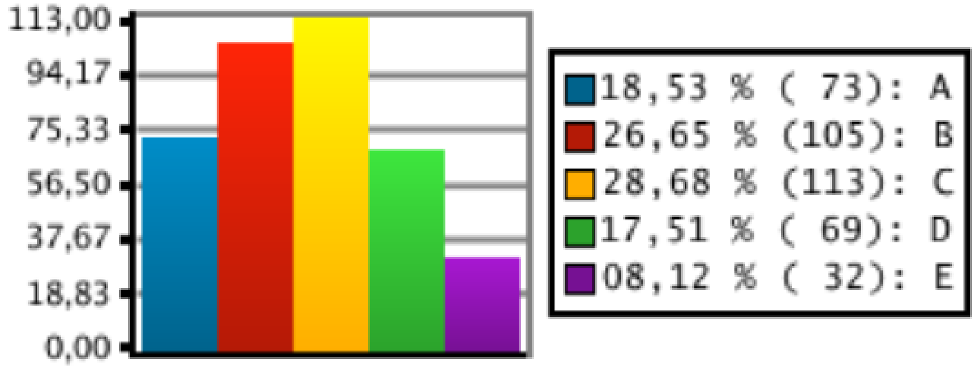

Legende:

A Sehr wenig
B Ziemlich wenig
C Etwas
D Ziemlich viel
E Sehr viel
() Absolutwert

Wenn man Ihre Arbeit insgesamt betrachtet, wie viele Möglichkeiten zu eigenen Entscheidungen bietet Ihnen Ihre Arbeit?

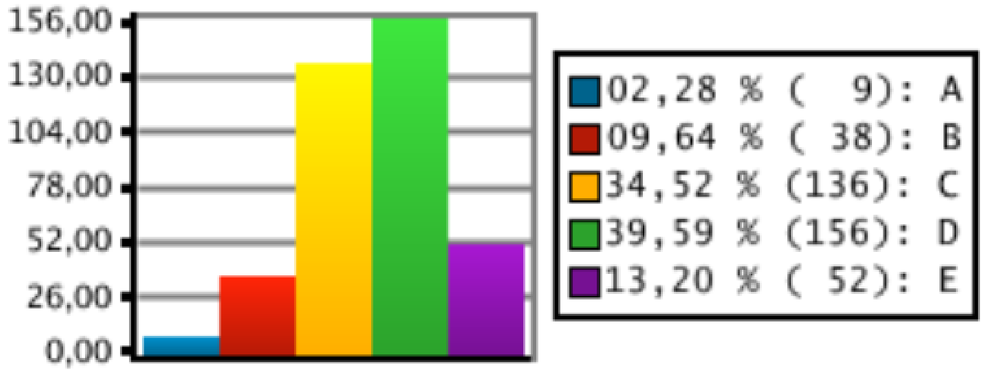

Legende:

A Sehr wenig
B Ziemlich wenig
C Etwas
D Ziemlich viel
E Sehr viel
() Absolutwert

Können Sie selbst bestimmen, auf welche Art und Weise Sie Ihre Arbeit erledigen?

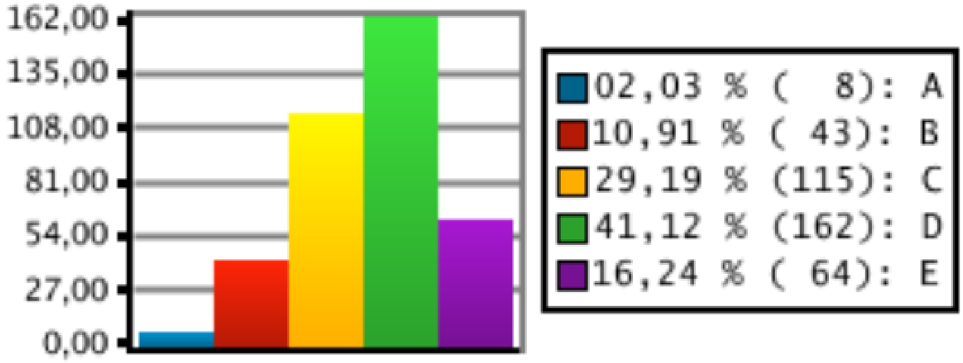

Legende:
A Sehr wenig
B Ziemlich wenig
C Etwas
D Ziemlich viel
E Sehr viel
() Absolutwert

Inwieweit sind ausschließlich Sie selbst für die Kontrolle Ihres Arbeitsergebnisses zuständig?

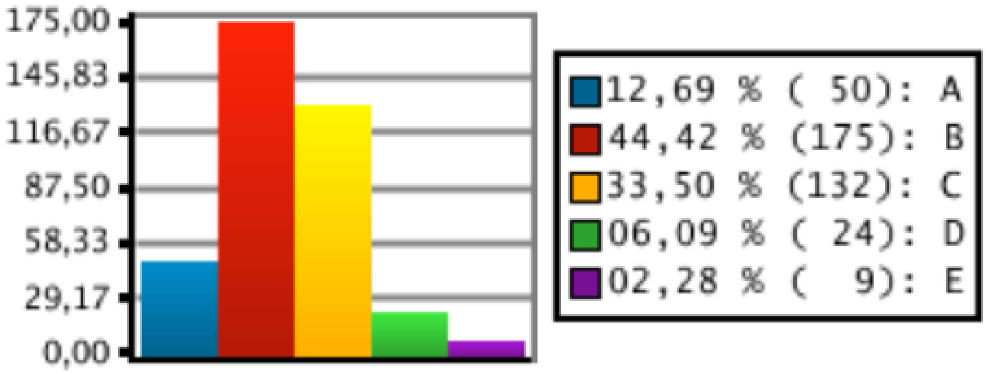

Legende:
A Von allen Arbeitsergebnissen
B Von den meisten Arbeitsergebnissen
C Von einem Teil der Arbeitsergebnisse
D Von wenigen Arbeitsergebnissen
E Gar nicht
() Absolutwert

A hat Arbeitsaufgaben, die sich häufig wiederholen. B hat viele verschiedene Arbeitsaufgaben. Welcher der beiden Arbeitsplätze ist Ihrem Arbeitsplatz am ähnlichsten?

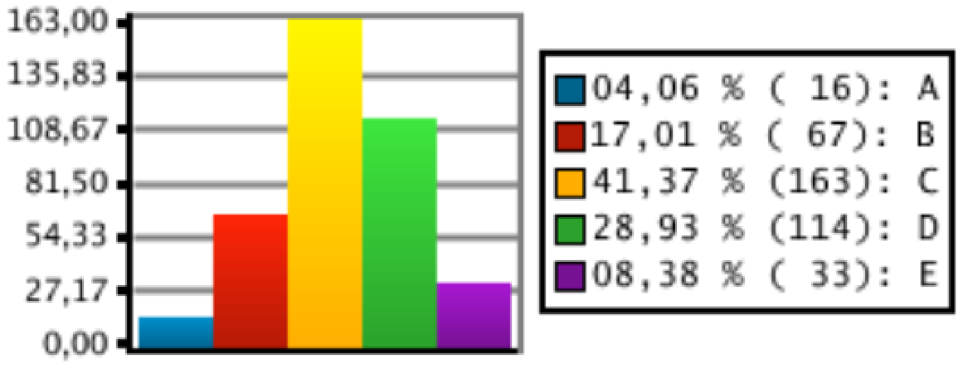

Legende:

A Genau wie der von A
B Ähnlich wie der von A
C Zwischen A und B
D Ähnlich wie der von B
E Genau wie der von B
() Absolutwert

A muss bei seiner/ihrer Arbeit mit vielen verschiedenen Arbeitsmitteln hantieren. B kommt mit sehr wenigen Arbeitsmitteln aus. Welcher der beiden Arbeitsplätze ist Ihrem Arbeitsplatz am ähnlichsten? Keine Unterstreichungen durchgängig in der ganzen Arbeit

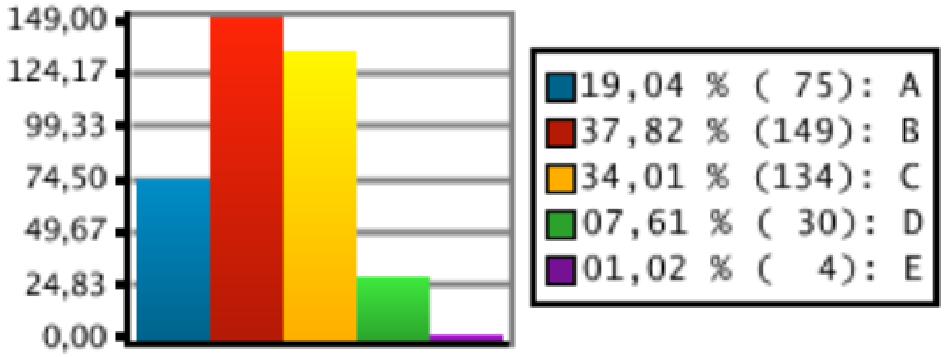

Legende:

A Genau wie der von A
B Ähnlich wie der von A
C Zwischen A und B
D Ähnlich wie der von B
E Genau wie der von B
() Absolutwert

Im Folgenden wollen wir wissen, wie viel Einfluss Sie auf Ihre Arbeitssituation haben.

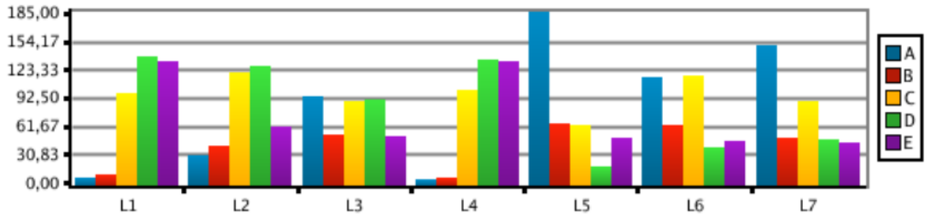

Legende:

A Ich habe keinerlei Einfluss-möglichkeiten
B Ich werde nur informiert
C Ich kann Vorschläge machen
D Ich bin bei der Entscheidung beteiligt
E Ich habe großen Einfluss auf die Entscheidung
L1 Bei der Aufstellung der Urlaubspläne
L2 Bei der Planung der Arbeitszeit (Schichten und Überstunden)
L3 Auf die Pausengestaltung
L4 Auf die Möglichkeiten zur Weiterbildung
L5 Bei der Einstellung neuer KollegInnen
L6 Bei der Anschaffung neuer Geräte
L7 Auf die Ausgestaltung meines Arbeitsplatzes (z.B. Einrichtung des Arbeitszimmers)
() Absolutwert

	A	B	C	D	E
**L1**	2,28 % (9)	3,30 % (13)	25,13 % (99)	35,28 % (139)	33,76 % (133)
**L2**	8,38 % (33)	11,17 % (44)	30,96 % (122)	32,49 % (128)	15,99 % (63)
**L3**	24,37 % (96)	14,21 % (56)	23,10 % (91)	23,60 % (93)	13,71 % (54)
**L4**	1,78 % (7)	2,54 % (10)	25,89 % (102)	34,26 % (135)	33,76 % (133)
**L5**	46,95 % (185)	17,01 % (67)	16,50 % (65)	5,33 % (21)	13,20 % (52)
**L6**	29,44 % (116)	16,50 % (65)	29,95 % (118)	10,66 % (42)	12,44 % (49)
**L7**	38,07 % (150)	13,20 % (52)	22,84 % (90)	12,69 % (50)	11,68 % (46)

Die Arbeit von A erfordert nur zwei bis drei verschiedene Handgriffe, die immer wiederkommen. Die Arbeit von B umfasst viele verschiedene Handgriffe. Welcher der beiden Arbeitsplätze ist Ihrem Arbeitsplatz am ähnlichsten?

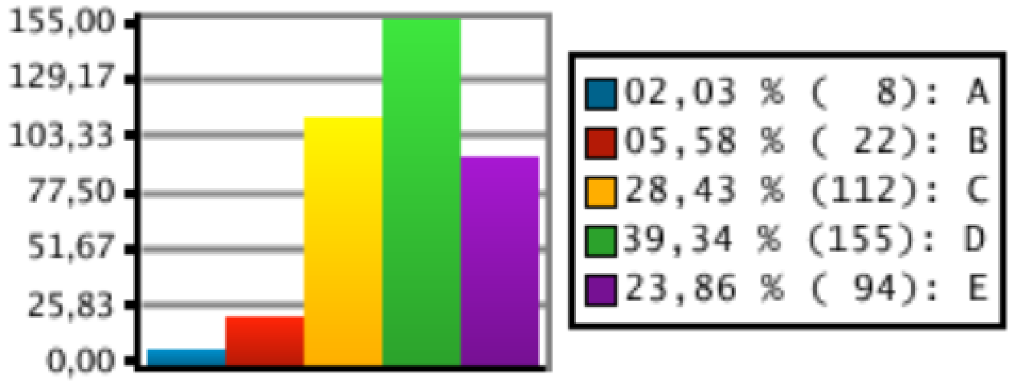

Legende:

A Genau wie der von A
B Ähnlich wie der von A
C Zwischen A und B
D Ähnlich wie der von B
E Genau wie der von B
() Absolutwert

A muss sehr unterschiedliche Aufgaben erfüllen. B erfüllt sehr ähnliche Aufgaben. Welcher der beiden Arbeitsplätze ist Ihrem Arbeitsplatz am ähnlichsten

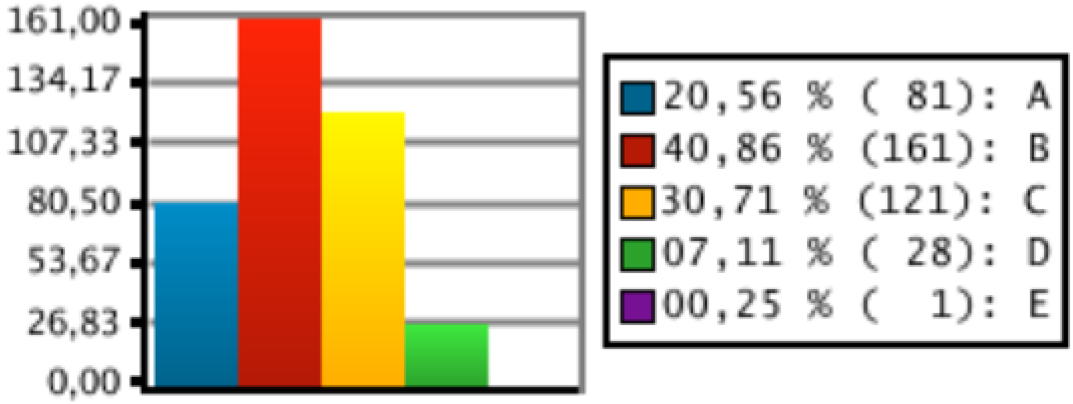

Legende:
A Genau wie der von A
B Ähnlich wie der von A
C Zwischen A und B
D Ähnlich wie der von B
E Genau wie der von B
() Absolutwert

A hat in seiner/ihrer Arbeit sehr viele Routineaufgaben zu tun. In der Arbeit von B wiederholen sich die Aufgaben nur wenig. Welcher der beiden Arbeitsplätze ist Ihrem Arbeitsplatz am ähnlichsten?

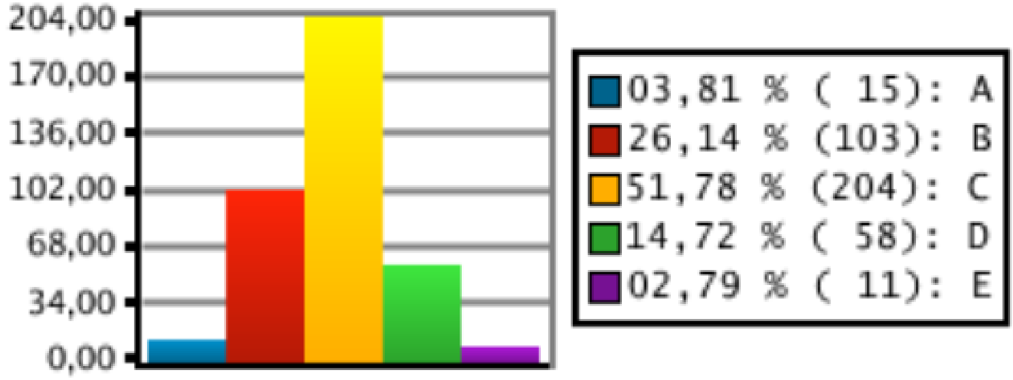

Legende:
A Genau wie der von A
B Ähnlich wie der von A
C Zwischen A und B
D Ähnlich wie der von B
E Genau wie der von B
() Absolutwert

Wie lange können Sie während der Arbeitszeit Ihren Arbeitsplatz verlassen?

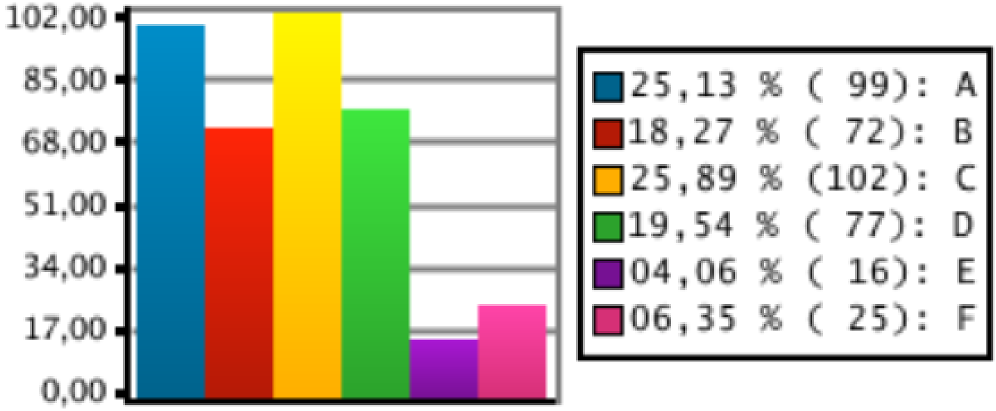

Legende:

A Gar nicht

B Bis zu 5 Minuten

C Mehr als 5, bis 15 Minuten

D Mehr als 15, bis zu 30 Minuten

E Mehr als 30 Minuten, bis zu 1 Stunde

F Mehr als 1 Stunde

() Absolutwert

Wie sehr können Sie Ihre Arbeitsgeschwindigkeit selbst bestimmen?

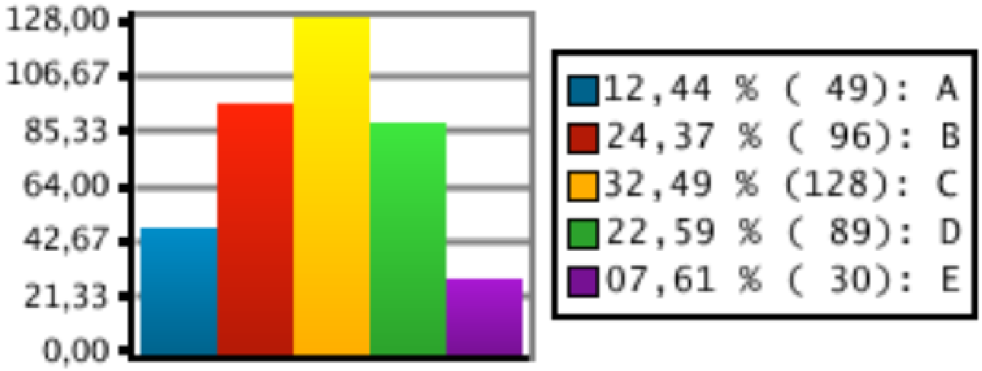

Legende:

A Sehr wenig

B Ziemlich wenig

C Etwas

D Ziemlich viel

E Sehr viel

() Absolutwert

Wie lange können Sie sich von Ihrer Arbeit abwenden und irgendetwas anderes dazwischen schieben (zum Beispiel eine Pause), ohne mit Ihrer eigentlichen Arbeitsaufgabe in Verzug zu geraten?

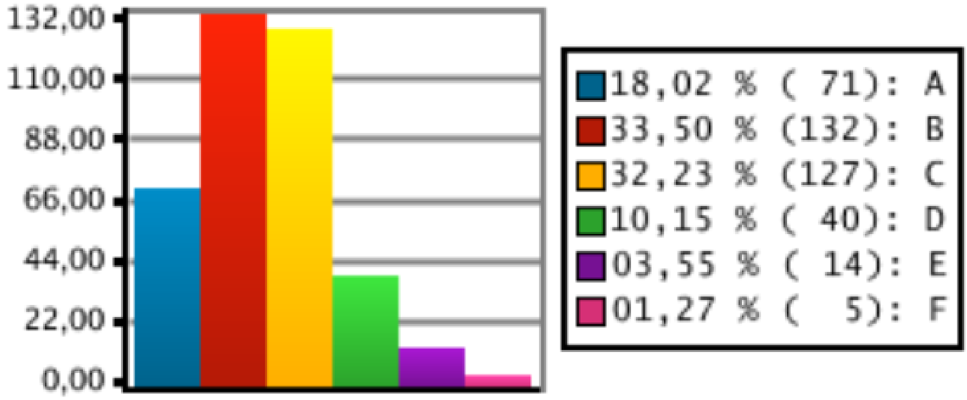

Legende:

A Weniger als 5 Minuten am Tag
B Mehr als 5, bis zu 10 Minuten am Tag
C Mehr als 15, bis zu 30 Minuten am Tag
D Mehr als 30 Minuten, bis zu 1 Stunde am Tag
E Mehr als 1 Stunde, bis zu 2 Stunden am Tag
F Mehr als 2 Stunden am Tag
() Absolutwert

In wieweit können Sie selbst bestimmen, wie lange Sie an einer Sache arbeiten?

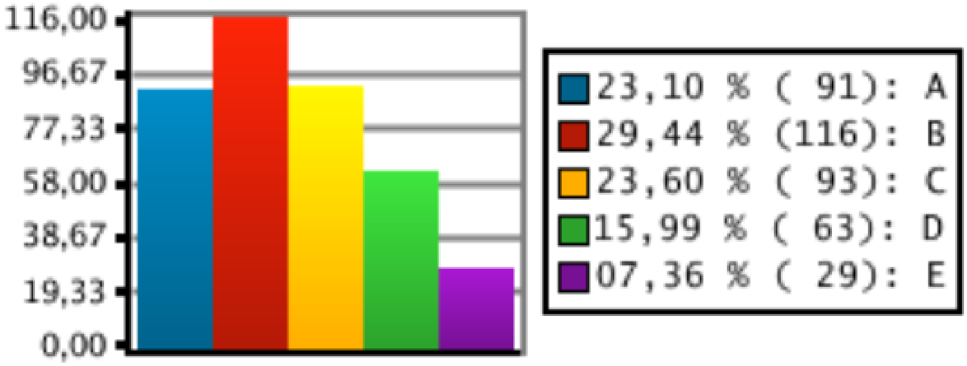

Legende:

A Sehr wenig
B Ziemlich wenig
C Etwas
D Ziemlich viel
E Sehr viel
() Absolutwert

Können Sie Ihren Arbeitstag selbständig einteilen?

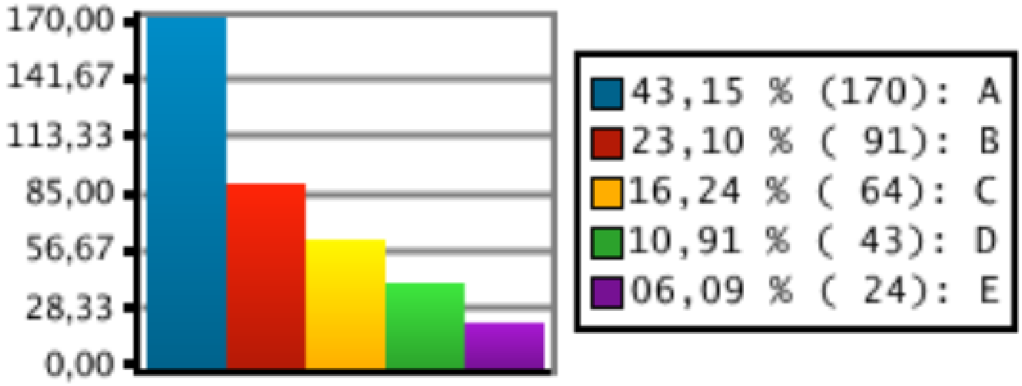

Legende:

A Sehr wenig
B Ziemlich wenig
C Etwas
D Ziemlich viel
E Sehr viel
() Absolutwert

Von wie vielen Leuten erhalten Sie regelmäßig Anweisungen?

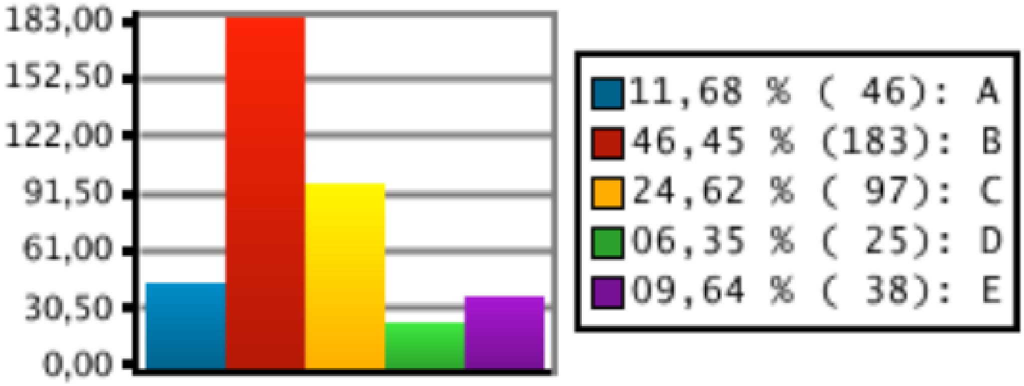

Legende:

A Von keinem Vorgesetzten
B Von einem Vorgesetzten
C Von zwei Vorgesetzten
D Von drei Vorgesetzten
E Von mehr als drei Vorgesetzten

()Absolutwert

Wie oft erhalten Sie unklare Anweisungen?

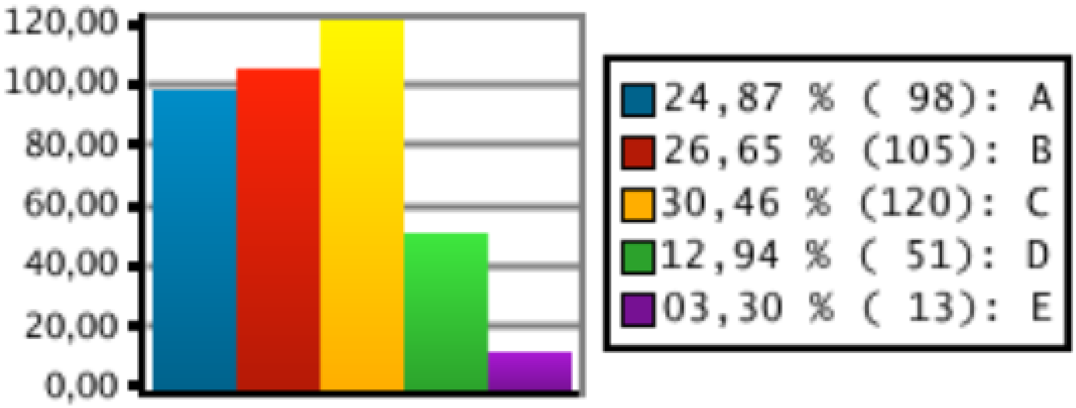

Legende:

A Sehr selten/nie
B Selten (etwa 1 x pro Monat)
C Gelegentlich (etwa 1 x pro Woche)
D Oft (mehrmals pro Woche)
E Sehr oft (ein bis mehrmals täglich)
() Absolutwert

Wie oft erhalten Sie von verschiedenen Vorgesetzten widersprüchliche Anweisungen?

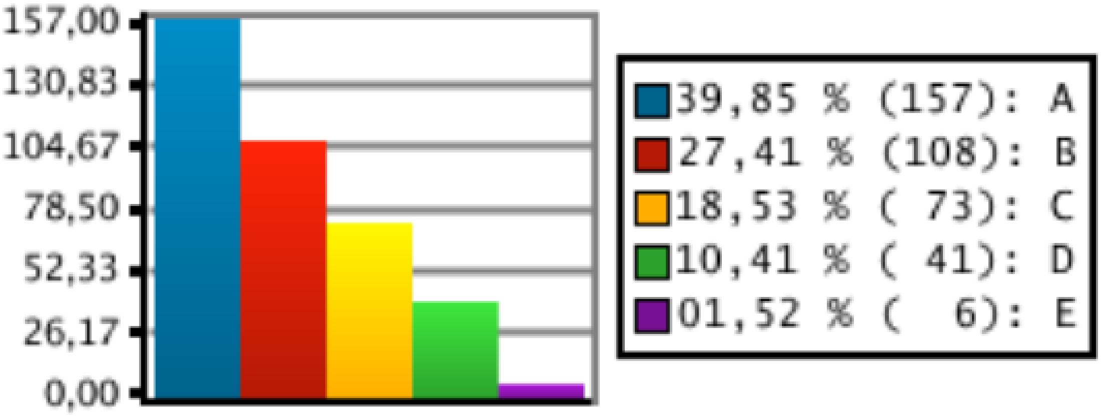

Legende:

A Sehr selten/nie
B Selten (etwa 1 x pro Monat)
C Gelegentlich (etwa 1 x pro Woche)
D Oft (mehrmals pro Woche)
E Sehr oft (ein bis mehrmals täglich)
() Absolutwert

Wie oft kommt es vor, dass Sie bei Ihrer Arbeit Entscheidungen fällen müssen, ohne dass ausreichende Informationen zur Verfügung stehen?

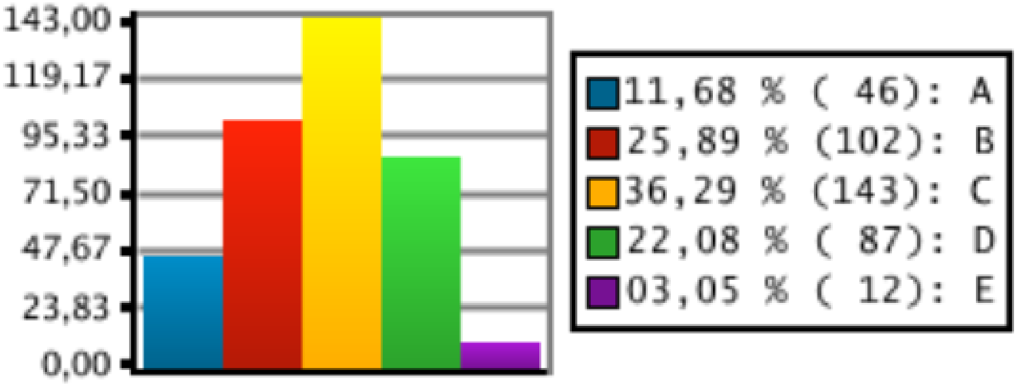

Legende:

A Sehr selten/nie
B Selten (etwa 1 x pro Monat)
C Gelegentlich (etwa 1 x pro Woche)
D Oft (mehrmals pro Woche)
E Sehr oft (ein bis mehrmals täglich)
() Absolutwert

Manchmal kann man nicht weiterarbeiten, weil woanders ein Problem entsteht. Wie oft tritt das bei Ihnen auf?

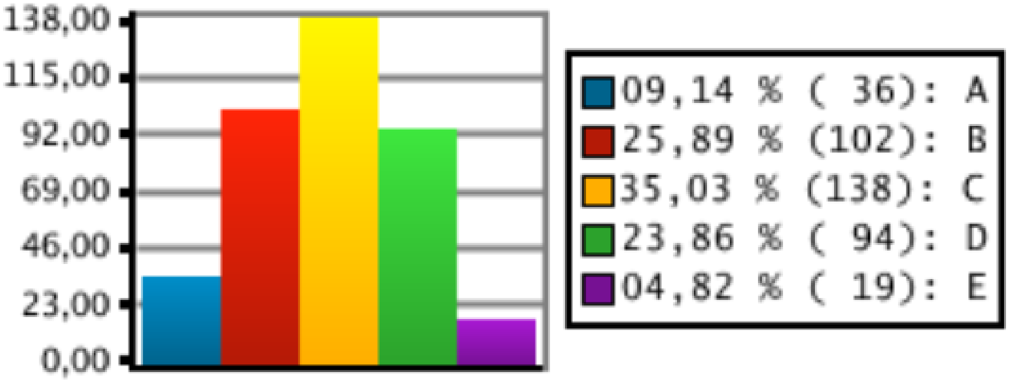

Legende:

A Sehr selten/nie
B Selten (etwa 1 x pro Monat)
C Gelegentlich (etwa 1 x pro Woche)
D Oft (mehrmals pro Woche)
E Sehr oft (ein bis mehrmals täglich)
() Absolutwert

A kann die Arbeitsaufträge gut erledigen, wenn er/sie sich an die vom Betrieb vorgesehenen Wege hält. B kann die Arbeitsaufträge nur bewältigen, wenn er/sie von den vom Betrieb vorgesehenen Wegen abweicht. Welcher der beiden Arbeitsplätze ist Ihrem am ähnlichsten?

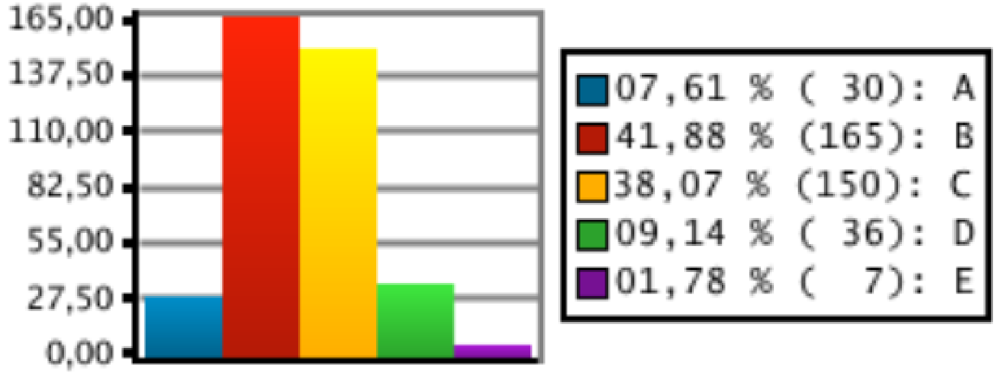

Legende:

A Genau wie der von A
B Ähnlich wie der von A
C Zwischen A und B
D Ähnlich wie der von B
E Genau wie der von B

A hat einen Arbeitsplatz, der im Großen und Ganzen so eingerichtet ist dass man gut arbeiten kann. B hat einen Arbeitsplatz, bei dem er/sie einige Dinge nur schwer erreichen kann und in seinen Bewegungen oft behindert ist. Welcher der beiden Arbeitsplätze ist Ihrem am ähnlichsten?

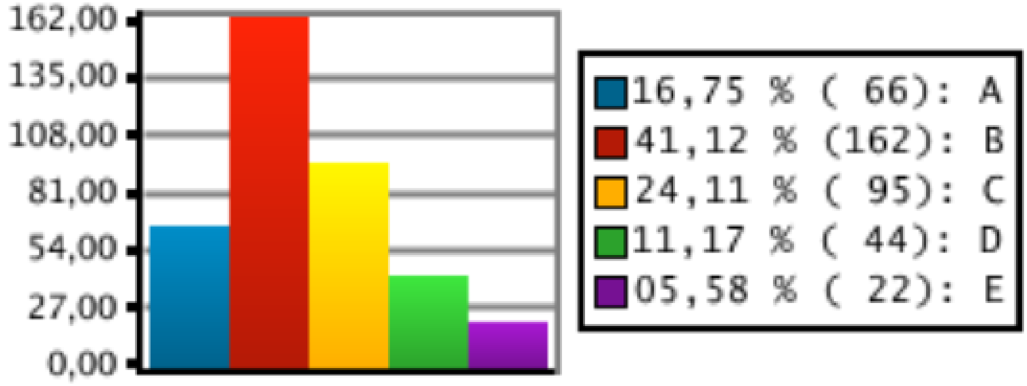

Legende:

A Genau wie der von A
B Ähnlich wie der von A
C Zwischen A und B
D Ähnlich wie der von B
E Genau wie der von B
() Absolutwert

A hat Unterlagen und Informationen, die immer genau stimmen und aktuell sind. B hat Unterlagen, bei denen Informationen oft unvollständig und veraltet sind. Welcher der beiden Arbeitsplätze ist Ihrem am ähnlichsten?

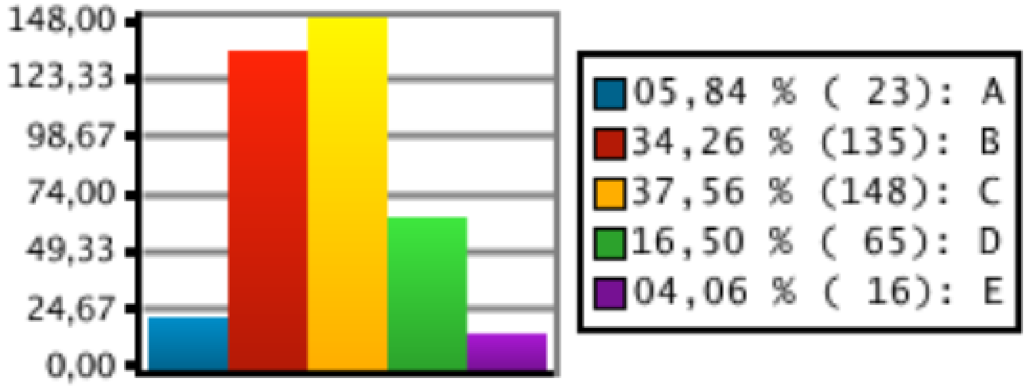

Legende:

A Genau wie der von A
B Ähnlich wie der von A
C Zwischen A und B
D Ähnlich wie der von B
E Genau wie der von B
() Absolutwert

A muss viel Zeit damit vertun, um sich Informationen, Material oder Werkzeuge zum Weiterarbeiten zu beschaffen. B stehen die nötigen Informationen, Material oder Werkzeuge immer zur Verfügung. Welcher der beiden Arbeitsplätze ist Ihrem am ähnlichsten?

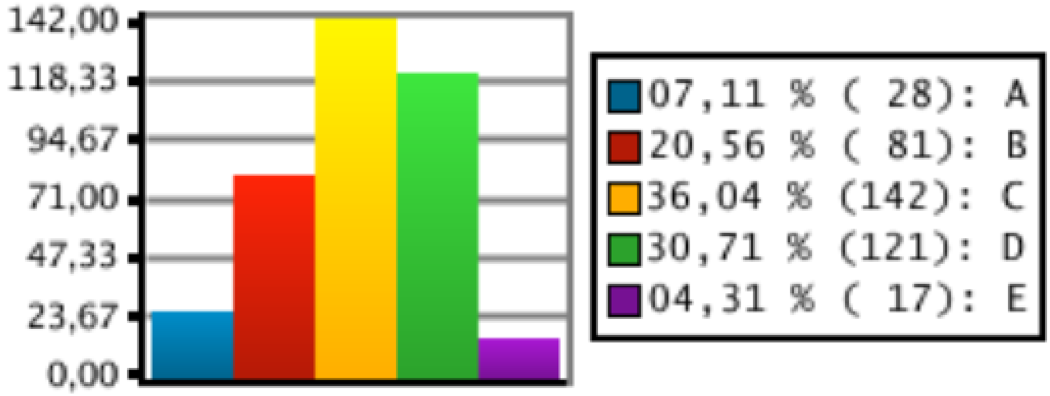

Legende:

A Genau wie der von A
B Ähnlich wie der von A
C Zwischen A und B
D Ähnlich wie der von B
E Genau wie der von B
() Absolutwert

A muss mit Material und Arbeitsmitteln arbeiten, das nicht viel taugt. B arbeitet mit einwandfreiem Material und Arbeitsmitteln. Welcher der beiden Arbeitsplätze ist Ihrem Arbeitsplatz am ähnlichsten?

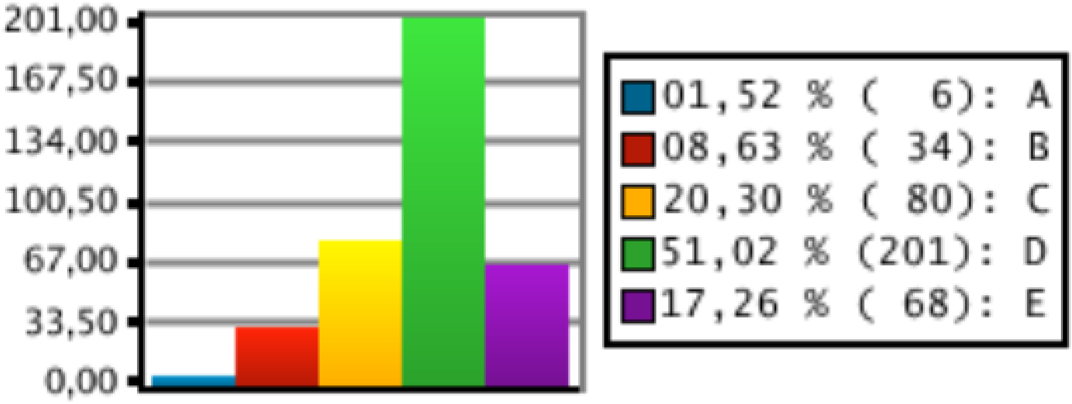

Legende:

A Genau wie der von A
B Ähnlich wie der von A
C Zwischen A und B
D Ähnlich wie der von B
E Genau wie der von B
() Absolutwert

Inwieweit ist Ihre Arbeit körperlich abwechslungsreich?

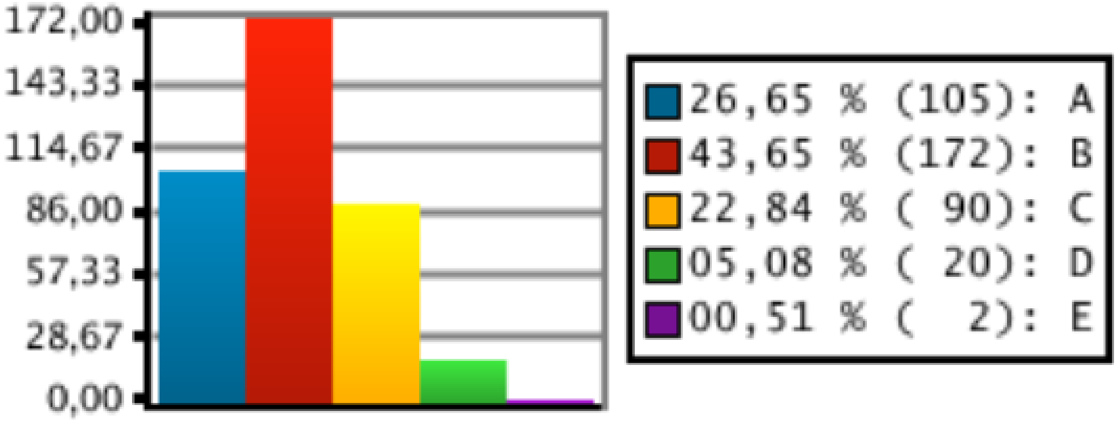

Legende:

A Sehr wenig
B Wenig
C Einigermaßen
D Stark
E Sehr stark
() Absolutwert

Wie häufig werden Sie durchschnittlich bei Ihrer Arbeit von Ihrem/Ihrer Vorgesetzten unterbrochen (zum Beispiel wegen einer Auskunft)?

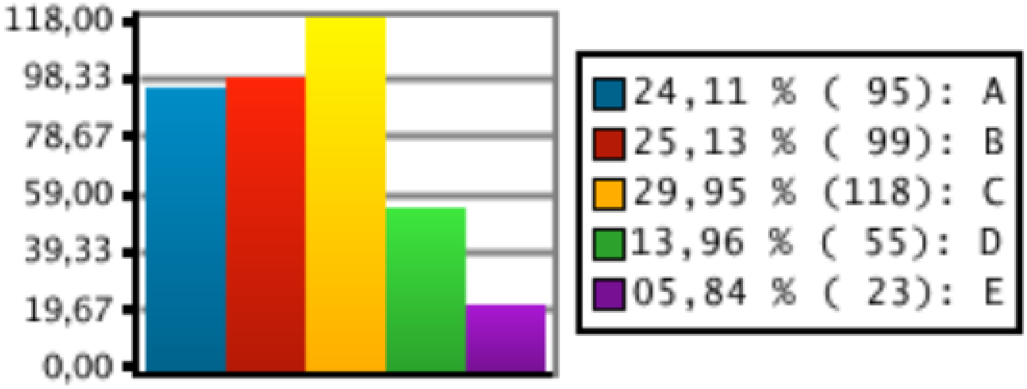

Legende:

A Sehr selten/nie
B Selten (etwa 1 x pro Monat)
C Gelegentlich (etwa 1 x pro Woche)
D Oft (mehrmals pro Woche)
E Sehr oft (ein bis mehrmals täglich)
() Absolutwert

Wie häufig werden Sie durch andere Kollegen/Mitarbeiter bei Ihrer Arbeit unterbrochen?

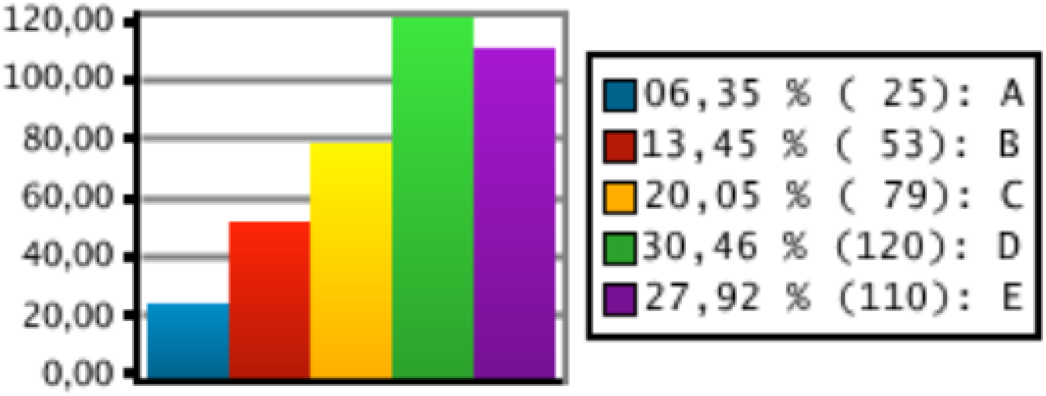

Legende:

A Sehr selten/nie
B Selten (etwa 1 x pro Monat)
C Gelegentlich (etwa 1 x pro Woche)
D Oft (mehrmals pro Woche)
E Sehr oft (ein bis mehrmals täglich)
() Absolutwert

Wie häufig werden Sie durch Patienten bei Ihrer Arbeit unterbrochen?

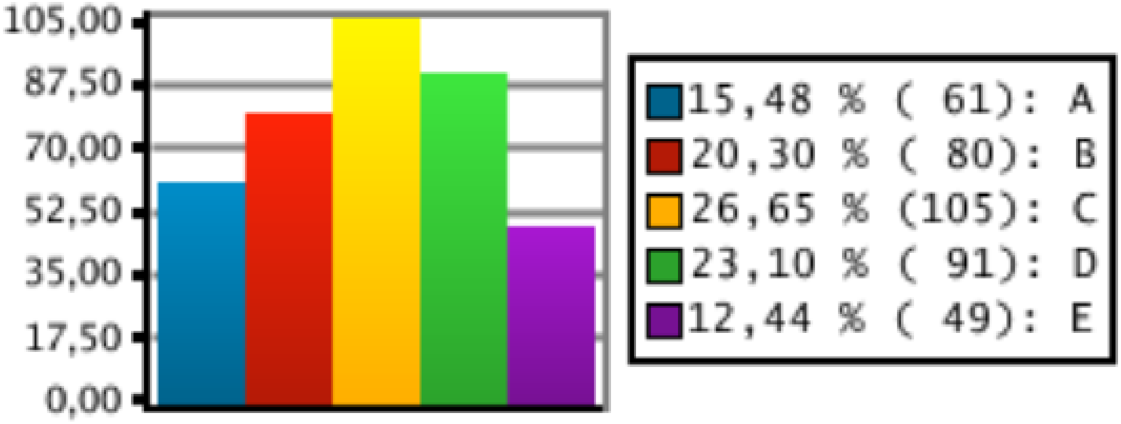

Legende:

A Sehr selten/nie
B Selten (etwa 1 x pro Monat)
C Gelegentlich (etwa 1 x pro Woche)
D Oft (mehrmals pro Woche)
E Sehr oft (ein bis mehrmals täglich)
() Absolutwert

Kommt es vor, dass Sie aktuelle Arbeiten unterbrechen müssen, weil etwas Wichtiges dazwischen kommt?

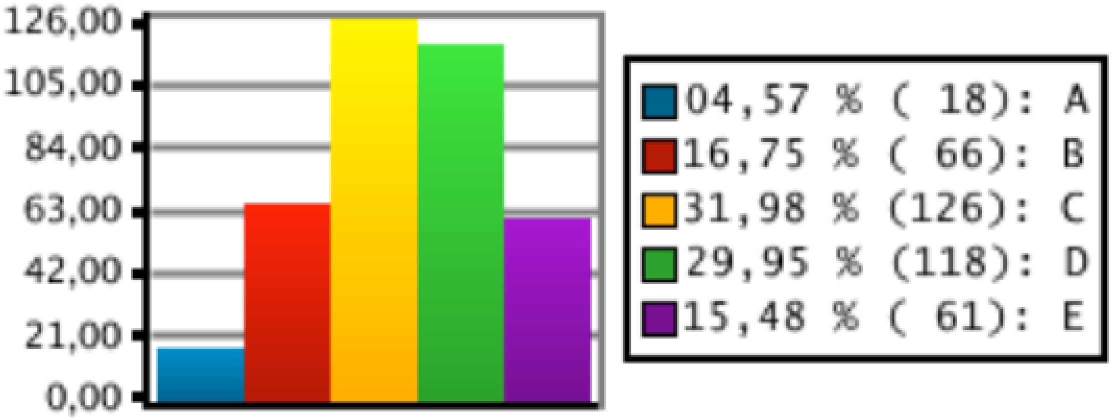

Legende:

A Sehr selten/nie
B Selten (etwa 1 x pro Monat)
C Gelegentlich (etwa 1 x pro Woche)
D Oft (mehrmals pro Woche)
E Sehr oft (ein bis mehrmals täglich)
() Absolutwert

Wie häufig kommt es vor, dass Sie an mehreren Aufgaben gleichzeitig arbeiten müssen und zwischen den Arbeitsaufgaben hin und her springen?

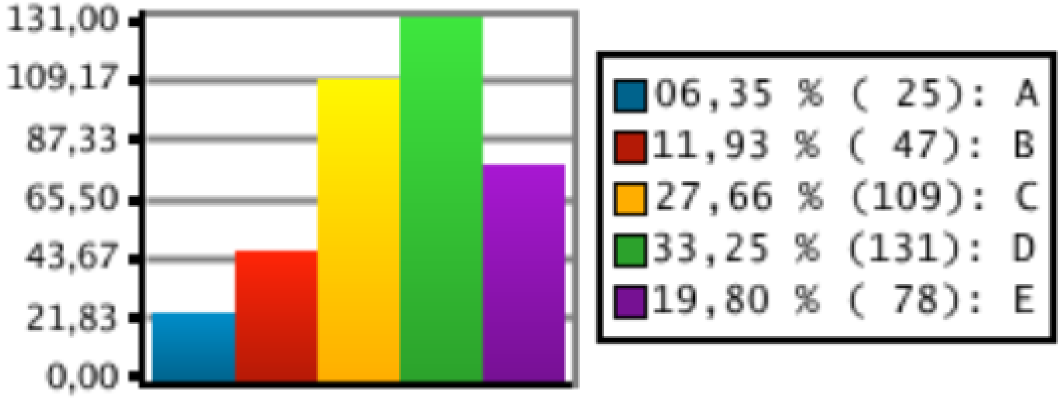

Legende:

A Sehr selten/nie
B Selten (etwa 1 x pro Monat)
C Gelegentlich (etwa 1 x pro Woche)
D Oft (mehrmals pro Woche)
E Sehr oft (ein bis mehrmals täglich)
() Absolutwert

Wie häufig kommt es vor, dass Sie sich bei der Arbeit so konzentrieren müssen, dass Störungen zu zusätzlichem Aufwand führen (Sie müssen zum Beispiel Zahlen oder Namen nochmals nachschlagen, Arbeitsmittel noch einmal neu ansetzen oder Berechnungen von vorne beginnen?)

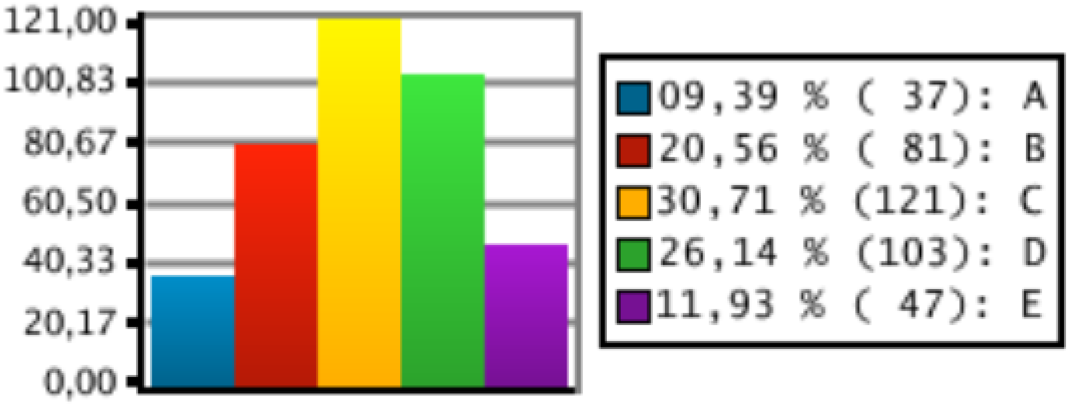

Legende:

A Sehr selten/nie
B Selten (etwa 1 x pro Monat)
C Gelegentlich (etwa 1 x pro Woche)
D Oft (mehrmals pro Woche)
E Sehr oft (ein bis mehrmals täglich)
() Absolutwert

Müssen Sie Informationen für kurze Zeit im Kopf behalten, die man sich schwer merken kann (zum Beispiel Zahlen, Namen, Tabellen)?

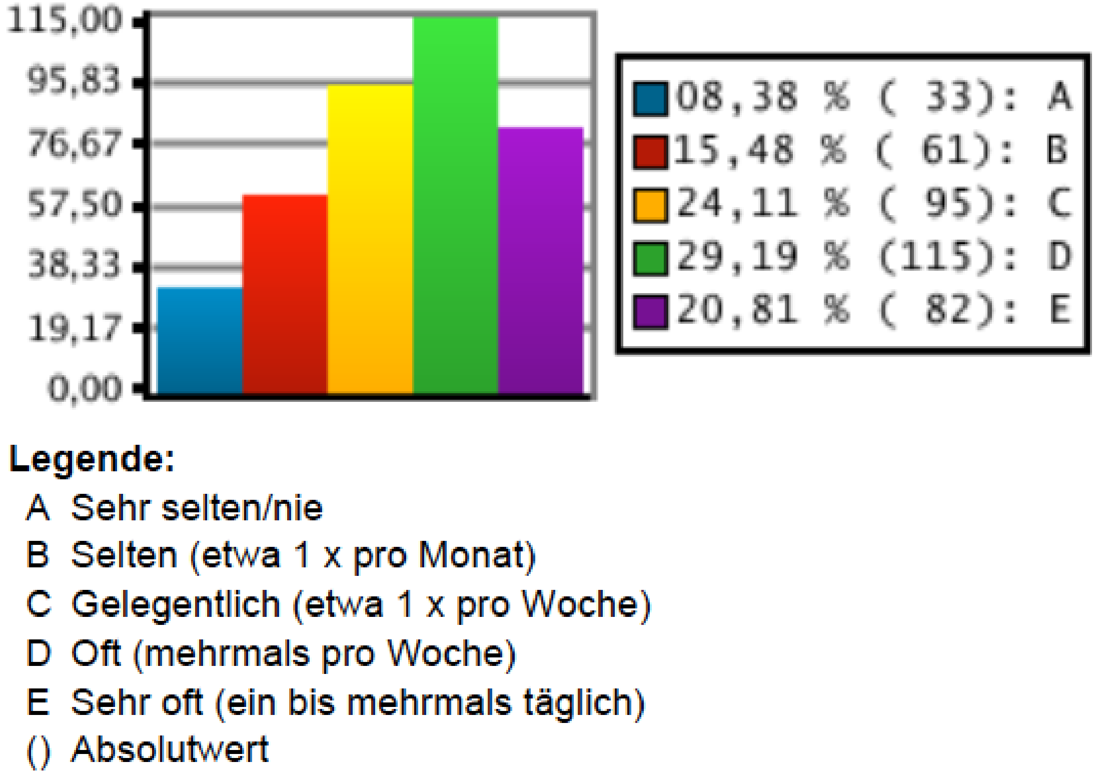

Wie oft müssen Sie bei Ihrer Arbeit viele Dinge gleichzeitig im Kopf haben?

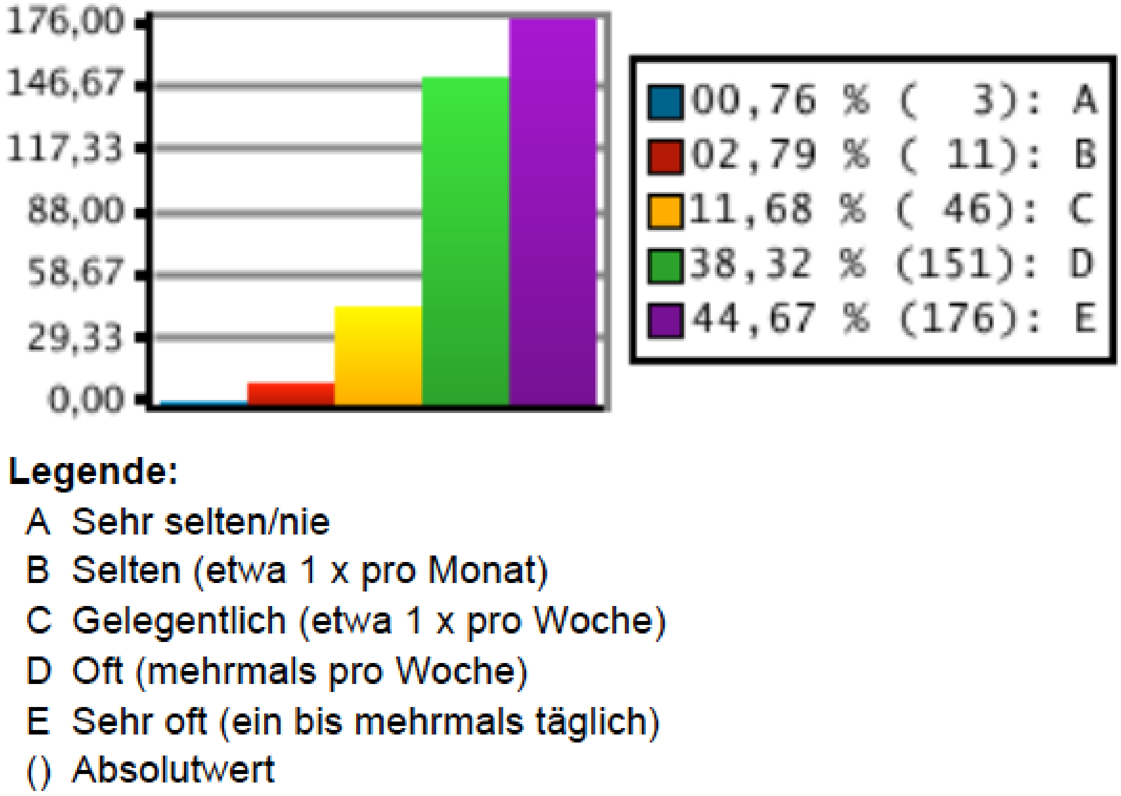

Kommt es vor, dass Sie eine Zeit lang bereit sein müssen, ohne dass etwas passiert und Sie dann sofort reagieren müssen?

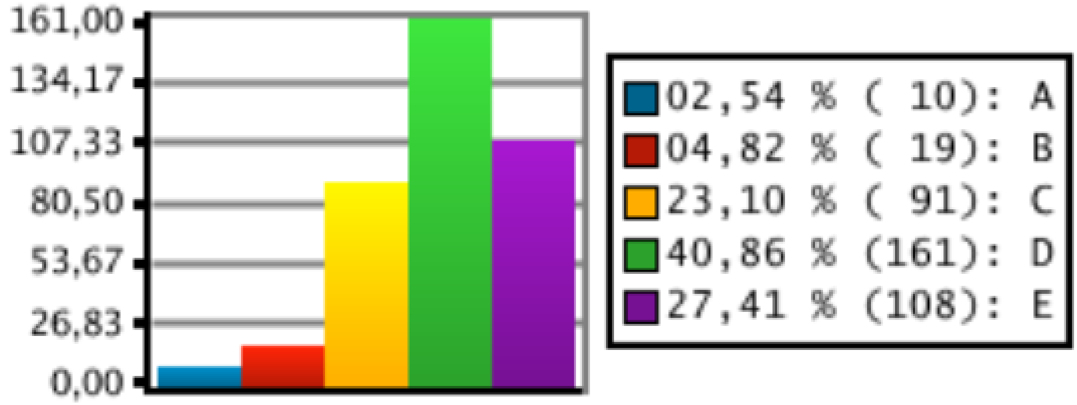

Legende:

A Sehr selten/nie
B Selten (etwa 1 x pro Monat)
C Gelegentlich (etwa 1 x pro Woche)
D Oft (mehrmals pro Woche)
E Sehr oft (ein bis mehrmals täglich)
() Absolutwert

Wie oft gibt es Momente in Ihrer Arbeit, die für kurze Zeit höchste Konzentration erfordern?

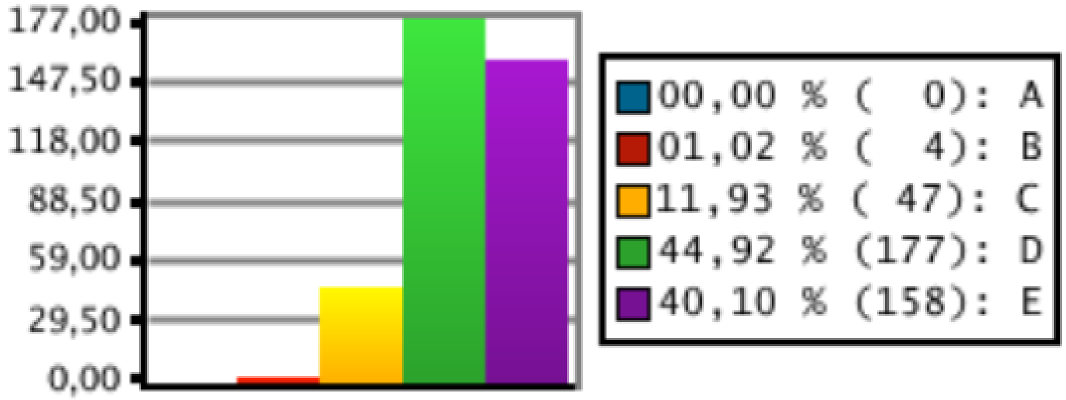

Legende:

A Sehr selten/nie
B Selten (etwa 1 x pro Monat)
C Gelegentlich (etwa 1 x pro Woche)
D Oft (mehrmals pro Woche)
E Sehr oft (ein bis mehrmals täglich)
() Absolutwert

Wie häufig stehen Sie unter Zeitdruck?

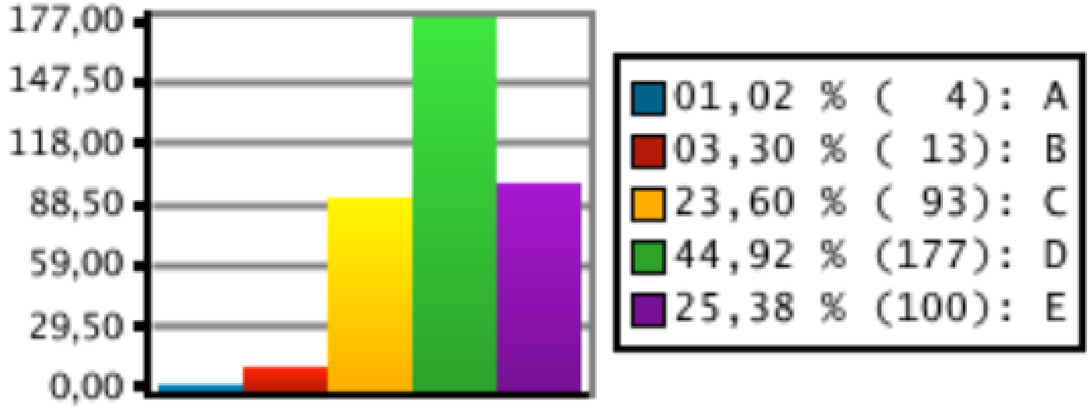

Legende:

A Sehr selten/nie
B Selten (etwa 1 x pro Monat)
C Gelegentlich (etwa 1 x pro Woche)
D Oft (mehrmals pro Woche)
E Sehr oft (ein bis mehrmals täglich)
() Absolutwert

Wie häufig passiert es, dass Sie schneller arbeiten, als sie es normalerweise tun, um die Arbeit zu schaffen?

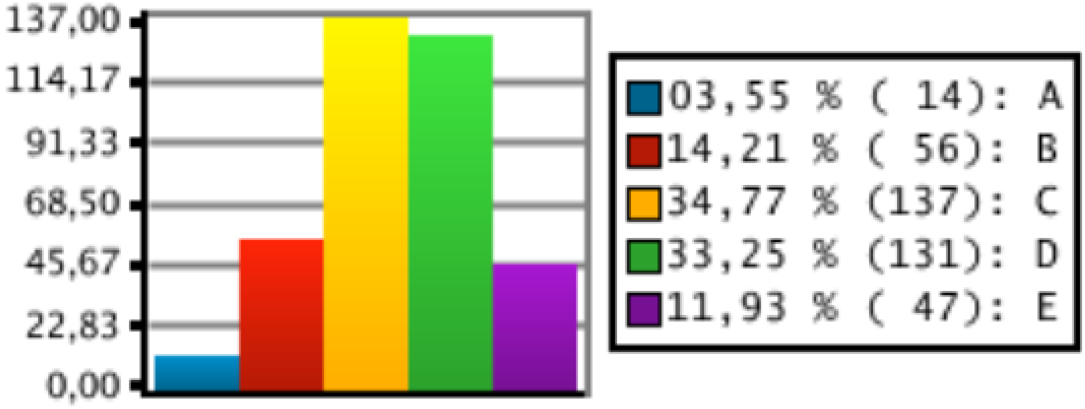

Legende:

A Sehr selten/nie
B Selten (etwa 1 x pro Monat)
C Gelegentlich (etwa 1 x pro Woche)
D Oft (mehrmals pro Woche)
E Sehr oft (ein bis mehrmals täglich)
() Absolutwert

Wie oft kommt es vor, dass Sie wegen zu viel Arbeit nicht oder verspätet in die Pause gehen können?

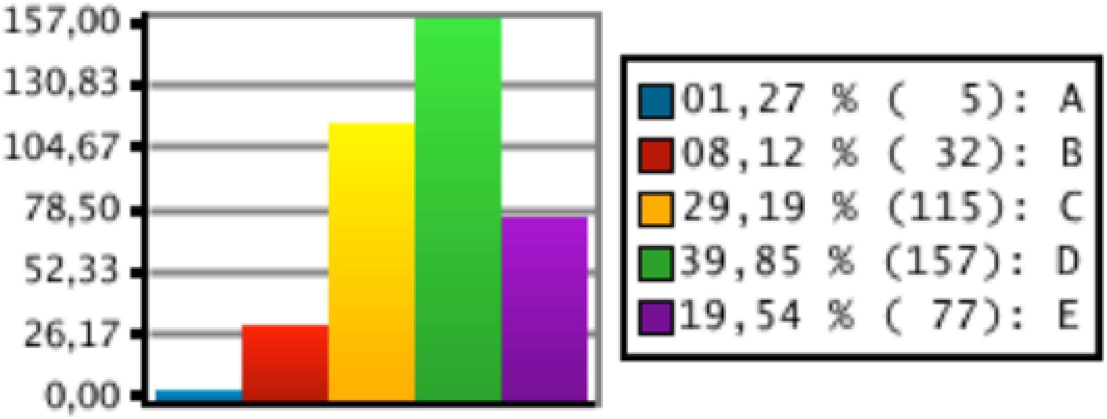

Legende:

A Sehr selten/nie
B Selten (etwa 1 x pro Monat)
C Gelegentlich (etwa 1 x pro Woche)
D Oft (mehrmals pro Woche)
E Sehr oft (ein bis mehrmals täglich)
() Absolutwert

Wie oft kommt es vor, dass Sie wegen zu viel Arbeit verspätet in den Feierabend gehen können?

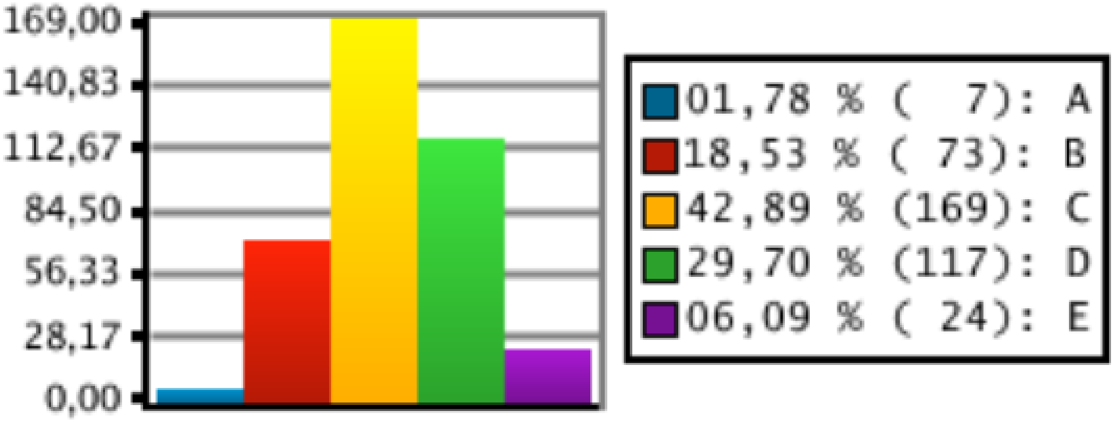

Legende:

A Sehr selten/nie
B Selten (etwa 1 x pro Monat)
C Gelegentlich (etwa 1 x pro Woche)
D Oft (mehrmals pro Woche)
E Sehr oft (ein bis mehrmals täglich)
() Absolutwert

Wie oft wird bei Ihrer Arbeit ein hohes Arbeitstempo verlangt?

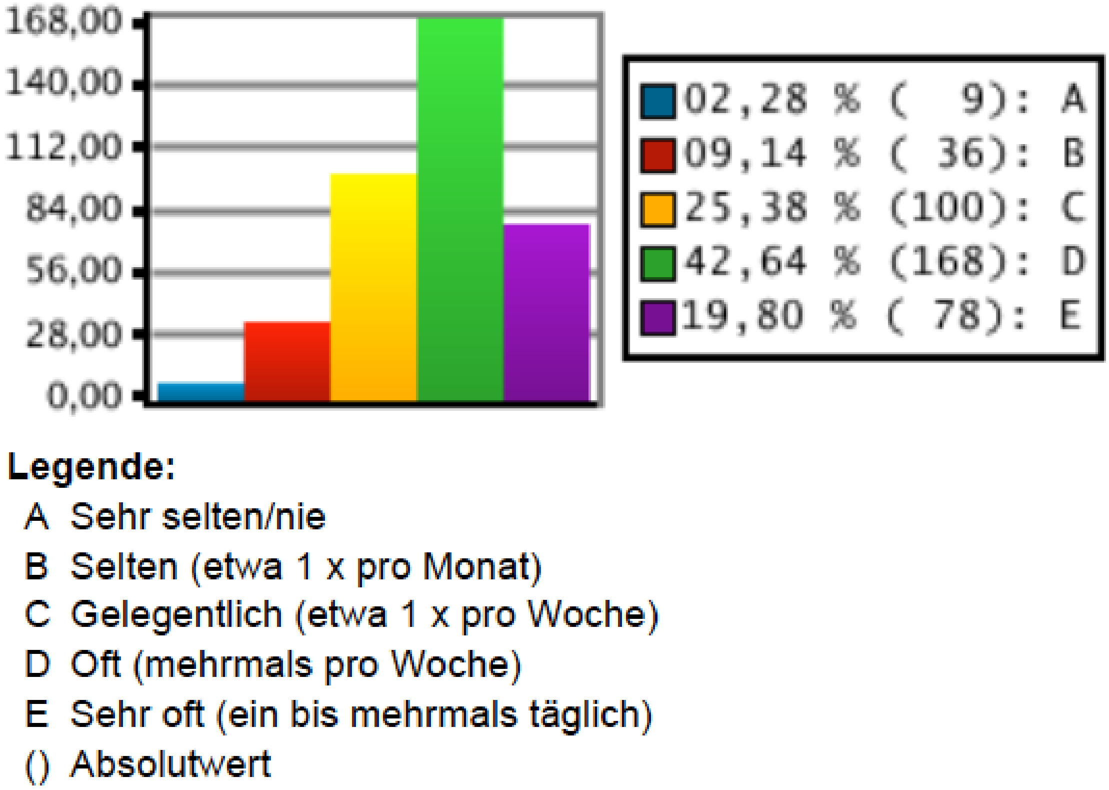

Im Folgenden ging es darum wie gut AnästhesistInnen mit anderen Personen Kontakt aufnehmen, jemandem helfen oder sich mit anderen unterhalten konnten. Es sollten dabei räumliche, technische oder organisatorische Gründe bedacht werden, die dies fördern oder behindern können. Soziale Gründe (zum Beispiel man konnte eine Person nicht leiden und wollte deshalb keinen Kontakt) oder fachliche Gründe (man hatte nicht die entsprechenden Kenntnisse um jemandem zu helfen) waren mit diesen Fragen nicht gemeint.

Mit wie vielen verschiedenen KollegInnen können Sie während der Arbeit Kontakt aufnehmen (zum Beispiel um sich mit ihnen über private oder dienstliche Dinge zu unterhalten)?

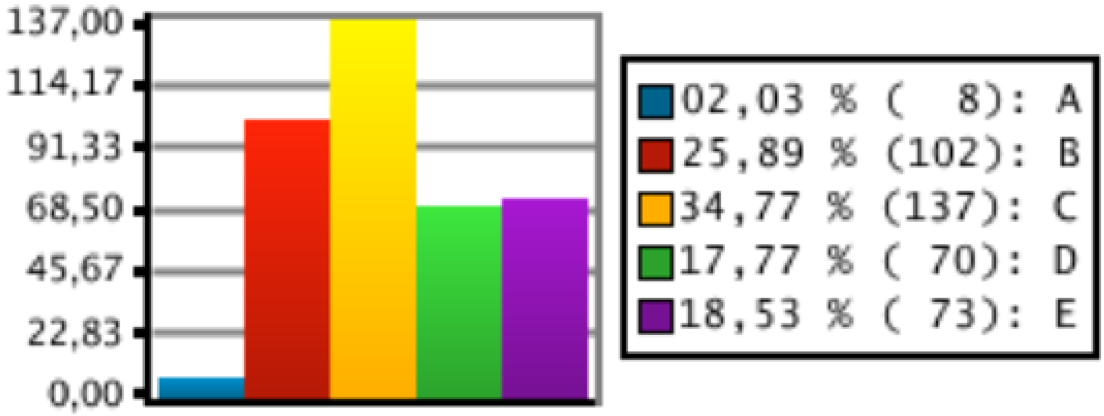

Legende:

A Mit keiner Person
B Mit 1 - 2 Personen
C Mit 3-5 Personen
D Mit 6 - 10 Personen
E Mit mehr als 10 Personen
() Absolutwert

Mit wie vielen anderen Personen (zum Beispiel Patienten) haben Sie an einem Arbeitstag durchschnittlich Kontakt?

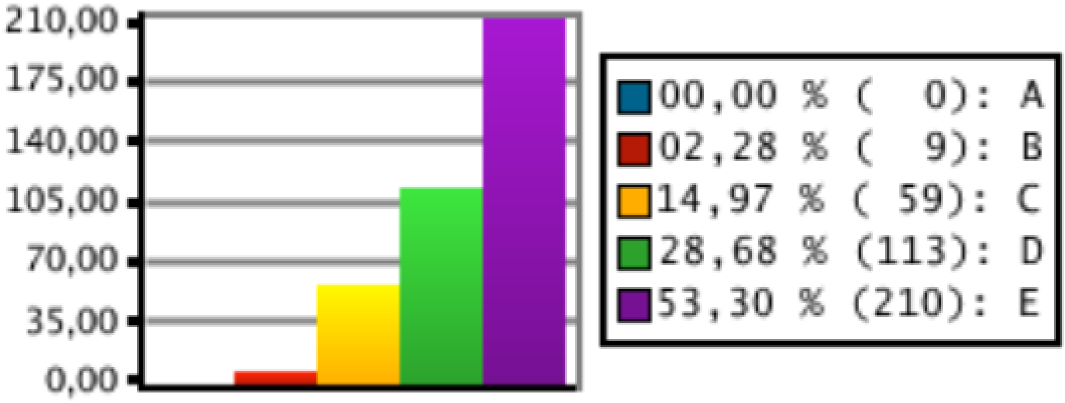

Legende:

A Mit keiner Person
B Mit 1 - 2 Personen
C Mit 3-5 Personen
D Mit 6 - 10 Personen
E Mit mehr als 10 Personen
() Absolutwert

Können Sie sich während der Arbeit mit Ihren KollegInnen über Dinge unterhalten, die nichts mit der Arbeit zu tun haben?

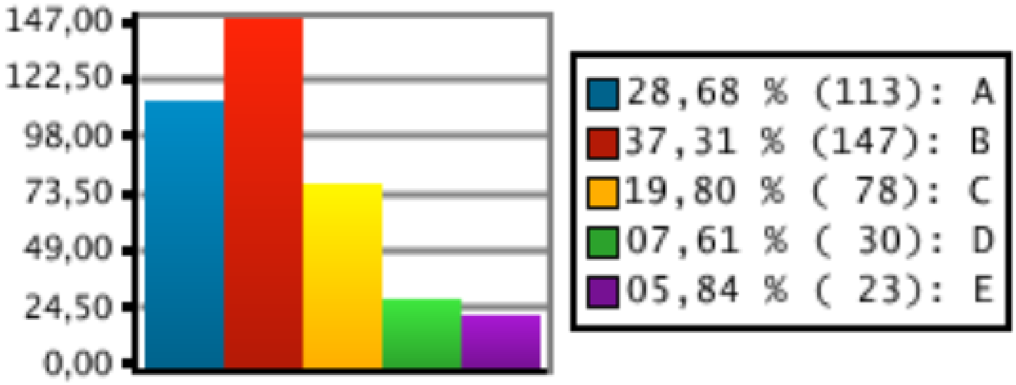

Legende:

A Ja, ohne Schwierigkeiten
B Ja, mit geringen Schwierigkeiten
C Mit einigen Schwierigkeiten
D Mit ziemlichen Schwierigkeiten
E Die Arbeit lässt es praktisch nicht zu
() Absolutwert

A kann in der Regel selbst entscheiden, mit welchen KollegInnen er oder sie zusammenarbeitet. Bei B ist immer genau vorgegeben, mit wem er oder sie jeweils zusammenarbeitet. Welcher der beiden Arbeitsplätze ist Ihrem am ähnlichsten?

Die Abbildungen werden immer größer….

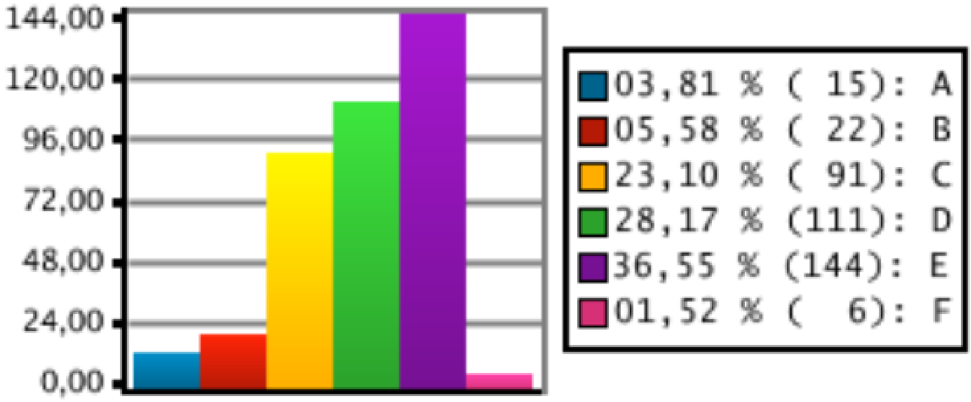

Legende:

A Genau wie der von A
B Ähnlich wie der von A
C Zwischen A und B
D Ähnlich wie der von B
E Genau wie der von B
F Ich arbeite mit niemandem zusammen
() Absolutwert

Wenn A mit einem Kollegen/Kollegin in der Zusammenarbeit nicht klar kommt, dann kann er/sie in Zukunft mit jemand anderem zusammenarbeiten. Wenn B mit einem Kollegen/Kollegin in der Zusammenarbeit nicht klar kommt, muss er/sie trotzdem weiter mit diesem Kollegen/Kollegin zusammenarbeiten. Welcher der beiden Arbeitsplätze ist ihrem am ähnlichsten?

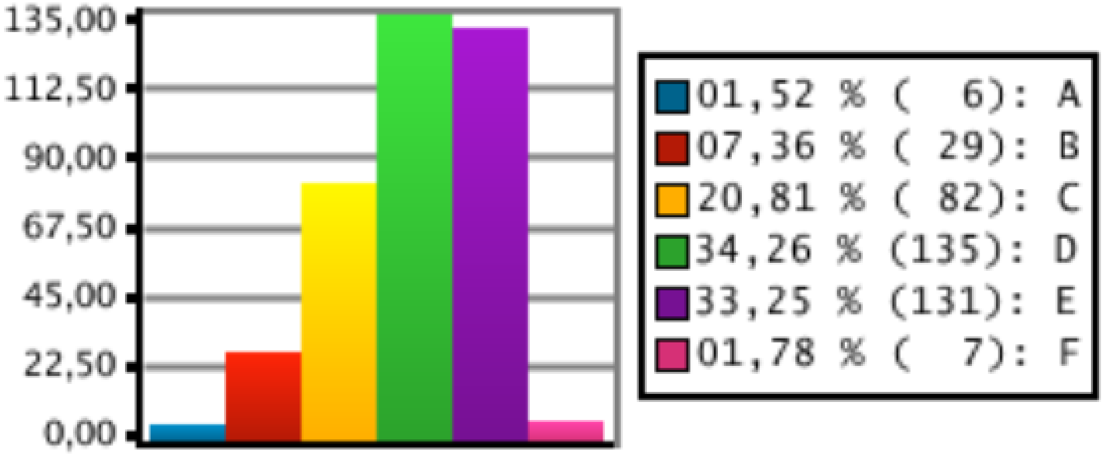

Legende:
A Genau wie der von A
B Ähnlich wie der von A
C Zwischen A und B
D Ähnlich wie der von B
E Genau wie der von B
F Ich arbeite mit niemandem zusammen
() Absolutwert

A kann in der Regel selber entscheiden, ob er/sie eine Arbeit alleine oder zusammen mit KollegInnen ausführen will. Bei B ist vorgegeben ob er/sie eine Arbeit alleine oder zusammen mit Kollegen ausführt. Welcher der beiden Arbeitsplätze ist ihrem am ähnlichsten?

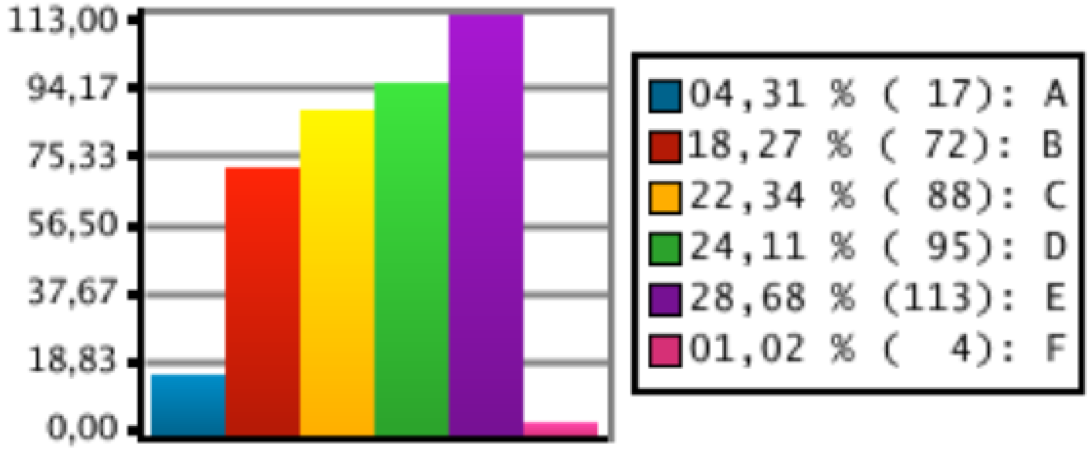

Legende:

A Genau wie der von A
B Ähnlich wie der von A
C Zwischen A und B
D Ähnlich wie der von B

Wie stark sind Sie bei Ihrer Arbeit davon abhängig, wie schnell oder wie langsam Ihre KollegInnen arbeiten?

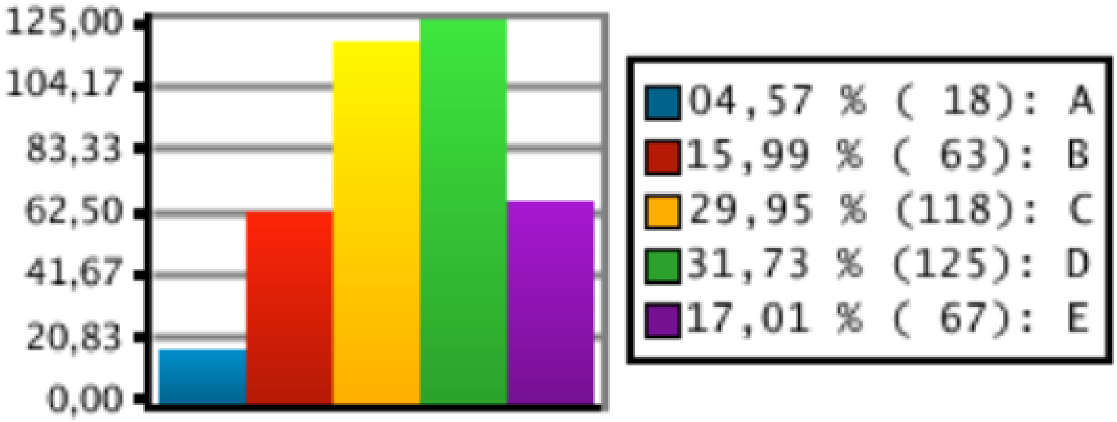

Legende:

A Gar nicht
B Wenig
C Einigermaßen
D Viel
E Sehr viel
() Absolutwert

Wie stark sind Sie bei Ihrer Arbeit davon abhängig wie gut oder wie schlecht Ihre KollegInnen arbeiten?

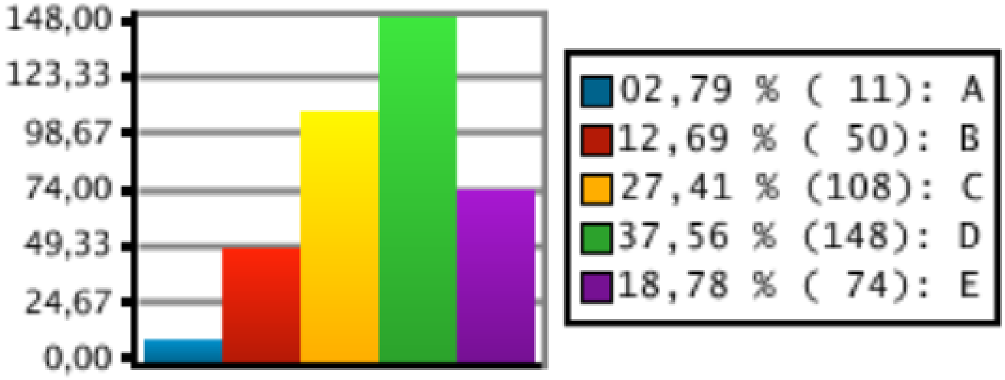

Legende:

A Gar nicht
B Wenig
C Einigermaßen
D Viel
E Sehr viel
() Absolutwert

Wenn A einen Fehler bei der Arbeit macht, dann wirkt sich dies unmittelbar auf seine/ihre KollegInnen aus. Wenn B einen Fehler bei der Arbeit macht, dann hat das keine Folgen für seine/ihre KollegInnen, sondern wirkt sich nur auf ihn(sie selbst aus. Welcher der beiden Arbeitsplätze ist ihrem am ähnlichsten?

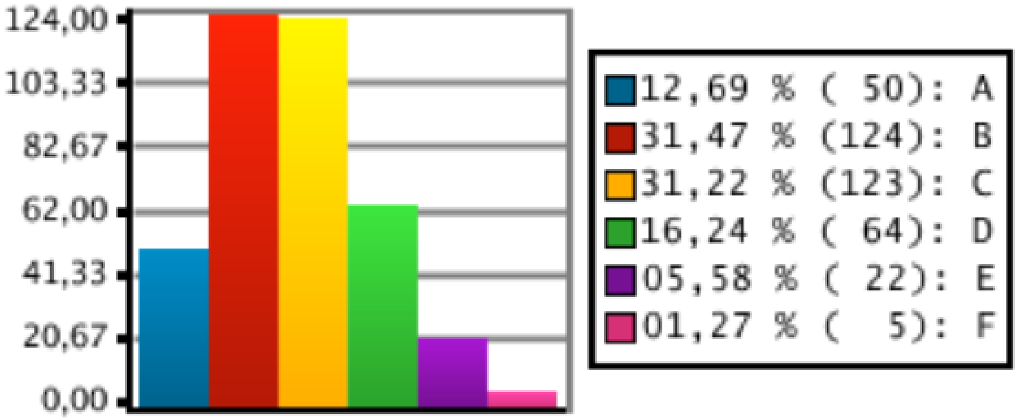

Legende:

A Genau wie der von A
B Ähnlich wie der von A
C Zwischen A und B
D Ähnlich wie der von B
E Genau wie der von B
F Ich arbeite mit niemandem zusammen
() Absolutwert

Wie stark sind Ihre KollegInnen von Ihrem Arbeitstempo abhängig?

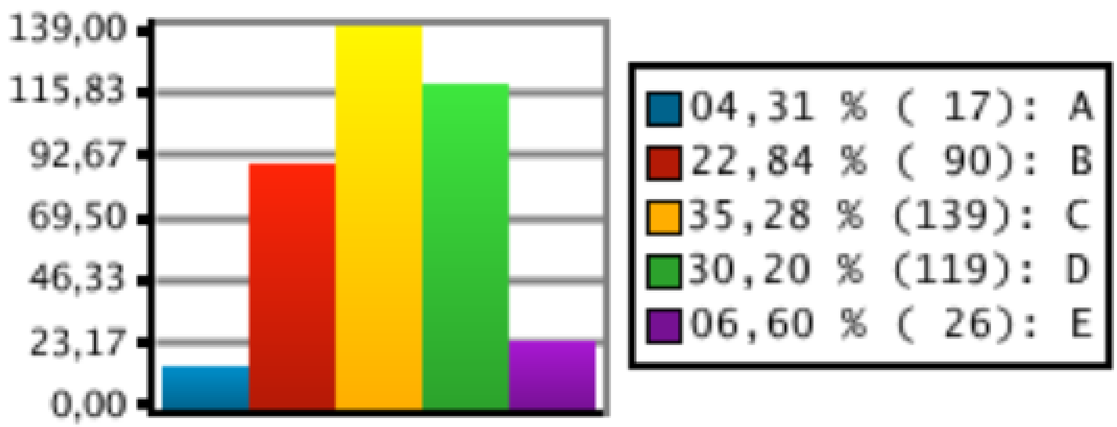

Legende:

A Gar nicht
B Wenig
C Einigermaßen
D Viel
E Sehr viel
() Absolutwert

Wie stark sind Ihre KollegInnen davon abhängig wie gut oder schlecht Sie arbeiten?

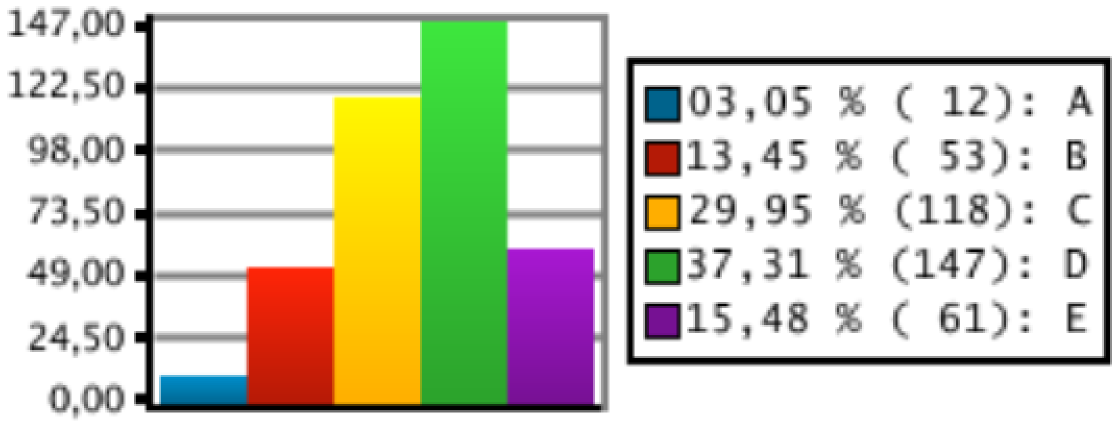

Legende:

A Gar nicht
B Wenig
C Einigermassen
D Viel
E Sehr viel
() Absolutwert

Ich arbeite mit niemanden zusammen

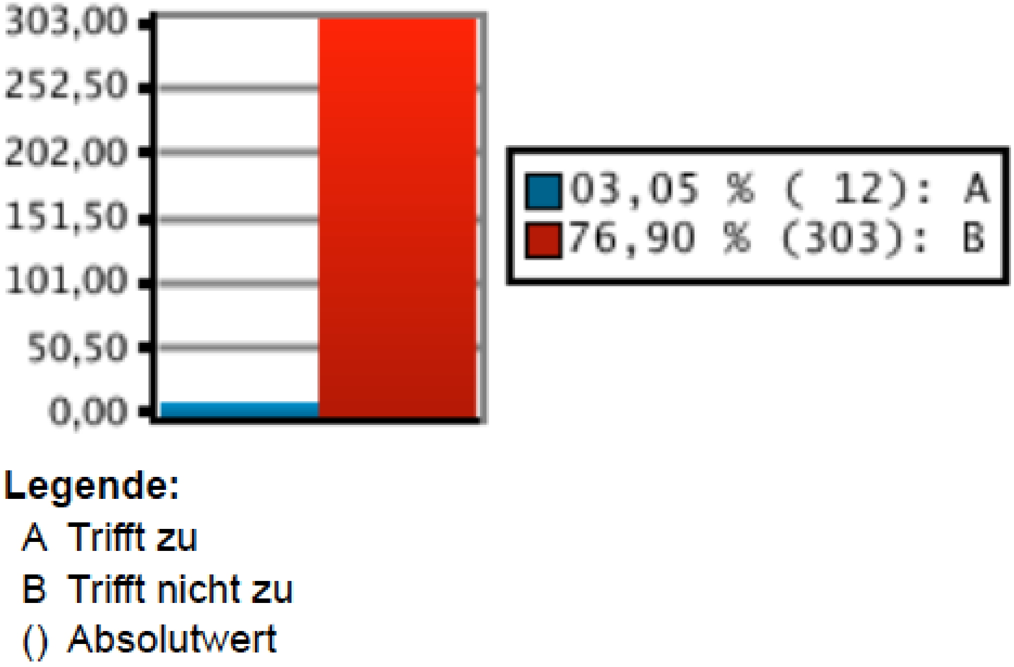

Die Fragen 116 bis 118 waren nur zu beantworten, wenn die Person mit jemandem zusammenarbeitete

Komplizierte Entscheidungen muß ich in der Arbeit...

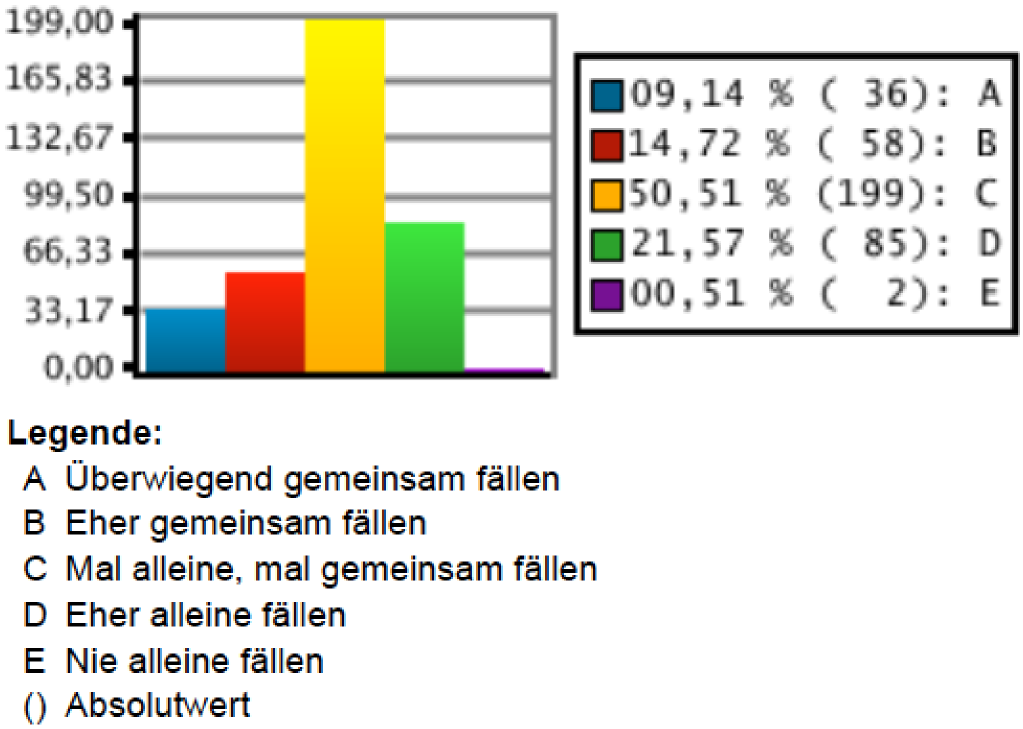

Inwieweit erfordert Ihre Arbeit eine gemeinsame Planung mit Ihren KollegInnen?

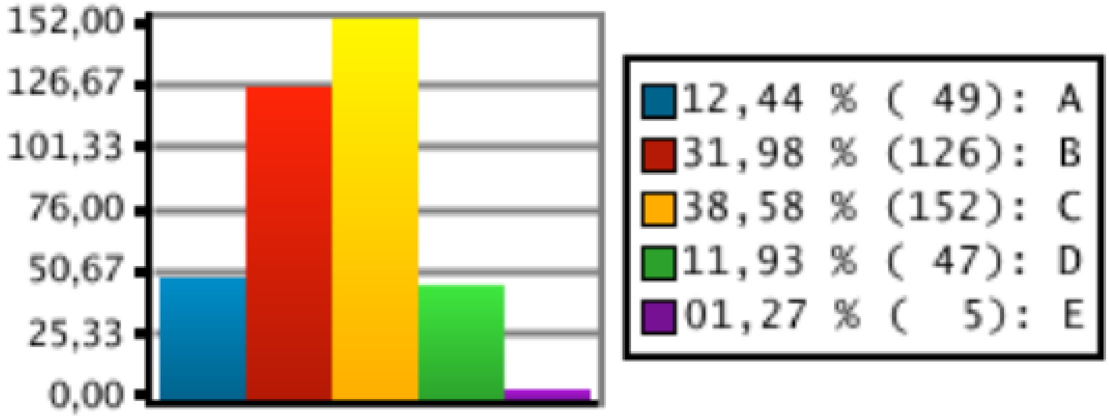

Legende:

A Sehr stark
B Stark
C Mittel
D Wenig
E Sehr wenig, gar nicht
() Absolutwert

Inwieweit erfordert Ihre Arbeit, dass Sie genau wissen, wie weit die anderen gerade sind?

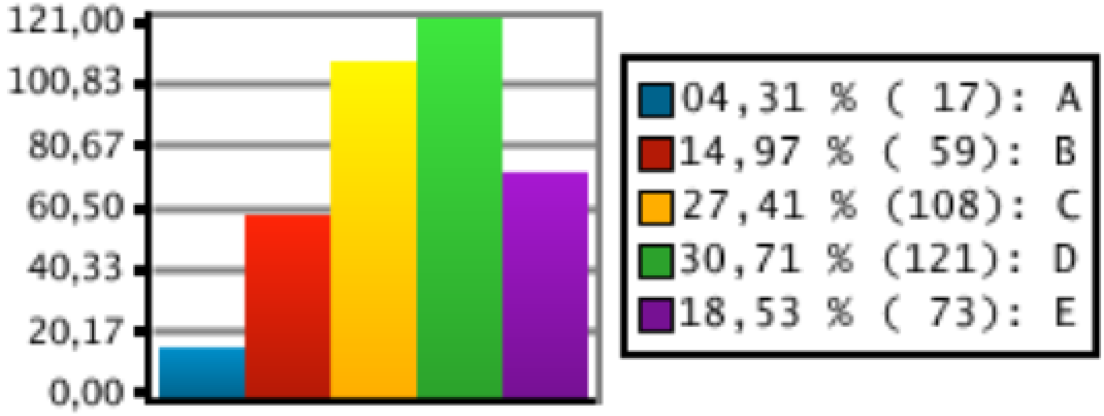

Legende:

A Sehr wenig/gar nicht
B Wenig
C Einigermaßen
D Ziemlich
E Sehr
() Absolutwert

Inwieweit müssen andere genau wissen, wie weit Sie gerade mit der Arbeit sind?

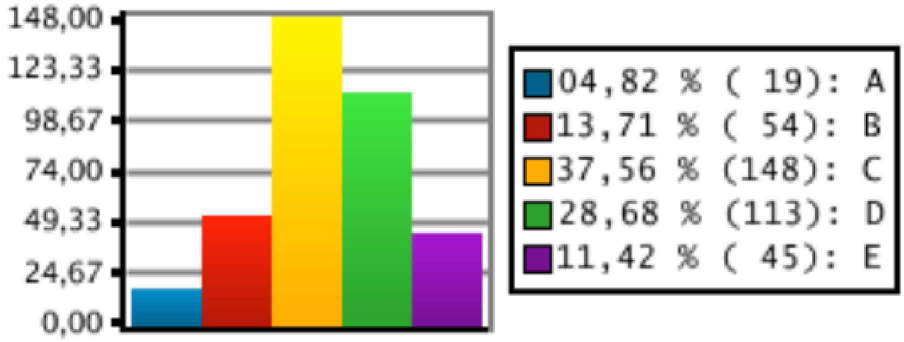

Legende:
A Sehr wenig/gar nicht
B Wenig
C Einigermassen
D Ziemlich
E Sehr
() Absolutwert

7.6. Mitarbeiterbindung

Welche Art von Arbeitsvertrag haben Sie?

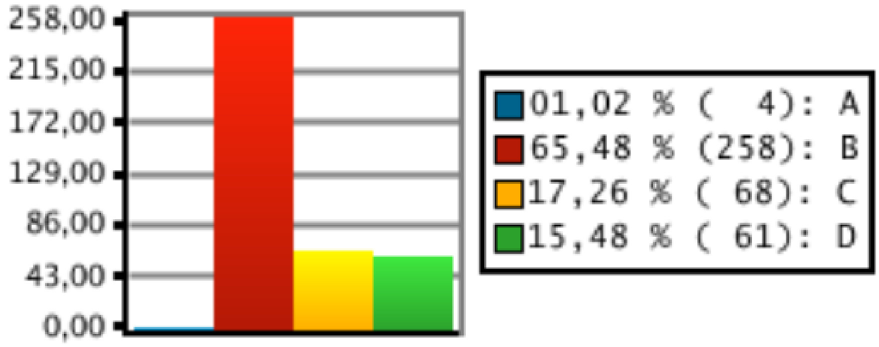

Legende:
A Auf selbstständiger Basis
B Einen zeitlich unbefristeten Vertrag
C Einen zeitlich befristeten Vertrag
D Einen Ausbildungsvertrag (Lehre) oder eine andere Fort- oder Weiterbildungsvereinbarung
() Absolutwert

Wie lange ist die genaue Vertragslaufzeit in Monaten?

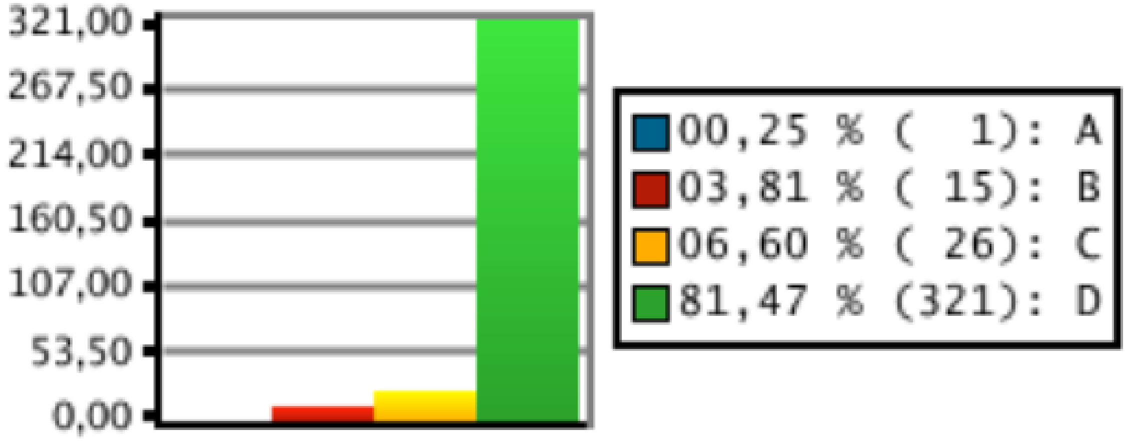

Legende:
A Weniger als 6 Monate
B 6 bis 12 Monate
C 12 bis 24 Monate
D Mehr als 48 Monate
() Absolutwert

Arbeiten Sie...

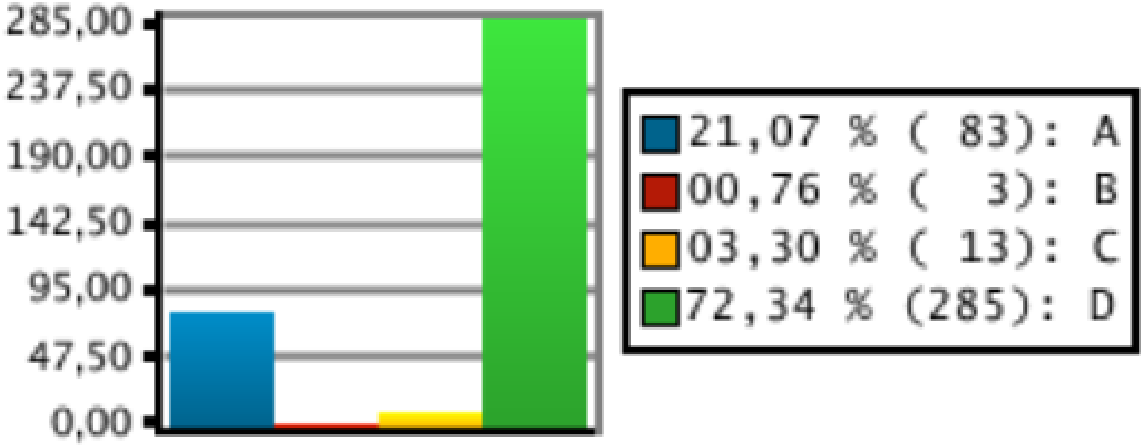

Legende:
A In gleich bleibenden Schichten (morgens, nachmittags oder abends)
B In geteilter Schicht (mit mindestens vier Stunden Pause dazwischen)
C In Nachtschichten
D In wechselnden Schichten
() Absolutwert

Wie viele Tage insgesamt fehlten Sie in den letzten 12 Monaten an Ihrem Arbeitsplatz aufgrund von Gesundheitsproblemen?

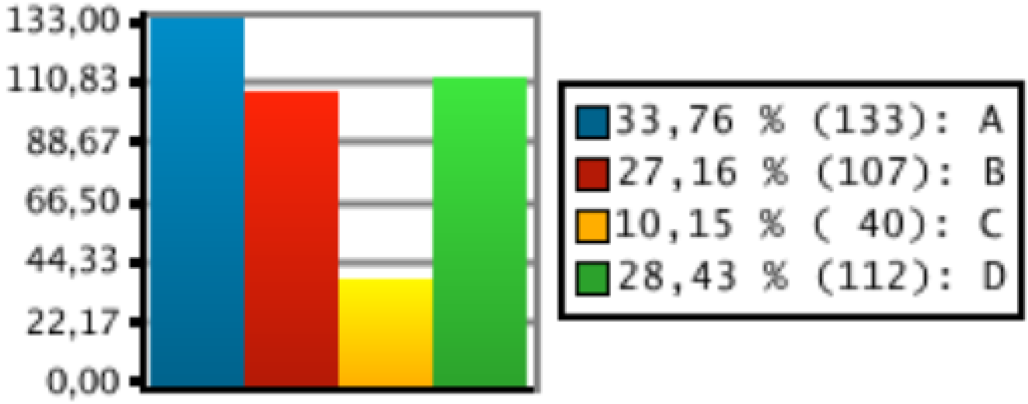

Legende:

A 1 bis 3 Tage
B Mehr als 3 Tage
C Mehr als 15 Tage
D Keine Fehltage
() Absolutwert

Haben Sie in den vergangenen 12 Monaten gearbeitet, wenn Sie krank waren?

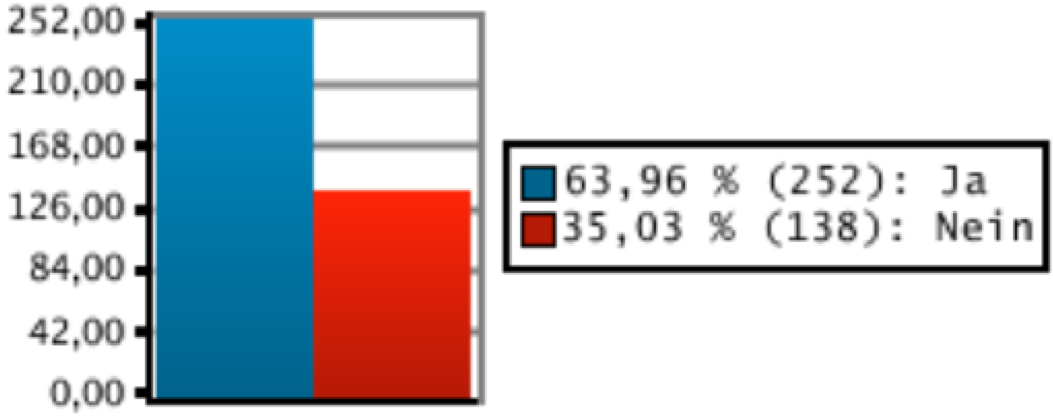

Legende:

() Absolutwert

In welchem Maß stimmen Sie den folgenden Aussagen in Zusammenhang mit Ihrer Arbeit zu?

Ich könnte meine Arbeit in den nächsten 6 Monaten verlieren.

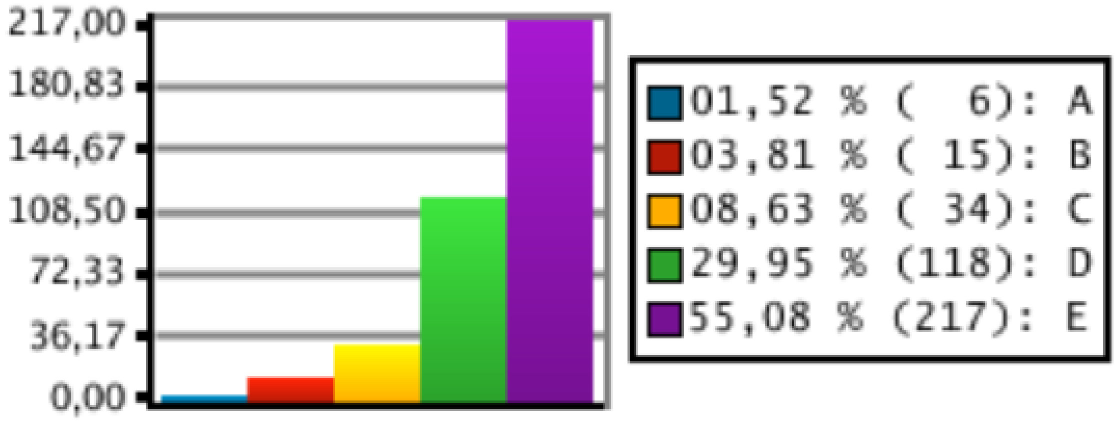

Legende:

A Stimme stark zu
B Stimme zu
C Stimme weder zu noch nicht zu
D Stimme nicht zu
E Stimme überhaupt nicht zu
() Absolutwert

Ich werde für meine Arbeit gut bezahlt

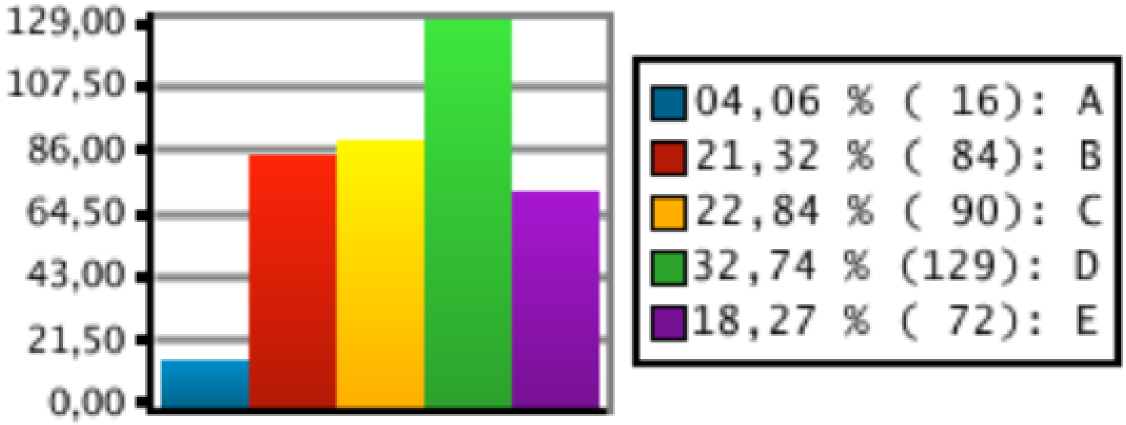

Legende:

A Stimme stark zu
B Stimme zu
C Stimme weder zu noch nicht zu
D Stimme nicht zu
E Stimme überhaupt nicht zu
() Absolutwert

Meine Arbeit bietet gute Karrierechancen

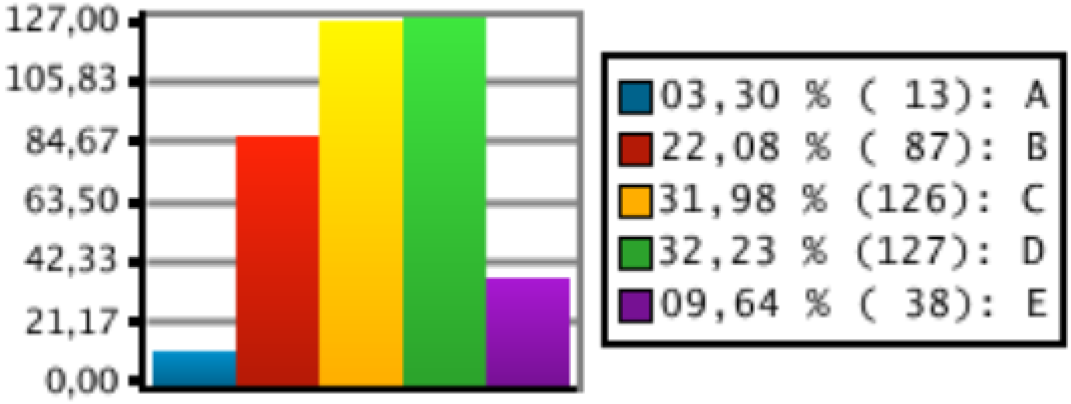

Legende:
- A Stimme stark zu
- B Stimme zu
- C Stimme weder zu noch nicht zu
- D Stimme nicht zu
- E Stimme überhaupt nicht zu
- () Absolutwert

Ich fühle mich in diesem Unternehmen „zu Hause“

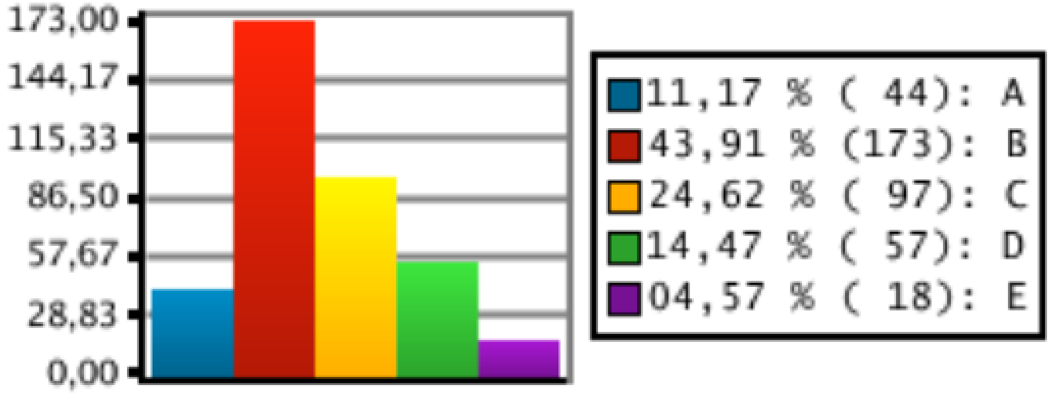

Legende:
- A Stimme stark zu
- B Stimme zu
- C Stimme weder zu noch nicht zu
- D Stimme nicht zu

Ich habe sehr gute Freunde bei der Arbeit

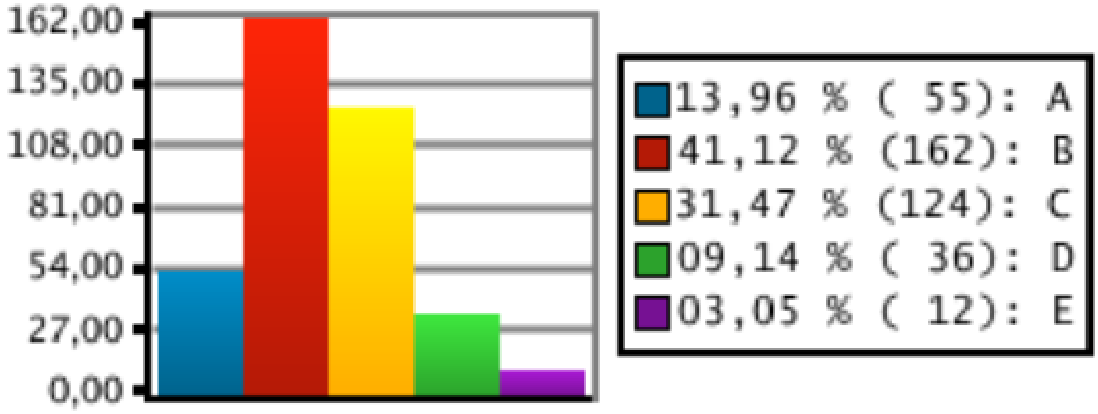

Legende:

A Stimme stark zu
B Stimme zu
C Stimme weder zu noch nicht zu
D Stimme nicht zu
E Stimme überhaupt nicht zu
() Absolutwert

Wenn ich meine derzeitige Arbeit verlieren oder kündigen würde, wäre es einfach für mich, eine Arbeit mit ähnlichem Gehalt zu finden

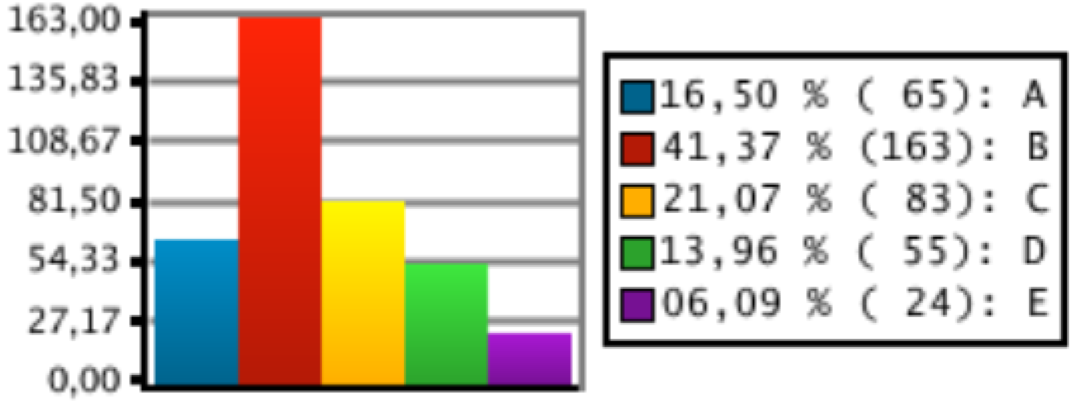

Legende:

A Stimme stark zu
B Stimme zu
C Stimme weder zu noch nicht zu
D Stimme nicht zu
E Stimme überhaupt nicht zu
() Absolutwert

Die Einrichtung, für die ich arbeite, motiviert mich, meine beste Arbeitsleistung zu erbringen

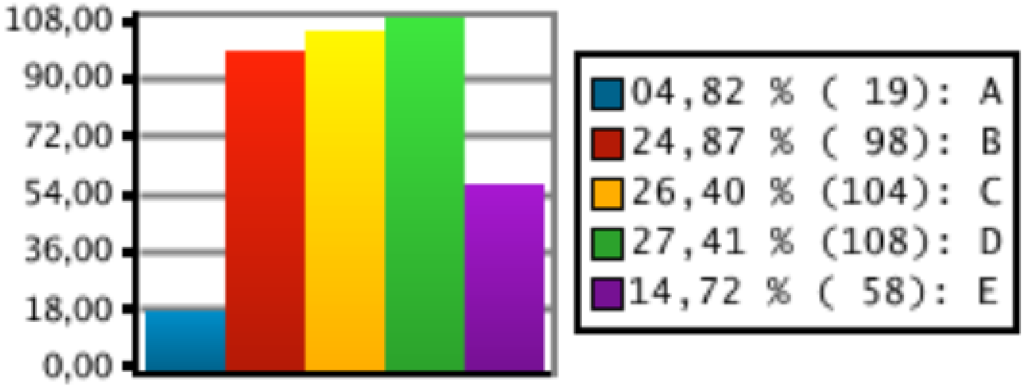

Legende:

A Stimme stark zu
B Stimme zu
C Stimme weder zu noch nicht zu
D Stimme nicht zu
E Stimme überhaupt nicht zu
() Absolutwert

7.7. Korrelationen

Um den Zusammenhang der demographischen Ergebnisse mit jenen aus dem Gesundheits- und Arbeitsbelastungsbogen darzustellen wurden mittels dem Chi-Quadrat-Test Korrelationen gerechnet. Die wichigsten signifikanten Ergebnisse sollen zur besseren Interpretation der Ergebnisse in der Folge angeführt werden. Die Ergebnisse sind entsprechend der Reihenfolge der demographischen Fragen angeordnet. Das **Geschlecht** korrelierte mit der Komplexität der Arbeit (p=0,025). Bei der **Hierarchie** korrelierte Arbeitszufriedenheit positiv mit einer führenden Position (p<0,001). AnästhesistInnen in leitenden Positionen haben vielfältigere Arbeitsfunktionen (p<0,001, aus Frage 67), sie lösen unterschiedlichere Aufträge (p<0,001), und haben komplexere Geräte zur Verfügung (p=0,008, Frage 68). Sie haben auch größeren Handlungspielraum bei der Planung von Urlauben, der Arbeitszeit und –pausen, sowie bei der Planung von Fortbildungen, bei der Aufnahme neuer MitarbeiterInnen, dem Erwerb neuer Geräte und der Ausstattung ihres Arbeitsplatzes (p<001, Frage 69). AnästhesistInnen in leitenden Funktionen haben besser ausgestatte Arbeitsplätze (p<0,001, Frage 84), sie haben genauere und aktuellere Informationen zur Verfügung (p<0,001, Frage 85), und sie können eher entscheiden mit wem sie arbeiten möchten (p<0,003, Fragen 107-109). Das **Ausbildungsniveau** korreliert mit der Komplexität der anstehenden Entscheidungen, Aufgaben

und der Arbeit (p<0,02, Fragen 57, 60 und 70), sowie der Vielfalt der Aufgaben (p=0,003, Frage 67). Weiters korreliert das Ausbildungsniveau mit dem Handlungspielraum bei der Planung von Urlauben, der Arbeitszeit und –pausen, sowie bei der Planung von Fortbildungen, bei der Aufnahme neuer MitarbeiterInnen, dem Erwerb neuer Geräte und der Ausstattung des Arbeitsplatzes (p<0,05, Frage 69). ÄrztInnen in Ausbildung zum Facharzt erhalten häufiger Kommandos von anderen (p<0,001, Frage 78). Das **Alter** korreliert mit der Komplexität der Entscheidungen (p<0,001, Frage 57) und der Menge der zu treffenden Entscheidungen (p=0,005, Frage 61). Weiters korreliert das Alter mit der Vielfalt der Aufgaben (p<0,02, Frage 71). Zudem korreliert das Alter mit dem Handlungspielraum bei der Planung von Urlauben, der Arbeitszeit und -pausen, bei der Planung von Fortbildungen, bei der Aufnahme neuer MitarbeiterInnen, dem Erwerb neuer Geräte und der Ausstattung des Arbeitsplatzes (p<0,05, Frage 69). Junge ÄrztInnen erhalten häufiger Kommandos von anderen (p<0,001, Frage 78). Schlußendlich korreliert Alter mit der Austattung der Arbeitsplätze (p<0,001, Frage 84). Der **Familienstand** korreliert mit der Komplexität der Aufgaben und der Arbeit (p<0,002, Frage 60), sowie der Vielfalt der Aufgaben (p=0,003, Frage 67), der Quantität an Routinetätigkeiten (p=0,039, Frage 72), sowie der Ausstattung des Arbeitsplatzes (p<0,001, Frage 84).

8. Diskussion

Dies ist die erste Studie, welche die Arbeitsbedingungen von AnästhesistInnen in Österreich analysiert. Eine Antwortrate von 34,4% ist für eine anonymisierte nationale Umfrage eine zufriedenstellende Antwortrate. Andere Umfragen unter ÄrztInnen erreichten Antwortraten von 31,8% bis 75,8% (LINZER et al., 2002, 191-193; MORAIS et al., 2006, 433-439; EMBRIACO et al., 2007, 686-692). Der Fragebogen welcher dieser Umfrage zu Grunde lag umfasste Fragen zu wichtigen Bereichen wie zum Beispiel psychische und körperliche Gesundheit, Arbeitsbelastung und Mitarbeiterbindung. Aus diesem Grund sind wir der Meinung, dass die Umfrage, die dieser Studie zu Grunde liegt, eine wertvolle Quelle darstellt, um das Wohlbefinden und die Arbeitsbedingungen von AnästhesistInnen in Österreich zu beurteilen.

In der Folge sollen andere Studien vorgestellt werden welche die Arbeitsbedingungen von AnästhesistInnen analysiert haben. Anschliessend wird auf einige kritische Faktoren der Arbeitsbedingungen eingegangen, wie zum Beispiel Zeit- und Produktivitätsdruck.

8.1. Arbeitsbedingungenen von AnästhesistInnen ausserhalb Österreich

Eine Umfrage bei belgischen AnästhesistInnen identifzierte (n=151) berufsspezifische Risikofaktoren, wie zum Beispiel Ausmaß der Kontrolle über die Arbeit und Gefahren bei der Arbeit (NYSSEN et al., 2003, 333-337). Die Arbeitszufriedenheit war hoch, aber 40% waren durch zu hohen emotionalen Streß belastet. AssistenzärztInnen jünger als 30 Jahre waren am meisten gefährdet. In einer österreichischen Single Center Studie wurde ein Viertel aller Antwortenden (n=89) als Burnout gefährdet eingestuft, drei AnästhesistInnen waren in einem klinisch voll ausgebildetem Burnout (KINZL et al., 2006, 2461-2464). Die Altersgruppe mit 31-40 Lebensjahren hatte das höchste Risiko für Burnout. Die folgenden Stressbewältigungsstrategien wurden beschrieben: Erwerb und Erhalt von speziellen technischen Fähigkeiten und kontinuierliche Fortbildung, abwechslungsreiche Arbeit, gegenseitige Wertschätzung unter KollegInnen, körperliche Fitness und intakte soziale Bezieungen ausserhalb des Arbeitsplatzes (KINZL et al., 2006, 2461-2464). Eine weitere Umfrage unter portugiesischen AnästhesistInnen (n=263) berichtete von einer Rate der emotionellen Erschöpfung von 60%, weiters waren 45% nur mehr eingschränkt belastungsfähig, und 90% hatten einen unpersönlichen Umgang mit den PatientInnen. Unzufriedenheit mit den Abläufen innerhalb des eigenen Unternehmens erhöhte die emotionelle Belastung. AnästhesistInnen in leitenden Funktionen waren weniger Stress belastet (MORAIS et al., 2006, 433-439). Eine französische Studie an AnästhesistInenn (n=1.189) welche vornehmlich auf Intensivstationen arbeiteten fand eine Burnout Rate von 46,5%; 51,4%

der Betroffenen plante einen Arbeitsplatz Wechsel im Vergleich zu 39,5% in der gesamten Studienpopulation. Frauen ware überproportional häufig Burnout gefährdet. Eine hohe Arbeitsbelastung (zum Beispiel definiert durch eine hohe Zahl von Nachtdiensten) und Konflikte mit KollegInnen und KrankenpflegerInnen korrelierte mit einer höheren Wahrscheinlichkeit für Burnout (EMBRIACO et al., 2007, 686-692).
Eine weitere französische Studie an Intensivstation PflegerInnen (n=2.525) identifizierte vier Hauptrisikofaktoren für Burnout: 1. Alter 30-40 Jahre; 2. Faktoren im Unternehmen wie zum Beispiel hohe Arbeitsbelastung, hoher Patienten Umsatz und unzureichende Kontrolle des Arbeitsablaufes, 3. Die Unternehmenskultur, zum Beispiel definiert über die Anzahl der Konflikte, 4. Die Notwendigkeit am Lebensende von PatientInnen lebensrelevante Entscheidungen mitzutragen (PONCET et al., 2007, 698-704). Andere Studien kamen zum Schluss, daß die Entscheidungsfreiheit einige Urlaubstage kurzfristig selbst zu planen oder über die Mitarbeit bei klinischen oder wissenschaftlichen Projekten mitzustimmen eine gute Burnout Prophylaxe darstellt. Interssanterweise war die Schwere der Krankheit von PatientInnen nicht mitursächlich für das subjektive Befinden des medizinsichen Personals (EMBRIACO et al., 2007, 686-692; MICHALSEN et al., 2011, 31-38).
Andere Studien kamen zu ähnlichen Ergebnissen (BUDDEBERG-FISCHER et al., 2008, 31-38; CHIRON et al., 2010, 948-958). Interessanterweise kamen die Autoren einiger Studien zum Schluss, das eine hohe Arbeitsbelastung keinen negativen Einfluss hat auf die geistige Gesundheit und die Qualität der Arbeit (FAHRENKOPF et al., 2008, 488-491; PRINS et al., 2009, 654-666; WEST et al., 2009, 1294-1300) und auch nicht auf die körperliche Gesundheit (BUDDEBERG-FISCHER et al., 2008, 31-38).

8.2. Die Entwicklung der Arbeitsbedingungen der AnästhesistInnen

In Deutschland leidet in der Gesamtbevölkerung jeder Dritte unter konstantem Druck (MICHALSEN et al., 2011, 31-38). Der Anteil an Abwesenheitstagen aufgrund psychischer Krankeit hat sich in den letzten zwanzig Jahren von 3,7% auf 10,8% fast verdreifacht (MICHALSEN et al., 2011, 31-38). Zudem dauert ein durchschnittlicher Krankenstand aufgrund einer psychischen Erkrankung mit 22,5 Tagen deutlich länger als bei den meisten anderen Krankheiten (MICHALSEN et al., 2011, 31-38).
In Deutschland verursacht die Arbeitsüberlastung einen volkswirtschaftlichen Schaden in Höhe von ca. sechs Milliarden € pro Jahr (BERTELSMANN, 2013). Zusätzlich lassen bei überlasteten ÄrztInnen die Arbeitsleistung und die Qualität der Arbeit nach. Insofern ist es wichtig das fragile Gleichgewicht zwischen einem qualitativen hochwertigen und einem finanziell leistbaren

Gesundheitssystem zu halten. Die Qualität des Gesundheitssystem kann auf der einen Seite mit den Dimensionen Zugänglichkeit, hohe Qualität und Angebotsbreite und auf der anderen Seite mit Finanzierbarkeit beschrieben werden (Abbildung 14) (SALFELD et al., 2009).

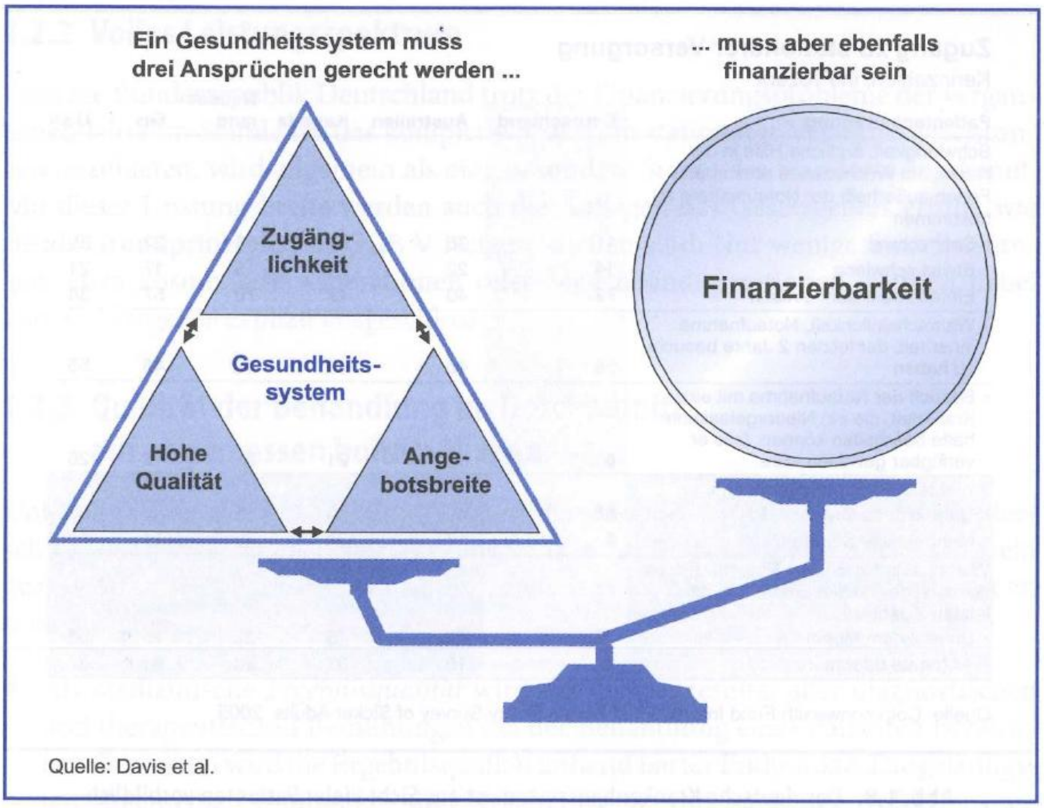

Abbildung 14. Die Qualität des Gesundheitssystem kann auf der einen Seite mit den Dimensionen Zugänglichkeit, hohe Qualität und Angebotsbreite und auf der anderen Seite mit Finanzierbarkeit beschrieben werden (SALFELD et al., 2009).

Die Arbeitsbedingungen und die Gesundheit der Mitarbeiter haben Manager in vielen Unternehmen seit vielen Jahrzehnten beschäftigt. Viele positive Verbesserungen wurden im Laufe dieser Zeit durchgeführt. Man denke zum Beispiel in Deutschland des 19. Jahrhundert an die Vorreiter unter den Großindustriellen WERNER VON SIEMENS (1850er Jahre: Einführung von erfolgsabhängigen Tantiemen; 1872: Pensions-, Witwen- und Waisenkasse; 1873: Neun-Stunden-Arbeitstag), und ROBERT BOSCH (1906: Acht-Stunden-Arbeitstag; gerechte Bezahlung; viele Stiftungen für soziale Zwecke) und im 20. Jahrhundert an Unternehmer wie Götz Werner (dm Drogeriemarkt; konstant hohe Mitarbeiterzufriedenheit), sowie im 21. Jahrhundert in den USA die Google Inc. Gründer SERGEY BRIN und LARRY PAGE (bekannt sind die großen Freiheiten für Mitarbeiter inkl. Sporteinrichtungen und Funparks) (BEER, 2011; KANTER, 2012, 26-39).
Bis vor wenigen Jahren standen die Arbeitsbedingungen von ÄrztInnen im Allgemeinen und AnästhesistInnen im Speziellen nicht im Blickfeld der öffentlichen und wissenschaftlichen Diskussion. Die „Götter in Weiß“ waren hoch angesehen, und eine Aufnahme in diesen

Berufsstand kam einem Ritterschlag gleich. Der Andrang auf Ärztestellen war groß. Was dabei übersehen wurde, war dass die Arbeitsbedingungen aufgrund der reichlichen Bewerber und der im Verhältnis zu wenigen freien Arbeitsplätze kontinuierlich schlechter wurden: Die Bürokratie nahm zu, die Autonomie und das Gehalt ab. Innerhalb der letzten zehn Jahre haben sich aber die Zeiten von zu viel Medizin Absolventen und zu wenigen freien Arbeitsplätzen in das drastische Gegenteil gewandelt. Knappere öffentliche Etats, teils ineffiziente Krankenhausbetriebe, Mehrgleisigkeiten, und ein immer eklatanterer Personalmangel im ärztlichen und pflegerischen Bereich haben zu einer abnehmenden Bewerberzahl und einer Verschärfung der Arbeitsintensität geführt. Dem „Gott in Weiß“ wurden die Daumenschrauben immer mehr angezogen (BAKKER et al., 2000, 884-891). Zudem wird die Medizin deutlich weiblicher: Der Frauenanteil bei den 55+- jährigen beträgt zum Beispiel 24%, bei den bis 35-jährigen hingegen bereits 63% (BAKKER et al., 2000, 884-891). Viele weibliche aber auch zunehmend männliche ÄrztInnen fordern andere Arbeitszeitmodelle um Beruf und Familie kombinieren zu können (zum Beispiel Karenz und Elternteilzeit). Sie erheben aber dennoch auch Anspruch auf Karriere inkl. Erlangung von Führungspositionen. Fast zwei Drittel der befragten ÄrztInnen können sich mit den aktuellen Bedingungen eine Tätigkeit im Krankenhaus bis zur Pensionierung nicht vorstellen (ANONYM, 2013, 20-21).

In Deutschland reagierte der Marburger Bund (Verband der angestellten und beamteten Ärztinnen und Ärzte Deutschlands e.V.) ab 2009 auf die schlechten Arbeitsbedingungen mit Streiks und konnte 2011 den Abschluss besserer Arbeitsverträge erzielen. In Österreich regt sich letzthin in ähnlicher Weise der Widerstand der Klinikärzte (NINDLER, 2013). Tragischer Höhepunkt der Verteilungskämpfe in Österreich war der Selbstmord eines Poolratsleitenden Oberarztes an der Innsbrucker Universitätsklinik (MARKARITZER, 2009). Faktum ist, dass die Arbeitsbedingungen und die Verträge vieler Klinikärzte in Österreich international nicht mehr konkurrenzfähig sind. Zum Beispiel konnte die Ärztegeneration in den 1970er Jahren mit einem Monatsgehalt noch ein Kleinauto kaufen. Heutzutage ist das Monatsgehalt eines Assistenzarztes mit einer 40 Stundenwoche an der Universitätsklinik Innsbruck ca. 1.500€ netto, und mit drei Nacht- oder Wochenenddiensten ca. 2.200€ netto. Im Vergleich dazu verdient ein Assistenzarzt an der Universitätsklinik Regensburg mit drei Diensten 3.500€ netto, die Immobilienpreise sind in Regensburg zudem ca. 20-30% (ANONYM, 2013). Natürlich ist die Bezahlung nur der offensichtlichste Teil der Wertschätzung eines Mitarbeiters. Andere Arten der Wertschätzung schließen zum Beispiel ein Ausbildungsmöglichkeiten und –finanzierung, Anerkennung der Leistungen, Arbeitsklima, Karrieremöglichkeiten und Eingehen auf Vorschläge seitens der Mitarbeiter.

Anästhesisten arbeiten im Schnittfeld von Operationssaal, Notfallmedizin und Intensivmedizin. Sie koordinieren die Betreuung eines Patienten rund um die Operation, von der Aufklärung der Narkose über die Narkose im Operationssaal mit Aufrechterhaltung der lebenswichtigen Funktionen und führen auch die postoperative Nachbetreuung auf der Aufwach- oder Intensivstation durch (Abbildung 15).

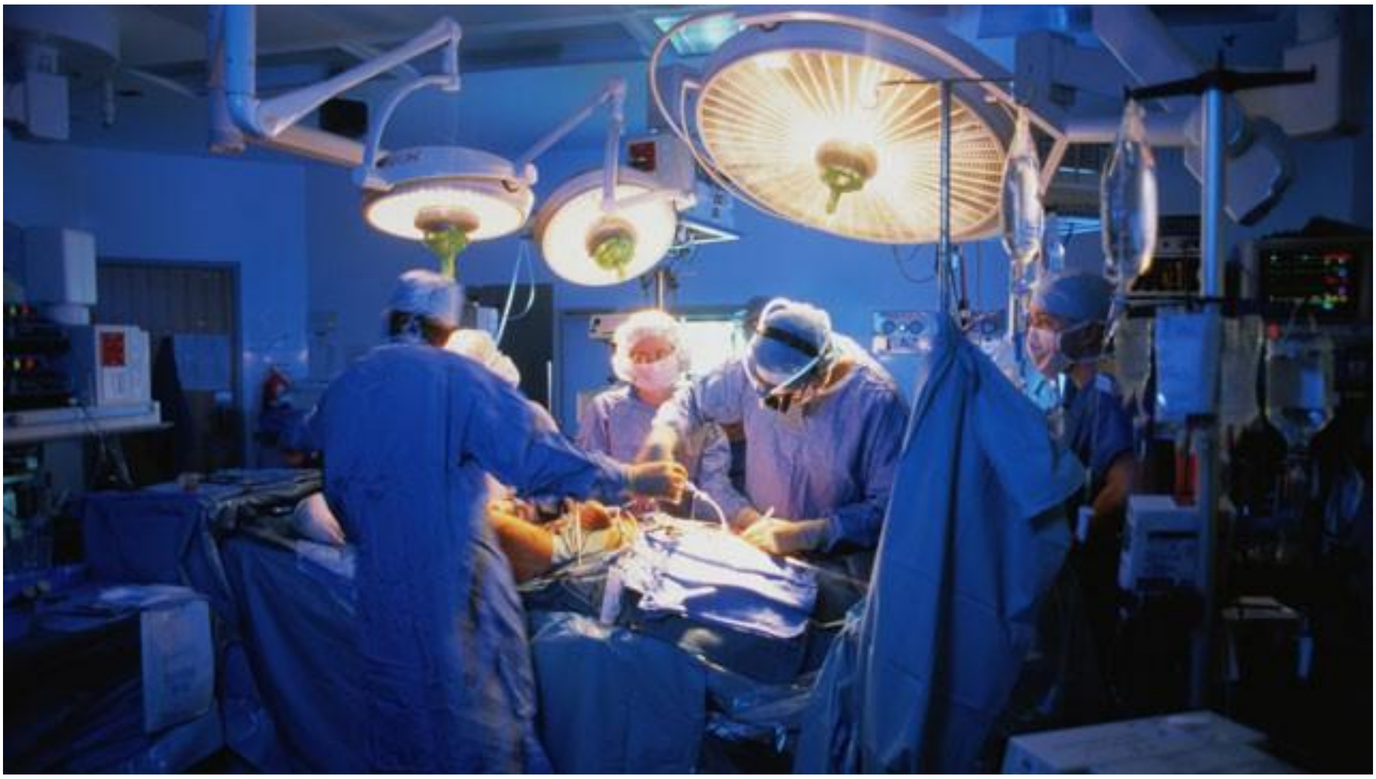

Abbildung 15. Das Chirurgie Team und der Anästhesist bei einem Eingriff im Operationssaal. Der Chirurg ist augenscheinlich im Zentrum des medizinischen Geschehens, der Anästhesist steht abseits des Operationsfeldes hinter dem Operationsvorhang (ABC, 2013).

Häufig stehen AnästhesistInnen im Konflikt mit Chirurgen, die PatientInnen operieren möchten die zu krank für eine Operation sind oder wo schlicht keine Kapazitäten bestehen den Eingriff zum vom Chirurgen geplanten Zeitpunkt durchzuführen. Es ist deshalb nicht verwunderlich dass AnästhesistInnen aufgrund ihres beruflichen Streß häufig geistige und körperliche Gesundheitsprobleme aufweisen. Hohe Depressions-, Scheidungs- und Suizidraten belegen dies.

8.3. Kritische Bereiche bei den Arbeitsbedingungen der AnästhesistInnen in Österreich

Die Ergebnisse der Studie zeigen einige wesentliche Aspekte auf, welche die Arbeitsbedingungen von AnästhesistInnen in Österreich einschränken:

Erstens, empfinden viele AnästhesistInnen dass KollegInnen anderer Fachdisziplinen und die Bevölkerung nur eine neutrale bis geringe Wertschätzung Ihnen gegenüber erbringen. Es gibt keine Daten welche zeigen, dass dieses Gefühl einen Wahrheitsgehalt hat. Marketing und Branding Maßnahmen (zum Beispiel prä- als auch postoperative Visiten der PatientInnen und das Austeilen von Visitenkarten an die PatientInnen bei der präoperativen Visite) erhöhen die Wahrnehmung und damit auch die Wertschätzung bei KollegInnen anderer Fachbereiche und in der Bevölkerung (JESKE et al., 2001, 1262-1264).
Zweitens, die intensive Beanspruchung durch viele Arbeitsstunden schränkt das private Wohlbefinden aber auch die soziale Interaktion ein. Viele AnästhesistInnen beklagen dass ihnen ausreichend Zeit zum Essen fehlt. Die ist ein Unding, da entsprechend dem Zwei Faktoren Modell von Frederick Herzberg Zeit für sich, die Familie und Freunde sowie für das Essen Grundbedürfnisse des Menschen sind (HERZBERG et al., 1959; HERZBERG, 2003).

**Hygienefaktoren (in der Organisation)**	**Motivationsfaktoren (bei der Arbeit)**
Arbeitsbedingungen	Anerkennung
Lohn	Die Arbeit selbst
Sicherheit	Erfolg
Status	Fortschritt
Supervision	Verantwortung
Unternehmensphilosophie	Wachstum

Tabelle 3. Hygiene and Motivationsfaktoren, adaptiert von (HERZBERG et al., 1959)

Lösungsansätze könnten sein, dass man mehr Aufmerksamkeit auf die Einhaltung der Pausen lenkt und dass zur Einhaltung der Pausen AnästhesistInnen abgestellt werden welche ihre KollegInnen zu den Pausen auslösen.
Drittens, geben viele AnästhesistInnen an, dass sie zu lange arbeiten. Zahlreiche Studien belegen dass die wöchentliche Arbeitszeit eines vollbeschäftigten Anästhesisten häufig 50 in einigen Fällen auch 80 Stunden überschreitet. Der Weg von zu wenig Zeit für Privates, hin zu sozialer Verarmung, geistiger und körperlicher Krankheit und schlussendlich dem Vollbild eines Burnouts liegt auf der Hand. Möglichkeiten gegen ein Burnout sind in Tabelle 4 wiedergegeben.

Einführungs- und Trainingsprogramm für neue Mitarbeiter
Klärung der Aufgabenbereiche, der Anforderungen und der Hierarchie
Gelegentlicher Wechsel des Arbeitsbereiches
Begrenzung der Arbeitszeit- sowohl in Bezug auf den Arbeitstag als auch auf die Urlaubsplanung
Pflege von Kollegialität und kollegialem Austausch
Stiftung und Pflege von „corporate identity“
Regelmäßige Fortbildung , sowohl interne als auch externe
Ausreichende Personalstärke
Adäquate Gratifikation
Erhaltung der geistigen und körperlichen Fitness

Tabelle 4. Präventionsmaßnahmen gegen Burnout, adaptiert aus (MICHALSEN et al., 2011, 31-38)

Maßnahmen gegen die angesprochenen exzessiven Arbeitszeiten könnten sein: strikte Einhaltung der geplanten Arbeitszeiten, Beschränkung der zusätzlichen Aufgaben neben dem klinischen Alltag, Anhebung des Grundgehaltes sodass AnästhesistInnen nicht gezwungen sind zahlreiche Nacht- und Wochenenddienste zu arbeiten um einen angemessenen Lebensstandard zu haben (Abbildung 16, HILLERT et al., 2007).

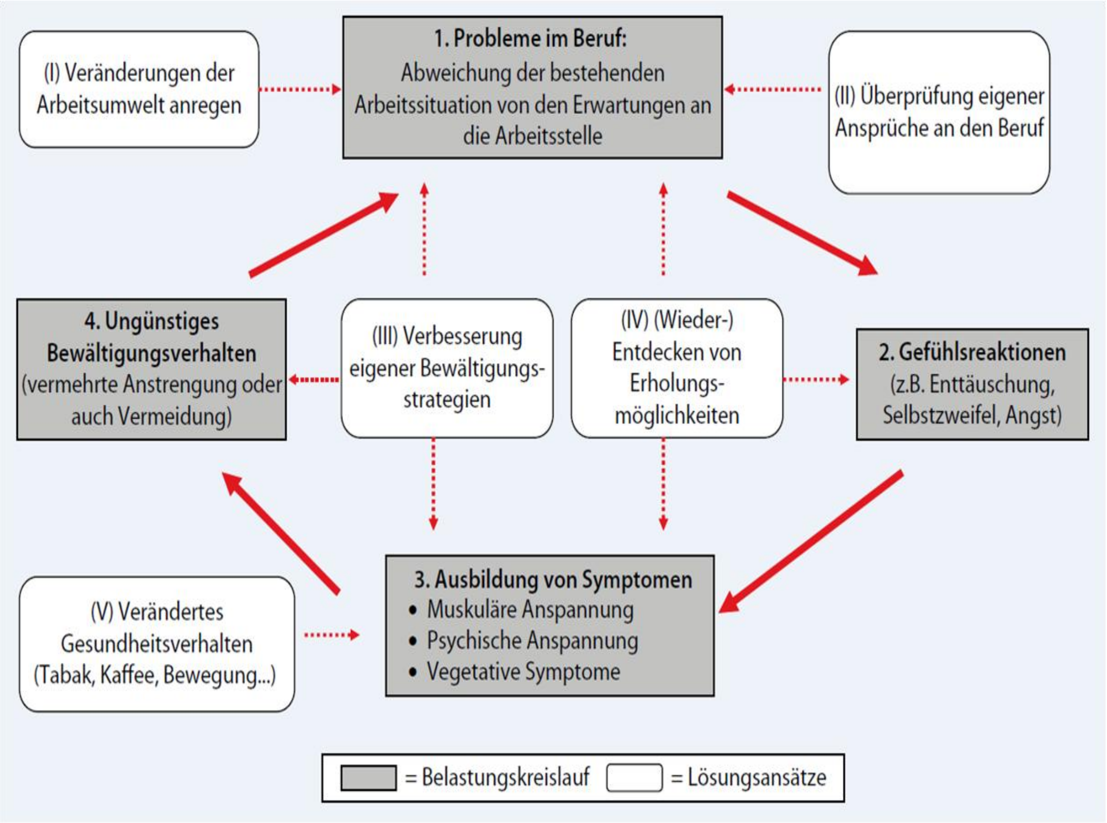

Abbildung 16. Belastungskreislauf und Entlastungsmodell bei Burnout, (HILLERT et al., 2007)

In diesem Zusammenhang ist erwähnenswert dass jüngere Generationen wie zum Beispiel die Generationen X, Y und Z nicht mehr gewillt sind so viele Wochenstunden wie die Nachkriegs- und der Babyboomer Generation zu arbeiten. Eine typische Aussage der jüngeren Generationen ist “Die Familie kommt vor dem Lohn” (OTTENSCHLÄGER, 2013, 89). Weiters wird die Medizin immer weiblicher. Zum Beispiel sind mittlerweile bei den Medizin-StudentInnen 60% Frauen, der weibliche Anteil unter den AssistenzärtzInnen ist ähnlich hoch (DERKZEN et al., 2013, 27; OTTENSCHLÄGER, 2013, 89). Um die Anästhesiologie für Frauen attraktiver zu gestalten müssen Kliniken noch mehr als bisher Anstrengungen unternehmen um die Verbindung von Familie und Beruf zu ermöglichen. Öffentliche Krankenhäuser hinken in diesen Belangen vielen privaten Krankenhäusern hinterher. Möglichkeiten zur Attraktivitätssteigerung der Arbeitsplätze könnten sein: flexiblere Arbeitszeiten (zum Beispiel angepasste Früh- und Spätschichten), flexible Karenzzeiten für Frauen und Männer, Teilzeitmodelle für Frauen und Männer, Arbeitsplatzteilung (job sharing) für Führungspositionen.

Zudem sollten einige Krankenhausverbunde die Gehälter an ein international kompetitives Niveau anheben um ausreichend junge Arbeitskräfte anzuziehen und ältere KollegInnen zu halten (SALFELD et al., 2009; NINDLER, 2013) (Abbildung 17).

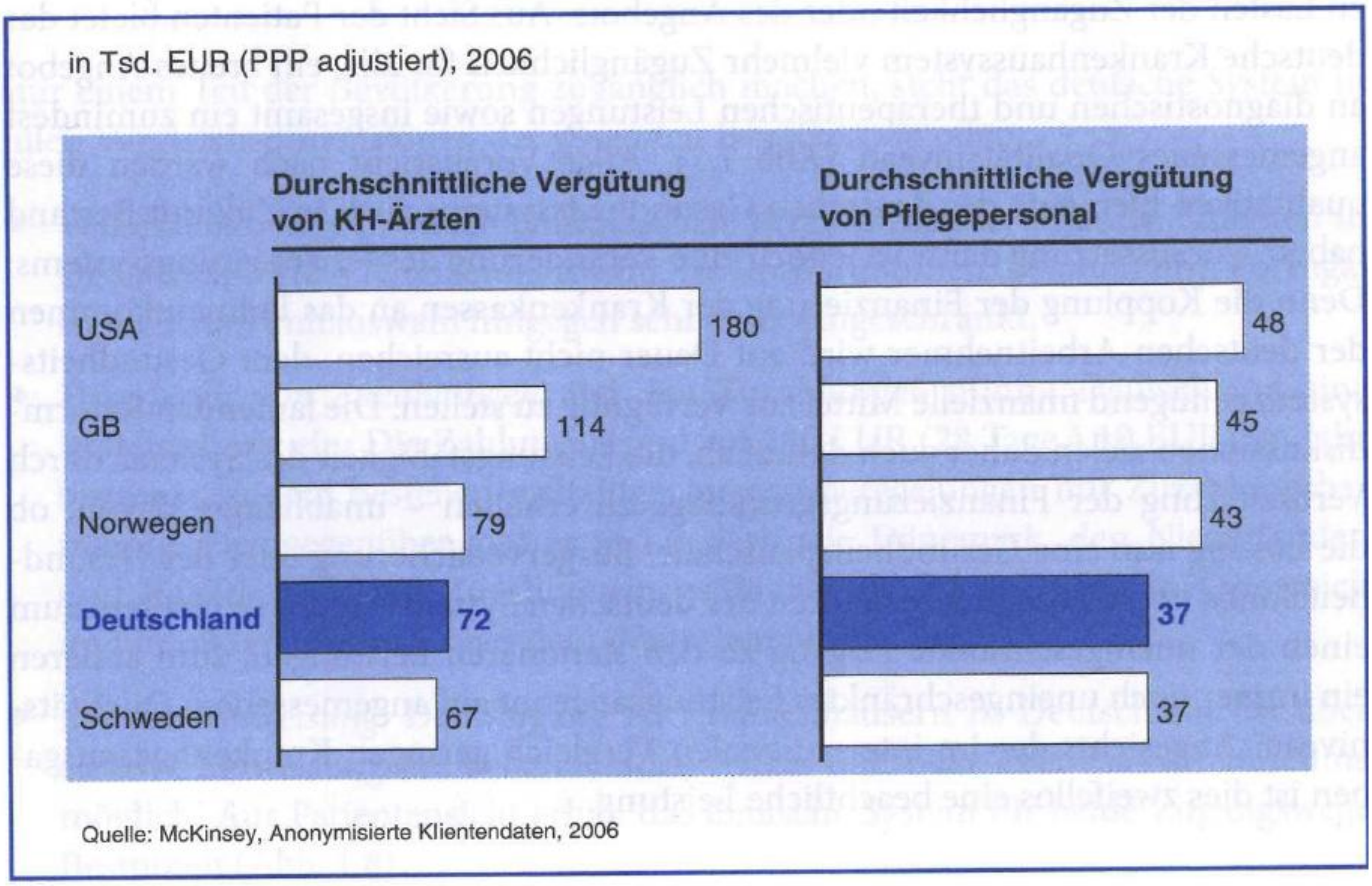

Abbildung 17 zeigt Gehälter von KrankenhausärtzInnen und Pflegekräften in verschiedenen Ländern. Österreichische ÄrztInnen werden ähnlich gezahlt wie ihre deutschen KollegInnen; die TILAK zahlt im österreichischen Schnitt vor allem Jüngeren deutlich weniger (SALFELD et al., 2009).

Viertens gibt es bei den AnästhesistInnen in Österreich alarmierend häufig Zeichen für geistige und körperliche Belastung und Erkrankung. Zahlreiche AnästhesistInnen haben angegeben dass sie Muskelschmerzen verspüren und an Rückenproblemen leiden. Die geistige und körperliche Schwäche äussert sich bei vielen aufgrund einer leicht Ermüdbarkeit und einem verminderten sexuellen Interesse und Vergnügen. Initiativen sollten ergriffen werden damit AnästhesistInnen mehr Zeit haben in ihrer Freizeit entspannenden Hobbies wie zum Beispiel soziale Interaktion und Sport nachzugehen. Sport Initiativen durch Krankenhäuser, Ermässigungen beim Besuch von Sportanlagen oder beim Erwerb von Sportartikeln könnten im Sinne eines Cafeteria Systems helfen. Die wichtigste aller Maßnahmen ist aber den AnästhesistInnen ein Arbeitsumfeld zu geben, das Ihnen die Einhaltung ausreichend langer Erholungszeiten ermöglicht. Zudem sollte zur Stärkung der Gesundheit gesundes Essen im Krankenhaus angeboten werden, zum Beispiel Gemüse und Obst auch in den entlegenen aber

häufig frequentierten Räumlichkeiten eines Klinikareals, da AnästhesistInnen oft nicht ausreichend Zeit haben die Mensa aufzusuchen.

Als *letzter und fünfter Punkt* sticht heraus, dass AnästhesistInnen unter Druck stehen produktiver zu arbeiten. Neuere Vergleichsuntersuchungen konnten ein eklatantes Produktivitätsgefälle zwischen einzelnen Krankenhäusern feststellen (Abbildung 18).

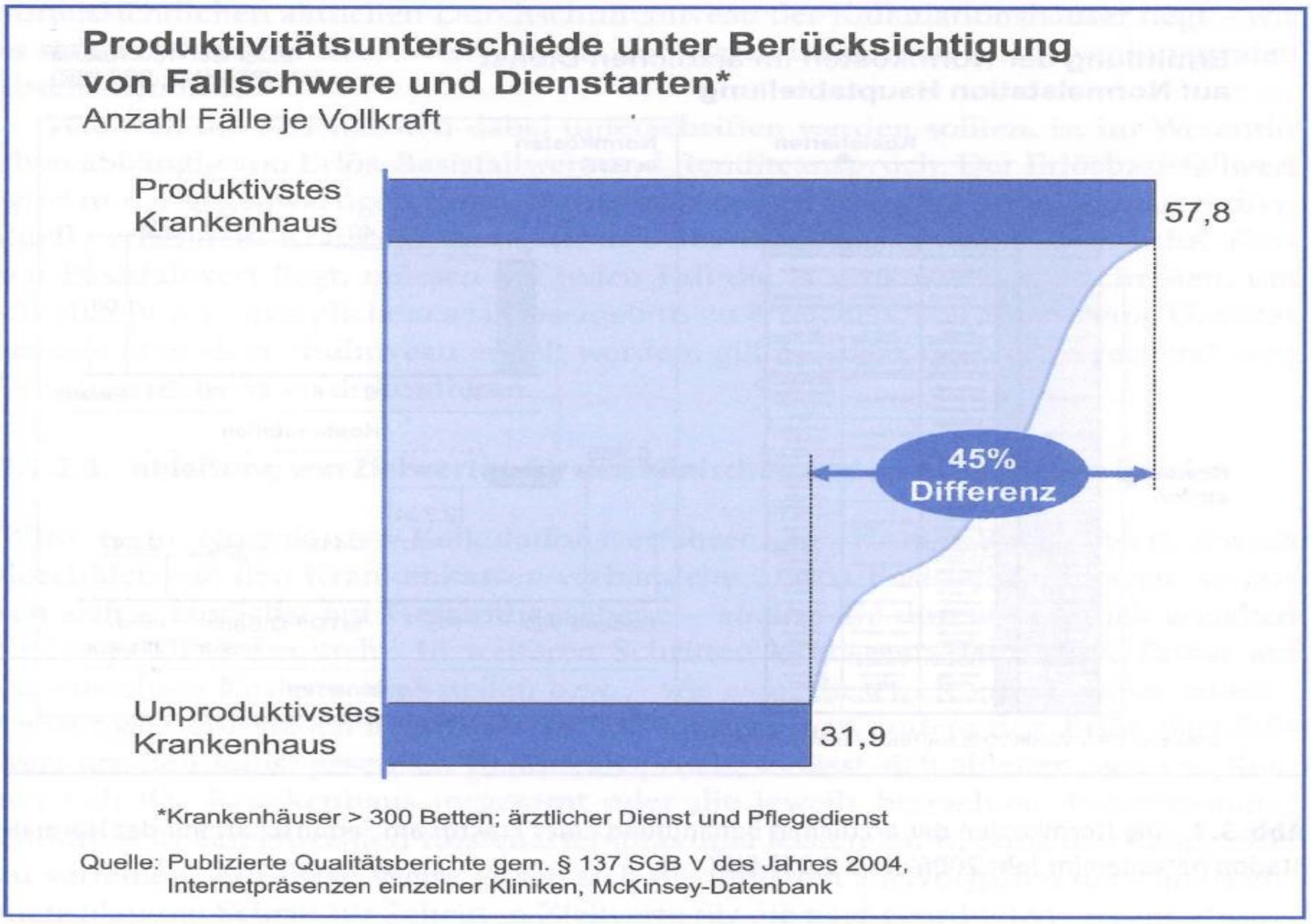

Abbildung 18. Produktivitätsunterschiede in verschiedenen Krankenhäusern unter Berücksichtigung der Fallschwere und der Dienstarten. Für Krankenhäusern mit mehr als 300 Betten) (SALFELD et al., 2009).

Insofern erscheint es sinnvoll in einigen Krankenhäusern die Produktivität mit Rücksicht auf die lokalen Bedingungen zu steigern, in anderen Krankenhäusern mit bereits sehr hoher Produktivität kann eine weitere geplante Produktivitätssteigerung evtl. zu einer Überlastung von AnästhesistInnen führen. Zum Beispiel verspüren viele AnästhesistInnen dass in ihrem Krankenhaus bereits eine hohe Produktivität herrscht. Ein kontinuierliches Benchmarking mit anderen Krankenhäusern sollte hierbei hilfreich sein. Interessanterweise stufte eine aktuelle

internationale Studie das Personal in den österreichischen Krankenhäuser bereits unter den produktivsten der untersuchten Länder ein (Abbildung 19).

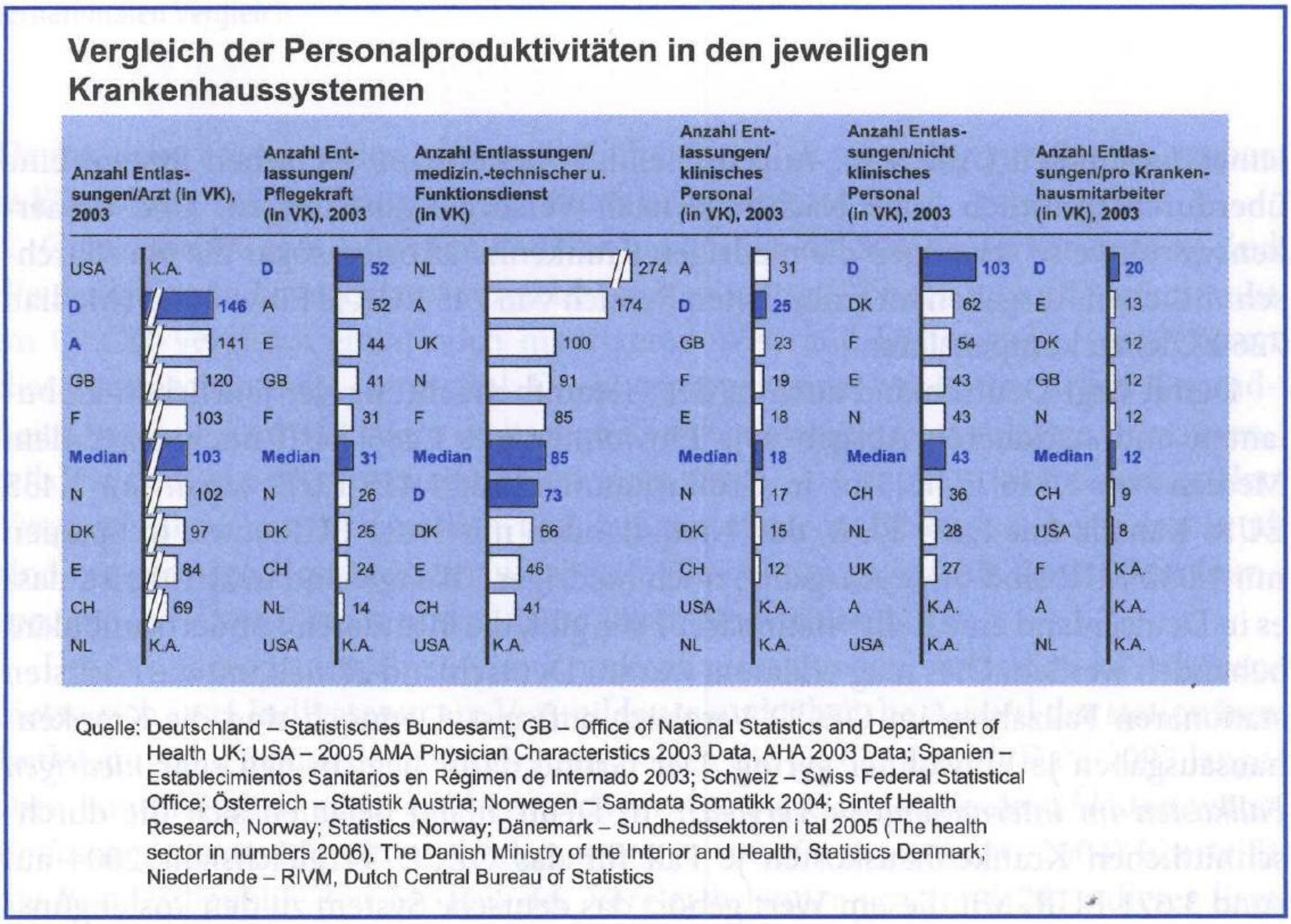

Abbildung 19. Produktivität des Personals in verschiedenen nationalen Krankenhaussystemen) (SALFELD et al., 2009).

Zu viel Produktivitätsdruck kann das Gegenteil von Leistungsteigerung und Kosteneinsparung bewirken wenn nämlich oberflächliches Arbeiten, Überarbeitung und Erschöpfung die Folge sind. Die Arbeitsqualität kann unter dem Produktivitätsdruck und der Überarbeitung der AnästhesistInnen leiden und Fehler können gehäuft auftreten, zum Teil mit Todesfolge (SHANAFELT et al., 2002, 358-367; FAHRENKOPF et al., 2008, 488-491; PRINS et al., 2009, 654-666; WEST et al., 2009, 1294-1300; DE OLIVIERA et al., 2013, 182-193). Möglichkeiten um Überarbeitung zu vermeiden können Standard Operating Procedures für häufige Krankheitsbilder (zum Beispiel anästhesiologisches Vorgehen bei geplanten Operationen bei Hüftfraktur oder Leistenbruch), Anästhesie Ein- und Ausleitungszonen mit eigenen Teams welche zu schnelleren Umlagerungszeiten in den Operationssälen beitragen können (zum Beispiel sind damit Wechsel von einer Operation zur nächsten in ca. 15 Minuten anstatt ca. 45 Minuten möglich).

9. Schlussfolgerungen aus der Beantwortung der forschungsleitenden Fragen

Im Folgenden sollen im Lichte der Ergebnisse der aktuellen Umfrage die zwei forschungsleitenden Fragen beantwortet werden. Da die Antworten komplex sind und Einfluss auf die Arbeitsbedingungen von AnästhesistInnen in Österreich und darüber hinaus haben können werden die Ergebnisse im Lichte der aktuellen Literatur diskutiert.

9.1. Anhand welcher Merkmale können die Arbeitsbedingungen der AnästhesistInnen in Österreich am zweckmäßigsten beschrieben werden?

Die Arbeitsbedingungen von AnästhesistInnen können wie folgt am zweckmäßigsten beschrieben werden:

- *Das Ansehen des Berufsstandes erscheint niedrig*: AnästhesistInnen erachten das Ansehen, das sie bei KollegInnen anderer Fachdisziplinen und in der Bevölkerung genießen als neutral bis gering. Möglichserweise vermuten AnästhesistInnen deswegen ein niedriges Ansehen bei KollegInnen anderer Fachdisziplinen da die Abhängigkeit von den KollegInnen der chirurgischen Fächer hoch ist. Zum Beispiel können AnästhesistInnen im Vergleich zu KollegInnen anderer Fachdisziplinen in der Regel PatientInnen nicht heilen, vielmehr führen AnästhesistInnen Anästhesien aus und arbeiten in der Notfall- und Intensivmedizin damit andere FachärztInnen die heilende Intervention- üblicherweise Operation- durchführen können.
- Der berufsbedingte *Zeitmangel und Arbeitsdruck machen krank:* Häufig haben AnästhesistInnen zu wenig Zeit für sich selbst, die Familie und Freunde. Manchmal fehlt ausreichend Zeit zum Essen. Manchmal sind die Arbeitszeiten zu lange und oft fehlt ausreichend Zeit zum Ausruhen und Schlafen. Oft leiden AnästhesistInnen an Ein- und Durchschlafstörungen, Muskelverspannungen und Rückenschmerzen. Die Zeit die Arbeit zu verlassen beträgt in der Regel Null bis zehn Minuten. Zeitdruck ist häufig (>90%); oft kommen AnästhesistInnen verspätet in die Pausen und spät nach hause. Das Arbeitstempo ist oft hoch. Die Arbeit wird häufig durch Vorgesetzte, KollegInnen, PatientInnen und weil Wichtiges dazwischen kommt unterbrochen. Häufig werden mehrere Arbeiten gleichzeitig durchgeführt. Die Arbeit ist mental komplex und fordernd, man muss sofort reagieren. Oft muss man viele und schwer zu merkende Dinge im Kopf behalten. Wechselnde Arbeitsschichten sind häufig.
- *Die Arbeit ist anspruchsvoll und macht viele krank**:*** Bei der Arbeit sind viel Überlegung, Aufmerksamkeit und die Beherrschung diverser Materialien gefordert. Der Einfluss auf viele Arbeitsprozesse ist gering aber bei der Durchführung der zugeteilten Arbeit besteht hohe Autonomie. Oft haben AnästhesistInnen Probleme mit der Aussprache über Gefühle und

Probleme. Selten sind sie ganz entspannt. Oft sind sie ruhelos und ungeduldig. Oft haben sie das Gefühl dass der Tag nicht gut wird, 10% sind gar oft oder sehr oft traurig und niedergeschlagen.

- *Die Arbeitszufriedenheit, Leistungsmotivation und Commitment werden durch Unternehmen selten optimal gefördert.* Die Sorge die Arbeit zu verlieren ist gering. Einige glauben für ihre Arbeit gut bezahlt zu werden (25%). Wenige (30%) stimmen zu dass Karrierechancen gut sind. 45% fühlen sich im Unternehmen nicht zuhause und 40% haben keine guten Freunde im Unternehmen. 50% stimmen zu dass es einfach ist einen anderen Arbeitsplatz mit ähnlicher Bezahlung zu finden. 40% meinen dass das Unternehmen sie nicht motiviert die beste Leistung zu bringen. Dennoch sind Fehlzeiten selten, und AnästhesistInnen sind häufig trotz Krankheit bei der Arbeit.

9.1.1. Der Durchschnittsanästhesist

Um die Arbeitsbedingungen bildlich darzustellen bietet es sich an aus den meistgenannten Antworten einen fiktiven Standard Anästhesisten für Österreich zu erstellen: Der Durchschnittsanästhesist ist 30-40 Jahre alt, verheiratet und Vater. Er ist Facharzt, ohne leitende Funktion, arbeitet seit über 10 Jahren im gleichen Krankenhaus, indem mehr als 10.000 Anästhesien pro Jahr durchgeführt werden. Seiner Ansicht nach haben Ärzte anderer Fachrichtungen eine neutrale Wertschätzung seinem Fach gegenüber, während er die Wertschätzung in der Bevölkerung niedrig erachtet. Glücklicherweise ist das Arbeitsklima an seinem Arbeitsplatz zufriedenstellend.

Manchmal hat er nicht genug Zeit für sich und oft hat er nicht genug Zeit für seine Partnerin, seine Kinder und Freunde. Manchmal fehlt ihm die Zeit zum Essen, und er erachtet die Arbeitsstunden die er leistet als zuviele. Die Zeit die er benötigt um zum Arbeitsplatz zu gelangen ist kurz, aber oft hat er nicht genug Zeit zum Schlafen.

Manchmal hat er Kopfschmerzen und Probleme ein- und durchzuschlafen. Oft hat er Muskelverspannungen und Rückenschmerzen, aber er leidet nie an Magenschmerzen, Kurzatmigkeit, Jucken oder Durchfall. Gelegentlich ist er matt und schwindlig, und hat Schwierigkeiten sich an Sachen zu erinnern. Er hat nie Probleme mit Herzrasen, Brustschmerzen, Verstopfung, trockenem Mund und Schlucken, oder Appetitmangel. Manchmal wird er schnell müde und hat ein reduziertes sexuelles Interesse und Vergnügen am Sex.

Oft geniesst er sein Leben und geht Probleme aktiv mit neuen Lösungsmöglichkeiten an. Manchmal fühlt er sich unzufrieden und angespannt und manchmal versucht er Dinge zum Besseren zu verändern. Selten hat er Schwierigkeiten über seine Gefühle zu sprechen, aber

selten zeigt er nahestehenden Personen seine Gefühle wenn er niedergeschlagen ist. Selten hat er Schwierigkeiten sich zu beruhigen wenn er aufgebracht war. Selten nur fühlt er sich ganz entspannt. Wenn er am Morgen aufsteht hat er oft das Gefühl dass es ein guter Tag werden wird.
Oft ist die Arbeit komplex, er arbeitet mit zahlreichen verschiedenen Geräten und autonom, aber er kann nur in sehr geringem Mass die Dauer seiner Arbeit beeinflussen. Bei der Arbeit ist die Pause in der Regel maximal 15 Minuten. Selten bekommt er von seinem Vorgesetzten unklare Anweisungen. Die Arbeit bietet nur minimale körperliche Abwechslung. Er wird mehrmals die Woche von seinen Mitarbeitern bei der Arbeit gestört. Oft muss er im Laufe der Woche mehrere Aufgaben gleichzeitig erledigen, muss komplexe Zusammenhänge im Kopf behalten und muss gezielt reagieren. Er erachtet das Arbeitstempo als hoch. Zeitdruck ist viele Male die Woche ein Thema und er versucht of schneller zu arbeiten. Jeden Tag ist er mit sechs und mehr Kollegen sowie mehr als 10 Patienten in Kontakt. Er hat keine Schwierigkeiten mit KollegInnen zu sprechen.

9.2. Welche vorläufigen Schlussfolgerungen für die Organisationsgestaltung im Krankenhausbereich können aus dieser Bestandsaufnahme abgeleitet werden wenn man dafür Kriterien wie Arbeitszufriedenheit, Leistungsmotivation und Commitment heranzieht?

In der folgenden Betrachtung soll der Einfluss der Organisationsgestaltung im Krankenhaus analysisert werden. Besonderer Wert wird auf die Analyse des Einflusses der Organisationsgestaltung auf die Kriterien Arbeitszufriedenheit, Leistungsmotivation und Commitment gelegt

9.2.1. Der Einfluss von Arbeitsmotivation und Führungsstil auf Leistungsmotivation und Commitment

In einem lesenswerten Buch hat STAHL die Arbeitsmotivation in Organisationen aus den vier Perspektiven Psychologie, praktische Philosphie, Neurobiologie und Management Theorie beleuchtet (STAHL, 2013b). Die Psychologie Perspektive diskutiert den Ursprung der Motivation im Allgemeinen und anschliessend im Speziellen. Die Philosophie Perspektive beschäftigt sich mit dem Menschenbild, der Wertedynamik, dem Sinn, der Gerechtigkeit, der Macht und dem Vertrauen. Diese Themen sind mit der Führungskunst eng verknüpft. Die Perspektive der Neurobiologie zeigt neue Erkenntnise über die Funktion verschiedener Hirnregionen und

diskutiert ihre Rolle für die Leistungsmotivation. Schlussendlich beschäftigt sich die Perspektive der Management Theorie mit dem Übergang von einer klassischen zu einer postklassischen Führung. Letztere setzt den Mensch mit all seien Stärken und Schwächen in den Mittelpunkt der Führungsanstrengungen. Postklassische Führung heißt reflektieren über die eigene Persönlichkeit, die Unternehmenskultur, das Menschenbild und das Unternehmen. Der Führungsstil sollte an den einzelnen Mitarbeiter angepasst werden. Auf diese Weise kann man aus Mitarbeitern ein Maximum an Arbeitszufriedenheit, Leistungsmotivation und Commitment mobilisieren.

STAHL führt einige Empfehlungen an um die Etablierung von postklassischem Management zu erleichtern (STAHL, 2013b): Komplexität sollte akzeptiert werden. Kontingenz (die Kunst der Improvisation) und Vielfalt sollten bei den Mitarbeitern gefördert werden. Bei der Auswahl von Mitarbeitern sollte auf die Vielfalt von Interessen und soziale Kompetenz Wert gelegt werden. Mitarbeiter im Zenturm aber auch in der Peripherie des Unternehmens sollten aufmerksam und mit Respekt behandelt werden. Vertrauen sollte im Unternehmen gestärkt werden, dadurch können die Produktivität und das Netzwerken im Unternehmen gefördert werden. Die Vielfalt ethischer Werte bei den Mitarbeitern sollte erlaubt sein und die Organisation sollte sich kontinuierlich dem Zeitgeist anpassen. Führung sollte individualisiert und an den Reifegrad der Mitarbeiter angepasst werden. Informalität sollte erlaubt sein. Fehler sollten in Leistungsgesprächen angesprochen werden, aber es sollte Wert auf den positiven Unterschied, das heisst wie die Situation verbessert werden kann, gelegt werden. Der Fokus sollte liegen auf “Wie kann man es besser machen?” anstatt “Wer hat den Fehler gemacht?”. Fakten sollten in Geschichten verpackt werden, da man Geschichten länger in Erinnerung bleiben. Man sollte sich vor Affekten hüten und zurückhaltend und demütig sein.

Unternehmen sollten Mitarbeiter im Aufbau von Commitment fördern (STAHL, 2013a). Mitarbeiter, welche Commitment für ein Unternehmen zeigen sollten graduell “empowered” werden, das heisst sie sollten in ihrer Ausbildung, geistig und sozial gestärkt werden. Damit können ihnen immer anspruchsvollere Aufgaben übertragen werden (Abbildung 20).

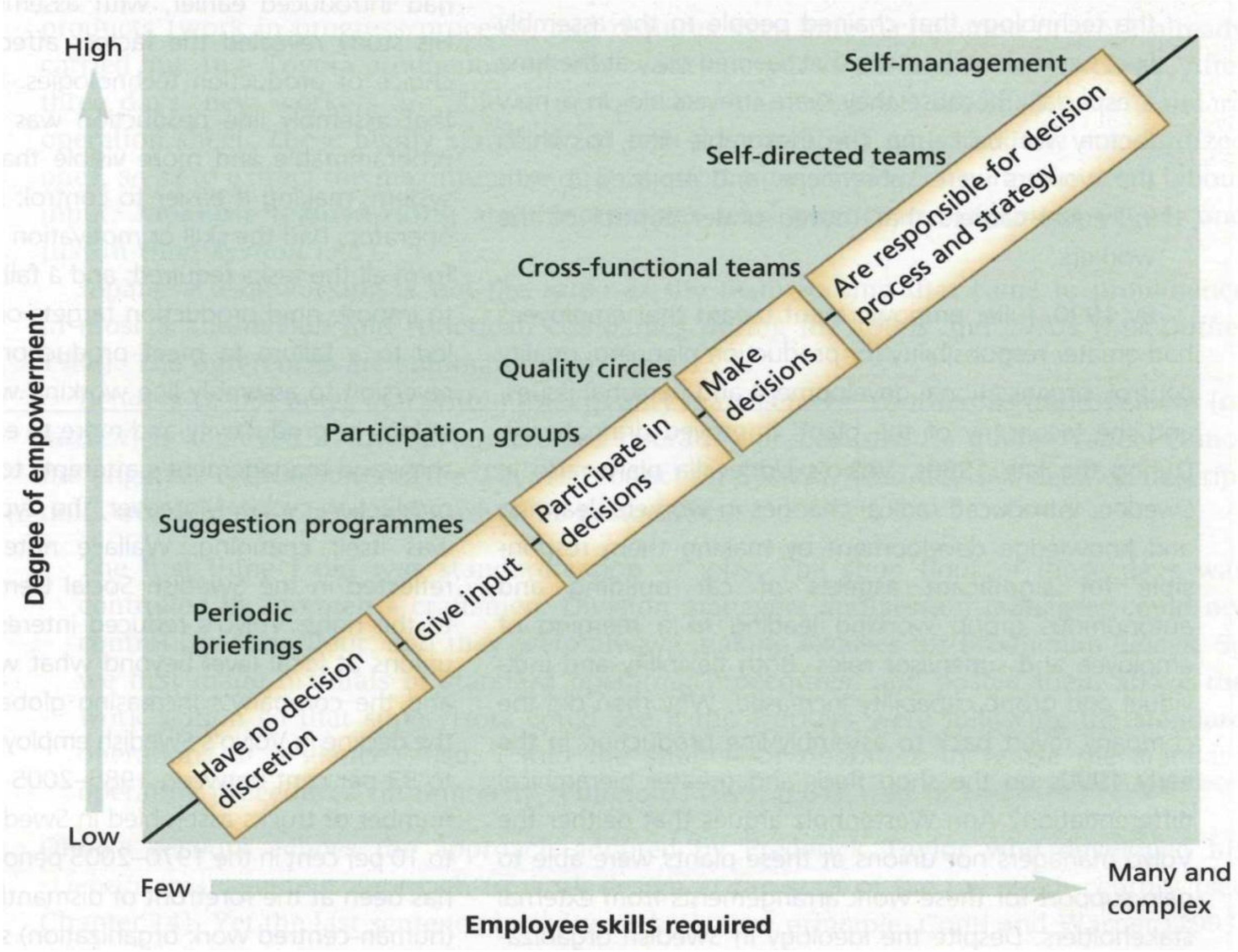

Abbildung 20. Ein Kontinuum des Empowerment (BUCHANAN et al., 2010, 385-416).

Schlussendlich resultiert Flow (eine Balance aus Leistungsanfoderung und Leistungsfähigkeit in der ein Mitarbeiter die Zeit vergisst und optimal arbeitet) (CZIKZSENTMIHALYI, 2004), und ein Unternehmen mit nachhaltigem Erfolg wird etabliert (PIRCHER, 2011). In dieser Diskussion ergibt sich automatisch die- rhetorische- Frage “Warum sollte es sich für einen Krankenhausträger auszahlen sozial zu führen?” Es gibt zahlreiche Gründe die ein soziales Führen nicht nur rechtfertigen sondern sinnvoll machen, adaptiert nach (KANTER, 2012, 26-39):

- Sind Vision und Wertevorstellungen des Krankenhauses definiert führt diese Sicherheit zu Klarheit bei MitarbeiterInnen und PatientInnen. Arbeitszufriedenheit, Leistungsmotivation und Commitment steigen an
- Verstehen Management, MitarbeiterInnen und andere StakeholderInnen ein Krankenhaus als gesellschaftliche Institution denken und handeln sie langfristiger- Weitsicht schafft Nachhaltigkeit.
- Starke instiutionelle Werte motivieren die MitarbeiterInnen.

- Sozial agierende Unternehmen sind angesehener und sind attraktive Arbeitgeber.
- Mit anderen Unternehmen eng interagierende Krankenhäuser inspirieren mehr und sind innovativer.
- In sozial geführten Krankenhäusern ist die Verantwortung des einzelnen größer, Empowerment und damit die Effizienz steigen.

9.2.2. Einfluss der Organisationsgestaltung auf Arbeitszufriedenheit, Leistungsmotivation und Commitment

Krankenhäuser befinden sich zunehmend in einem Spannungsfeld der Ressourcenverknappung inkl. Personalmangel, Anforderungen an die Wirtschaftlichkeit und steigenden Ansprüchen an die Qualität (zum Beispiel durch PatientInnen und im Rahmen von interklinischen Benchmarkings). Dieses Spannungsfeld wird als Magisches Dreieck des Krankenhausmanagements beschrieben (PADOSCH et al., 2011, 364-369) (Abbildung 21).

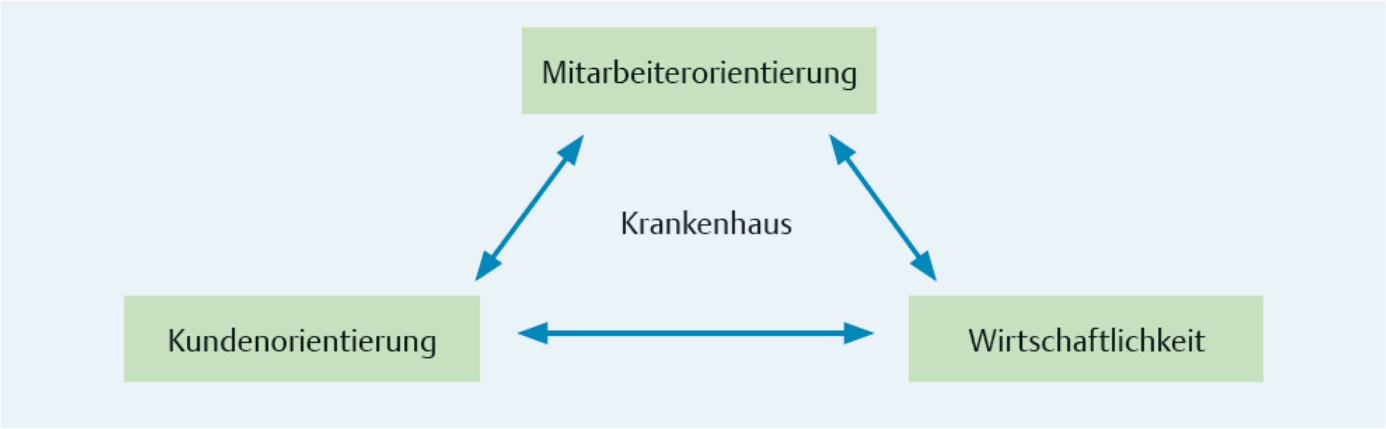

Abbildung 21. Das Magische Dreieck des Krankenhausmanagements.

In diesem Zusammenhang verdient die folgende Tatsache, die von eigenen Beobachtungen gestützt wird, Beachtung. Die Expertenorganisation „Krankenhaus“ zerfällt typischerweise in vier “Welten”, wie sie zum Beispiel im GLOUBERMAN-MINTZBERG-Modell dargestellt werden (Abbildung 22) (GLOUBERMAN et al., 2001, 56-69).

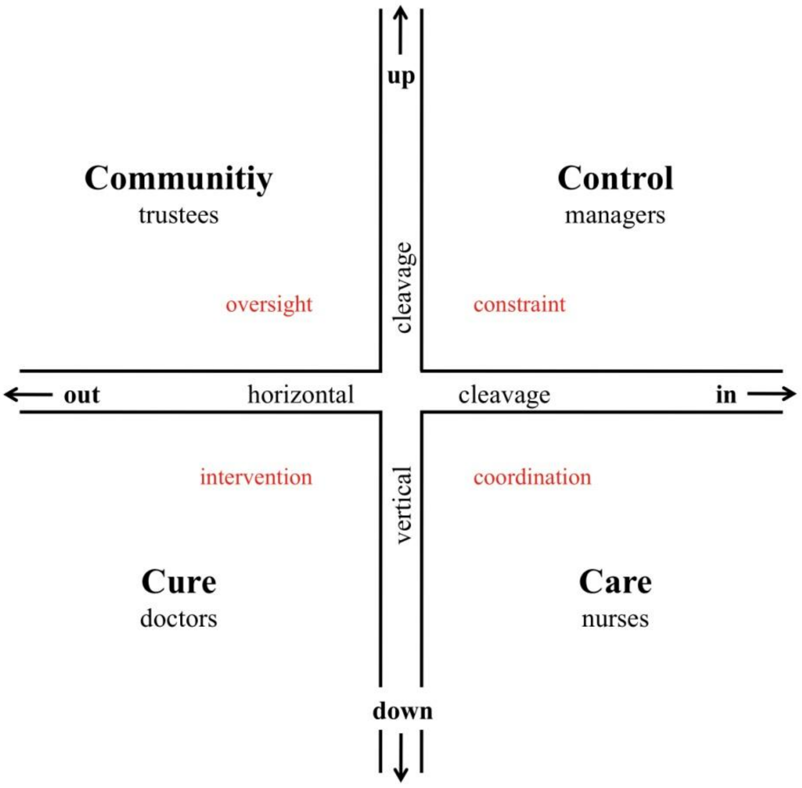

Abbildung 22. Die „Vier Welten“ des Krankenhauses aus (MAYERHOFER, 2013, 326), adaptiert nach (GLOUBERMAN et al., 2001, 56-69).

Die „Vier Welten“ sind im Uhrzeigersinn von links unten in Abbildung 22 startend die ÄrztInnen die heilen (Cure), die PflegerInnen die pflegen (Care), das Management das leitet (Control) und die Krankenhausträger die das Krankenhaus finanziell tragen (Community; zum Beispiel Gemeinden, Land, Bund, private Krankenhausträger). Die „Vier Welten“ des Krankenhauses sind in ihren Interessen voneinander getrennt. Sie agieren oft auch konträr, das wird durch den horizontalen und den vertikale Balken aufgezeigt. Für dieses Modell besonders kritisch ist die Trennung der medizinisch Tätigen (unten im Bild: ÄrztInnen und PflegerInnen; sie arbeiten im Kerngeschäft „Medizin“ des Krankenhauses), von jenen die nicht im Kerngeschäft „Medizin“ tätig sind (oben im Bild: ManagerInnen und Krankenhausträger). Zudem trennt der vertikale Balken jene die ins Krankenhaus orientiert sind (rechts im Bild: PflegerInnen da sie viel Patientenkontakt haben und die ManagerInnen) von jenen mit Aussenorientierung (ÄrztInnen die hauptsächlich nur punktuell beim Patient sind und auch oft ausserhalb des Krankenhauses arbeiten, und die Krankenhausträger).
Durch die unterschiedlichen Interessen der „Vier Welten“ des Krankenhauses entstehen zahlreiche Missverständnisse und Reibereien in der Organisationsgestaltung eines Krankenhauses, die durch parallel aneinander vorbei oder gegeneinander Arbeiten können in einem Krankenhaus viele Ressourcen vernichtet werden. Arbeitsmotivation, Leistungsmotivation

und Commitment von AnästhesistInnen können darunter sehr leiden. Der Autor möchte hierzu für die Universitätsklinik Innsbruck zwei aktuelle Beispiele anbringen:
Erstens gibt es an der Universitätsklinik Innsbruck zwei Krankenhausträger und zwar die Tiroler Landeskrankenanstalten Gesellschaft (TILAK), die ihren Fokus primär in der Patientenversorgung hat, und die Medizinische Universität Innsbruck (MUI), welche primär für Forschung und Lehre zuständig ist. Da an einer Universitätsklinik Medizin aber untrennbar aus den drei Säulen Patientenversorgung, Forschung und Lehre besteht kann man die Aufgaben von TILAK und MUI nicht trennen. Da dies aber von den zwei Krankenhausträgern häufig gewünscht wird leiden die AnästhesistInnen die im Arbeitsalltag den Brückenschlag zwischen klinischer Versorgung der Patienten, Forschung und Lehre versuchen: Sie Können und Wollen, aber zum Teil Dürfen und Sollen sie nicht.
Zweitens zeigen die derzeitigen Arbeitsvertrag Verhandlungen der TILAK ÄrztInnen deutlich wie sich Krankenhausträger und Management miteinander verbünden um den Forderungen der ÄrztInnen geschlossen entgegentreten zu können (mehr dazu in den nächsten Absätzen).
Gloubermann und Mintzberg stellten für die „Vier Welten“ des Krankenhauses treffend fest: „Thus we find four worlds, all necessary components of the system of health care and disease cure yet unnecessarily disconnected – by unreconciled values, incompatible structures, intransigent attitudes. Divisions of labor are necessary – the boundaries are inevitable – but the disconnections are destructive“ (GLOUBERMAN et al., 2001, 56-69).
Im Krankenhaus sind ÄrztInnen Experten, und Experten lassen sich ungern in ihren Bereich von anderen wie zum Beispiel vom Management oder den Krankenhausträgern etwas vorschreiben, da sie in ihrem Fachgebiet den anderen weit überlegen sind und auch dementsprechend auf Änderungswünschen ausweichend argumentieren können (MAYERHOFER, 2013, 326). Es bilden sich im Krankenhaus zwischen den „Vier Welten“ wechselnde Allianzen zur Verteidigung und Durchsetzung von Interessen, wobei die in der Abbildung 23 veritkal und horizontal benachbarten Berufsgruppen koalieren, quere Koalitionen sind sehr selten.

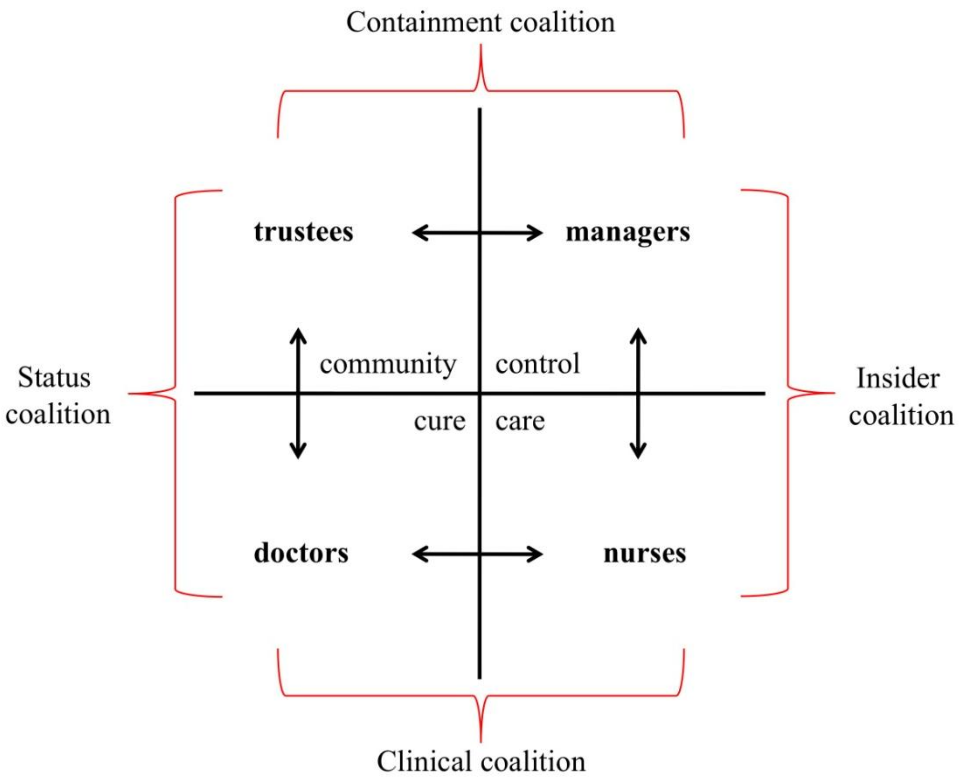

Abbildung 23. Koalitionen zwischen den „Vier Welten“ des Krankenhauses aus (MAYERHOFER, 2013, 327), adaptiert nach (GLOUBERMAN et al., 2001, 56-69).

Diese Spannungen in der Organisationsgestaltun färben negativ auf die Arbeitsbedingungen sowie die Arbeitszufriedenheit, Leistungsmotivation und Commitment von AnästhesistInnen ab. Im Lichte des Besprochenen (zum Beispiel Produktivitätssteigerung, Zeitknappheit, Personalmangel) ist nachvollziehbar, dass ÄrztInnen gegen das Management koalieren, zum Teil mit den PflegerInnen (siehe zum Beispiel die Tendenzen in Österreich Anästhesien durch PflegerInnen durchführen zu lassen wie in Skandinavien, Schweiz und USA) oder mit Krankenhausträgern. Umgekehrt koaliert derzeit das Management eng mit den Krankenhausträgern (die politisch stützen) um den derzeit unzufriedenen KrankenhausärztInnen keine zu großen Zugeständnisse bei den aktuellen Arbeitsvertragverhandlungen machen zu müssen. Zum Teil koaliert das Management auch mit den PflegerInnen gegen die ÄrztInnen. In der aktuellen Zeit von knapper gewordenen Ressourcen haben sich auch die Arbeitsbedingungen von AnästhesistInenn verschlechtert. Beim derzeitigen Kampf um Ressourcen und in Hinblick auf die vorher vorgestellten Modelle zu Arbeitszufriedenheit ist nachvollziehbar dass Arbeitszufriedenheit, Leistungsmotivation und Commitmend der AnästhesistInnen in Österreich derzeit leiden (HERZBERG et al., 1959; ADAMS, 1965, 267-299; MCCLELLAND, 1975; CZIKZSENTMIHALYI, 2004; STAHL, 2013a, 2013b). Aus diesem Grund ist es

wichtig, dass Krankenhausträger und Management bald möglichst die Verteilungskämpfe gegen die ÄrztInnen einstellen und faire und gerechte Verträge vorlegen und akzeptable Arbeitsbedingungen ermöglichen.

9.2.3. Nachhaltige Organisationsgestaltung und Führung im Krankenhaus

Die Organisationsgestaltung im Krankenhaus sollte sich an Zielen ausrichten, die folgenden Merkmalen gerecht werden:

- Management sollte by Objectives erfolgen (zum Beispiel mittels Zielvereinbarungen und -erreichung, Entwicklungs- und Konfliktgespräche).
- Ungerechtigkeit sollte vermieden werden.
- Die Führung im Krankenhausbereich sollte durchaus nach betriebswirtschaftlichen Kriterien (das heißt Kostenminimierung, Gewinn Absicht und optimale Ressourcennutzung) erfolgen, zugleich aber die Grundzüge einer balancierten Führung berücksichtigen.
- Führung sollte balanciert sein. Ein balanciertes Führungsverhalten zeichnet sich dadurch aus, dass kritische Dualitäten der Führung wie zum Beispiel Misstrauen vs. Vertrauen, Konkurrenz und Kooperation, Bewahren und Verändern in einem Unternehmen wie dem Krankenhaus koexistieren können (STAHL, 2013c). Die Führungsperson muss sich also nicht für den radikalen Weg einer Seite entscheiden sondern akzeptiert- situationsabhängig- beide Dualitäten (Abbildung 24).

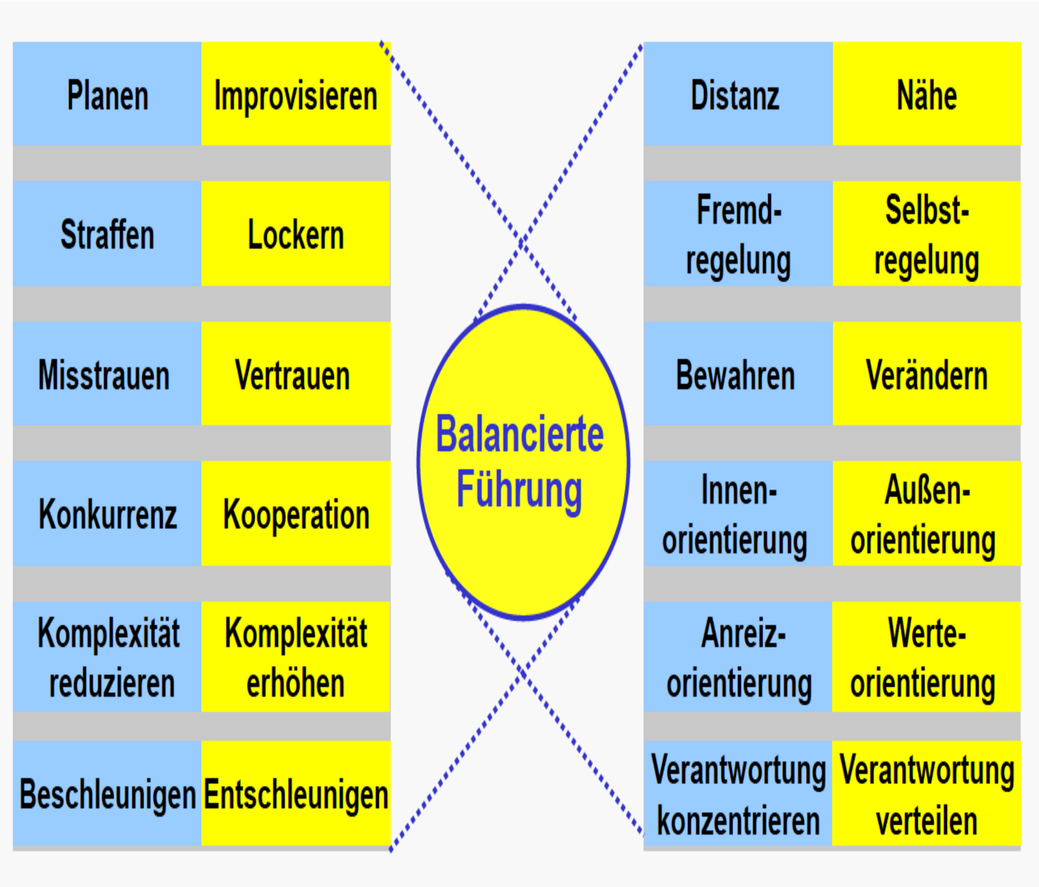

Abbildung 24. Balancierte Führung akzeptiert beide kritischen Dualitäten: Die Führungskraft entscheidet sich nicht für eine Seite der Führung (zum Beispiel *nur* Planen), sondern wählt der Situation entsprechend aus einer der beiden Seiten der Führung aus (zum Beispiel *sowohl* Planen *als auch* Improvisieren) (STAHL, 2013c).

Ein praktisches Beispiel dazu ist der Umgang mit AnästhesistInnen in einer Univeristätsklinik. Im Rahmen einer klassischen Führung wird eine Führungskraft die Entscheidungsmöglichkeiten beschneiden und die Führung straffen, im Rahmen einer balancierten Führung wird eine Führungskraft entsprechend der Situation die Führung lockern (zum Beispiel im klinischen Alltag) und bei Bedarf straffen (zum Beispiel in einer Krisensituation wie zum Beispiel bei Behandlung einer bekannten Persönlichkeit oder einem schweren klinischen Zwischenfall mit Todesfolge).

- Entsprechend dem Können der AnästhesistInnen (sie sind Experten in ihrem Arbeitsgebiet) und der aktuellen Situation in einem Krankenhaus (zum Beispiel Alltag vs. Schwerwiegendes Ereignis) kann die Führung in den meisten Fällen wie in einem Netzwerk von Experten oder einer Adhokratie erfolgen. Im Falle einer Katastrophe kann auf eine Hierarchie Führung gewechselt werden da dann ein starker Führer gebraucht wird (Tabelle 5).

**Hierarchie**	Es herrschen Über- und Unterordnung, Entscheidungen werden schnell gefällt, wenn das Subsidaritätsprinzip befolgt wird ist der Kommunikationsaufwand gering	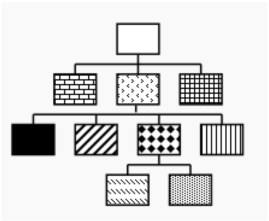
**Heterarchie**	Entspricht der Funktionsweise des Gehirns, Koordination erfolgt gleichberechtigt, keine Über- und Unterordnung, die Machtverhältnisse sind ausgeglichen	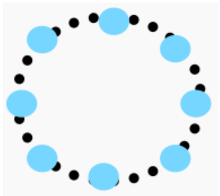
**Netzwerk**	Hohe Flexibilität, niedrige Transaktionskosten und –risiken, gesunde Mischung aus Vertrauen und Verträgen, Mitarbeiter müssen für diese Führung geeignet und vorbereitet sein	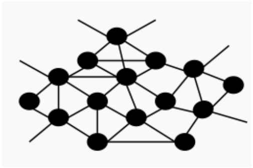
**Adhokratie**	Organische Struktur, Abläufe und Verhalten nicht nach Standard, Verhalten informell, hohe Berufsspezialisierung, Fachwissen wird sehr betont	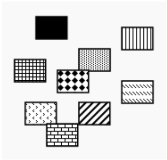
**Virtuelle Organisation**	Ist ein Zusammenschluss rechtlich unabhängiger Personen oder Organisatione, verbunden durch Informationstechnologie, jeder Partner trägt nur seine Kernkompetenzen	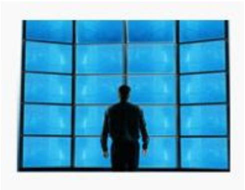

Tabelle 5. Idealtypische Strukturen der Führung, adaptiert von (STAHL, 2013c)

- Weiters sollten die Unternehmenskultur und –strukturen die Freiheitsgrade Sollen und Dürfen eines Mitarbeiters maximal unterstützen (Abbildung 26).

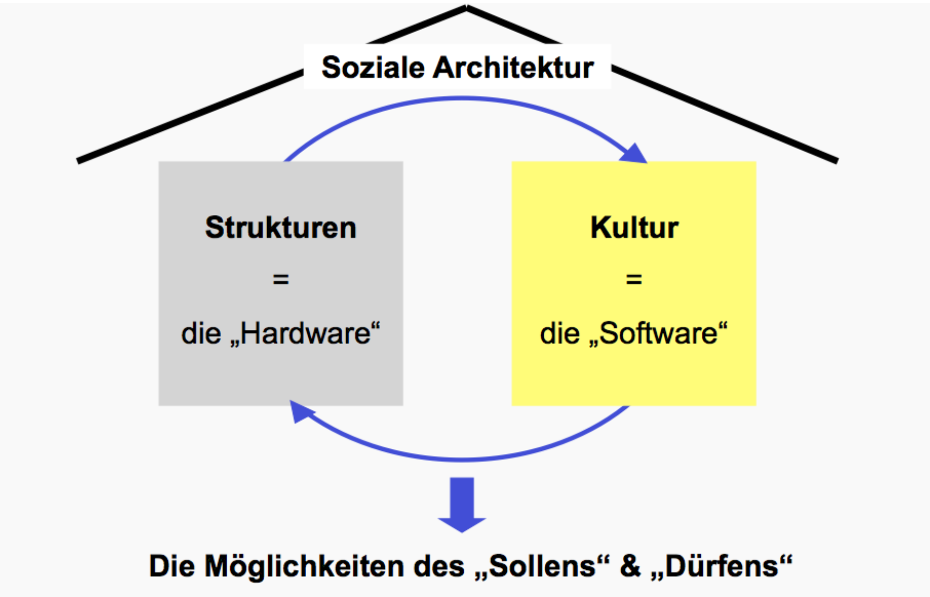

Abbildung 25. Die Soziale Architektur in einem Unternehmen kann Sollen und Dürfen maximal unterstützen (STAHL, 2013c).

- Für die Freiheitsgrade Sollen und Dürfen ist bei AnästhesistInnen das Krankenhaus verantwortlich. Werden die vier Freiheitsgrade eines Mitarbeiters maximal unterstützt so kann sich ein Anästhesist im Können, Wollen, Sollen und Dürfen optimal entfalten.

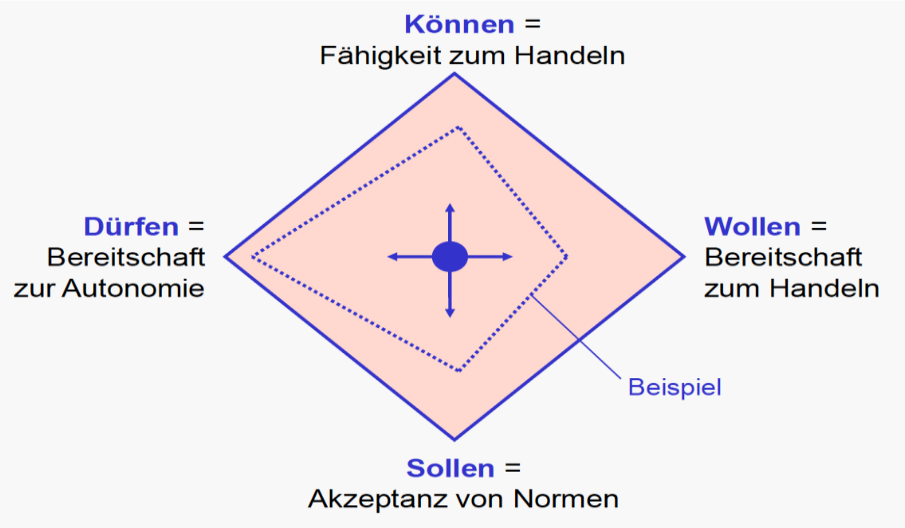

Abbildung 26. Die vier Freiheitsgrade Können, Wollen, Dürfen und Sollen aus (STAHL, 2013c).

- Um das Sollen und Dürfen zu erleichtern kann die Hierarchie im Krankenhaus durch Umstellung abgemildert werden (STAHL, 2013c) (Abbildung 27).

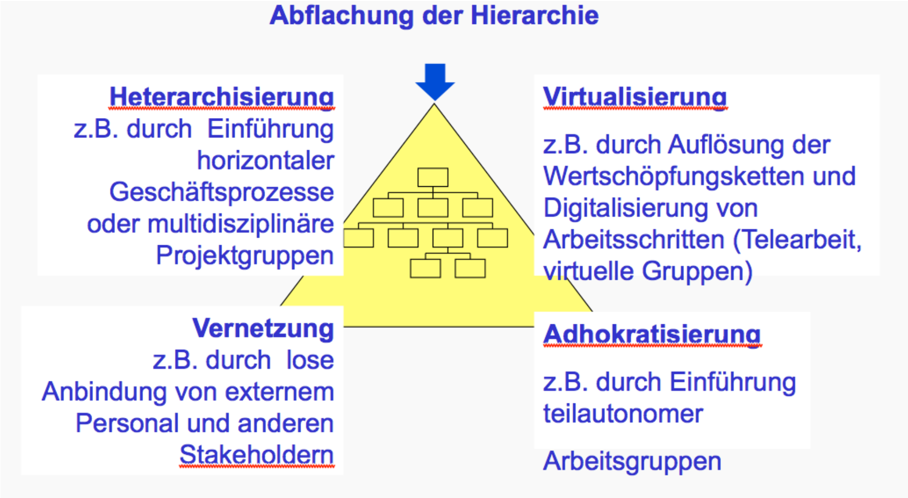

Abbildung 27. Abmilderung der Hierarchie um die vier Freiheisgrade „Können, Wollen, Dürfen, Sollen“ eines Anästhesisten zu erhöhen (STAHL, 2013c)

- Neben einer balancierten sollte ein postklassische Führung an Krankenhäusern ausgeübt werden, sie setzt sich zusammen aus und berücksichtigt (aus (STAHL, 2013c)):
  - Bescheidenheit, da Menschen nicht triviale Systeme sind, die sich nicht beliebig „lenken“ lassen.
  - Ursachen und Wirkungen sind zirkulär miteinander verbunden, das heißt, wer führt wird immer auch selbst geführt.
  - Die Dialektik des „Sowohl-als-auch“ ersetzt das Prinzip des „Entweder-oder“.
  - Führung lebt damit von Widersprüchen und Dilemmata.
  - Es gibt keine „objektive“ Wirklichkeit und damit auch keine „Eindeutigkeit“ auf die sich der Führende berufen kann.
  - „Rationales“ Handeln ist immer auch auf Emotionen angewiesen.
  - Ziele sollten die folgenden Merkmale besitzen: überlegt (richtiges Mass), herausgehoben (Vertrag), zeitlich definiert und begleitend (wiederholte Überprüfung anhand Teilziele) sein. Ziele sollten vorgegeben, ausgehandelt und freigegeben werden.

9.2.4. Konkrete Empfehlungen für die Organisationsgestaltung im Krankenhausbereich unter spezieller Berücksichtigung der Schleife Arbeitszufriedenheit- Leistungsmotivation- Commitment- Arbeitszufriedenheit

Konkret ergeben sich aus den Ergebnissen der Umfrage die dieser Arbeit zugrunde liegen fünf Schlussfolgerungen die in der Organisationsgestaltung beachtet werden sollten um Arbeitszufriedenheit, Leistungsmotviation und Commitment für AnästhesistInnen in Österreich zu steigern:

- Das Ansehen der AnästhesistInnen sollte gehoben werden, zum Beispiel durch Wertschätzung im Unternehmen, Marketing (Hervorheben der positiven Aspekte der Arbeit von AnästhesistInnen (zum Beispiel Interdisziplinarität und breites Spektrum der Arbeit, hohe Autonomie in den zugeteilten Aufgaben, erfüllende Arbeit).
- Der Zeitdruck und das Arbeitspensum sollten limitiert werden. Krankenhausträger sollten auf eine ausreichende Personaldecke achten, hierzu sind Maßnahmen wie zum Beispiel Marketing inkl. Employer Branding, Recruiting und Binden von AnästhesistInnen wichtig.
- Psychische Belastung sollte reduziert werden, zum Beispiel durch Coaching und Mentoring Programme. Es sollte Gesundheit gefördert und Krankheit durch Reduktion der Arbeitsbelastung vorgebeugt werden.
- Individuelles Führen ist bei der hohen und zunehmenden Vielfalt an Interessen der AnästhesistInnen notwendig. Die vier Freiheitsgrade eines Anästhesisten „Können, Wollen, Dürfen und Sollen“ sollen durch eine Experten gerechte Organisation des Krankenhauses (zum Beispiel Heterarchisierung oder Netzwerk) optimal gefördert werden.
- Arbeitszufriedenheit, Leistungsmotivation und Commitment von AnästhesistInnen sollte durch einen balancierten und postklassischen Führungsstil gesteigert werden.

10. Ausblick

Fast zwei Drittel der AnästhesistInnen die an dieser Umfrage teilgenommen haben haben in den letzten zwölf Monaten gearbeitet, obwohl sie krank waren. 51% Prozent sind der Meinung dass sie nicht gut gezahlt werden und nur 25% stimmen zu dass ihre Arbeit gute Karrierechance bietet. Achtundfünfzig Prozent geben an dass es leicht wäre eine Arbeit mit ähnlichem Gehalt zu finden, falls sie den aktuellen Job verlieren würden. Nun 29% Prozent bejahen dass die Organisation für die sie arbeiten sie motiviert das Beste zu geben, während 42% dies verneinen. 40% der AnäshtesistInnen sind der Meinung, dass KollegInnen anderer Fachdisziplinen und die Öffentlichkeit nur eine neutrale bis geringe Wertschätzung Ihnen gegenüber haben. Einige erachten die Arbeitszeiten als zu lang. Öfters haben AnästhesistInnen nicht genug Zeit zum Ruhen und Schlafen. Häufig leiden sie unter Muskelverspannung im Nacken- und Rückenbereich. Zudem leiden sie öfters an rascher Ermüdbarkeit und haben ein reduziertes sexuelles Interesse und Gefallen am Sex. Oft versuchen sie rascher zu arbeiten. Oft kommen sie auch spat in die Pause oder sie verzichten ganz darauf. Öfters kommen sie auch zu spat nach hause.

Diese Studie hat fünf zentrale Problembereiche bei AnästhesistInnen in Österreich gefunden welche von Seiten der Primarii der Anästhesiologie Abteilungen und Kliniken, Management und Krankenhausträgern berücksichtigt werden sollten um die Arbeitsbedingungen von AnästhesistInenn in Österreich zu verbessern:

Erstens sind viele AnästhesistInnen der Meinung dass der Stellenwert ihres Berufes bei KollegInnen anderer Fachgebiete und in der Bevölkerung gering bis neutral ist. Marketing- und “Branding”-Massnahmen sollten durchgeführt werden, um den Stellenwert der AnästhesistInnen zu eruieren und gegebenenfalls zu heben, zum Beispiel mittels Visiten der PatientInnen vor und nach der Anästhesie und der Ausgabe von Visitenkarten. Zusätzlich könnte die Wahrnehmung des Berufsstandes in der Bevölkerung durch Informationskampagnen angehoben werden.

Zweitens führen Zeitdruck und lange Arbeitszeiten zu einer Einschränkung der persönlichen und sozialen Freiheiten der AnästhesistInnen.

Darüberhinaus haben AnästhesistInnen oft ungenügend Zeit für Pausen, zum Essen, zur Ruhe und fur den Schlaf. Ihr Arbeitsethos ist dennoch so hoch, dass ca. zwei Drittel im Laufe des letzten Jahres gearbeitet haben, obwohl sie krank waren. Dass AnästhesistInnen arbeiten wenn sie krank sind, ist zwar lobenswert, in Zusammenschau aber mit der hohen Arbeitsintensität und der zu geringen Zeit für sich selbst und ihr soziales Umfeld und bereits bestehender Zeichen von Überlastung in bis zu 70% vorliegen sollte gezielt Augenmerk auf die Einhaltung der Pausen und der Arbeitszeite gelegt werden; zudem sollte das Ausmaß an zusätzlichen Aufgaben neben der klinischen Arbeit limitiert sein. Personal das für die pünktliche Auslöse sorgt könnte hier Entlastung bringen.

Drittens, die Arbeitszeiten werden von vielen als zu lang erachtet. Massnahmen gegen diesen Exzess wären zum Beispiel die strikte Einhaltung der vorgegebenen Arbeitszeiten, Limitierung der zusätzlichen Aufgaben welche neben der klinischen Arbeit anfallen, Anpassung der Bezahlung sodass ein gutes Auskommen möglich ist auch ohne dass AnästhesistInnen viele Nacht- und Wochenenddienste leisten müssen. Wichtig ist auch, dass die jüngeren Generationen (X bis Z) nicht mehr gewillt so viel zu arbeiten wie die Nachkriegs- und die Babyboomergeneration. Damit die Abteilungen und Kliniken für Anästhesiologie angepasst sind auf diese Generationen sollte Maßnahmen ergriffen werden wie zum Beispiel Flexibilisierung der Arbeitszeiten, der Karenzzeiten und Teilzeitarbeitsmöglichkeiten auch für Männer, Job Sharing auch für leitende Positionen, Kinderbetreuungsmöglichkeiten von Kindertagesheim bis hin zur Schule. Die Bezahlung für AnästhesistInnen sollte auf ein international konkurrenzfähiges Niveau angehoben werden.

Viertens gibt es alarmierende Zeichen geistiger und körperliche Überlastung bei den AnästhesistInnen in Österreich. Maßnahmen um hier entgegenzusteuern können sein: Ermöglichung von ausreichend Freizeit um soziale Kontakte zu pflegen und entspannenden Hobbies, wie zum Beispiel Sport, nachzugehen. Gesundes Essen (inkl. Gemüse und Obst) sollte auch an entlegenen Arbeitsplätzen angeboten werden.

Fünftens spüren AnästhesistInnen einen zunehmenden Produktivitätsdruck.

Während eine Produktivitätssteigerung in einigen Krankenhäusern notwendig erscheint, sind viele andere östereichische Krankenhäuser in den internationalen Produktivitätsrankings bereits ganz vorne. Der Versuch in diesen Häusern die Produktivität weiter zu forcieren kann hier das Gegenteil vom Erwarteten bewirken, da aufgrund von Druck und Überarbeitung oberflächliches Arbeiten, Überarbeitung und Burnout und mithin fatale Fehler resultieren können. Maßnahmen um Überarbeitung zu vermeiden könnten Standard Operating Procedures für häufige Krankheitsbilder, Ein- und Ausleitungszonen mit eigenen Anästhesieteams sein welche die Umlagerungszeiten in den Operationssälen deutlich verringern können ohne eine Qualitätseinschränkung zu bewirken.
Individuelles Führen ist bei der hohen und zunehmenden Vielfalt an Interessen der AnästhesistInnen notwendig. Die vier Freiheitsgrade eines Anästhesisten „Können, Wollen, Dürfen und Sollen“ sollen durch eine Experten-gerechte Organisation des Krankenhauses (zum Beispiel Heterarchisierung oder Netzwerk) optimal gefördert werden. Arbeitszufriedenheit, Leistungsmotivation und Commitment von AnästhesistInnen sollte durch einen balancierten und postklassischen Führungsstil gesteigert werden.

Krankenhausträger dürfen nicht vergessen dass sie mit Menschen arbeiten, welche nicht wie Dinge trivial sind. Eine betriebswirtschaftliche Denkweise im Krankenhaus unterstützt von einer balancierten und postklassischen Führung hat das Potential die Arbeitsbedingungen für AnästhesistInnen in Österreich- auch zum Wohl der Krankenhausträger- nachhaltig zu verbessern.

11. Literaturverzeichnis

2ASK. (2013). 2ask - Ihr Online-Umfrage-Tool. In: Amundis Communications GmbH www.2ask.de. Accesed on: 09262013.

ABC. (2013). ABC News. In: ABC http://a.abcnews.com/images/Health/gty_surgery_nt_111005_wg.jpg. Accesed on: 09272013.

ADAMS J. S. 1965. Inequity in Social Exchange. In: L. BERKOWITZ, L. BERKOWITZ.L. BERKOWITZs. Advances in Experimental Social Psychology. p 267-299.

ANONYM. 2013. Arbeitsbedingungen von AnästhesistInnen an der Universitätsklinik für Anästhesiologie Regensburg. In.

ANONYM. (2013). Spitalsärztebefragung 2013. Mitteilungen der Ärztekammer für Tirol:20-21.

ÄRZTEKAMMER_ÖSTERREICH. (2013). Standesmeldung (August 2013). Österreichische Ärzte Zeitung:47.

BADURA B., SCHRÖDER H., KLOSE J. and MACCO K. (2010). Fehlzeiten Report 2009, Arbeit und Psyche: Belastungen reduzieren- Wohlbefinden fördern. In: Springer.

BAKKER A. B., KILLMER C. H., SIEGRIST J. and SCHAUFELI W. B. (2000). Effort-reward imbalance and burnout among nurses. J Adv Nurs 31:884-891.

BAUER J., HAFNER S., KACHELE H., WIRSCHING M. and DAHLBENDER R. W. (2003). [The burn-out syndrome and restoring mental health at the working place]. Psychother Psychosom Med Psychol 53:213-222.

BEER M. (2011). Higher Ambition: How great leaders create economic and social value. . In: McGraw Hill Professional.

BERTELSMANN. (2013). Bertelsmann BKK Versicherung. In: http://www.bertelsmann-bkk.de/. Accesed on: 09272013.

BOYNTON P. M. (2004). Administering, analysing, and reporting your questionnaire. BMJ 328:1372-1375.

BOYNTON P. M. and GREENHALGH T. (2004). Selecting, designing, and developing your questionnaire. BMJ 328:1312-1315.

BRAUN M., C S.-L. and KESSLER H. (2008). Burnout, Depression und Substanzgebrauch bei deutschen Psychiatern und Nervenärzten.

Nervenheilkunde:800-804.

BREHM J. (1966). Theory of psychological reactance. In: Academic Press. 978-0121298500

BRUGGEMANN A., GROSKURTH P. and ULICH E. (1975). Arbeitszufriedenheit. In: Hans Huber.

BUCHANAN D. and HUCZYNSKI A. 2010. Teamworking. In: Organizational Behaviour. Harlow: Pearson Education Limited. p 385-416.

BUDDEBERG-FISCHER B., KLAGHOFER R., STAMM M., SIEGRIST J. and BUDDEBERG C. (2008). Work stress and reduced health in young physicians: prospective evidence from Swiss residents. Int Arch Occup Environ Health 82:31-38.

BURNS K. E., DUFFETT M., KHO M. E., MEADE M. O., ADHIKARI N. K., SINUFF T. and COOK D. J. (2008). A guide for the design and conduct of self-administered surveys of clinicians. CMAJ 179:245-252.

CHIRON B., MICHINOV E., OLIVIER-CHIRON E., LAFFON M. and RUSCH E. (2010). Job satisfaction, life satisfaction and burnout in French anaesthetists. J Health Psychol 15:948-958.

CZIKZSENTMIHALYI M. (2004). Flow im Beruf: Das Geheimnis des Glücks am Arbeitsplatz. In: Klett-Cotta Verlag. 978-3608935325

CZIKZSENTMIHALYI M. (2004). Flow im Beruf: Das Geheimnis des Glücks am Arbeitsplatz. In: Klett-Cotta Verlag. 978-3608935325

DE OLIVIERA G. S., JR., CHANG R., FITZGERALD P. C., ALMEIDA M. D., CASTRO-ALVES L. S., AHMAD S. and MCCARTHY R. J. (2013). The prevalence of burnout and depression and their association with adherence to safety and practice standards: a survey of United States anesthesiology trainees. Anesth Analg 117:182-193.

DERKZEN S. and LOEBENSTEIN C. (2013). Zeitjob in der Chefetage. DIE ZEIT:27.

EMBRIACO N., AZOULAY E., BARRAU K., KENTISH N., POCHARD F., LOUNDOU A. and PAPAZIAN L. (2007). High level of burnout in intensivists: prevalence and associated factors. Am J Respir Crit Care Med 175:686-692.

FAHRENKOPF A. M., SECTISH T. C., BARGER L. K., SHAREK P. J., LEWIN D., CHIANG V. W., EDWARDS S., WIEDERMANN B. L. and LANDRIGAN C. P.

(2008). Rates of medication errors among depressed and burnt out residents: prospective cohort study. BMJ 336:488-491.

FREUDENBERGER H. (1974). Staff Burn-Out. J Soc Issues 30:159-165.

GEBERT D. and ROSENSTIEL L. (2000). Organisationspsychologie: Person und Organisation. In: Kohlhammer W. 978-3170132726

GEISLER J. (1977). Psychologie der Karriere. In: rororo.

GLOUBERMAN S. and MINTZBERG H. (2001). Managing the Care of Health and the Cure of Disease – Part I: Differentiation. Health Care Management Review 26 56-69.

HERZBERG F. (2003). One more time: How do you motivate employees? Harvard Business Manager.

HERZBERG F., MAUNER B. and SYNDERMAN B. (1959). The motivation to work. In:

HILLERT A., KOCH S. and HEDLUND S. (2007). Stressbewältigung am Arbeitsplatz. Ein stationäres berufsbezogenes Gruppenprogramm. In: Vandenhoeck&Ruprecht.

JESKE H. C., LEDERER W., LORENZ I., KOLBITSCH C., MARGREITER J., KINZL J. and BENZER A. (2001). The impact of business cards on physician recognition after general anesthesia. Anesth Analg 93:1262-1264.

KANTER R. (2012). Anders Wirtschaften. Harvard Business Manager:26-39.

KELLEY K., CLARK B., BROWN V. and SITZIA J. (2003). Good practice in the conduct and reporting of survey research. Int J Qual Health Care 15:261-266.

KINZL J. F., KNOTZER H., TRAWEGER C., LEDERER W., HEIDEGGER T. and BENZER A. (2005). Influence of working conditions on job satisfaction in anaesthetists. Br J Anaesth 94:211-215.

KINZL J. F., TRAWEGER C., BIEBL W. and LEDERER W. (2006). [Burnout and stress disorders in intensive care doctors]. Dtsch Med Wochenschr 131:2461-2464.

KINZL J. F., TRAWEGER C., TREFALT E., RICCABONA U. and LEDERER W. (2007). Work stress and gender-dependent coping strategies in anesthesiologists at a university hospital. J Clin Anesth 19:334-338.

LEDERER W., KINZL J. F., TRAWEGER C., DOSCH J. and SUMANN G. (2008). Fully

developed burnout and burnout risk in intensive care personnel at a university hospital. Anaesth Intensive Care 36:208-213.

LEDERER W., KINZL J. F., TREFALT E., TRAWEGER C. and BENZER A. (2006). Significance of working conditions on burnout in anesthetists. Acta Anaesthesiol Scand 50:58-63.

LINZER M., MCMURRAY J. E., VISSER M. R., OORT F. J., SMETS E. and DE HAES H. C. (2002). Sex differences in physician burnout in the United States and The Netherlands. J Am Med Womens Assoc 57:191-193.

MARKARITZER K. (2009). Josef Margreiter: Erfolg nach dem Tod. Österreichische Ärzte Zeitung 1.

MAYERHOFER M. 2013. Mikropolitische Ziele und Mittel von Ärztinnen und Ärzten im Krankenhaus. In: Wirtschaftsuniversität Wien, Studiengang MBA für Health Care Management. p 326.

MCCLELLAND D. (1975). Power: The inner experience. In:

MICHALSEN A. and HILLERT A. (2011). [Burn-out in anesthesia and intensive care medicine. Part 1. Clarification and critical evaluation of the term]. Anaesthesist 60:23-30.

MICHALSEN A. and HILLERT A. (2011). [Burnout in anesthesia and intensive care medicine. Part 2: Epidemiology and importance for the quality of care]. Anaesthesist 60:31-38.

MORAIS A., MAIA P., AZEVEDO A., AMARAL C. and TAVARES J. (2006). Stress and burnout among Portuguese anaesthesiologists. Eur J Anaesthesiol 23:433-439.

NINDLER P. (2013). Für 40 % der Spitalsärzte hat sich die Arbeit verschlechtert. Tiroler Tageszeitung.

NYSSEN A. S. and HANSEZ I. (2008). Stress and burnout in anaesthesia. Curr Opin Anaesthesiol 21:406-411.

NYSSEN A. S., HANSEZ I., BAELE P., LAMY M. and DE KEYSER V. (2003). Occupational stress and burnout in anaesthesia. Br J Anaesth 90:333-337.

ÖGARI. (2013). Österreichische Gesellschaft für Anästhesie, Reanimation und Intensivmedizin. In: ÖGARI http://www.oegari.at/. Accesed on: 09262013.

OTTENSCHLÄGER M. (2013). Vom Halbgott zum Manager in Weiss. DIE

ZEIT:89.

PADOSCH S. A., SCHMIDT C. E. and SPOHR F. A. (2011). [Retention management by means of applied human resource development: lessons from cardiovascular anaesthesiology]. Anasthesiol Intensivmed Notfallmed Schmerzther 46:364-369.

PIRCHER A. (2011). Mit Sinn zum nachhaltigen Erfolg. In: Erich Schmitt Verlag. 3. Auflage. 978 3 503 129133

PONCET M. C., TOULLIC P., PAPAZIAN L., KENTISH-BARNES N., TIMSIT J. F., POCHARD F., CHEVRET S., SCHLEMMER B. and AZOULAY E. (2007). Burnout syndrome in critical care nursing staff. Am J Respir Crit Care Med 175:698-704.

PRINS J. T., VAN DER HEIJDEN F. M., HOEKSTRA-WEEBERS J. E., BAKKER A. B., VAN DE WIEL H. B., JACOBS B. and GAZENDAM-DONOFRIO S. M. (2009). Burnout, engagement and resident physicians' self-reported errors. Psychol Health Med 14:654-666.

SALFELD R., HEHNER S. and WICHELS R. (2009). Modernes Krankenhausmanagement. In: Springer. 978 3 540 873983

SCHMIDT C. E., MOLLER J., SCHMIDT K., GERBERSHAGEN M. U., WAPPLER F., LIMMROTH V., PADOSCH S. A. and BAUER M. (2011). [Generation Y : recruitment, retention and development]. Anaesthesist 60:517-524.

SCHMIDT C. E., MOLLER J., SCHMIDT K., GERBERSHAGEN M. U., WAPPLER F., LIMMROTH V., PADOSCH S. A. and BAUER M. (2012). [Generation 55+: leadership and motivation of generations in hospitals]. Anaesthesist 61:630-634, 636-639.

SCHNEEWIND K. and WEIß J. (1999). Der Fragebogen "Gesundheit und Streß". Institut für Psychologie der Universität München. Bern: Huber. .

SEMMER N., ZAPF D. and DUNCKEL H. 2007. ISTA - Instrument zur Stressbezogenen Arbeitsanalyse. In.

SHANAFELT T. D., BRADLEY K. A., WIPF J. E. and BACK A. L. (2002). Burnout and self-reported patient care in an internal medicine residency program. Ann Intern Med 136:358-367.

STAHL H. (2013a). Führungswissen. In: Erich Schmidt Verlag. 978 3 503 144181

STAHL H. (2013b). Leistungsmotivation in Organisationen. In: Erich Schmidt Verlag. 2nd. . 978 3 503 144044

STAHL H. 2013c. Vorlesungsunterlagen. In.

VERDON M., MERLANI P., PERNEGER T. and RICOU B. (2008). Burnout in a surgical ICU team. Intensive Care Med 34:152-156.

WEINERT A. (1998). Lehrbuch der Organisationspyschologie. In: BeltzPVU. 4. Auflage. 978-3621274098

WEST C. P., TAN A. D., HABERMANN T. M., SLOAN J. A. and SHANAFELT T. D. (2009). Association of resident fatigue and distress with perceived medical errors. JAMA 302:1294-1300.

WIELAND R., KRAJEWSKI J. and MEMMOU M. 2005. Arbeitsgestaltung, Persönlichkeit und Arbeitszufriedenheit. In: L. Fischer, L. Fischer.L. Fischers. Arbeitszufriedenheit- Konzepte und empirische Befunde. 978-3801717308.

12. Eidesstattliche Erklärung

Ich erkläre hiermit an Eides statt, dass ich die vorliegende Master´s Thesis selbstständig angefertigt habe. Die aus fremden Quellen direkt oder indirekt übernommenen Gedanken sind als solche kenntlich gemacht.

Die Arbeit wurde bisher weder in gleicher noch in ähnlicher Form einer anderen Prüfungsbehörde vorgelegt und auch nicht veröffentlicht.

Innsbruck, 22.10.2013

Ort, Datum Unterschrift
