# Supplementary material for: Consolidation of working hours and work-life balance in anaesthesiologists – A cross-sectional national survey
Source: PLoS One. 2018 Oct 31;13(10):e0206050. doi: 10.1371/journal.pone.0206050 (PMC6209218; doi:10.1371/journal.pone.0206050)
Supplement: S1 Fig — (PDF) [file pone.0206050.s002.pdf]

# Arbeitszufriedenheit von Ärzten am Beispiel der Anästhesisten

Die Anästhesie gehört zu jenen Bereichen der Medizin, die aufgrund der anspruchsvollen Arbeitsbedingungen den idealen Nährboden für chronische Überlastung und psychische Erschöpfung liefern. Wie ist das in Österreich?

Peter Paal, Heinz K. Stahl

## Anästhesie – das unbekannte Wesen?

Seit nunmehr sechzig Jahren gibt es die Ausbildung zum Facharzt für Anästhesie und Intensivmedizin. Dabei wird oft übersehen, dass dessen Arbeit auch die Notfall- und Schmerzmedizin umfasst. Er geht zudem täglich mit hochwirksamen und schnell wirkenden Pharmaka um. Ohne Anästhesistinnen und Anästhesisten würde die moderne Medizin, wie wir sie heute kennen und schätzen, nicht funktionieren. Da sich die Patienten im Operationssaal und in der Intensivstation meist in einem schlafähnlichen Zustand befinden, wenn der Anästhesist sie behandelt, wird sein Handeln kaum wahrgenommen. Besonders im Vergleich mit dem Chirurgen, wirkt er unauffällig. Vielfach werden Anästhesisten auch heute noch als „Gasleute“ bezeichnet. Es haftet ihnen der Ruf an, keine „richtigen“ Ärzte zu sein.

So unfair können Klischees sein. Dabei gehört die Anästhesie zu jenen Bereichen der Medizin, die aufgrund der anspruchsvollen Arbeitsbedingungen den idealen Nährboden für chronische Überlastung und psychische Erschöpfung liefern. Zahlreiche ausländische Studien weisen darauf hin. Ist das in Österreich, mit der höchsten Ärztedichte in der Europäischen Union, anders?

## AnästhesistInnen in Österreich unter die Lupe

In Zusammenarbeit mit Wolfgang Lederer (Univ. Klinik für Anästhesie und Intensivmedizin Innsbruck, Medizinische Universität Innsbruck) und Johann Kinzl (Univ. Klinik für Psychosomatik und Psychiatrie, Medizinische Universität Innsbruck) wurden die Arbeitsbedingungen der Anästhesisten in Österreich untersucht, um daraus erste Schlussfolgerungen zu ziehen. Befragt wurden jene 1.145 Anästhesisten, die bei der Österreichischen Gesellschaft für Anästhesiologie, Reanimation und Intensivmedizin (ÖGARI) zum Zeitpunkt der Untersuchung als Mitglieder gemeldet und in den zwölf Monaten vor der Befragung in Österreich anästhesiologisch tätig waren. 394 (34,4 %) vollständige Antworten konnten gewonnen werden. Die meisten Befragten waren Fachärzte, während sich 113 noch in der Ausbildung zum Facharzt befanden. Ein Drittel der Befragten war in leitender Position tätig. Die Zahl der jährlichen Narkosen in den Krankenhäusern betrug bei knapp 40 % der Befragten mehr als 10.000.

## Wenig soziale Anerkennung

Anästhesisten empfinden das Ansehen, das sie bei Kollegen anderer Fachdisziplinen und in der Bevölkerung genießen, als neutral bis gering. Möglicherweise liegt das daran, dass sie sich als „Appendix“ der chirurgischen Fächer fühlen. Vor allem können Anästhesisten im Vergleich zu Mitgliedern anderer Fachdisziplinen Patienten in der Regel nicht heilen. Sie sind unterstützend tätig, damit andere Fachärzte die kurative Intervention durchführen können.

## Zeitmangel und Arbeitsdruck

Das Arbeitstempo ist hoch und wechselnde Arbeitsschichten sind an der Tagesordnung. Die Zeitspanne, innerhalb derer Anästhesisten die Arbeit verlassen können, bemisst sich meist nur in Minu-

**Abb. 1: Ein Modell der Arbeitszufriedenheit.** In Anlehnung an Agnes Bruggemann (Bruggemann/Großkurth/Ulrich, Bern 1975)

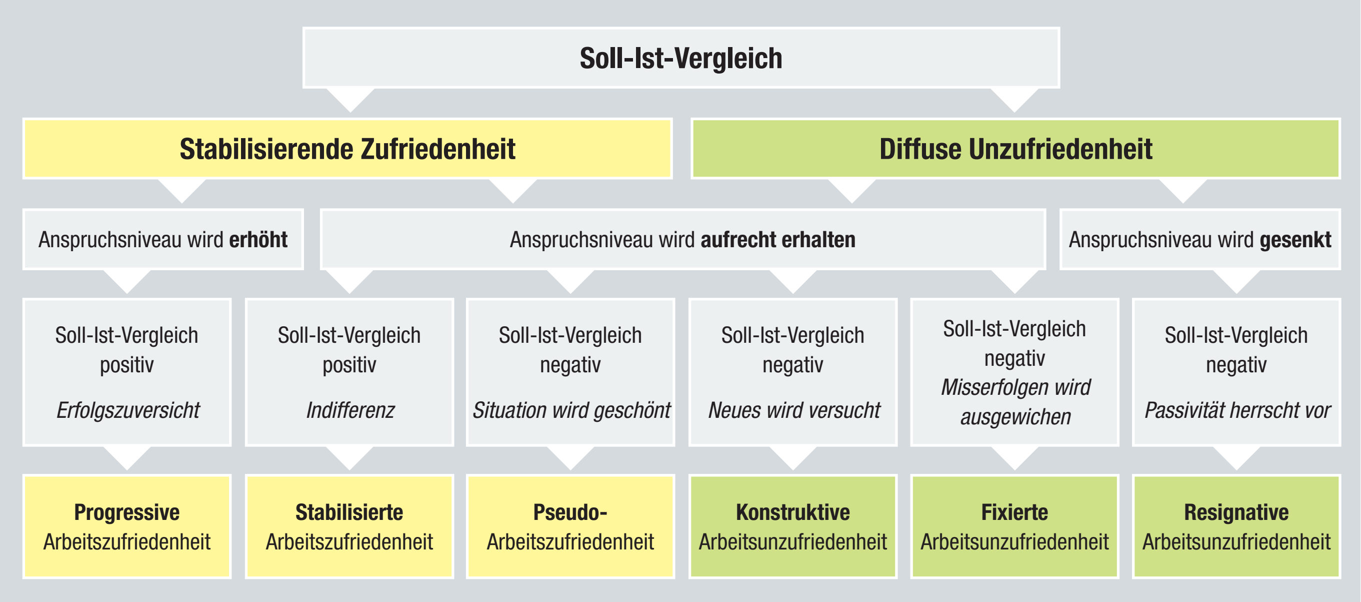


ten. Die Tätigkeit wird häufig durch Vorgesetzte, Kollegen oder Unerwartetes unterbrochen. Auch müssen mehrere Schritte gleichzeitig durchgeführt werden. Dieses „Multitasking“ (neurobiologisch betrachtet gibt es keine „Gleichzeitigkeit“, der präfrontale Kortex wechselt „nur“ sehr rasch zwischen einzelnen Aufgaben hin und her) macht die Arbeit mental fordernd. Anästhesisten müssen schnell reagieren und viele Details im Kopf behalten. Sie können die Arbeitsprozesse kaum beeinflussen, sind aber bei der Durchführung der zugeteilten Arbeit autonom.

## Krankmachende Bedingungen

Die Arbeitszeiten sind lang und häufig fehlt ausreichend Zeit zum Ausruhen, Essen oder Schlafen. Anästhesisten leiden regelmäßig an Ein- und Durchschlafstörungen, Muskelverspannungen und Rückenschmerzen. Aus den Antworten zu schließen, sind sie oft ruhelos und ungeduldig; selten ganz entspannt. Mit jemandem über ihre Empfindungen und Probleme zu sprechen, erweist sich für sie als schwierig. Auffallend ist eine pessimistische Grundhaltung: Viele fürchten, dass der Tag nicht gut wird. 10 % sind sogar immer oder sehr häufig niedergeschlagen. Die Furcht, dass Produktivitätsdruck und Überarbeitung zu Fehlern führen können, ist ausgeprägt.

## Das Problem der „Hygienefaktoren“

Die Sorge, die Arbeit zu verlieren, ist bei den Befragten zwar gering. Für ihre Arbeit auch gut bezahlt zu werden, glauben jedoch nur wenige. Auch die Karrierechancen beurteilen sie als eher „durchwachsen“. Die häufige Antwort, dass es einfach sei, einen anderen Arbeitsplatz mit ähnlicher Bezahlung zu finden, lässt auf eine hohe Wechselbereitschaft schließen. Anästhesisten sind häufig trotz Krankheit bei der Arbeit, sie „beißen sich durch“. Fast die Hälfte der Befragten fühlt sich in der eigenen Organisation nicht zu Hause und vermisst dort „gute Freunde“. Die „Hygienefaktoren“ (sie führen im klassischen Zufriedenheitsmodell des Psychologen Frederick Herzberg bei Nichterfüllung zu hoher Unzufriedenheit) sind also entweder gar nicht bekannt oder sie werden zu wenig berücksichtigt.

## Die Crux der Arbeitszufriedenheit

So trivial dieser Begriff zunächst anmutet, so fundamental ist seine Bedeutung für zwei Schlüsselfaktoren der Arbeitswelt: die *Leistungsmotivation* (das Streben nach Erfolg bzw. Vermeidung von Misserfolg, verbunden mit der Neigung, die eigene Tüchtigkeit an einem Gütemaßstab zu messen) und das *Commitment* (die Bereitschaft, sich an eine Aufgabe oder Organisation zu binden). Sind beide ausgeprägt positiv, so legt dies nahe, dass Menschen wohl einen „psychologischen Arbeitsvertrag“ mit ihrer Organisation abgeschlossen haben. Solche impliziten Verträge liefern genau jenen „Mehrwert“, ohne den eine (nicht subventionierte) Organisation langfristig nicht überlebensfähig ist.

Die gegenständliche Untersuchung über Anästhesistinnen und Anästhesisten in Österreich förderte zutage, dass es bei der Leistungsmotivation knirscht und das Commitment eher abnimmt. Die Ursache dafür könnte in einer *diffusen* Arbeitsunzufriedenheit der Anästhesisten liegen (siehe Abb. 1). Sie bleibt bei der Messung von

Arbeitszufriedenheit häufig unentdeckt oder wird, weil ein offener Widerspruch der Betroffenen fehlt, als „indifferent“ oder „normal“ eingestuft. Eine solche diffuse Unzufriedenheit kann sich unterschiedlich auf die Einstellung zur Leistung auswirken. Im besten Fall fällt der Vergleich zwischen Erwartungen und erlebten Tatsachen (noch) positiv aus und das gewohnte Anspruchsniveau wird beibehalten. Hier sind weder Begeisterung noch ein „Grummeln“ zu erwarten.

Anders reagieren Menschen mit einem starken impliziten Leistungsmotiv. Sie behalten auch bei enttäuschten Erwartungen ihr Anspruchsniveau zunächst bei und probieren, die Situation durch eigenen Antrieb und die Suche nach anderen Wegen zu verbessern. Dieser Frustrationspuffer einer *konstruktiven* Arbeitsunzufriedenheit ist allerdings irgendwann aufgebraucht. Manche scheiden aus dem Krankenhausbetrieb aus, während andere Unzufriedene danach trachten, Misserfolge zu vermeiden und neue Lösungsversuche gar nicht erst zu riskieren. Die Arbeitsunzufriedenheit wird auf diese Weise *fixiert*.

Der Unzufriedene kann sich auch die Situation schönfärben, indem er z.B. ein anderes Arbeitsumfeld als noch schlechter als das eigene bewertet. Eine solche *Pseudo*-Arbeitszufriedenheit ist brüchig und kann sogar in Aggression gegen den Arbeitgeber kippen. Letztendlich spitzt sich alles ins Negative zu; *resignative* Arbeitsunzufriedenheit stellt sich ein. Diese geht oft mit dem Gefühl des Kontrollverlusts einher und kann durchaus in „erlernter Hilflosigkeit“, einer Vorstufe der Depression, münden.

## Was ist zu tun?

Die Arbeitszufriedenheit von Anästhesisten sollte regelmäßig gemessen werden. Multiattributives Abfragen, wie dies in Konsumgüter- und Dienstleistungsbereichen üblich ist, führt hier nicht zum Ziel. Vielmehr steht mit der „Methode der kritischen Ereignisse“ (Critical Incident Technique CIT) ein Instrument zur Verfügung, das sich sehr zweckmäßig auf die arbeitsteiligen Prozesse im Krankenhaus anwenden lässt. Dabei werden die Probanden eingeladen, über besondere Vorkommnisse zu reflektieren und die erlebte Differenz, im positiven wie negativen Sinne, zwischen Erwartungen und Tatsachen offenzulegen. Mithilfe einer (erlernbaren) qualitativen Interviewführung ist es möglich, auf die unabdingbaren Hygienefaktoren und andere Voraussetzungen für eine Verbesserung der Arbeitszufriedenheit zu schließen.

Das Ansehen der Anästhesisten muss gehoben werden. Im Innenverhältnis, indem man z.B. die Interdisziplinarität, das breite Spektrum der Arbeit und die Rolle in der Risikoabklärung verdeutlicht. Nach außen hin muss offensichtlich werden, welchen Beitrag Anästhesisten für das Gelingen der arbeitsteiligen Prozesse im Krankenhaus leisten. Beiträge in relevanten Medien sowie Visiten der Patienten vor und nach der Anästhesie mit der Übergabe von Visitenkarten könnten dabei helfen.

Die Abläufe sind so zu gestalten, dass Pausen und Arbeitszeiten eingehalten werden und das Ausmaß an zusätzlichen Aufgaben neben der klinischen Arbeit limitiert wird. Standard Operating Procedures für häufige Krankheitsbilder können dabei ebenso helfen wie zentrale Ein- und Ausleitungszonen in den OP-Zonen, was auch die Umlagerungszeiten in den Operationssälen ohne Qualitätseinbußen deutlich verringern könnte. Vielen österreichischen Krankenhäusern wird im internationalen Vergleich eine hohe Produktivität bescheinigt. Weitere Produktivitätssteigerungen dürfen jedoch nicht zu Lasten der Anästhesisten erfolgen.

Der psychischen Überforderung sollte durch individuelles *Coaching* (Hilfe zur Selbsthilfe = „learning *with*“) oder *Mentoring* (Weitergabe von Erfahrung = „learning *from*“) vor allem für Berufseinsteiger entgegengewirkt werden. Auch Teamtraining kann hierzu, über seine Bedeutung für das Risikomanagement hinaus, einen Beitrag leisten. Die sogenannten Generationen X, Y und Z (Alterskohorten der Geburtsjahrgänge ab etwa 1966) sind nicht mehr gewillt, wie die Wirtschaftswunder- und die Babyboomer-Generation, sich für die Arbeit aufzuopfern („Die Familie kommt vor dem Lohn“). Forderungen nach flexiblen Arbeitszeiten, Teilzeitarbeit auch für Männer, Job Sharing und Kinderbetreuung dürfen nicht als Zumutung gelten. Kinderbetreuung ist besonders wichtig, um die Anästhesiologie für Frauen attraktiver zu gestalten.

Zum Schluss ein Punkt, der nicht nur die Anästhesisten betrifft. Die Führung im Krankenhaus darf nicht mehr von der Blaupause eines für alle und alles geltenden Führungsverhaltens ausgehen. Führung muss individualisiert werden. Gerade bei den Anästhesisten sind jene vier Dimensionen sichtbar, an denen sich eine individualisierte Führung ausrichten sollte: Das *Können* (die fachlichen Fähigkeiten & Fertigkeiten), das *Wollen* (die Bereitschaft, aus eigenem Antrieb zum Wohle des Ganzen tätig zu werden), das *Dürfen* (das Erkennen von Freiräumen, die es zum Wohle der Organisation zu nutzen gilt) und das *Sollen* (die Verinnerlichung der Werte und Normen der eigenen Organisation). ■

Literatur:
- PAAL, P. (2013): Arbeitsbedingungen von AnästhesistInnen in Österreich. Master's Thesis, Management Center Innsbruck.
- STAHL, H. K. (2013): Leistungsmotivation in Organisationen. 2. Aufl., Berlin.

**AO. UNIV.-PROF. DR. HEINZ K. STAHL**

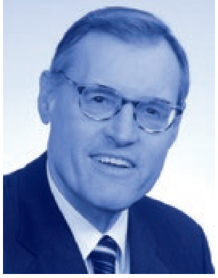

**Interdisziplinäre Abteilung für Verhaltenswissenschaftlich Orientiertes Management, Wirtschaftsuniversität Wien**
**hks-research@utanet.at**
